# Design, Synthesis, Molecular Modeling, Biological Activity, and Mechanism of Action of Novel Amino Acid Derivatives of Norfloxacin

**DOI:** 10.1021/acsomega.3c07221

**Published:** 2023-11-01

**Authors:** Ahmed M. Kamal El-sagheir, Ireny Abdelmesseh Nekhala, Mohammed K. Abd El-Gaber, Ahmed S. Aboraia, Jonatan Persson, Ann-Britt Schäfer, Michaela Wenzel, Farghaly A. Omar

**Affiliations:** † Medicinal Chemistry Department, Faculty of Pharmacy, Assiut University, Assiut 71526, Egypt; ‡ Division of Chemical Biology, Department of Life Sciences, Chalmers University of Technology, Gothenburg 412 96, Sweden; § Center for Antibiotic Resistance Research in Gothenburg (CARe), Gothenburg 405 30, Sweden

## Abstract

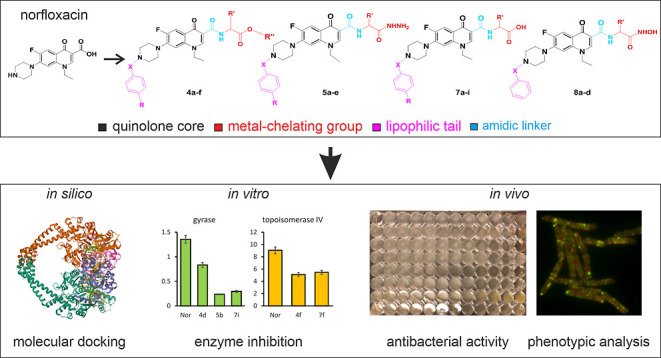

Two series of *N*4-substituted piperazinyl amino acid derivatives of norfloxacin (24 new compounds) were designed and synthesized to attain structural surrogates with additional binding sites and enhanced antibacterial activity. Synthesized derivatives showed increased antibacterial and antimycobacterial activity compared to their lead structure, norfloxacin. Molecular modeling studies supported the notion that the derivatives can establish additional bonds with the target enzymes gyrase and topoisomerase IV. *In vitro* enzyme inhibition assays confirmed that the tested compounds were significant inhibitors of these enzymes. Inhibition of gyrase and topoisomerase IV was then confirmed in living bacterial cells using bacterial cytological profiling of both Gram-negative *Escherichia coli* and Gram-positive *Bacillus subtilis*, revealing a typical topoisomerase inhibition phenotype characterized by severe nucleoid packing defects. Several derivatives exhibited additional effects on the Gram-positive cell wall synthesis machinery and/or the cytoplasmic membrane, which likely contributed to their increased antibacterial activity. While we could not identify specific cell wall or membrane targets, membrane depolarization was not observed. Our experiments further suggest that cell wall synthesis inhibition most likely occurs outside the membrane-bound lipid II cycle.

## Introduction

Increasing antibiotic resistance has created an urgent need for novel antibacterial agents and strategies. Both Gram-positive and Gram-negative bacteria, as well as mycobacteria, contribute to this problem.^1^ Bacterial strains that accumulate multiple resistances are of particular concern; *e.g.*, methicillin-resistant *Staphylococcus aureus* (MRSA) poses a constant threat of severe nosocomial infections. Several cases of pan-resistant Gram-negative bacteria have resulted in untreatable infections, most prominently *Pseudomonas aeruginosa*, *Klebsiella pneumoniae*, and *Acinetobacter baumannii*. Moreover, extensively and totally drug-resistant *Mycobacterium tuberculosis* strains plague low-income countries.^2−4^

Fluoroquinolones are an important class of broad-spectrum antibiotics that are effective against Gram-positive, Gram-negative, and mycobacteria and are orally available. Three fluoroquinolones, namely ciprofloxacin, moxifloxacin, and levofloxacin, are on the WHO’s list of essential medicines, the latter two for the treatment of tuberculosis (https://list.essentialmeds.org/). The success of fluoroquinolones is partly based on their dual mechanism of action on two essential bacterial enzymes,^5^ gyrase, and topoisomerase IV. Both proteins are involved in DNA packing: DNA gyrase, mainly in relieving DNA strand tension during replication and transcription, and topoisomerase IV, mainly in segregating replicated chromosomes. Inhibition of these enzymes leads to DNA aggregates, inhibits cell division, and ultimately leads to cell death.^6,7^ In Gram-negative and mycobacteria, the primary target of fluoroquinolones is topoisomerase IV, while gyrase is the secondary target. In Gram-positive bacteria, the opposite is the case.^8,9^

Fluoroquinolones are fully synthetic antibiotics and, as such, have undergone considerable derivatization approaches. Almost any residue apart from the quinolone core can be modified to alter the drug profile of the resulting compound, and structure–activity relationship studies have revealed the importance of individual side chains.^10^ Moreover, fluoroquinolones have been used to develop hybrid molecules that combine two pharmacophores in one structure.^11^

In this work, we aimed at synthesizing novel amino acid derivatives of norfloxacin, one of the oldest fluoroquinolones and the most important lead structure, with the aim of introducing new binding sites capable of interacting with additional targets in the bacterial cell. To this end, we were inspired by the enzymes NagA and LpxC, both of which contain catalytic metal cofactors in their active centers (Zn^2+^ and Cd^2+^). This is similar to DNA gyrase and topoisomerase IV, which both contain catalytic Mg^2+^ that interacts with norfloxacin. NagA is an *N*-acetylglucosamine-6-phosphate deacetylase, which catalyzes the deacetylation of *N*-acetylglucosamine-6-phosphate (GlcNAc6P) to glucosamine-6-phosphate (GlcN6P). It is involved in cell wall peptidoglycan turnover and has been proposed as a new drug target in *M. tuberculosis* since, in contrast to other bacteria, this protein is essential in this organism.^12^ LpxC is a UDP-3-O-(*R*3̅-hydroxymyristoyl)-*N*-acetylglucosamine deacetylase that catalyzes the second step of lipid A synthesis. Lipid A is the lipid anchor of lipopolysaccharides, which decorate the outer membrane of Gram-negative bacteria and confer its high impermeability.^13^ Its expression levels are tightly regulated, and both the deletion and overexpression of the *lpxC* gene are lethal.^14^

**Figure 1:**
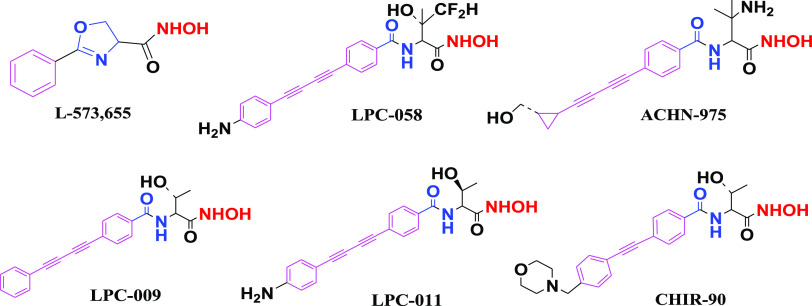
Selected LpxC inhibitors and shared pharmacophoric features: hydroxamate headgroup (red), central linker (blue), and lipophilic tail (purple).

In contrast to NagA, several inhibitors of LpxC have been reported.^15−17^ Most LpxC inhibitors share common features, namely, a hydroxamate headgroup, a central linker, and a lipophilic tail (Figure 1). From the structure of LpxC, it can be hypothesized that the hydroxamate headgroup binds the catalytic metal ion, while the lipophilic tail occupies a hydrophobic tunnel containing the myristate fatty acid side chain.^18^

Thus, we took known LpxC inhibitors as inspiration to introduce additional pharmacophores to norfloxacin, speculating that the addition of a metal-chelating group and a hydrophobic tail would afford additional binding sites capable of enhancing its interaction with gyrase and topoisomerase IV and/or interacting with other metalloenzymes and hydrophobic interaction partners. These modifications were introduced by using amino acid groups. Amino acids have at least two functional groups, the carboxylic acid group and the amino group, which can be easily coupled with a biologically active core such as a quinolone ring. Alterations of amino acids have been successfully explored for their antibacterial potential by coupling them to other compounds with biological activity, such as oxolinic, nalidixic, cinoxacin, and flumequine amino acid derivatives.^19^ Moreover, the bioisosteric replacement of the C-3 carboxylic acid moiety of fluoroquinolones, *e.g.*, by amidation, esterification, or conjugation with other compounds such as carbohydrates, increases antibacterial potency.^20^

**Figure 2:**
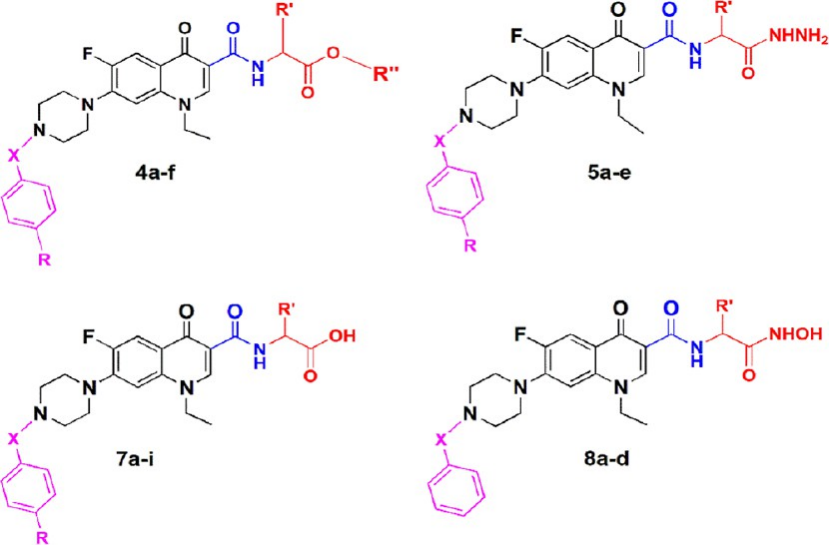
Designed compound series: amino acid esters and hydrazides of *N*-acyl, sulfonyl, and alkylpiperazinyl derivatives of norfloxacin (**4a**–**f**, **5a**–**e**) and amino acids and hydroxamic acids of different *N*-acylpiperazinyl derivatives of norfloxacin (**7e**–**i** and **8a**–**d**). The hydroxamate headgroup is shown in red, the central linker in blue, and the lipophilic tail in purple.

Thus, we kept the quinolone ring of the norfloxacin lead structure and introduced modifications at two sites: metal-chelating groups at the carboxylic acid group and lipophilic tails at the *N*-piperazinyl ring. This yielded four series of compounds in two groups (Figure 2): amino acid ester and hydrazide derivatives of *N*-acyl, sulfonyl, and alkylpiperazinyl derivatives of norfloxacin (**4a–f, 5a–e**) and amino acid and hydroxamic acid derivatives of different *N*-acylpiperazinyl derivatives of norfloxacin (**7a–i, 8a–d**). These new derivatives were then characterized with respect to their antibacterial activity, cytotoxicity, and mechanism of action by using *in silico*, *in vitro*, and *in vivo* assays.

## Results and Discussion

### Chemistry

**Scheme 1:**
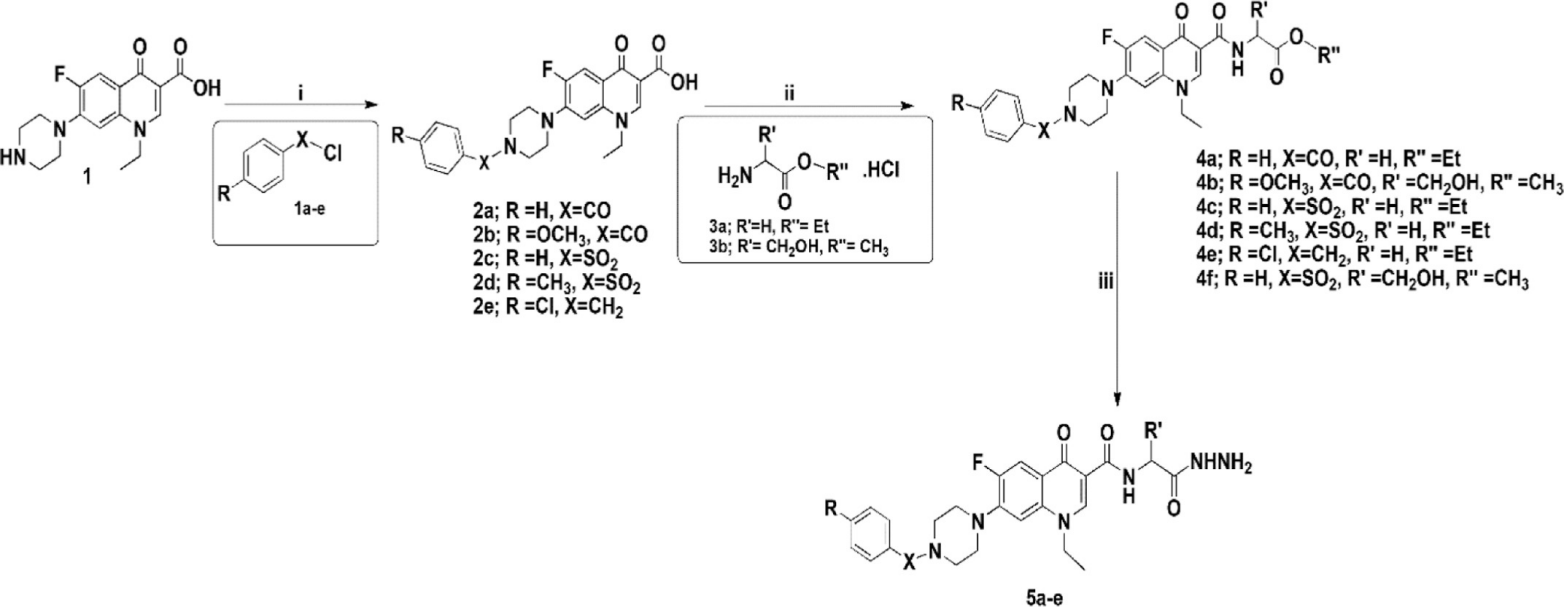
Reagents and Conditions: (i) THF, Et_3_N, Reflux; (ii) DCM, Et_3_N, ClCOOEt. rt; and (iii) MeOH, Hydrazine Hydrate, Reflux

**Scheme 2:**
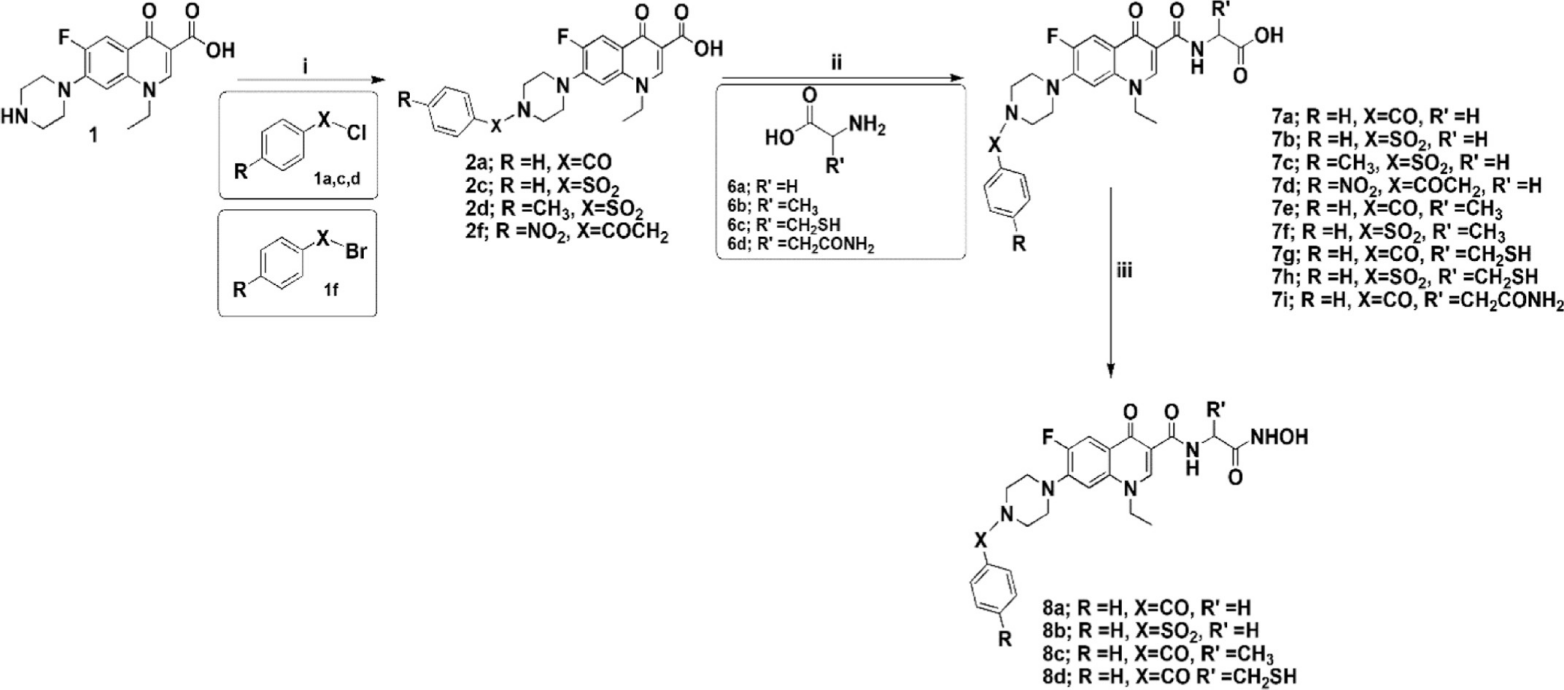
Reagents and Conditions: (i) THF, Et_3_N, Reflux; (ii) DCM, Et_3_N, ClCOOEt. rt; and (iii) DCM, Et_3_N, ClCOOEt. rt

A series of amino acid esters of *N*-acyl, sulfonyl, and alkyl derivatives of norfloxacin were synthesized, as outlined in Schemes 1 and 2. Known acyl, sulfonyl, alkyl, and phenacyl derivatives **2a**–**f** were synthesized according to published procedures.^21,22^ Subsequently, amino acid ester derivatives **4a**–**f** (Scheme 1, final yield 49–72%) were synthesized by the reaction of N-substituted piperazinyl norfloxacin derivatives with ethyl chloroformate in the presence of triethylamine in dichloromethane to afford mixed anhydrides, which then interacted with added amino acid ester hydrochloride. Finally, hydrazide derivatives **5a**–**e** were prepared by the reaction of *N*-acyl norfloxacin amino acid esters with hydrazine hydrate in methanol (final yield 53–70%). The final products were purified by crystallization using ethanol and washed with diethyl ether.

IR spectra of ester compounds **4a**–**f** showed absorption bands at 3450, 1748, 1655, and 1629 cm^–1^ attributed to NH, ester C=O, carbamide C=O, and quinolone C=O, respectively. The ^1^HNMR spectra of all ester compounds were characterized by the disappearance of the broad singlet signal of the COOH groups of the intermediate compounds **2a**–**e** and the presence of a triplet or doublet (in the case of serine amino acid ester) signal at δ 10.5–10.24 ppm assigned to amidic CONH. In addition, glycine ethyl ester compounds **4a, 4c–e** showed triplet signals at δ 1.41–1.26 ppm assigned to CH_3_CH_2_OOC and quartet signals at δ 4.15–4.12 ppm assigned to CH_3_CH_2_OOC, which overlapped with the doublet signal assigned to NHCH_2_CO. For the serine methyl ester compounds **4b** and **4f**, we observed singlet signals at δ 3.78–3.10 ppm assigned to COOCH_3_ and a triplet peak at δ 5.24 assigned to CH_2_OH. Other signals, including aromatic protons, appeared at their expected chemical shift in agreement with the reported data.^21,22^ In the case of the hydrazide compounds **5a**–**e**, IR spectra showed absorption bands at 3315, 1693, 1669, and 1610 cm^–1^ assigned to NH, hydrazide C=O, carbamidic C=O, and quinolone C=O groups, respectively. In addition to the expected aromatic and characteristic protons of their parent compounds **4a**–**e**, the ^1^HNMR spectra of the hydrazides **5a**–**e** were also characterized by the disappearance of ester group signals, the presence of triplet or doublet (in the case of serine amino acid) signals at δ 10.32–10.17 ppm assigned to amidic CONH, broad singlet signals at δ 9.19–9.13 ppm assigned to NH_2_NH of hydrazide, and a singlet signal at δ 4.23 ppm assigned to NHNH_2_ of hydrazide.

The second series of amino acid and hydroxamic acid derivatives of norfloxacin were prepared, as depicted in Scheme 2. First, free amino acid derivatives **7a**–**i** were prepared by the reaction of *N*-acyl norfloxacin derivatives **2a**, **c**, **d**, and **f** with ethyl chloroformate in dichloromethane in the presence of triethylamine to give mixed anhydrides. Then, two equivalents of amino acids (glycine, l-alanine, l-cysteine, and l-asparagine) were added (final yield: 45–63%). Acid compounds were purified by column chromatography using 9.7:0.3 dichloromethane/methanol as the mobile phase. To prepare hydroxamic acid derivatives **8a**–**d**, *N*-acyl norfloxacin amino acid derivatives **7a**, **7b**, **7e**, and **7g** were treated with ethyl chloroformate in dichloromethane in the presence of triethylamine to produce mixed anhydrides. Then, hydroxylamine hydrochloride was added to obtain the amino acid hydroxamic derivatives of **8a**–**d**. Compounds were purified by column chromatography using 9.5:0.5 dichloromethane/methanol as the mobile phase (final yield: 29–68%). Chemical structures of the newly prepared compounds were assessed by ^1^H NMR, ^13^C NMR, mass spectrometry, and elemental analysis (Figures S1–S6, Table S1).

IR spectra of the free amino acid derivatives **7a**–**i** showed absorption bands at 3435–3420, 3400–2500, 1710–1725, 1655–1650, and 1640–1625 cm^–1^ attributed to NH, OH, acidic C=O, amidic C=O, and quinolone C=O, respectively. In addition to anticipated aromatic protons, ^1^HNMR spectra of **7a**–**i** also showed triplet (in the case of glycine amino acid) or doublet (in the case of alanine, cysteine, and asparagine amino acids) signals at δ 10.44–10.16 ppm assigned to amidic CONH and broad singlet signals at δ 12.55–10.35 ppm assigned to the COOH group. Compounds **7a**–**d** showed doublet signals at δ 4.50–4.04 ppm assigned to the NHCH_2_CO group, which appeared as δ 4.46 ppm multiplet for compounds **7e**–**f**, which also showed a doublet peak at δ 1.36–1.26 ppm assigned to the COCHCH_3_ group. Compounds **7g**–**h** gave multiplet signals at δ 4.10–4.70 ppm assigned to the CHCH_2_SH group, triplet peaks at δ 3.75–3.33 ppm assigned to the CHCH_2_SH group, and singlet signals at δ 3.75–3.44 ppm attributed to the CHCH_2_SH group. The ^1^HNMR spectrum of compound **7i** showed a singlet signal at δ 10.36 ppm assigned to the CH_2_CONH_2_ group, a multiplet signal at δ 3.86 ppm assigned to the CHCH_2_CO group, and a doublet peak at δ 1.41 ppm attributed to the CHCH_2_CO group.

IR spectra of the hydroxamic acid derivatives **8a**–**d** showed absorption bands at 3420, 3230, 1670, 1650, and 1625 cm^–1^ attributed to NH, OH, hydroxamic CO, amidic CO, and quinolone C=O, respectively. In addition to the expected aromatic and characteristic protons of the corresponding amino acid derivatives **7a, 7b, 7e, and 7g**, the ^1^HNMR spectra of their hydroxamic derivatives were characterized by the disappearance of the broad singlet peak of COOH and the presence of broad singlet signals at δ 9.12–7.58 ppm assigned to the NH of hydroxamic acid.

### Antibacterial Activity

Antibacterial activity of norfloxacin derivatives **2**–**8** was evaluated against *Escherichia coli* W3110, *P. aeruginosa* PAO1, *K. pneumoniae* ATCC 10031 (Gram-negative wild type strains), *S. aureus* CCUG1800T, and *Enterococcus faecalis* ATCC 19433 (Gram-positive wild type strains), and *M. tuberculosis* MC26020 (live attenuated strain for use in BSL-II laboratories^23^). Additionally, all compounds were tested against a norfloxacin-resistant clinical isolate of *E. coli* and an MRSA strain resistant to both norfloxacin and ciprofloxacin.^24^ Several of the new derivatives showed equal or enhanced activity when compared to norfloxacin and ciprofloxacin (Gram-negative and Gram-positive strains) or isoniazid (*M. tuberculosis*) (Table 1, indicated in bold). While EUCAST breakpoints were not reached (https://www.eucast.org/clinical_breakpoints), it should be noted that derivatives **2e**, **4d**, and **8a** were 2.8–3.5 times more active than norfloxacin against the tested norfloxacin-resistant clinical isolate of *E. coli*, suggesting that they are good lead structures for further improvement.

**Table 1:** Minimal Inhibitory Concentrations (MICs) in μM^a^

compound	E. coli W3110	E. coli^b^	P. aeruginosa PAO1	K. pneumoniae ATCC 10031	S. aureus CCUG1800T	S. aureus^b^ (ATCC 43300)	E. faecalis ATCC 19433	M. tuberculosis MC26020
INH								1.82
Nor	0.39	50.10	6.26	9.39	3.13	100.21	4.69	1.56
Cip	0.37	193.17	3.01	7.54	3.01	96.58	6.03	2.26
**2a**	>1209.14	>1209.14	>1209.14	1209.14	75.57	604.57	113.35	>1209.14
**2b**	>1129.09	282.27	>1129.09	**5.51**	70.56	**17.64**	70.56	>1129.09
**2c**	>1114.27	>1114.27	>1114.27	1114.27	69.64	1114.27	1114.27	>1114.27
**2d**	>1081.26	1081.26	>1081.26	>1081.26	67.57	>1081.26	>1081.26	>1081.26
**2e**	72.08	**18.02**	>1153.41	>1153.41	36.04	576.70	288.35	>1153.41
**2f**	5.18	>1061.22	1061.22	>1061.22	16.58	**66.32**	33.16	>1061.22
**4a**	>1006.80	>1006.80	>1006.80	>1006.80	1006.80	>1006.80	62.92	>1006.80
**4b**	>923.23	923.23	>923.23	>923.23	923.23	923.23	86.55	>923.23
**4c**	>940.13	>940.13	>940.13	>940.13	>940.13	940.13	>940.13	235.03
**4d**	4.47	**14.32**	>916.54	**2.68**	114.56	>916.54	>916.54	>916.54
**4e**	>967.86	241.96	>967.86	>967.86	>967.86	483.93	967.86	>967.86
**4f**	2.67	57.08	>913.32	**5.35**	**1.78**	>913.32	**3.56**	114.16
**5a**	>1035.34	>1035.34	>1035.34	258.83	>1035.34	>1035.34	>1035.34	1035.34
**5b**	7.21	>923.23	>923.23	57.70	57.70	**14.42**	115.40	7.21
**5c**	>964.99	241.24	>964.99	>964.99	>964.99	>964.99	>964.99	482.49
**5d**	>940.13	>940.13	>940.13	29.37	>940.13	256.00	**4.59**	>940.13
**5e**	>994.21	994.21	994.21	248.55	>994.21	497.10	>994.21	>994.21
**7a**	133.19	133.19	1065.57	66.59	33.29	>1065.57	>1065.57	4.16
**7b**	61.95	991.21	991.21	>991.21	7.74	>991.21	15.48	>991.21
**7c**	120.62	>964.99	>964.99	964.99	**3.76**	**30.15**	**3.76**	>964.99
**7d**	14.82	**29.65**	711.75	237.25	59.31	237.25	>949.00	355.87
**7e**	158.84	129.42	>1035.36	>1035.36	16.17	>1035.36	>1035.36	194.13
**7f**	241.24	>964.99	>964.99	120.62	**1.88**	>964.99	5.65	241.24
**7g**	15.19	972.31	972.31	60.76	7.59	**15.19**	**1.89**	**1.42**
**7h**	113.75	>910.01	>910.01	>910.01	14.21	227.50	>910.01	>910.01
**7i**	7.44	**29.76**	952.48	**4.65**	14.88	>952.48	>952.48	1.86
**8a**	129.16	**16.14**	1033.29	**6.05**	8.07	1033.29	1033.29	161.45
**8b**	240.80	>963.20	963.20	>963.20	60.20	>963.20	60.20	90.30
**8c**	7.85	>1004.84	1004.84	62.80	94.20	**62.80**	**2.94**	**1.47**
**8d**	>945.36	236.34	>945.36	945.36	472.68	>945.36	>945.36	>945.36

a MICs better than those of norfloxacin (isoniazid for *M. tuberculosis*) are indicated in **bold**.

b Fluoroquinolone-resistant strains.

Given the structural similarity of some derivatives, it was surprising that their activities differed considerably. For example, compounds **8a** and **8c** were more potent than norfloxacin against a number of strains, yet **8b** and **8d** showed poor or no activity. The latter two both carry sulfur (X = phenylsulfonyl and R′ = mercaptomethyl, respectively), while **8a** and **8c** carry carboxy and methyl/hydrogen in these positions, suggesting that hydrophobicity could play a role in the difference in activity. Interestingly, **8a** is more active against Gram-negative bacteria, while **8c** shows better activity against Gram-positive bacteria and mycobacteria. The structural difference between these compounds is minute, defined only by a methyl group or hydrogen as R′. It could be speculated that the increased hydrophobicity conferred by the methyl group may facilitate uptake into Gram-negative cells.

### *In silico* Prediction of Drug Likeness and Cytotoxicity

The compliance of the newly synthesized compounds to Lipinski’s and Veber’s rules was estimated *in silico*. Lipinski’s rule of five states that a compound with a molecular weight under 500 Da, a coefficient of partition between octanol and water lower than 5, no more than five hydrogen bond donors, and no more than 10 hydrogen bond acceptors is likely to be a good drug candidate. Veber’s rule states that a compound that has 10 or fewer rotatable bonds and a polar surface area no greater than 140 Å^2^ is likely to exhibit good oral bioavailability.^25^ Except for compounds **4b**, **5b**–**d**, **7d**, **7i**, and **8b**, all tested derivatives complied with both Lipinski’s and Veber’s rules (Table S2).

**Figure 3:**
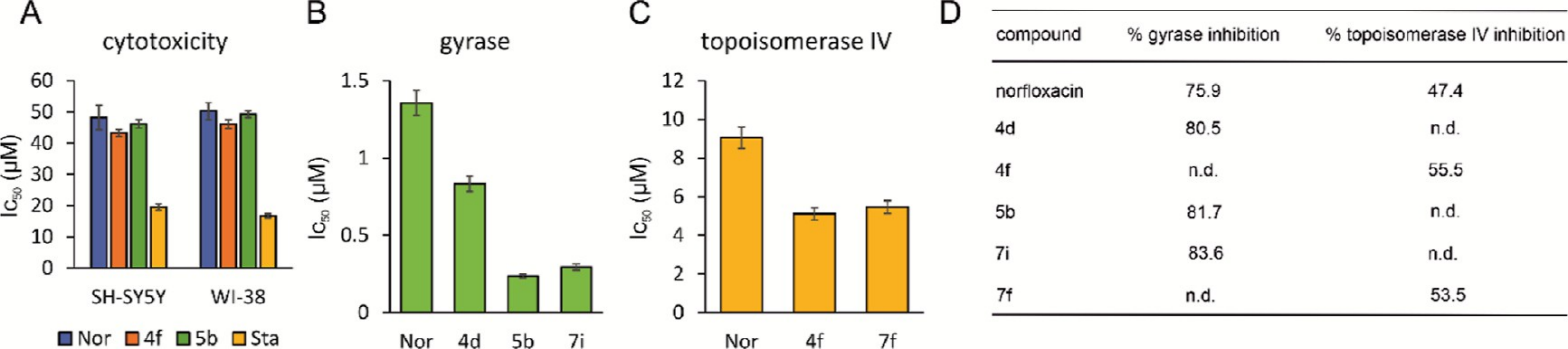
Cytotoxicity and *in vitro* enzyme inhibition. (A) IC_50_ values of compounds against human neuroblastoma (SH-SY5Y) and human fetal lung fibroblast (WI-38) cells. Staurosporine (Sta) served as a positive control. (B) IC_50_ values of compounds against purified *E. coli* gyrase. (C) IC_50_ values of compounds against purified *E. coli* topoisomerase IV. (D) Percent inhibition of purified *E. coli* gyrase and topoisomerase IV at 10 μM of the respective compounds. Nor: norfloxacin.

Further pharmacokinetic and toxicity predictions are shown in Tables S3–S4 and discussed in Text S1–S3. In essence, most newly synthesized derivatives showed properties similar to or better than norfloxacin. Following favorable toxicity predictions, we experimentally evaluated cytotoxic effects on the human neuroblastoma SH-SY5Y and human fetal lung fibroblast (WI-38) cell lines for exemplary compounds **4f** and **5b** (selected based on antibacterial and *in vitro* enzyme inhibition activities). Compounds **4f** and **5b** exhibited similar IC_50_ values as norfloxacin and were clearly less toxic than the apoptosis-inducing kinase inhibitor staurosporine (Figure 3A). This leaves greater therapeutic windows than norfloxacin for **4f** in the case of *K. pneumoniae*, *S. aureus*, and *E. faecalis* and for **5b** in the case of MRSA (Figure S57). While norfloxacin has a much greater therapeutic window for fluoroquinolone-sensitive *E. coli*, **5f** and **5b** still have considerable windows against the same strain.

### Molecular Docking

**Figure 4:**
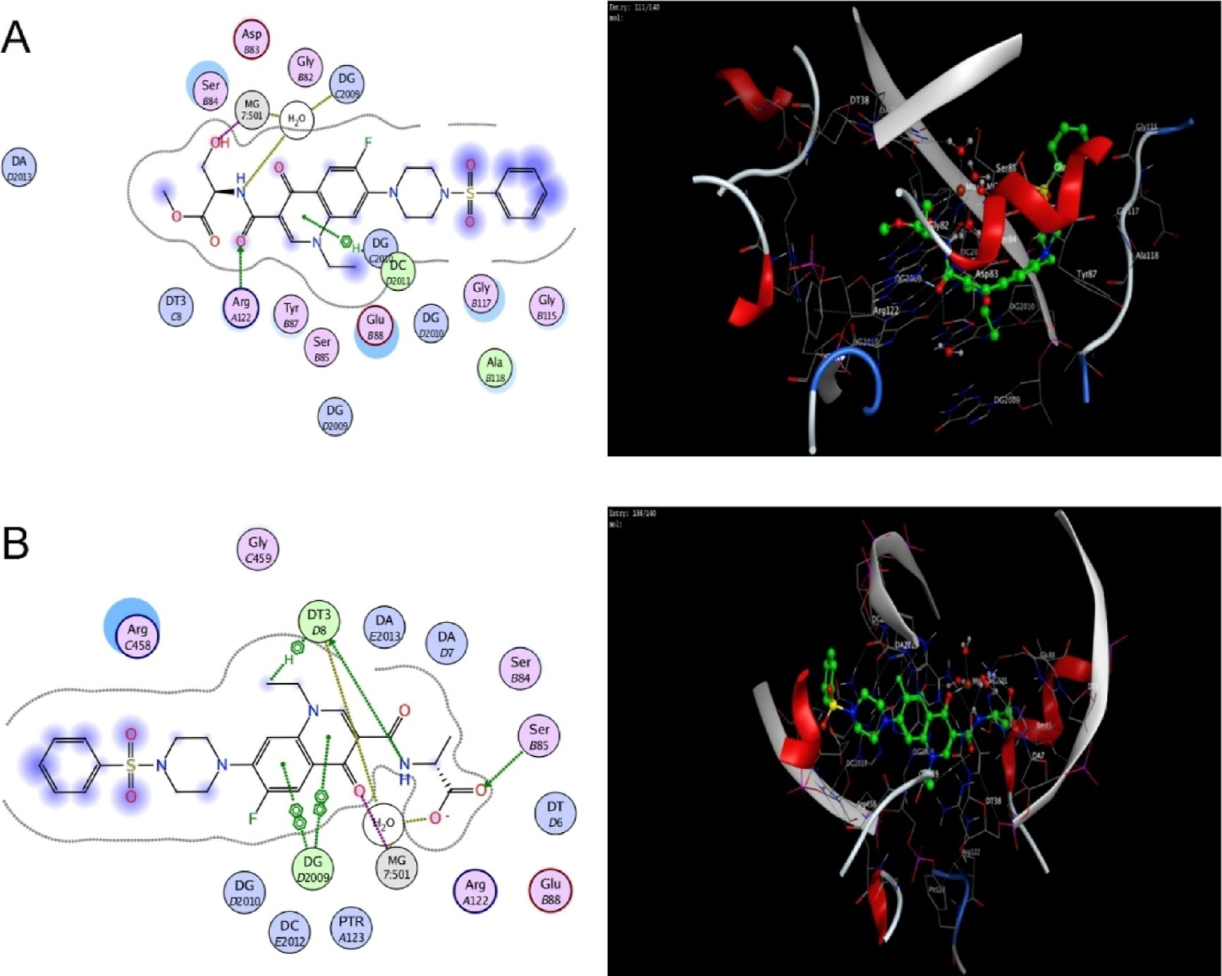
2D and 3D interactions of compounds **4f** (A) and **7f** (B) with *S. aureus* DNA gyrase. For color coding, see Figure S98.

**Figure 5:**
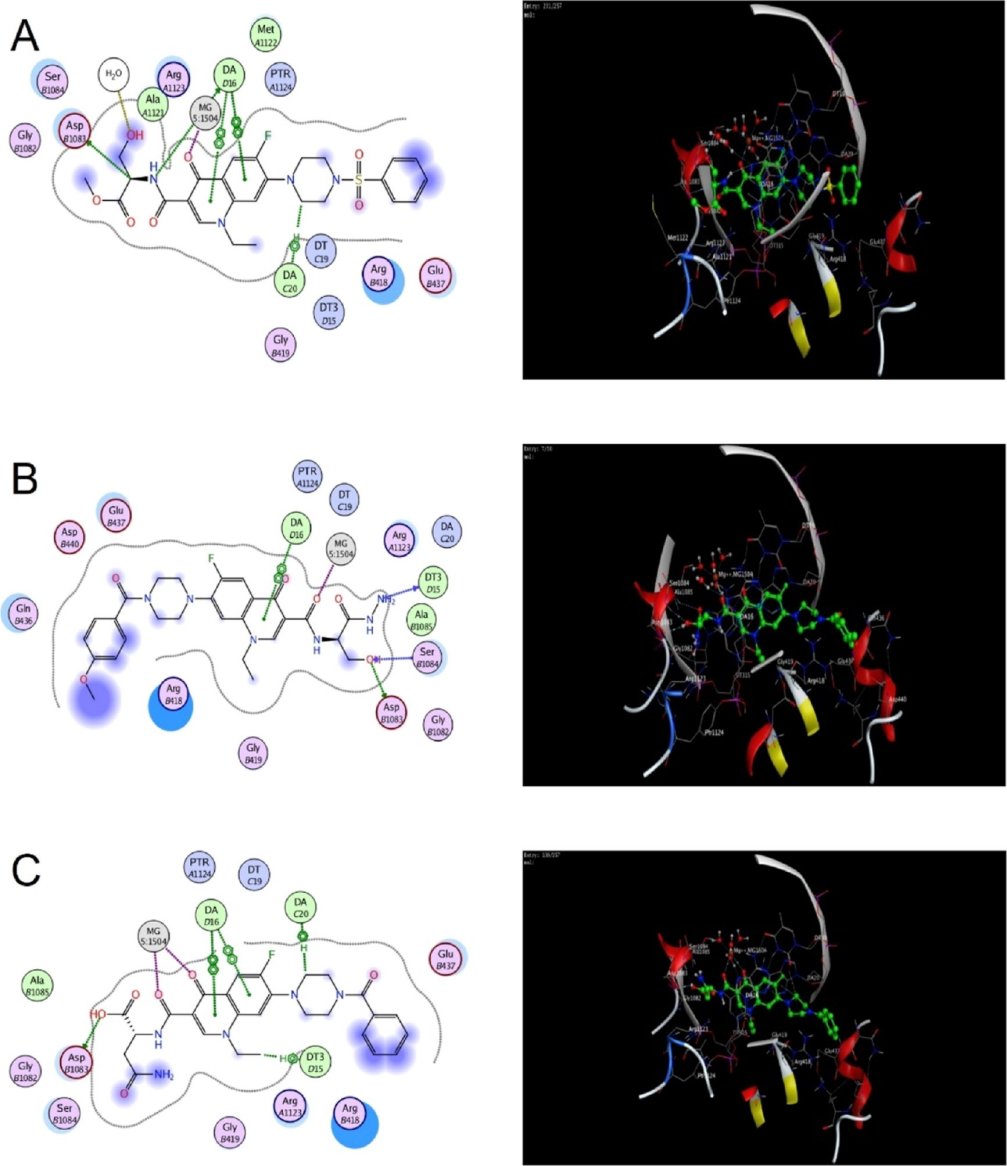
2D and 3D interactions of compound **4f** (A), **5b** (B), and **7i** (C) with *A. baumannii* topoisomerase IV. For color coding, see Figure S98.

To assess the interaction of the newly synthesized derivatives with their target enzymes, molecular docking studies were performed. Test compounds were selected based on their antibacterial activity and docked to *S. aureus* DNA gyrase (Figures 4, S58, S59) and *A. baumannii* topoisomerase IV (Figures 5, S60, S61). Docking studies were performed on the crystal structure of *S. aureus* DNA gyrase, the primary target of fluoroquinolones in Gram-positive bacteria, in complex with moxifloxacin and DNA (PDB 5cdq).^26^ The docking protocol was validated by redocking of the cocrystallized moxifloxacin ligand (Figure S58, redocking rmsd = 0.6010 Å, binding score = −10.76 kcal mol^–1^), and the validated docking setup was then used to investigate the interactions of norfloxacin (Figure S59, binding score = −9.54 kcal mol^–1^). The main interactions of norfloxacin were two coordination bonds with Mg^2+^ (2.44 and 2.34 Å) and H-bonding between the carbonyl of the carboxylic acid group and Ser B84 (2.26 Å), as well as a π–hydrogen bond with deoxyadenosine (DA) E2013 and π–π stacking between the quinolone ring and deoxyguanosine (DG) D2009.

Compounds **4f** and **7f** were selected for docking studies on gyrase (Figure 4), showing binding scores of −10.23 and −11.93 kcal mol^–1^, respectively. Similarly, to norfloxacin, they formed coordination bonds with Mg^2+^ through the oxygen of their carboxylic carbonyl and hydroxyl groups, respectively (2.40 and 2.42 Å). H-bonding with Ser B84 was mediated by the hydroxyl group of **4f** and the oxygen of the carboxylic carbonyl group of **7f** (1.83 and 2.52 Å). Interactions with the nitrogenous bases DG D2009 and DA E2013 were mediated by π–π stacking and π–H bonding with the quinolone and piperazine rings, respectively. Additionally, H-bonds were formed between Arg A122 and the amidic carbonyl group of **4f** and the oxygen of the carboxylate of **7f** (1.64 and 2.34 Å).

Further interactions were mediated by the structural moieties that were added to *N*4 of the piperazine ring and the carboxylic acid group of norfloxacin. This included H-bonding and π-cation bonds, such as H-bonds between Lys C417 and the amidic carbonyl group of **4f** and the sulfonyl group of the phenylsulfonyl moiety of **7f** (2.27 Å), a π–H bond between DC C2010 and the phenyl ring of the phenylsulfonyl moiety of **7f**, a H-bond with Arg C458 with the carboxylate group of **7f**, and a H-bond with DG C2009 through the hydroxyl group of the serine moiety of **4f**. These additional interactions might stabilize the binding of compounds **4f** and **7f** to gyrase, possibly explaining their higher antibacterial activity against Gram-positive bacteria.

Similar results were obtained for DNA topoisomerase IV, which is the primary target of norfloxacin in Gram-negative bacteria. Docking studies were performed on the crystal structure of *A. baumannii* topoisomerase IV in complex with moxifloxacin and DNA (PDB 2xkk).^27^ Redocking of moxifloxacin gave a redocking rmsd of 0.3718 Å and a binding score of −10.62 kcal mol^–1^ (Figure S60). Docking of norfloxacin gave a binding score of −9.32 kcal mol^–1^ and revealed the main interactions to be a coordination bond with Mg^2+^ (2.41 Å), H-bonding between the carbonyl of the carboxylic acid group and Arg A1123 (3.45 Å), a π–hydrogen bond with DA C20, and π–π stacking of A D16, both with the quinolone ring (Figure S61).

Compounds **4d**, **4f**, **5b**, **7i**, and **8c** were selected for docking on topoisomerase IV (Figures 5, S62–S64), of which **4f**, **5b**, and **7i** showed the lowest binding scores (−10.94, −11.43, and −11.58 kcal mol^–1^, respectively). Coordination bonds with Mg^2+^ were formed with the quinolone carbonyl group of **4f** (2.43 Å), the amidic carbonyl group of **5b** (2.24 Å), and both the quinolone and amidic carbonyl groups of **7i** (2.54 and 2.36 Å). H-bonds with Arg A1123 were formed with the amidic carbonyl groups of **5b** and **7i** (average length of 1.9 Å) and the ester carbonyl group of **4f** (2.46 Å). All investigated compounds formed a π–hydrogen bond with DA C20 and π–π stacking with DA D16 with their quinolone and piperazine rings.

In addition to these interactions, the added moieties of the newly synthesized compounds formed H- and π-cation bonds, such as H-bonds with Ser B1084 mediated by the ester carbonyl group of **4f** and the amidic carbonyl group of **5b** and **7i** (average length 2.15 Å) and π-cation bonds between Arg B418 with the quinolone ring of **5b** and the phenyl ring of the benzoyl moiety of **7i**. Instead, compound **4f** formed a hydrogen bond with Arg B418 through the sulfonyl group of its phenylsulfonyl moiety. Additionally, **5b** and **7i** formed H-bonds with Asp B1083 through the NH_2_ of the hydrazide and carboxylate groups, respectively (2.27 and 1.98 Å). These additional interactions might stabilize the binding of compounds **4f**, **5b**, and **7i** to topoisomerase IV, possibly explaining their higher antibacterial activity against Gram-negative bacteria.

To assess possible interactions with additional target enzymes, docking experiments were also performed with *Mycobacterium smegmatis* NagA and *P. aeruginosa* LpxC (Figures S65–S86). To further assess the ability of the new derivatives to interact with LpxC, ligand-based pharmacophore modeling was performed (Figures S87–S89, Tables S5, S6). Details on these experiments are described in Text S4–S6.

### *In Vitro* Inhibition of *E. coli* Gyrase and Topoisomerase IV

To confirm that the new compounds interact with gyrase and topoisomerase IV, *in vitro* inhibition studies were performed on the purified *E. coli* enzymes (Inspiralis).^28^ Compounds **4d**, **5b**, and **7i** were tested against gyrase and **4f** and **7f** against topoisomerase IV (Figure 3B–D). All compounds showed lower IC_50_ values than norfloxacin in these assays. Notably, **5b** and **7i** were 5.7 and 4.6 times more active than norfloxacin against DNA gyrase, respectively.

Since coordination bonds with Mg^2+^ in the active centers of gyrase and topoisomerase IV were crucial for the interaction of norfloxacin and its derivatives with these enzymes, their ability to bind metal ions was assessed. UV–vis spectroscopy was used to study the chelation of Mg^2+^, Zn^2+^, and Cd^2+^, the latter two being present in the active centers of the possible additional target enzymes NagA and LpxC. All tested compounds (**5b**, **7b**, **7i**, **8a**, and **8c**) showed a spectral shift indicative of metal complexation at a 1:1 ratio (Table S6, Figures S90, S91). Thereby, affinity for zinc was higher than that for magnesium and cadmium, and the derivatives showed higher binding of metal ions than norfloxacin. Further, hydroxamic acid and hydrazide derivatives showed higher metal chelation than carboxylic compounds, aligning well with the molecular docking results.

### Bacterial Cytological Profiling of *E. coli*

**Figure 6:**
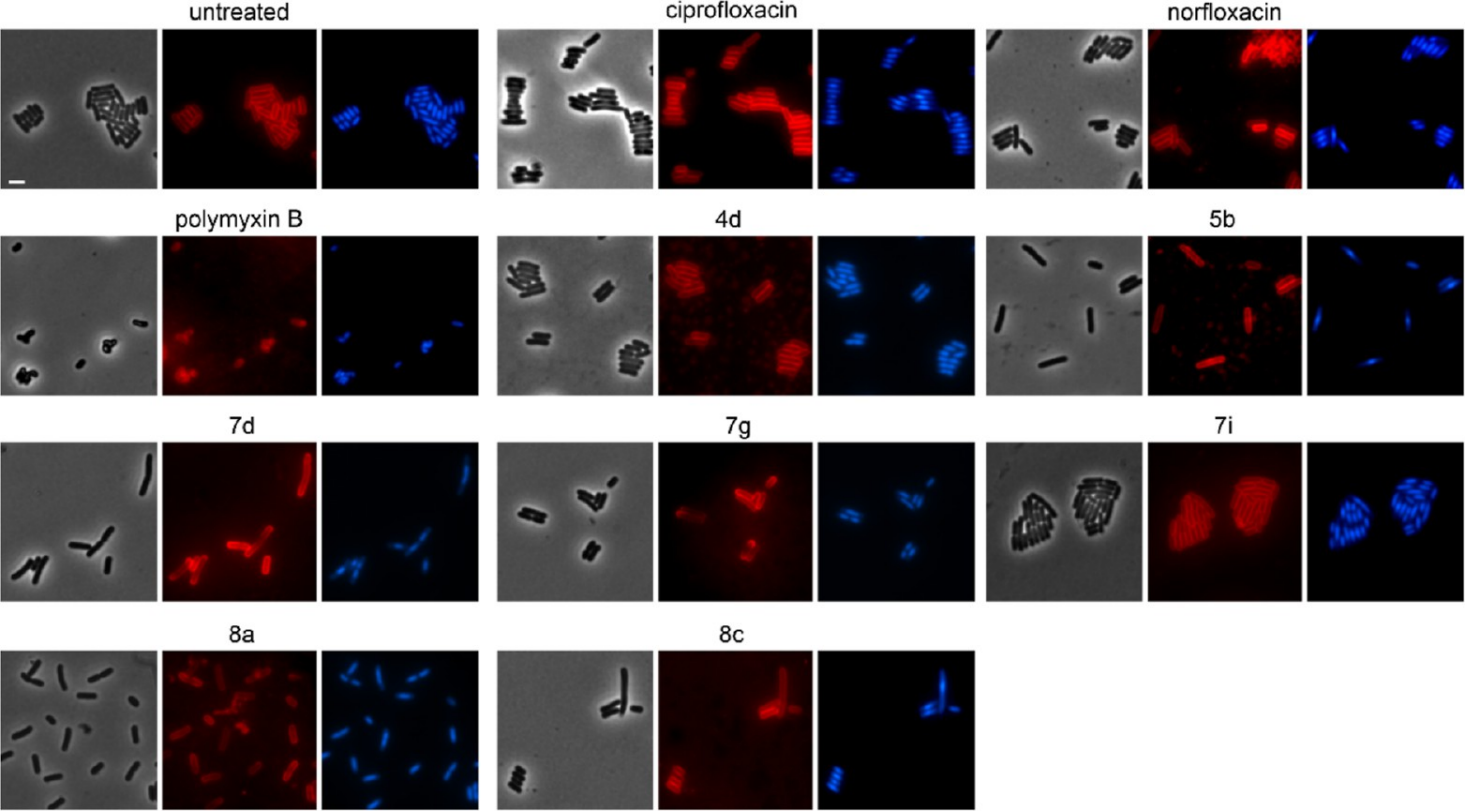
Bacterial cytological profiling of *E. coli* W3110. Cells were treated with 1xMIC of the respective compounds for 10 min (polymyxin B) or 1 h (all other compounds) prior to staining with FM4-64 (membrane, red) and DAPI (nucleoid, blue). Scale bar = 2 μm.

To confirm that the new compounds inhibit DNA gyrase *in vivo*, we used bacterial cytological profiling (BCP). BCP is a phenotypic analysis method based on phase contrast microscopy combined with fluorescent staining of the cell membrane and DNA that can be employed to elucidate antibiotic targets.^29^ Gyrase/topoisomerase IV inhibition by fluoroquinolones results in characteristic and unique nucleoid packing defects that are not observed with other antibiotics.^30^ Compounds **4d**, **5b**, **7d**, **7g**, **7i**, **8a**, and **8c** were subjected to BCP in Gram-negative *E. coli* (Figure 6, Table S8). Gyrase inhibition could be confirmed for **5b**, **7d**, **7i**, and **8c**, but not for **4d**, **7g**, and **8a**. Since **4d** was active against gyrase *in vitro*, it may interact with a different target *in vivo*.

No membrane aberrations were observed in the FM4-64 membrane stain, yet this dye does not discriminate between inner and outer membrane effects. To confirm that there are no additional effects on the cytoplasmic membrane, a green-fluorescent protein (GFP) fusion to the ubiquitous membrane protein GlpT was used as a proxy for the inner membrane (strain BCB472^31^), and indeed no effects were observed (Figure S92).

### Effects on the Outer Membrane and LpxC

Since molecular docking revealed a possible interaction of the new compounds with LpxC, outer membrane integrity was studied by assessing synergy with mupirocin, a translation inhibitor that is able to inhibit *E. coli* isoleucine tRNA synthase but cannot pass its outer membrane.^34^ If LpxC is indeed inhibited, the outer membrane will be weakened, resulting in increased uptake of mupirocin and consequent synergy, as observed with the LpxC inhibitor ACHN-975. However, synergy assays revealed only additive effects of the tested norfloxacin derivatives (Table S9), suggesting that they do not permeabilize the outer membrane.

To confirm these results and rule out an inhibition of LpxC that would not manifest in increased outer membrane permeability, we additionally employed a newly developed *in vivo* screening assay for LpxC inhibition. To this end, MICs were determined against a strain overexpressing the *lpxC* gene from the arabinose-inducible *P*_*BAD*_ promoter.^35^ If LpxC is a target of the tested compound and its inhibition contributes to its antibacterial activity, the MIC should increase with rising arabinose concentrations due to the presence of more target molecules. This was indeed the case for the positive control ACHN-975, but not for other control antibiotics (polymyxin B and nitrofurantoin), demonstrating that the assay works and is specific (Figure S93). However, no arabinose-dependent MIC increase was observed for any of the test compounds, confirming that they do not inhibit LpxC.

### Bacterial Cytological Profiling of *B. subtilis*

To validate topoisomerase inhibition as a mechanism of the compounds in Gram-positive bacteria, BCP was performed in the model organism *Bacillus subtilis* (see Table S10 for MICs). Gyrase inhibition could be confirmed for compounds **5b**, **7a**, **7b**, **7c**, **7d**, **7e**, **7f**, **7g**, **7h**, and **7i**, but not **8a**. Compound **4a** showed slight DNA packing defects, indicating a possible gyrase inhibition, yet no clear phenotype could be observed even at higher concentrations (Figure S94, Table S11). Compounds **5b**, **7b**, **7c**, **7d**, **7e**, **7h**, and **8a** showed aberrations in the membrane stain, suggesting additional membrane effects. However, similar phenotypes were observed with norfloxacin and ciprofloxacin as well, indicating that membrane activity may be a shared feature of fluoroquinolones. Membrane aberrations are most commonly caused by depolarization.^32^ To assess whether the observed membrane aberrations are indicative of dissipation of the membrane potential, the membrane potentiometric probe DiSC(3)5 was employed. Yet, no effects were observed (Figure S95), suggesting that membrane defects may rather be caused by phase separation or inhibition of cell wall synthesis.^33^

### Effects on Peptidoglycan Synthesis

Since molecular docking revealed a possible effect on NagA, which is involved in cell wall turnover, and membrane aberrations observed in the *B. subtilis* BCP could point to possible cell wall defects, we tested the effects of the new compounds on *B. subtilis* cell wall synthesis using different phenotypic assays. Table 2 shows an overview of the results obtained in all of these assays.

**Table 2:** Summary of Cell Wall Synthesis Experiments in *B. subtilis*^a^

compound	concentration (μM)	PG integrity	MreB mobility	MurG localization	MraY localization	PbpB localization	PonA localization	PG synthesis inhibition
untreated		intact	mobile	spotty	rough	smooth	septal	no
Cip	**3.01**	intact	mobile, static clusters	smooth	rough	smooth	septal	no
Nor	**18.11**	intact	mobile, static clusters	smooth/spotty	rough	smooth	patchy	no
Van	**0.68**	compromised	static	patchy/dispersed	rough	smooth	septal	yes
**5b**	**28.85**	intact	static clusters	smooth/patchy	rough	smooth	septal	yes
**7a**	**1.56**	intact	static clusters	n.d	n.d	n.d	n.d	yes
**7b**	**1.45**	intact	static clusters	smooth	n.d	n.d	n.d	yes
**7c**	**1.88**	compromised	static clusters	smooth	n.d	n.d	n.d	yes
**7d**	**51.89**	compromised	mobile	patchy/dispersed	rough	smooth	septal	no^b^
**7e**	**4.04**	intact	mobile	smooth	n.d	n.d	n.d	no
**7f**	**3.76**	compromised	mobile	n.d	n.d	n.d	n.d	no^b^
**7g**	**1.90**	intact	mobile	n.d	n.d	n.d	n.d	no
**7h**	**1.77**	intact	mobile	smooth/patchy	rough	smooth	septal	no
**7i**	**5.58**	intact	static clusters	smooth	n.d	n.d	n.d	yes
**8a**	**8.07**	compromised	mobile	smooth	n.d	n.d	n.d	no^b^

a PG: peptidoglycan, Cip: ciprofloxacin, Nor: norfloxacin, Van: vancomycin, D-cyc: D-cycloserine, Fos: fosfomycin, and Tun: tunicamycin.

b Possible indirect effects on peptidoglycan synthesis.

**Figure 7:**
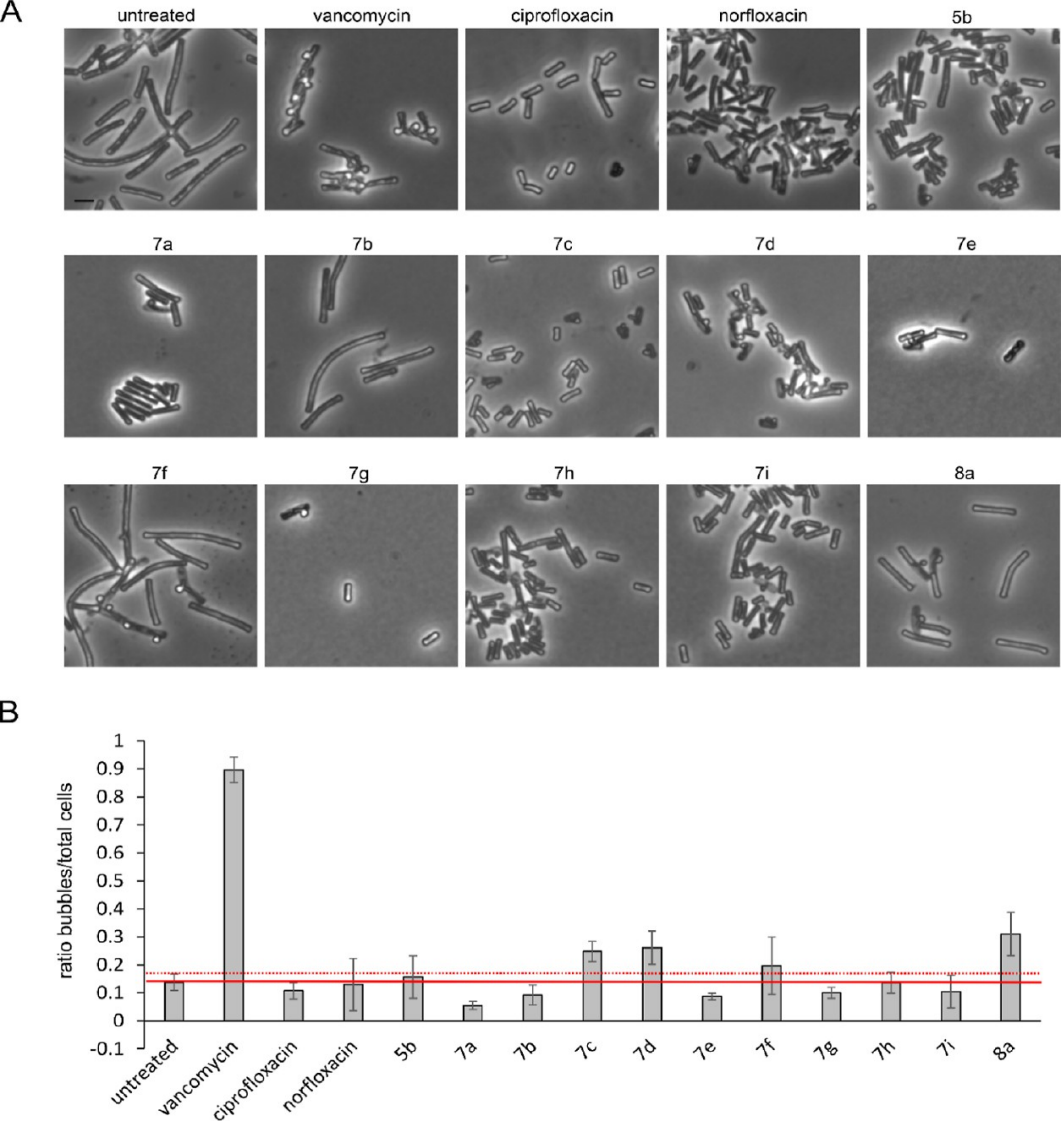
Effects on peptidoglycan synthesis. (A) Phase contrast microscopy of *B. subtilis* DSM402. Cells were treated with 1xMIC of the respective compounds for 10 min (fosfomycin, tunicamycin, and vancomycin) or 1 h (all other compounds) prior to fixation in 1:3 acetic acid/methanol. Scale bar: 2 μm. (B) Quantification of microscopy images from (A) shown as a ratio of bubbles per total number of cells. Error bars show the standard deviation of three data sets. A minimum of 50 cells were examined per individual sample. The solid red line indicates the average, and the dotted red line, the upper margin of standard deviation in the untreated control sample.

First, we tested peptidoglycan integrity with an established acetic acid/methanol fixation protocol.^36,37^ If the cell wall is compromised, the protoplast can protrude through cell wall breaches, which is promoted by the fixation and can be observed as “bubbles” on the cell surface in phase contrast microscopy.^36^ No strong effects comparable to those of the positive control vancomycin were observed, yet compounds **7c**, **7d**, **7f**, and **8a** showed slightly elevated numbers of bubbles (Figure 7, Table 2), suggesting mildly compromised peptidoglycan integrity.

**Figure 8:**
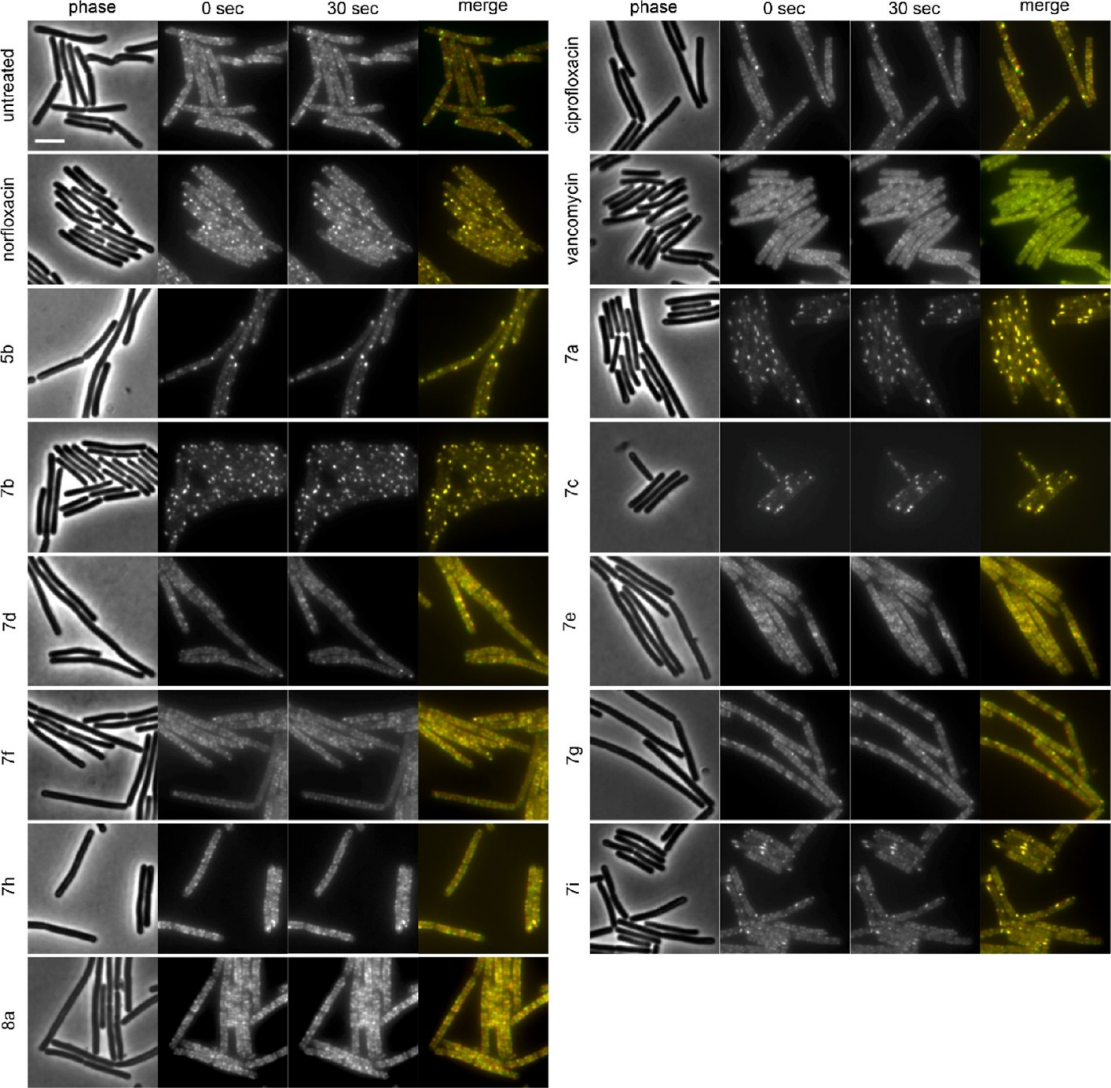
Fluorescence and phase contrast microscopy of *B. subtilis* MW10. Expression of MreB-msfGFP was induced with 0.3% xylose. Cells were treated with 1xMIC of the respective compounds for 10 min (vancomycin) or 60 min (all other compounds) prior to microscopy. To capture MreB mobility, two separate images were taken 30 s apart, false-colored green and red, and overlaid (yellow). Scale bar 2 μm.

Acetic acid/methanol fixation only tests positive when cell wall autolysins are active, is strongly concentration-dependent, and does not react to all types of cell wall synthesis inhibition.^36,37^ Moreover, it can also test positive when cell wall synthesis is impaired due to indirect effects, *e.g.*, an interference with membrane binding of cell wall synthetic proteins.^38^ It is therefore advisible to combine the fixation assay with additional cell wall reporters. Hence, we followed up with an MreB mobility assay using a GFP fusion to this cell wall synthesis regulation protein. MreB is an actin homologue that forms filaments that align along the lateral cell axis in a spiraling pattern. It moves along the long axis of the cell, thereby driving lipid II synthesis and ensuring rod shape.^39^ MreB localization can be affected by membrane depolarization, phase separation, and invaginations, as well as inhibition of cell wall synthesis, manifesting in partial clustering or loss of membrane binding of the protein. However, MreB mobility is highly sensitive and specific to the latter (Figure S96).^32,40^ To visualize MreB mobility, two images of the same field of view were taken 30 s apart, false-colored in green and red, and overlaid, resulting in a yellow signal where the images overlap. Distinct red and green foci indicate mobile MreB (see untreated control), while entirely yellow cells indicate loss of MreB mobility (see positive control vancomycin). Loss of MreB mobility was observed for compounds **5b**, **7a**, **7b**, **7c**, and **7i**, which was accompanied by clear clustering of MreB into distinct, immobile clusters at the cell membrane (Figure 8, Table 2), suggesting that these derivatives interfere with the cell wall synthesis machinery. While some clustering was observed as well in cells treated with norfloxacin and, to a lesser extent, ciprofloxacin, the remaining MreB in these cells retained normal mobility, suggesting that this clustering is due to membrane effects. Since depolarization could be excluded as part of their mechanism (Figure S95), the most likely causes of these effects are membrane phase separation or invaginations. Compounds **7d**, **7f**, and **8a**, which tested slightly positive in the acetic acid/methanol fixation assay, retained MreB mobility, suggesting that their peptidoglycan synthesis may be mildly compromised by indirect effects, *e.g.*, on the cell membrane.

**Figure 9:**
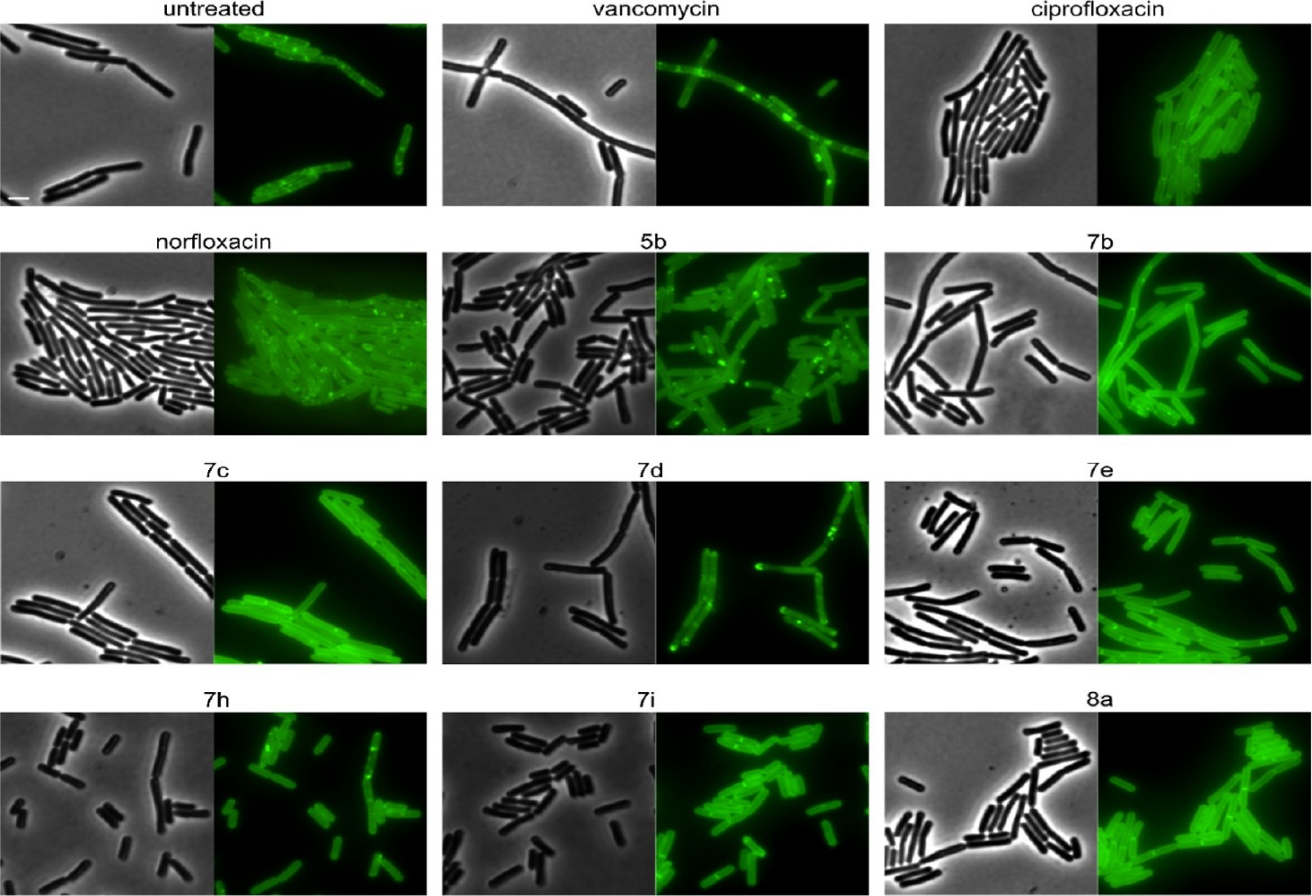
Fluorescence and phase contrast microscopy of *B. subtilis* TNVS175. Expression of MurG-msfGFP was induced with 0.05% xylose. Cells were treated with 1xMIC of the respective compounds for 10 min (vancomycin) or 1 h (all other compounds) prior to microscopy. Scale bar: 2 μm.

We then tested the effects on further cell wall synthesis proteins, including the lipid I synthase MraY, the lipid II synthase MurG, and the penicillin-binding proteins PbpB and PonA, which incorporate the precursor into the peptidoglycan cell wall all around the cell or specifically at the cell division septum, respectively.^41−43^ We first examined the localization of MurG, which is a peripheral membrane protein and the most sensitive out of the four. In fast-growing cells, MurG localizes in small spots at the membrane (see untreated control, “spotty”), while in slow-growing cells, its localization becomes smooth (see ciprofloxacin, “smooth”). If peptidoglycan synthesis is inhibited, it forms large clusters in the membrane, accompanied by loss of membrane binding (see vancomycin, “patchy/dispersed”). Compound **7d** showed the strongest effect with clear clusters and a partially dispersed GFP signal, as well as signs of phase separation and invaginations (Figure 9, Table 2). **5b**, **7b**, **7c**, **7e**, **7h**, **7i**, and **8a** showed smooth MurG localization, probably due to general growth inhibition, whereby **5b** and **7i** showed additional membrane clusters. Overall, no compound showed effects on MurG that would be characteristic for inhibition of lipid II (see vancomycin), suggesting that they do not affect peptidoglycan synthesis at the level of lipid II or its synthesis by the MurG enzyme.

Effects on MraY, PbpB, and PonA were then tested for compounds **5b**, **7d**, and **7h** (Figure S97, Table 2). None of the tested compounds affected the localization of these proteins. Yet, norfloxacin showed clear clustering on PonA, suggesting that it may have a so-far unknown secondary mechanism on the divisome.

Most cell wall synthesis inhibitors inhibit the synthesis of lipid II by binding to either lipid-linked cell wall precursors (bactoprenol phosphate/pyrophosphate, lipid I/II) or penicillin-binding proteins, and less often to membrane-bound (MraY and MurG) or intracellular lipid II synthesis enzymes (MurA and d-alanine racemase/ligase). While compounds **5b**, **7a**, **7b**, **7c**, and **7i** stopped MreB motion and are thus likely to impair cell wall synthesis, they did not elicit characteristic phenotypes on MurG, MraY, PbpB, and PonA, and no compound showed strong effects in the acetic acid/methanol assay, which typically indicates inhibition of the lipid II cycle.^36,37^ Thus, we conclude that they may rather inhibit a so-far unknown, likely intracellular target affecting cell wall synthesis.

## Conclusions

Here, we synthesized two series of amino acid derivatives of norfloxacin. Several of the new compounds showed increased antibacterial and antimycobacterial activity compared to their parent compounds. This could be attributed to the more efficient inhibition of gyrase and topoisomerase IV, likely due to their ability to form additional bonds with these enzymes. Phenotypic analysis validated topoisomerase inhibition *in vivo* and revealed additional effects on the cell wall synthesis machinery (**5b**, **7a**, **7b**, **7c**, and **7i**) and/or the cytoplasmic membrane (**7d**, **7f**, and **8a**), which likely contribute to the increased antibacterial activity. Such polypharmacological properties can be beneficial for antibiotic drug candidates, as resistance develops much faster against single-target molecules.^5^ Inhibition of cell wall synthesis appeared to occur outside of the lipid II cycle, suggesting that the compounds have a novel secondary target. This is an interesting finding, as new antibiotic targets are highly desirable to avoid cross-resistance with existing drugs. Moreover, several of our compounds showed a narrow activity spectrum, being active against either Gram-negative bacteria (*e.g.*, **4d**, **7i**) or Gram-positive and mycobacteria (*e.g.*, **7g**, **8c**). Modern fluoroquinolones are typically broad-spectrum antibiotics. While these are certainly very important drugs, they can put considerable strain on the microbiome, making compounds with a narrower activity spectrum attractive for cases where the pathogen is known. Taken together, our novel compounds provide an interesting starting point for further derivatization approaches aimed at creating new fluoroquinolones with polypharmacological properties.

## Supporting Information Available

Supporting Information associated with this article contains: chemical characterization of compounds, molecular modeling studies, mode of action data, and methods (PDF)
