# Supplementary material for: Design, Synthesis, Molecular Modeling, Biological Activity, and Mechanism of Action of Novel Amino Acid Derivatives of Norfloxacin
Source: ACS Omega. 2023 Nov 1;8(45):43271–84. doi: 10.1021/acsomega.3c07221 (PMC10653056; doi:10.1021/acsomega.3c07221)
Supplement: Supplementary file 1 — ao3c07221_si_001.pdf [file ao3c07221_si_001.pdf]

Supplementary material

Design, Synthesis, Molecular Modeling, Biological Activity, and Mechanism of Action of Novel Amino Acid Derivatives of Norfloxacin

Ahmed M. Kamal El-sagheir[1], Ireny Abdelmesseh Nekhala[2], Mohammed K. Abd El-Gaber[1], Ahmed S. Aboraia[1], Jonatan Persson[2,3], Ann-Britt Schäfer[2,3], Michaela Wenzel[2,3*], Farghaly A. Omar[1].

[1]Medicinal Chemistry Department, Faculty of Pharmacy, Assiut University, Assiut, Egypt, 71526.

[2]Division of Chemical Biology, Department of Life Sciences, Chalmers University of Technology, 412 96 Gothenburg, Sweden.

[3]Center for Antibiotic Resistance Research in Gothenburg (CARe), Gothenburg, Sweden.

*Corresponding authors: Michaela Wenzel (wenzelm@chalmers.se) and Farghaly A. Omar (farghalyomar@pharm.aun.edu.eg)

Table of contents

1. **Chemical characterization of compounds**

   Figure S1-56: ^{1}H NMR and ^{13}C NMR spectra

   Table S1: Elemental analysis of newly synthesized compounds.

2. **Prediction of ADME/tox**

   Text S1: Prediction of physicochemical properties

   Table S2: Predicted physicochemical properties.

   Text S2: ADME/Tox prediction using pKCSM lab

   Table S3: ADME/Tox properties predicted by pKCSM

   Text S3: Prediction of physicochemical and pharmacokinetic properties by SwissADME

   Table S4: Physicochemical and pharmacokinetic properties predicted by SwissADME

   Figure S57: Therapeutic windows against different bacterial species based on toxicity against SH-SY5Y and WI-38 cell lines

3. **Molecular modeling**

   Figures S58-59: 2D and 3D interactions with *S. aureus* DNA gyrase

   Figure S60-63: 2D and 3D interactions with *A. baumannii* DNA topoisomerase IV

   Text S4: Docking on *P. aeruginosa* LpxC

   Figure S64-76: 2D and 3D interactions with *P. aeruginosa* LpxC

   Text S5: Docking on *M. smegmatis* NagA

   Figure S77-86: 2D and 3D interactions with *M. smegmatis* NagA

   Text S6: Ligand based pharmacophore modelling.

   Table S5: Query features calculated from the aligned molecules

   Table S6: Pharmacophore features with distance constraints (Å)

   Figure S87: Query features calculated from the aligned molecules

   Figure S88: Overlapping of some target compounds (**4b, 5a, 7b** and **8b**) with the generated pharmacophore query

   Figure S89: Alignment of **5d** compound and CHIR-90

4. **Mechanism of action**

   Table S7: Absorbance of investigated compounds and their metal complexes

   Figure S90: UV–vis absorption spectra of compounds **5b, 7b, 7i, 8a,** and **8c** in complex with metals

   Figure S91: Molar ratio of ligand/metal in metal complex of compound **8c**

   Figure S92: Fluorescence and phase contrast microscopy of *E. coli* BCB472

   Table S8: Results summary of bacterial cytological profiling of *E. coli*

   Table S9: Results of checkerboard assays of norfloxacin derivatives combined with mupirocin

   Figure S93: Effects on LpxC

   Table S10: Minimal inhibitory concentrations against *B. subtilis* DSM402

Figure S94: Bacterial cytological profiling of *B. subtilis*
Table S11: Results summary of bacterial cytological profiling of *B. subtilis*
Figure S95: Effects of the membrane potential in *B. subtilis* DSM402
Figure S96: MreB mobility after treatment with MP196
Figure S97: Fluorescence and phase contrast microscopy of *B. subtilis* TNVS284

5. **Methods**
   Text S7: Chemistry
   Text S8: Molecular modeling
   Figure S98: Color scheme for the 2D representations of the interactions between the docked ligands and the active site of the enzyme
   Table S12: Training set compounds
   Table S13: Validation test set compounds
   Text S9: Biology
   Table S14: Strains used in this study
6. **References**
   Text S10: References

1. Chemical characterization of compounds

2a

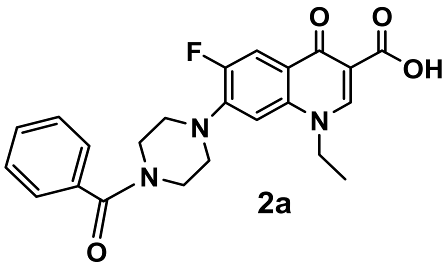

2a

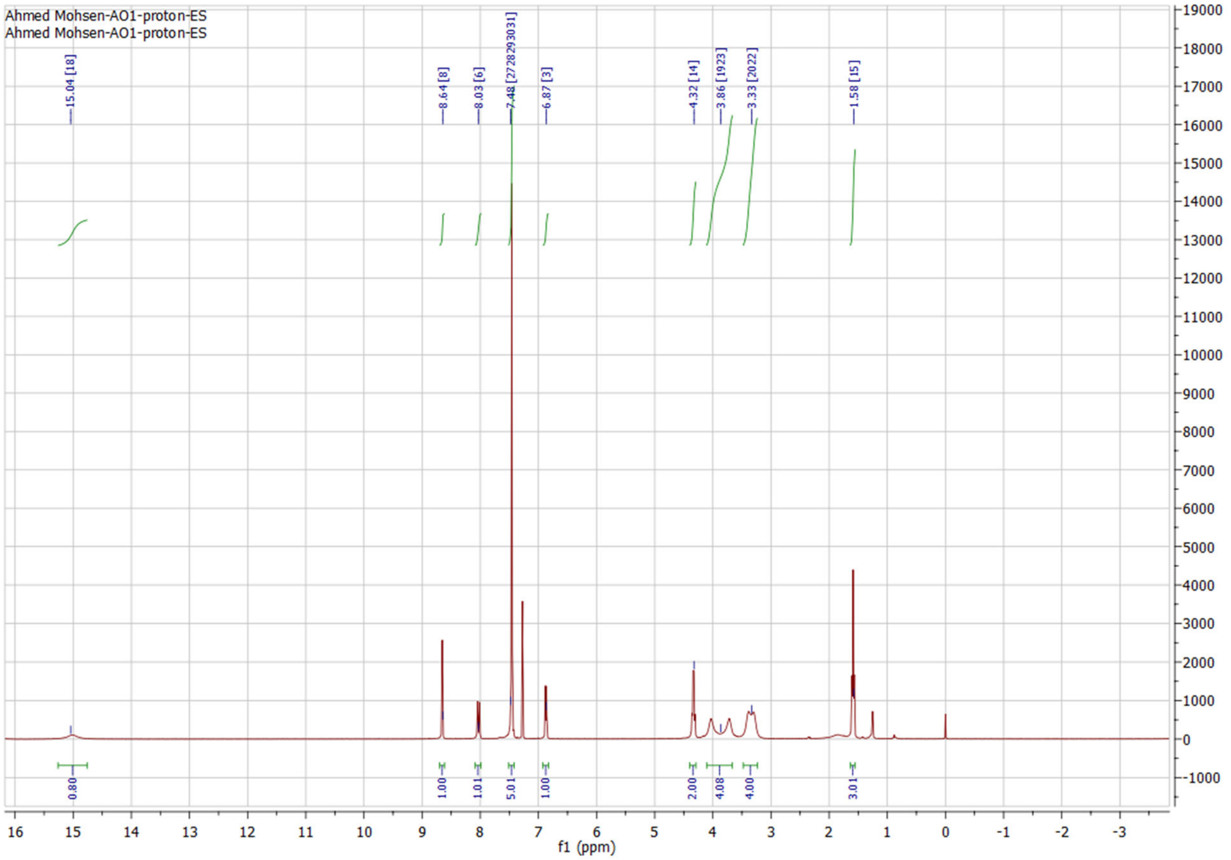

Figure S1: ^{1}H NMR of compound 2a

2b

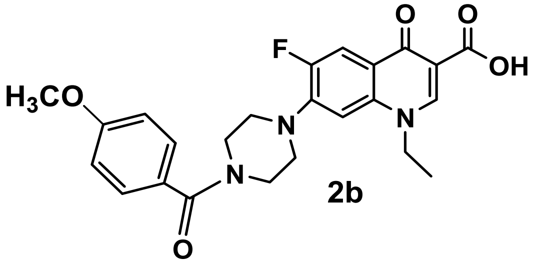

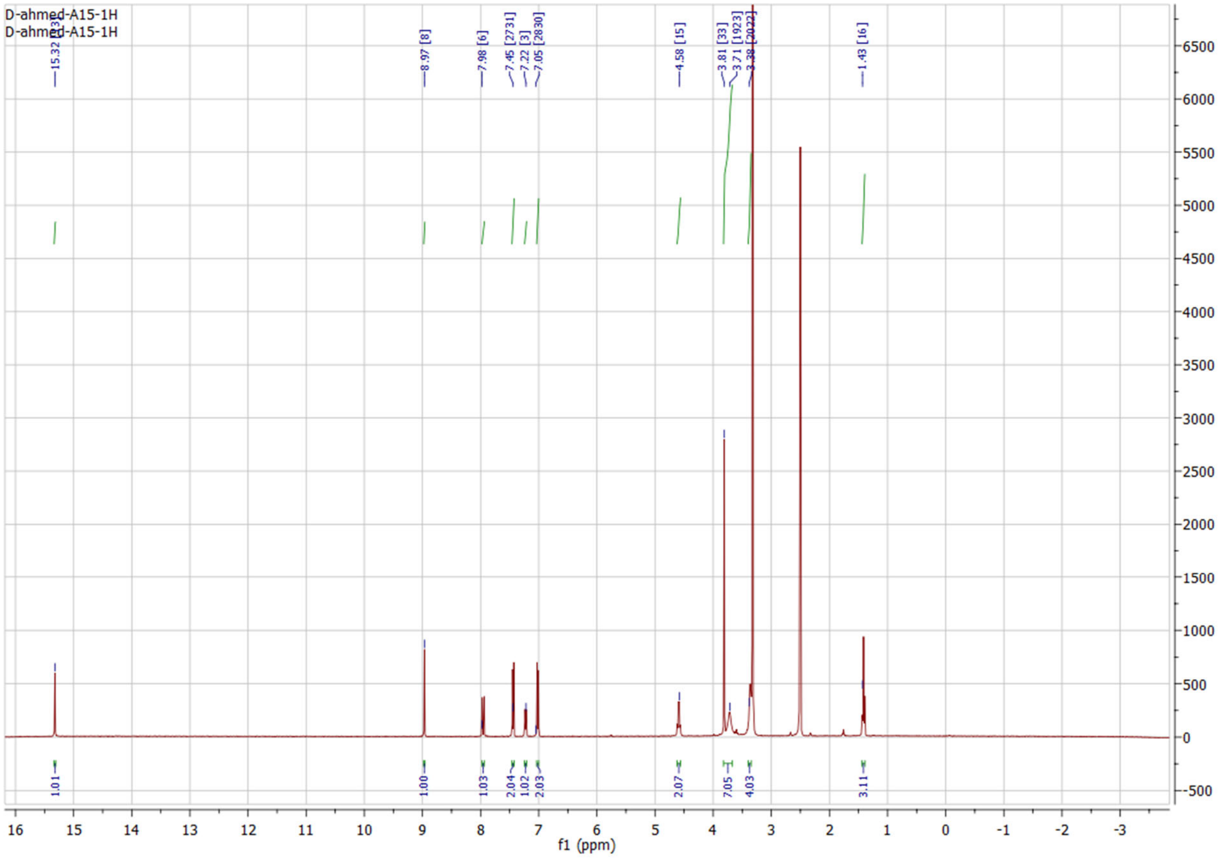

Figure S2: ^{1}H NMR of compound 2b

2c

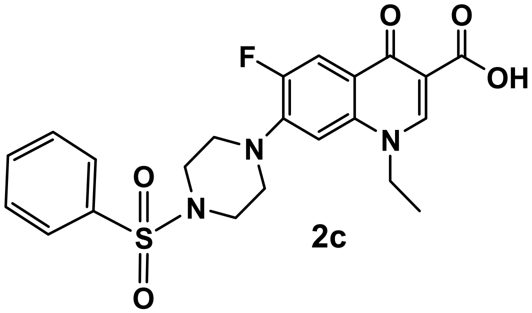

2c

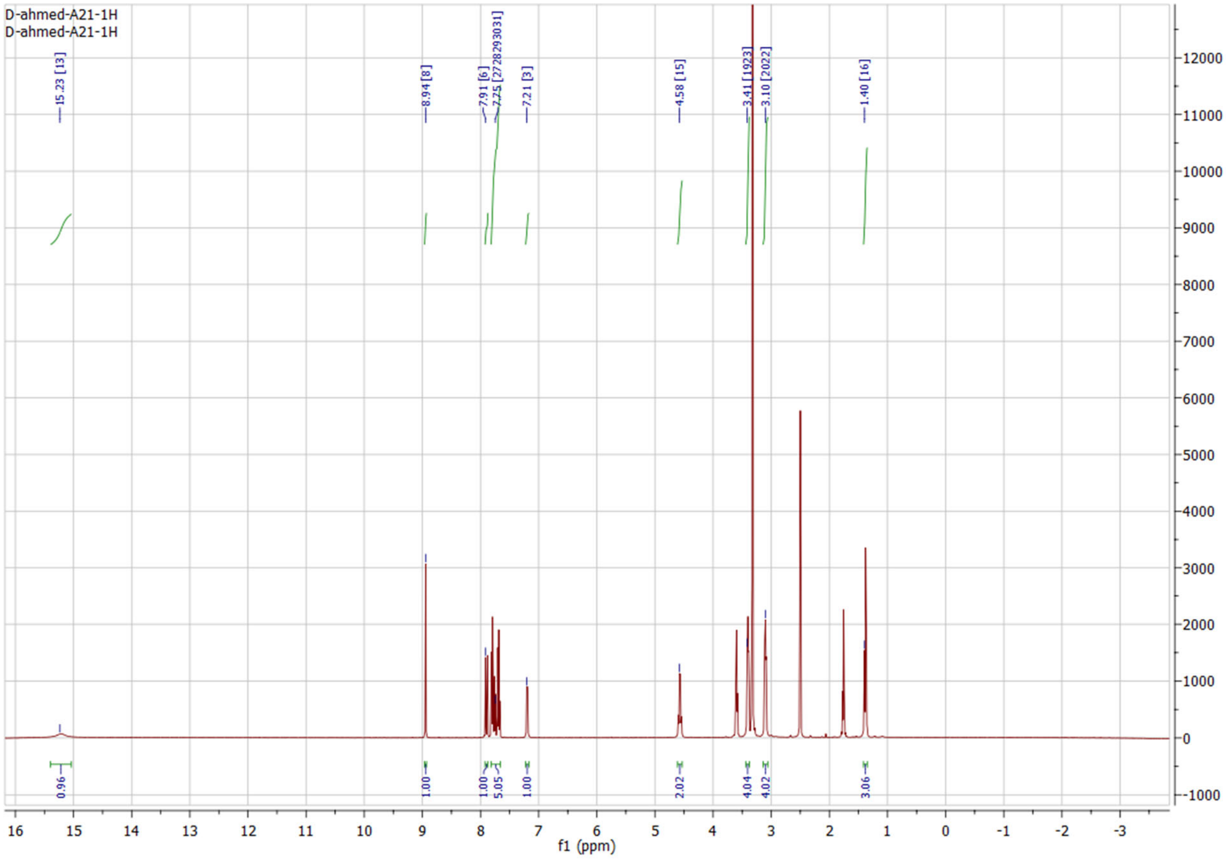

Figure S3: ^{1}H NMR of compound 2c

2d

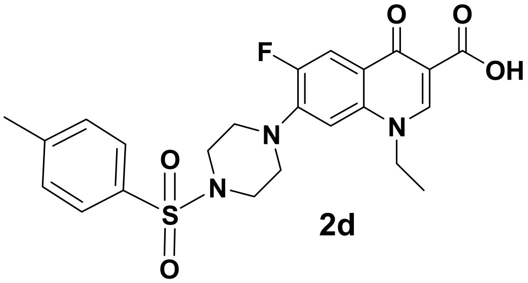

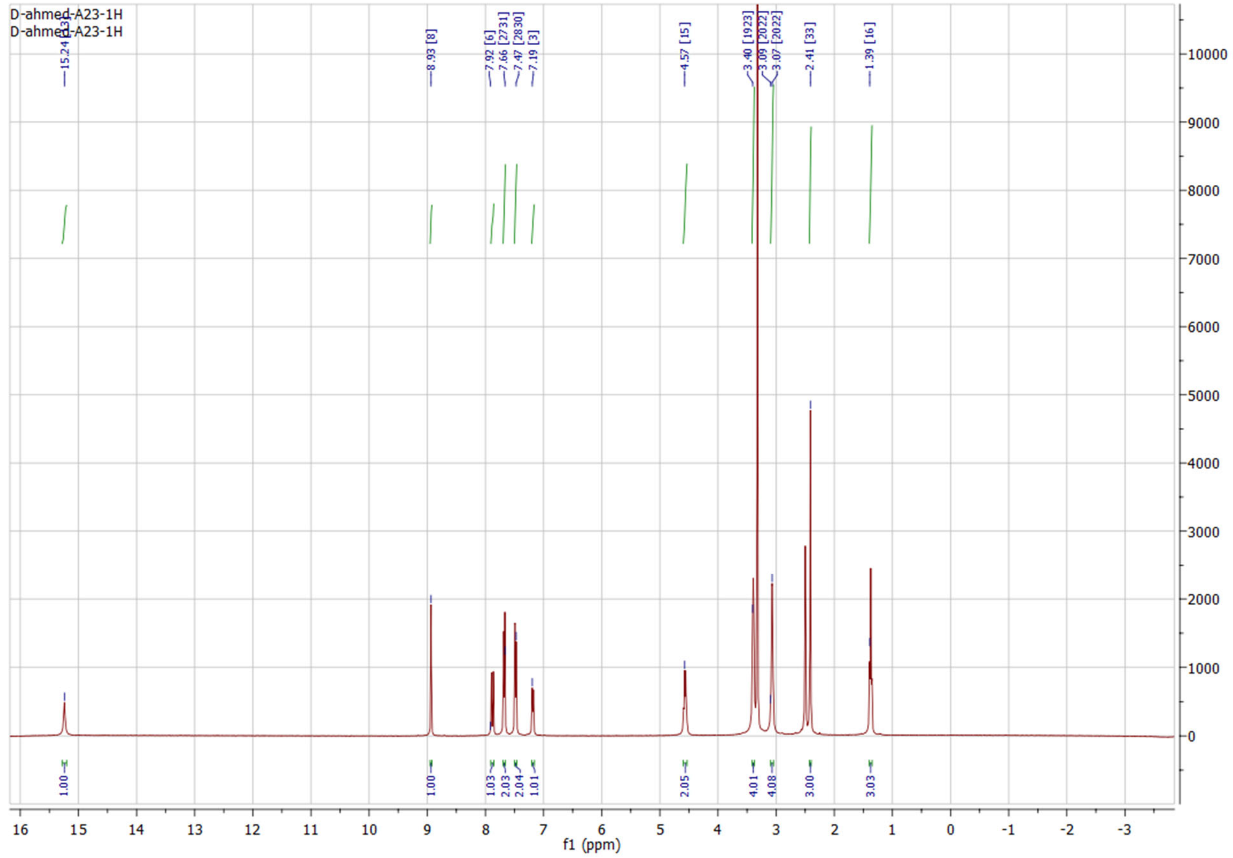

Figure S4: ^{1}H NMR of compound 2d

2e

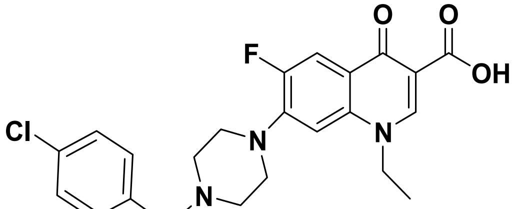

2e

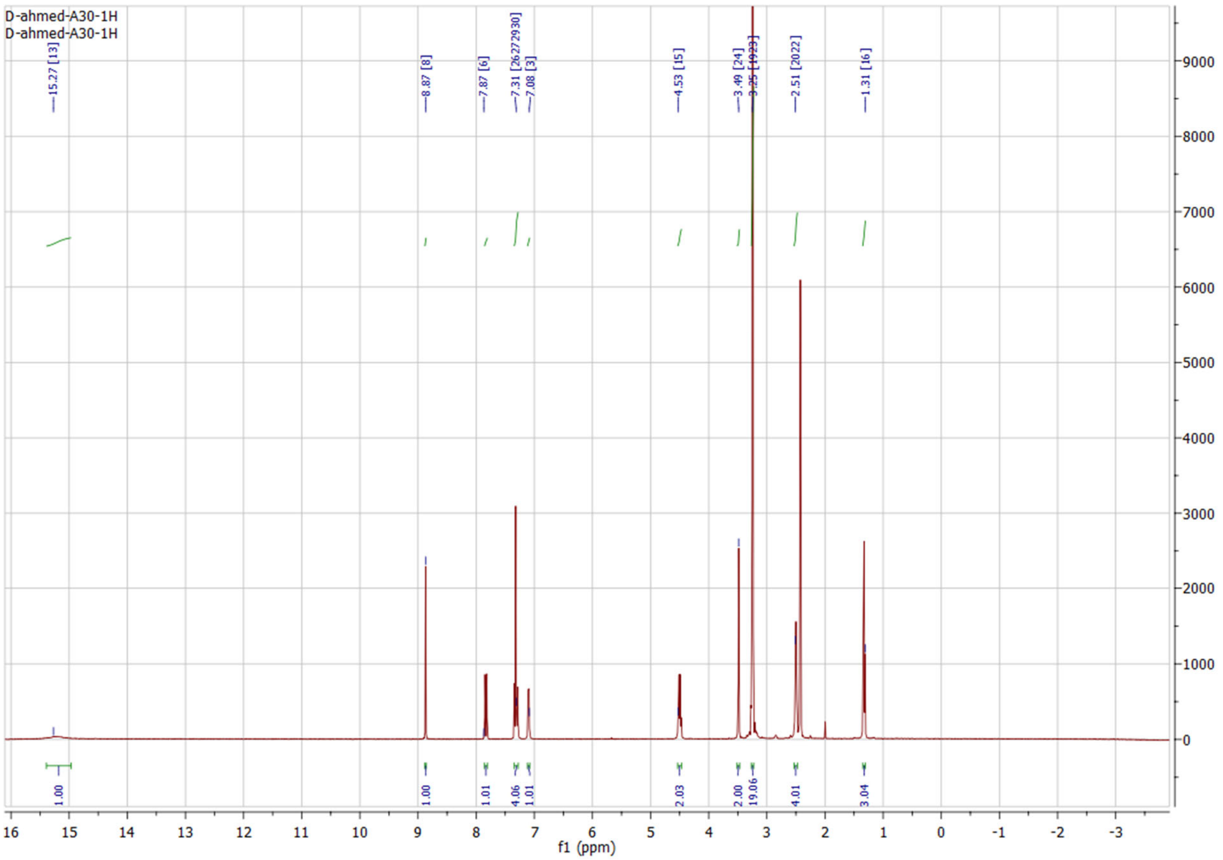

Figure S5: ^{1}H NMR of compound 2e

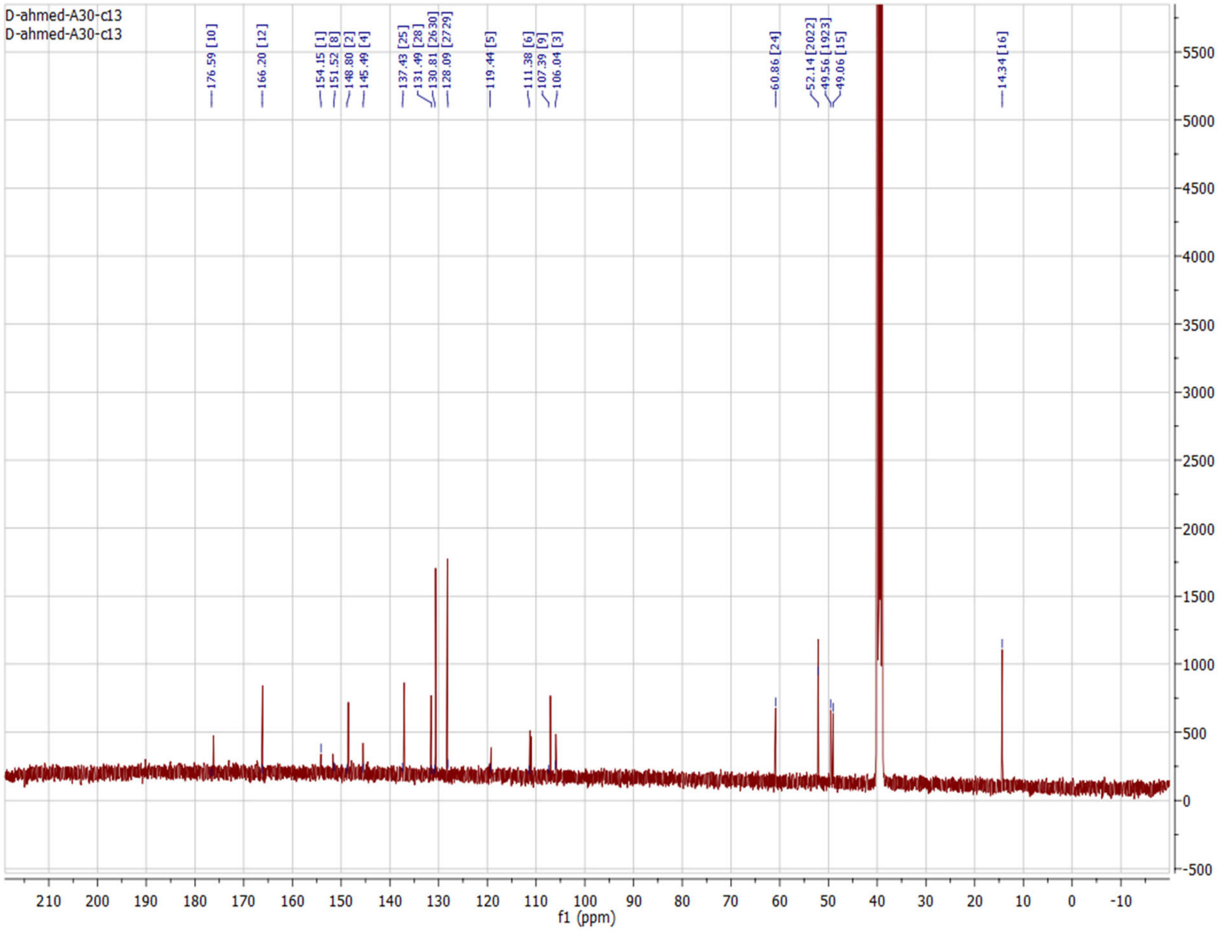

Figure S6: ^{13}CNMR of compound 2e

2f

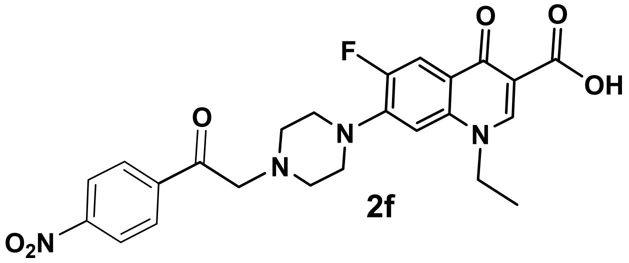

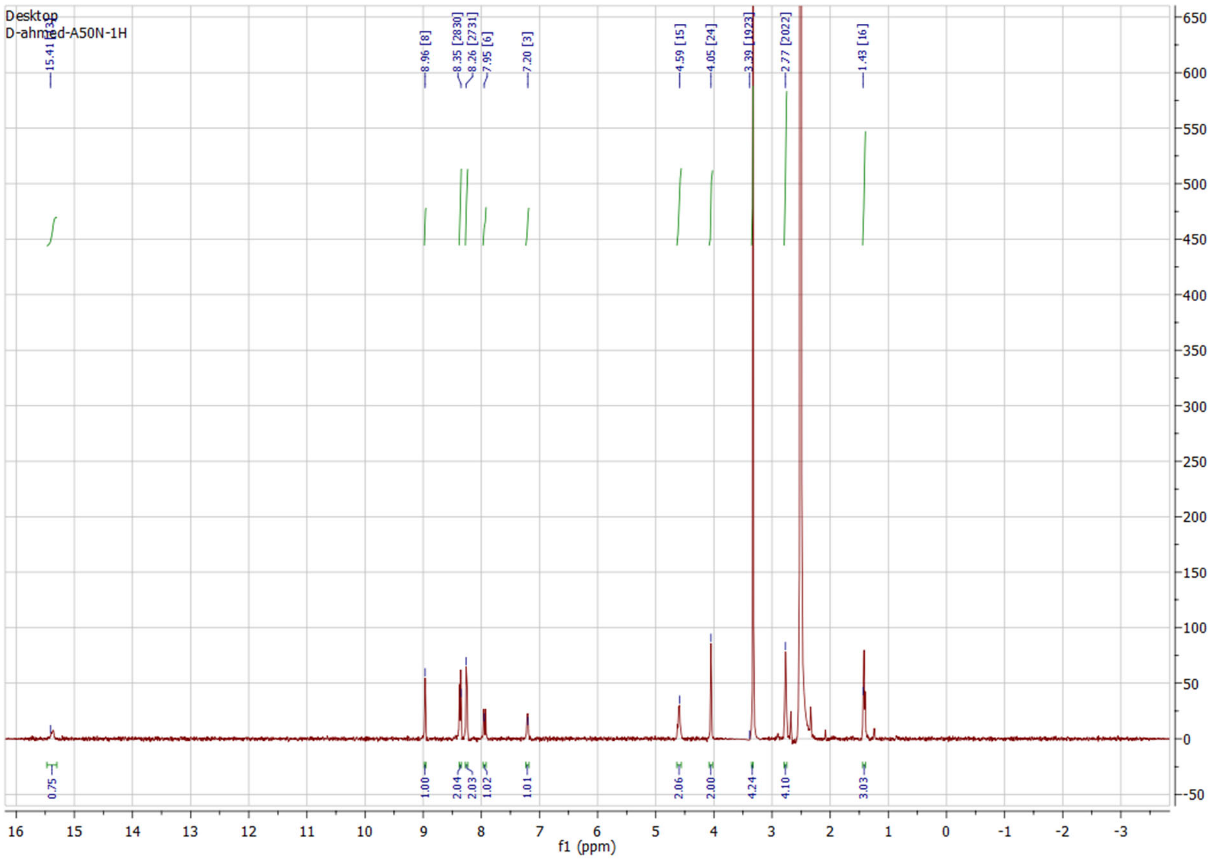

Figure S7: ^{1}H NMR of compound 2f

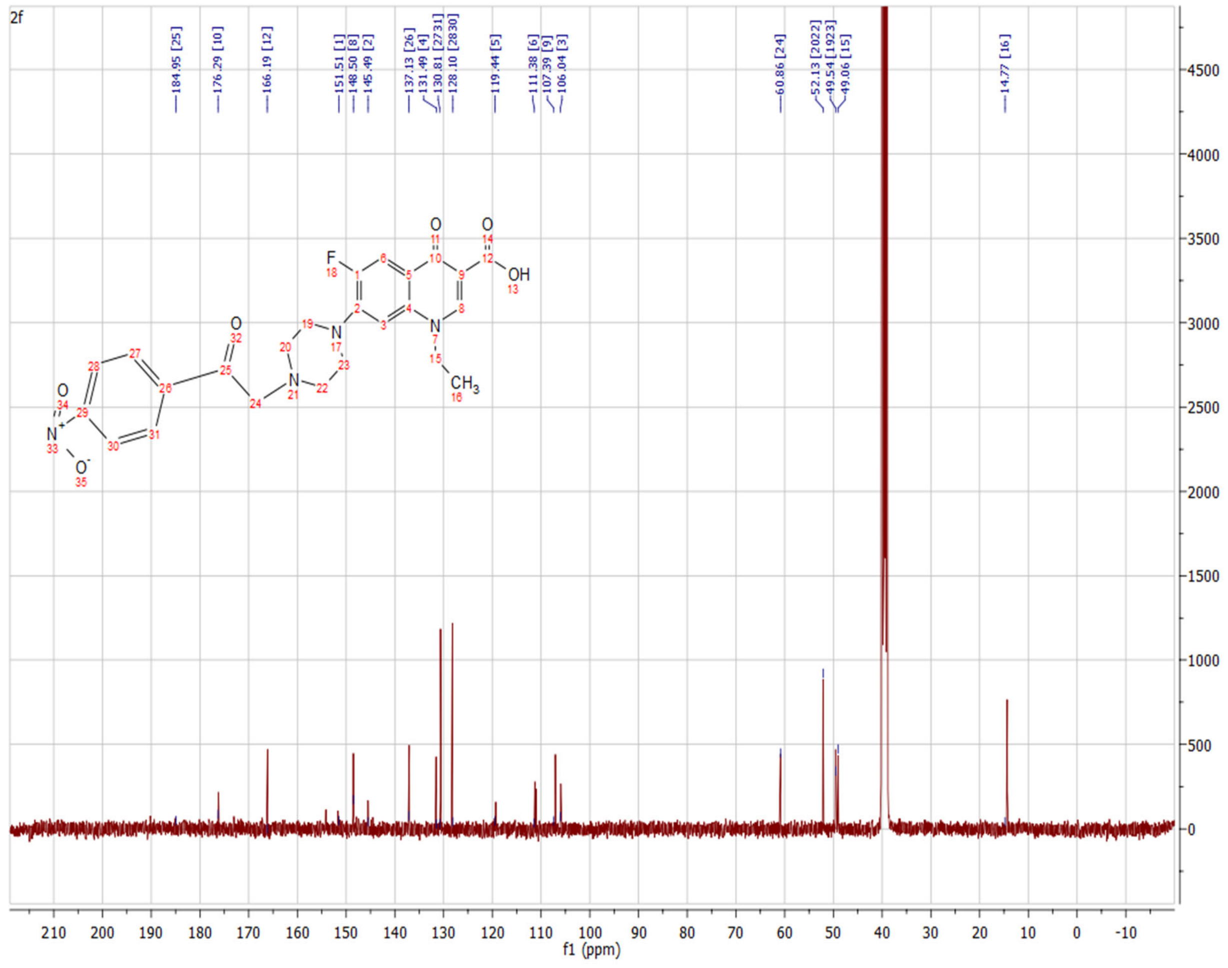

Figure S8: ^{13}CNMR of compound **2f**

4a

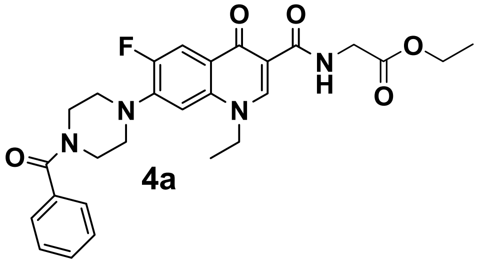

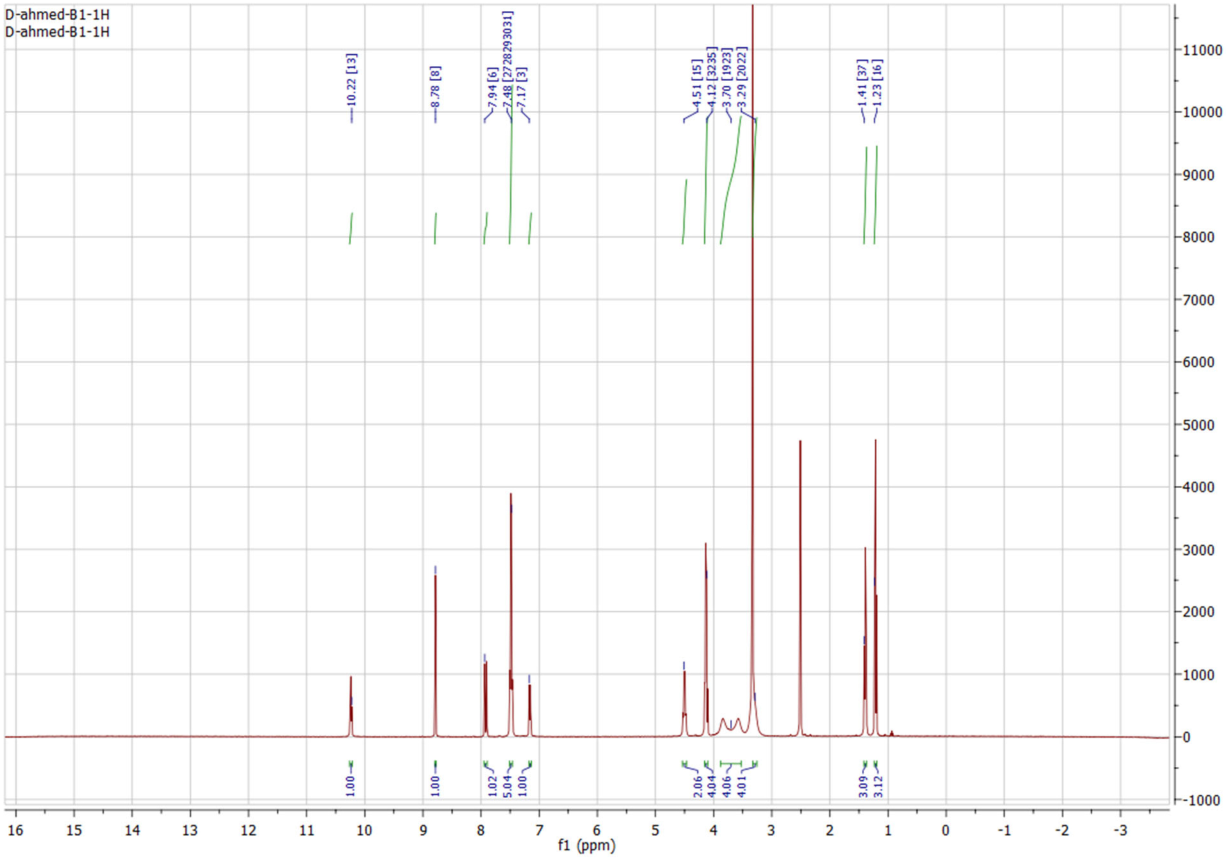

Figure S9: ^{1}H NMR of compound 4a

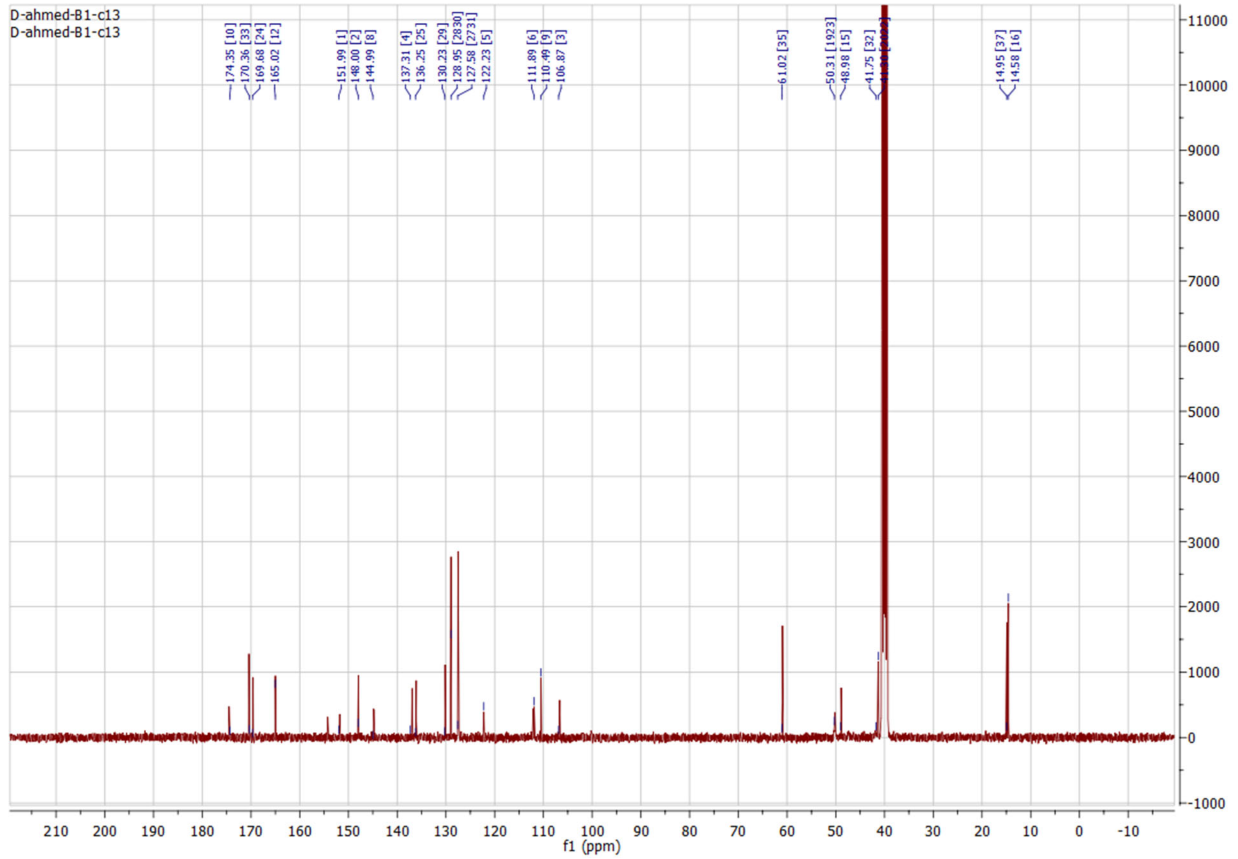

Figure S10: ^{13}C NMR of compound 4a

4b

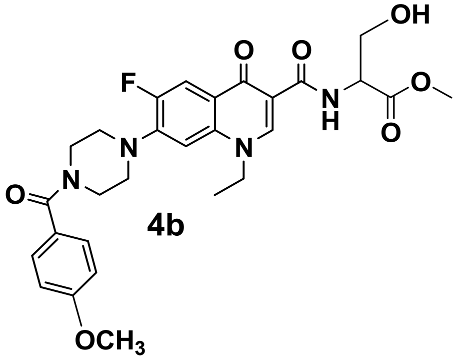

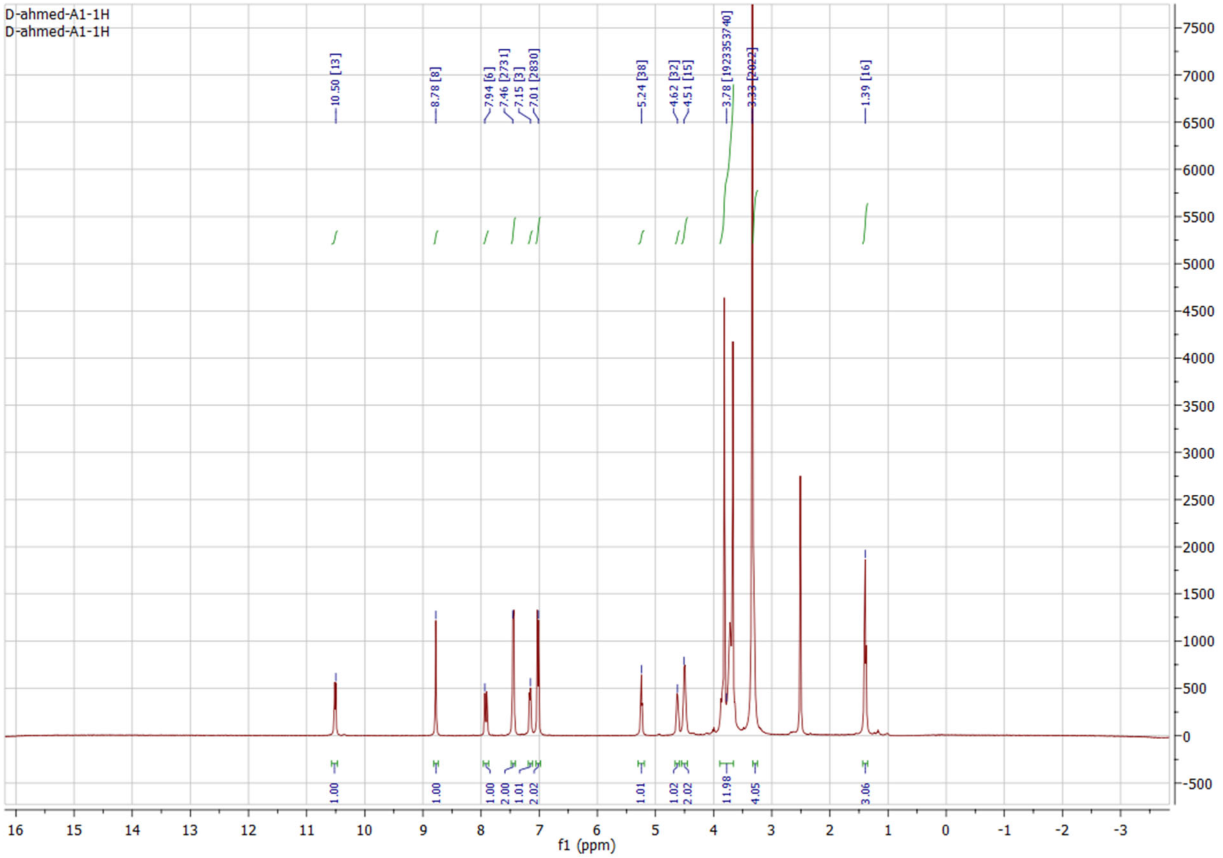

Figure S11: ^{1}H NMR of compound 4b

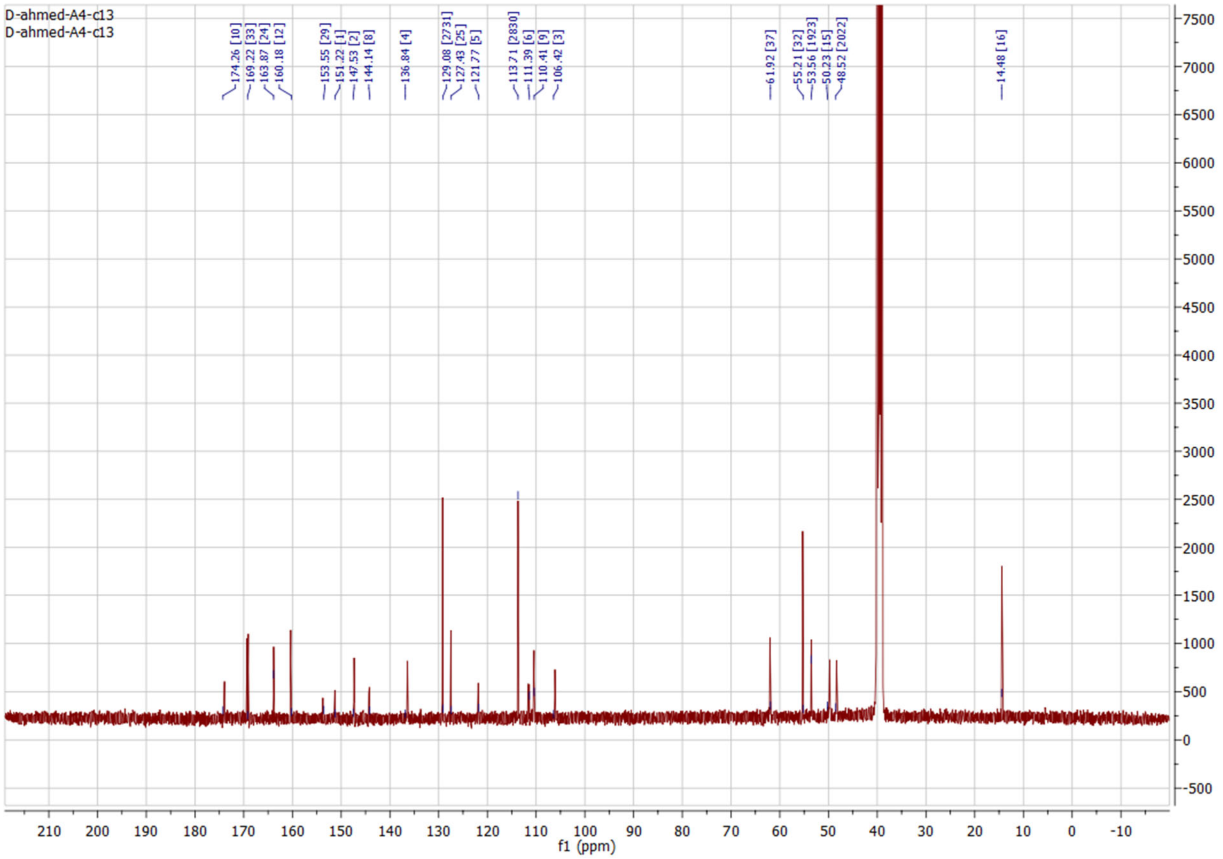

Figure S12: ^{13}C NMR of compound 4b

4c

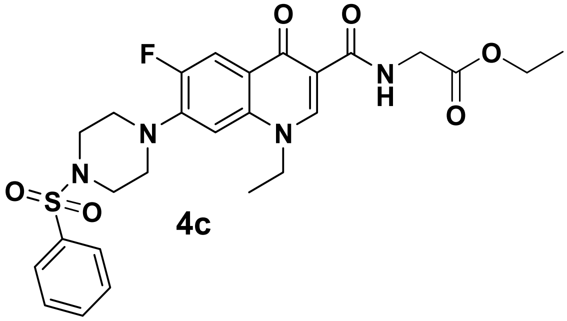

4c

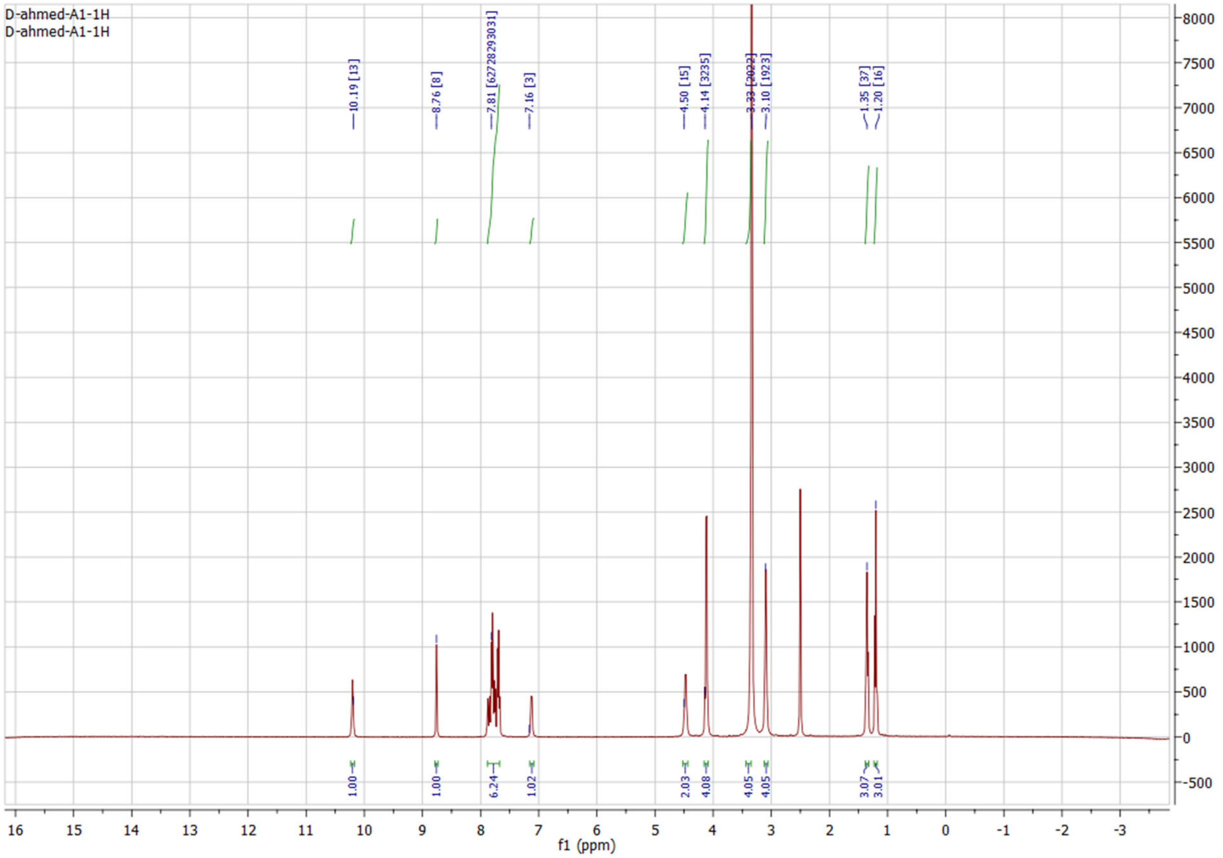

Figure S13: ^{1}H NMR of compound 4c

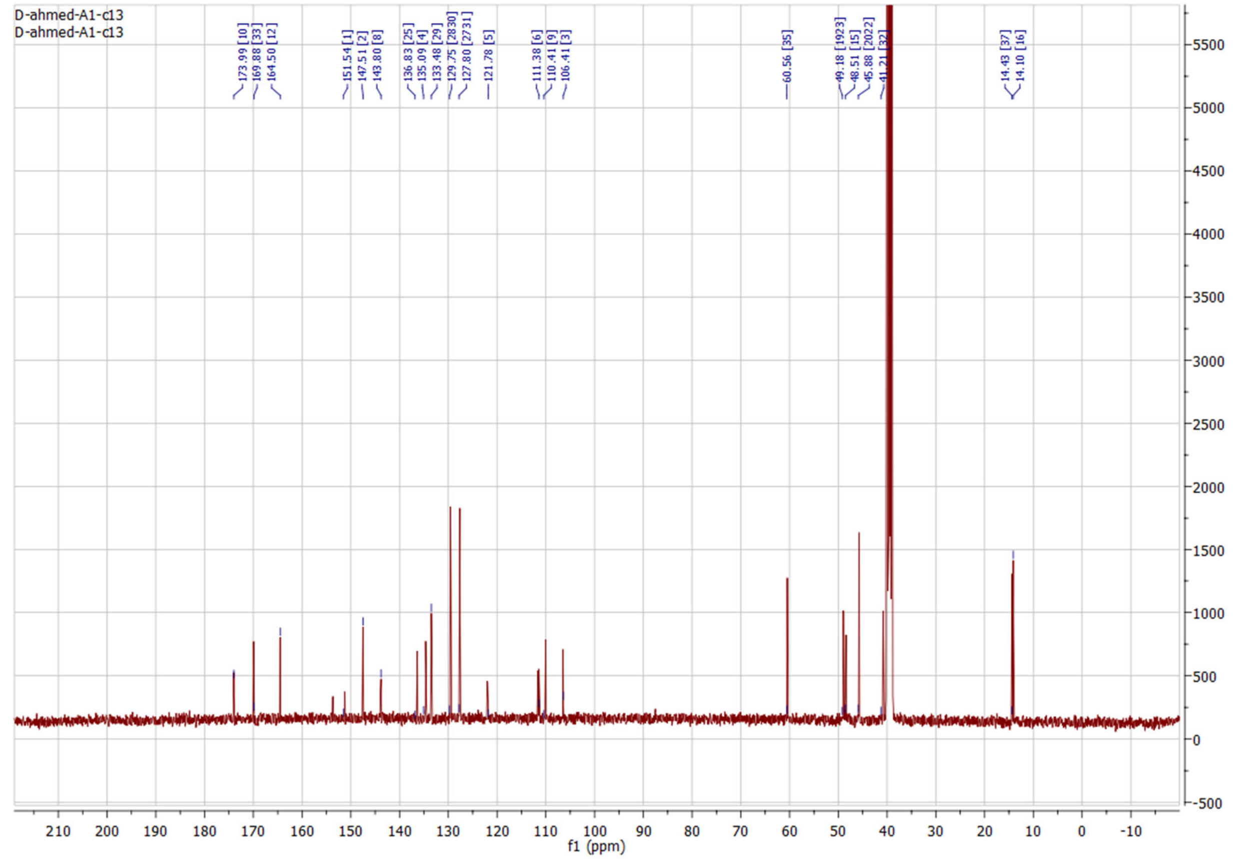

Figure S14: ^{13}C NMR of compound 4c

4d

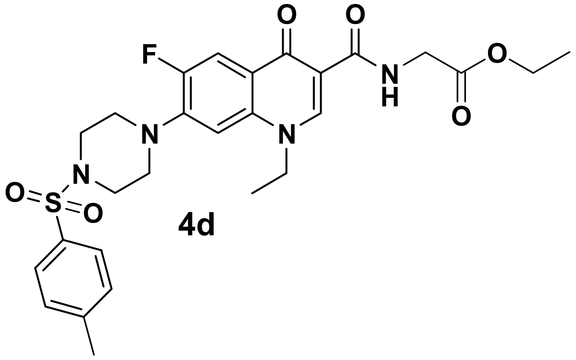

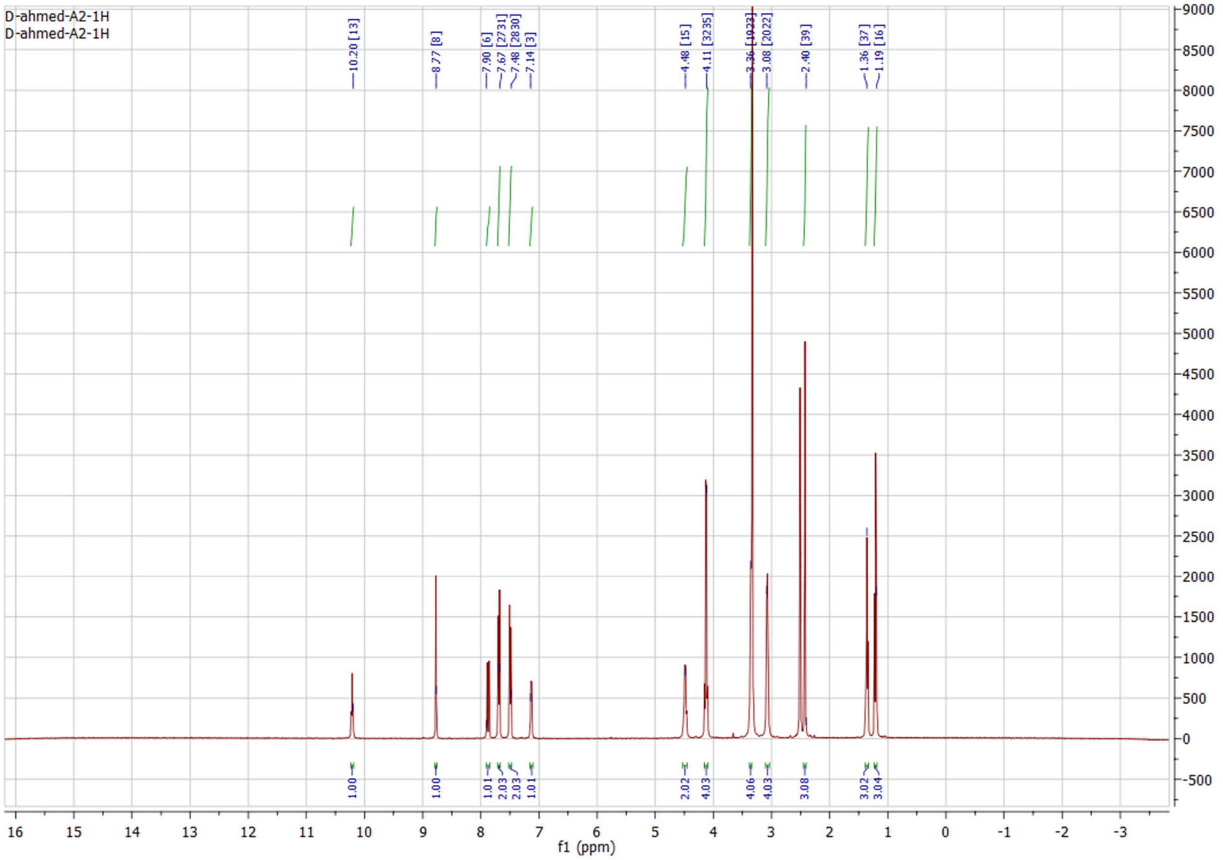

Figure S15: ^{1}H NMR of compound 4d

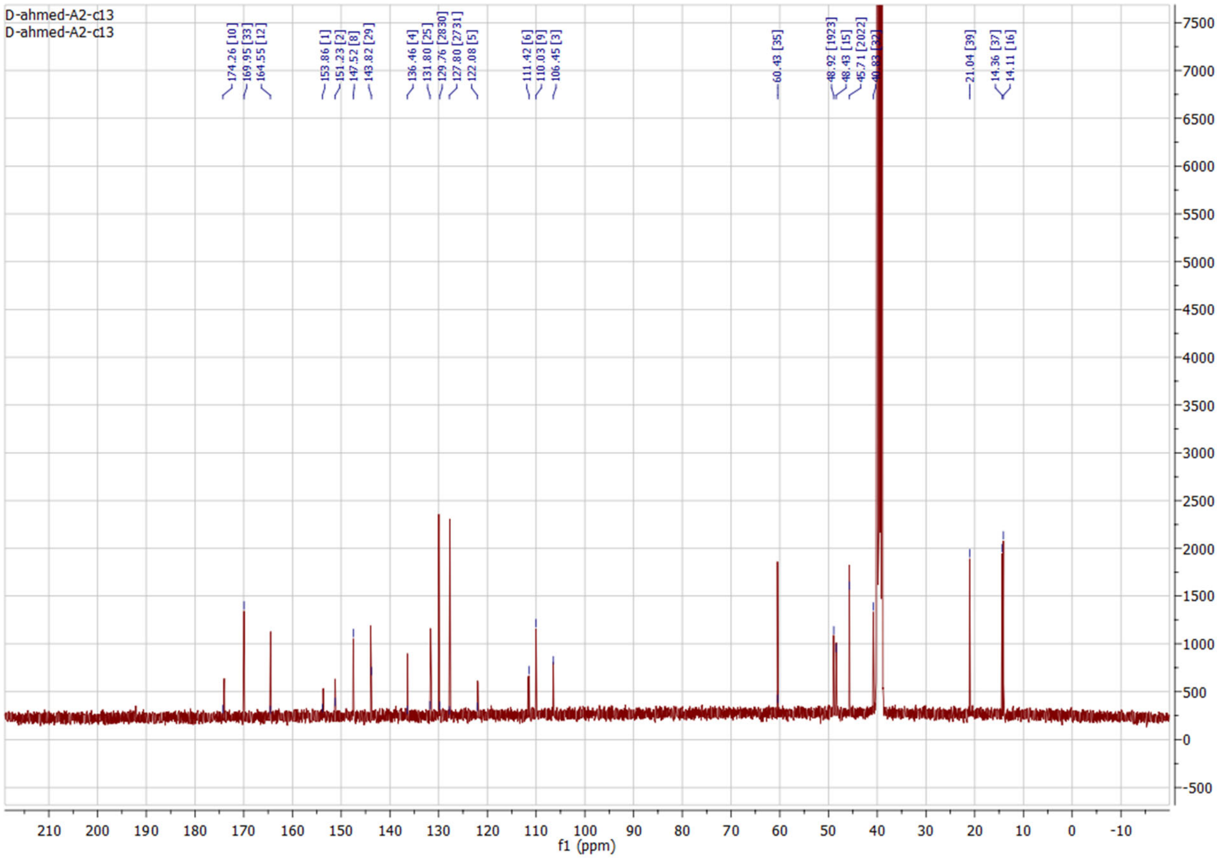

Figure S16: ^{13}C NMR of compound 4d

4e

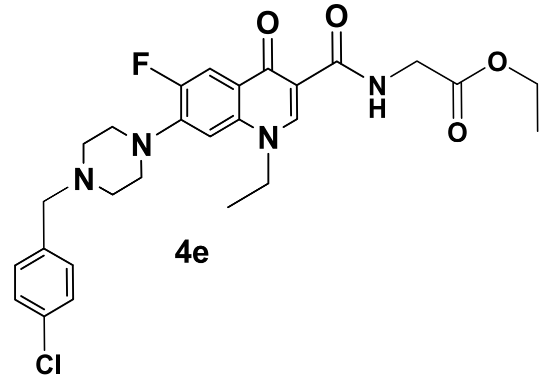

4e

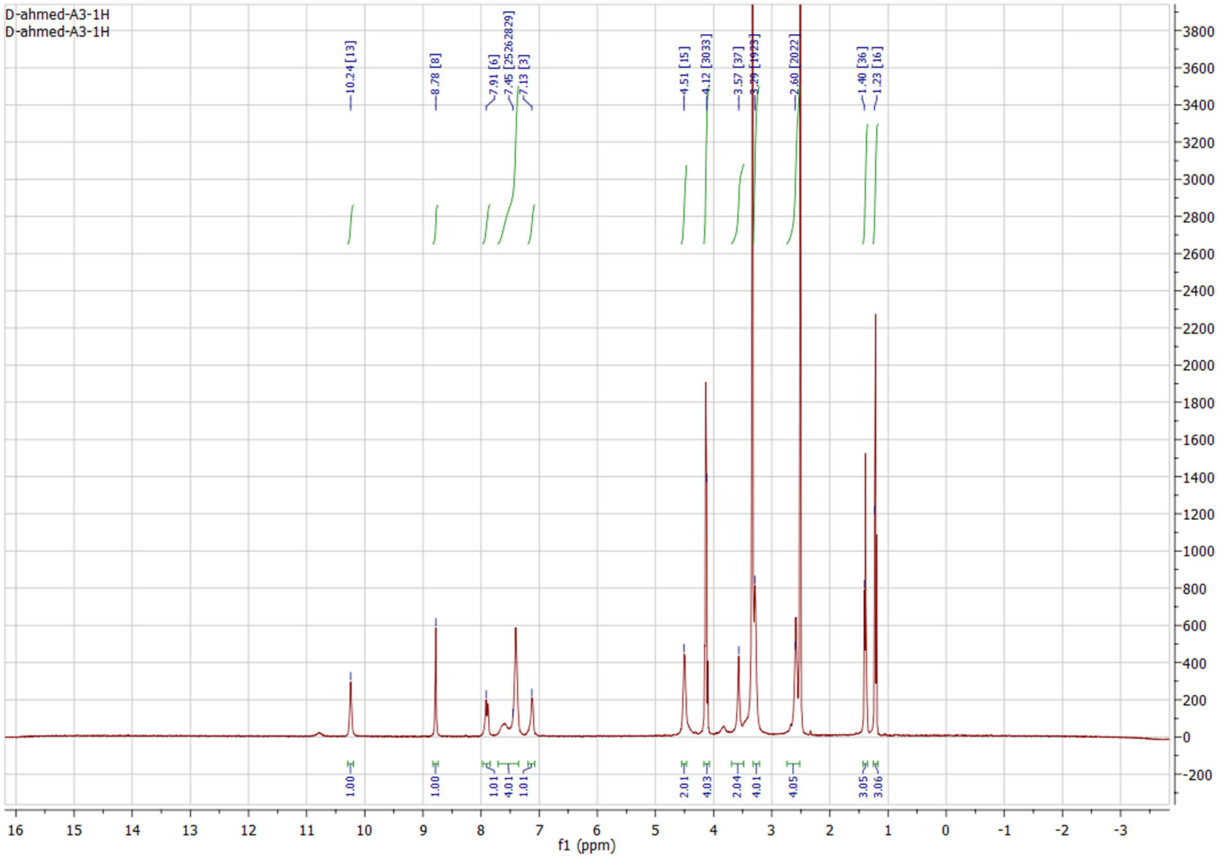

Figure S17: ^{1}H NMR of compound 4e

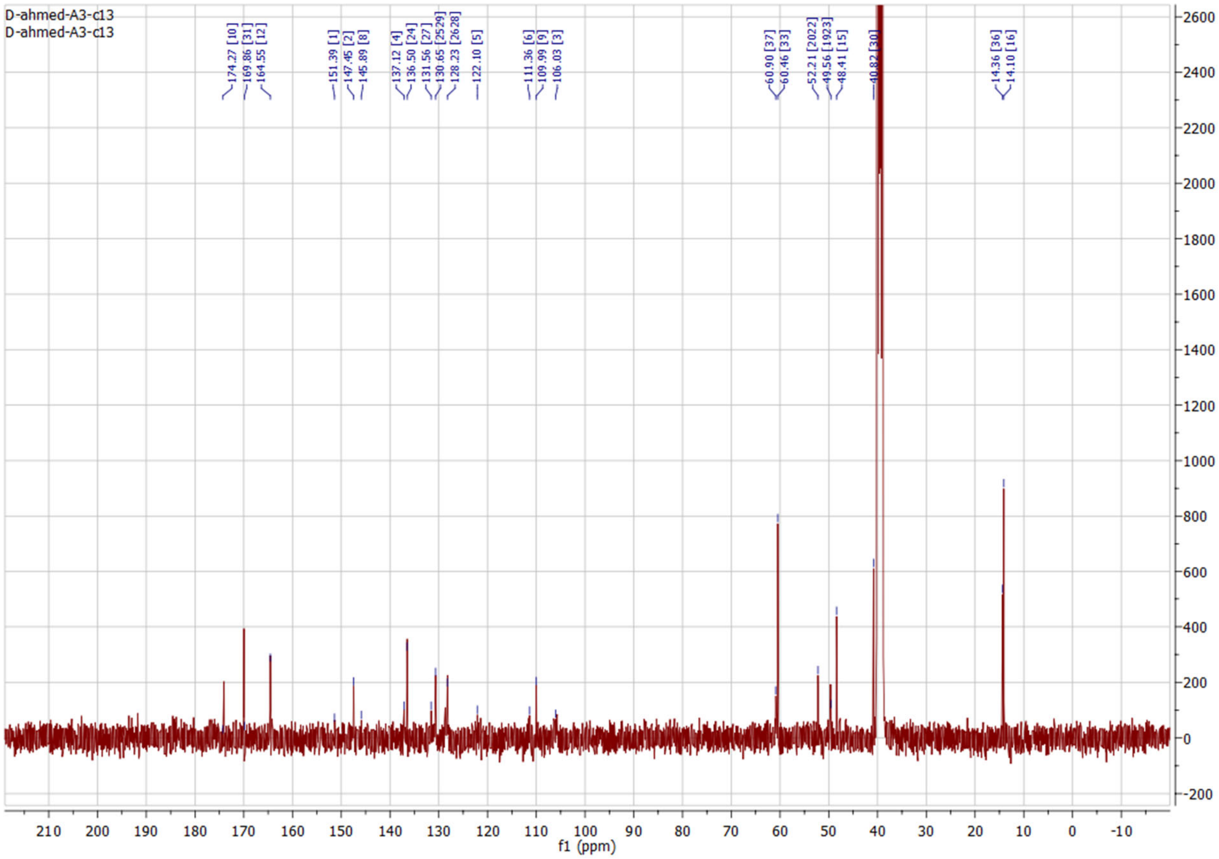

Figure S18: ^{13}C NMR of compound 4e

4f

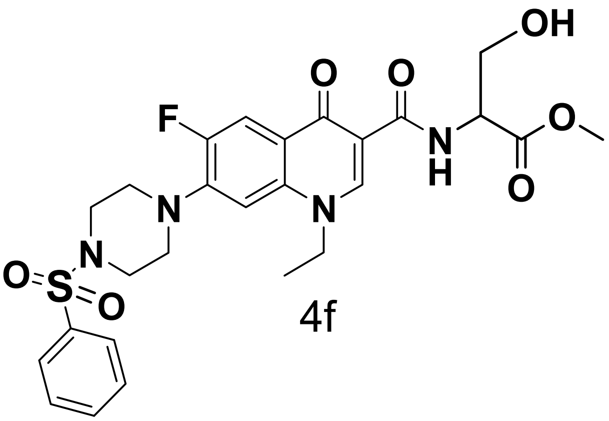

4f

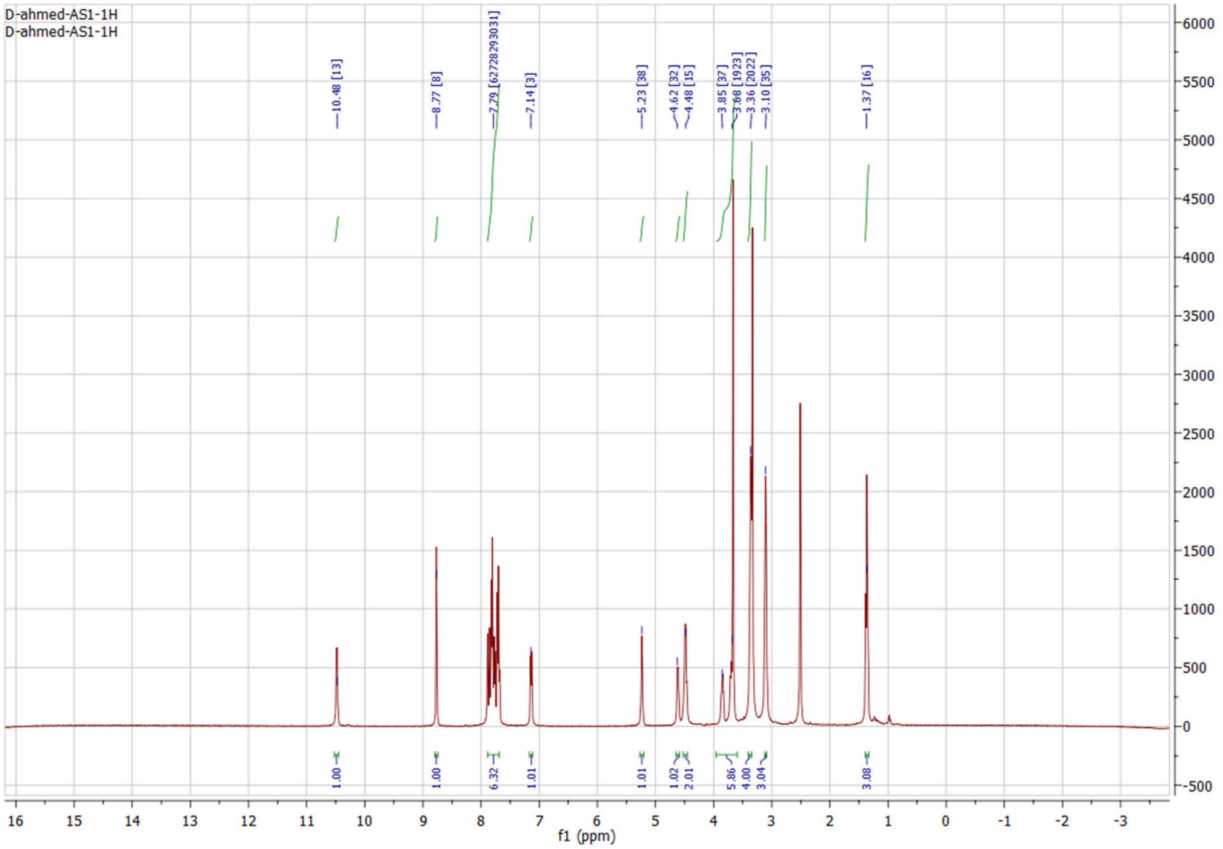

Figure S19: ^{1}H NMR of compound 4f

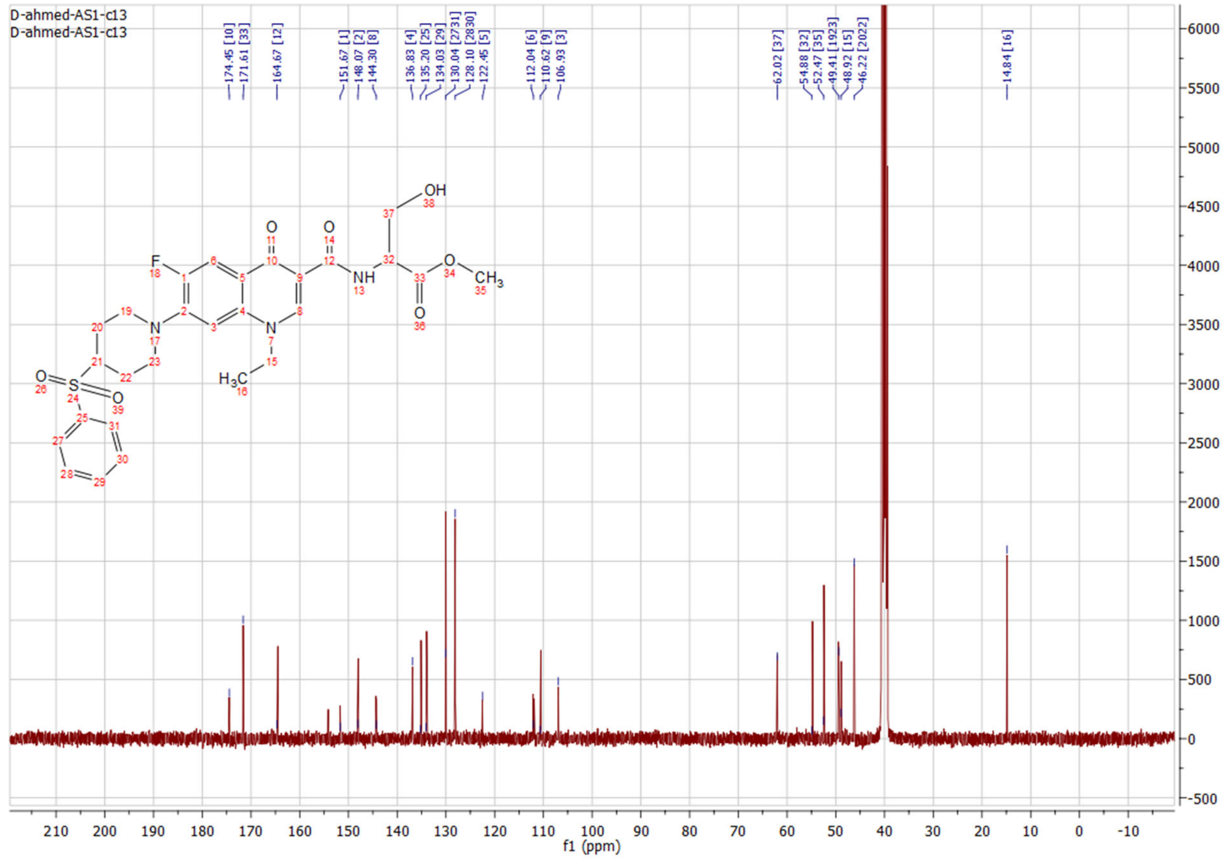

Figure S20: ^{13}C NMR of compound 4f

5a

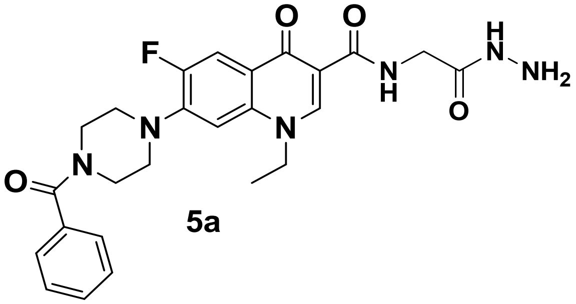

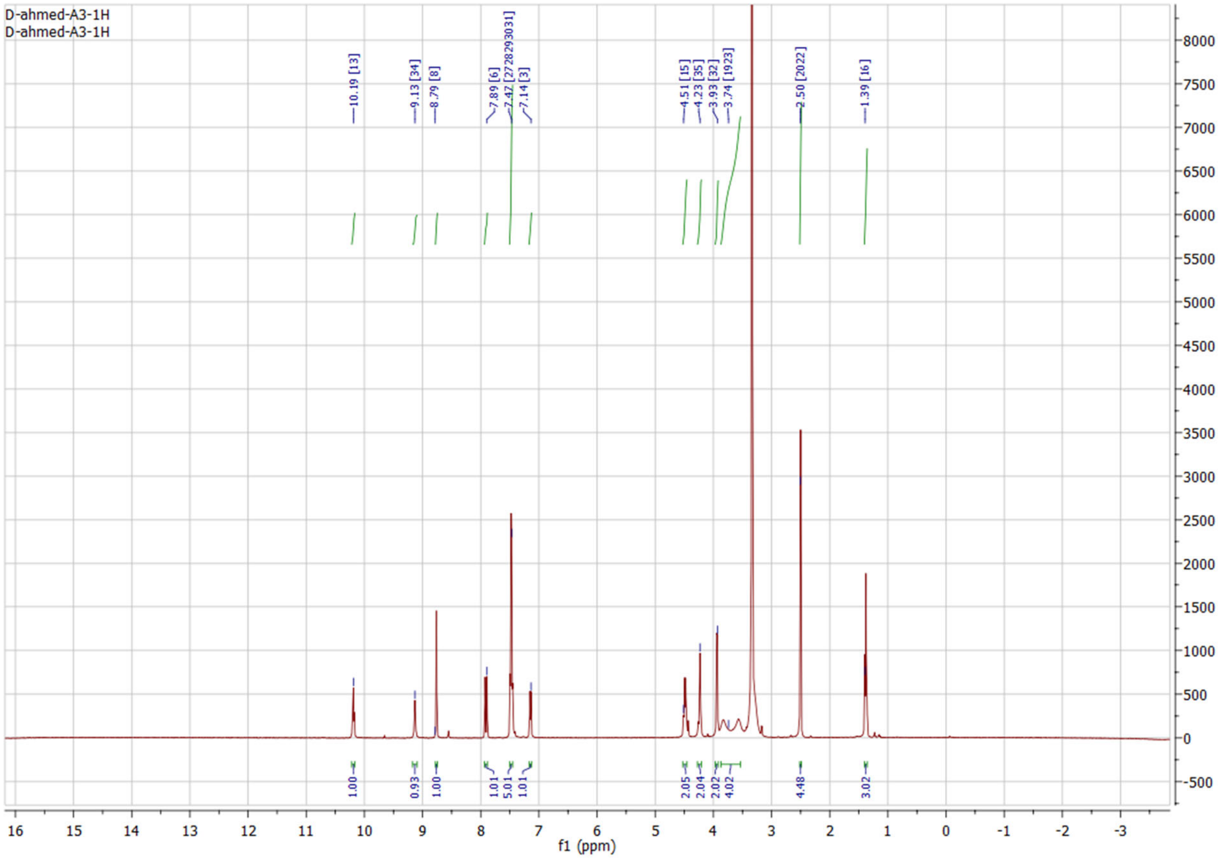

Figure S21: ^{1}H NMR of compound 5a

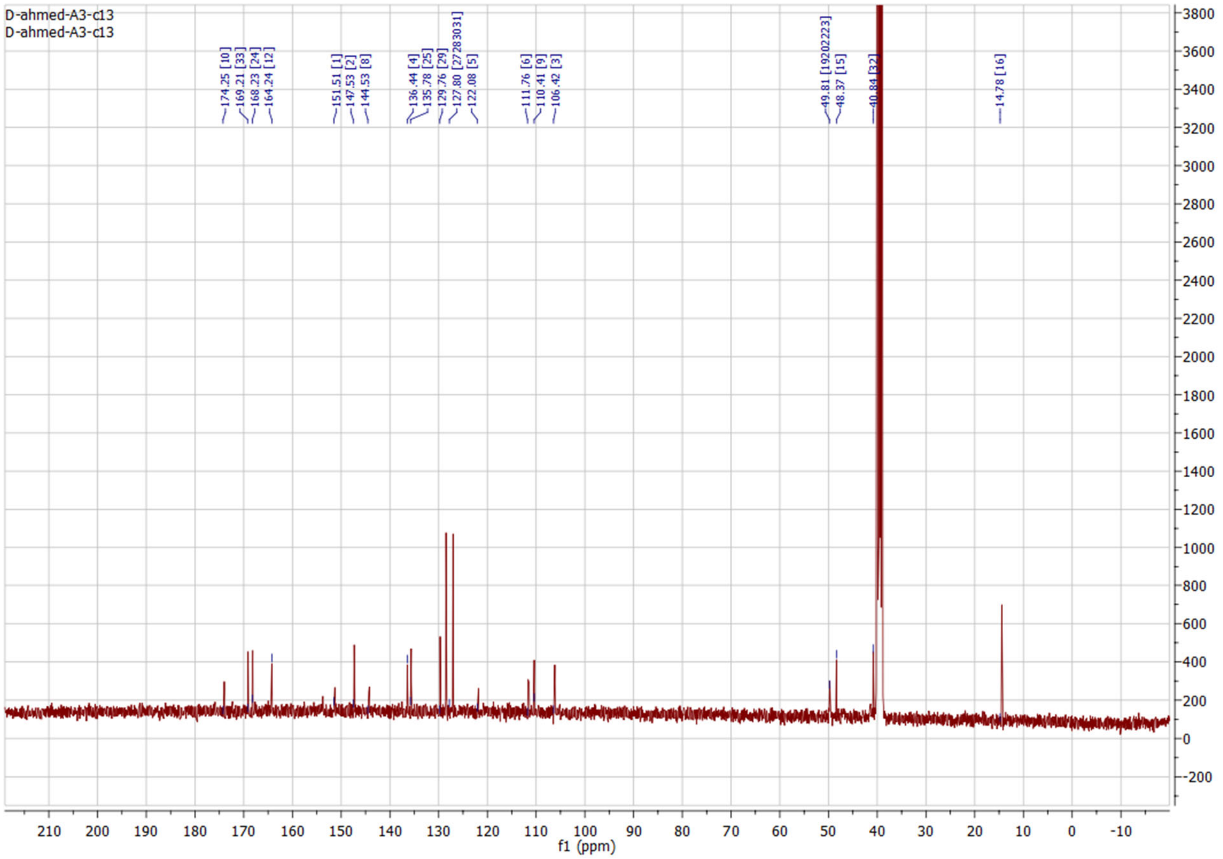

Figure S22: ^{13}C NMR of compound 5a

5b

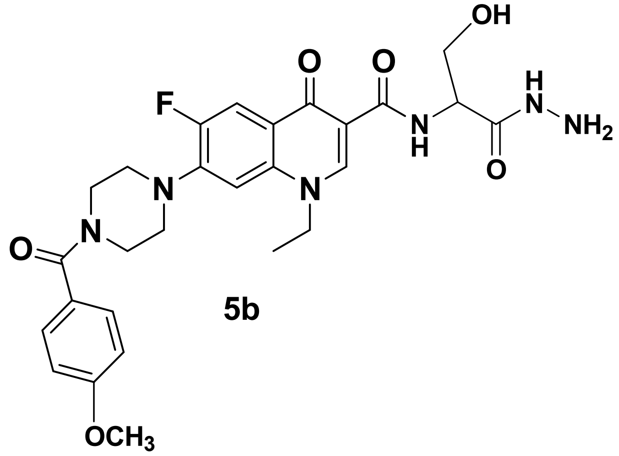

5b

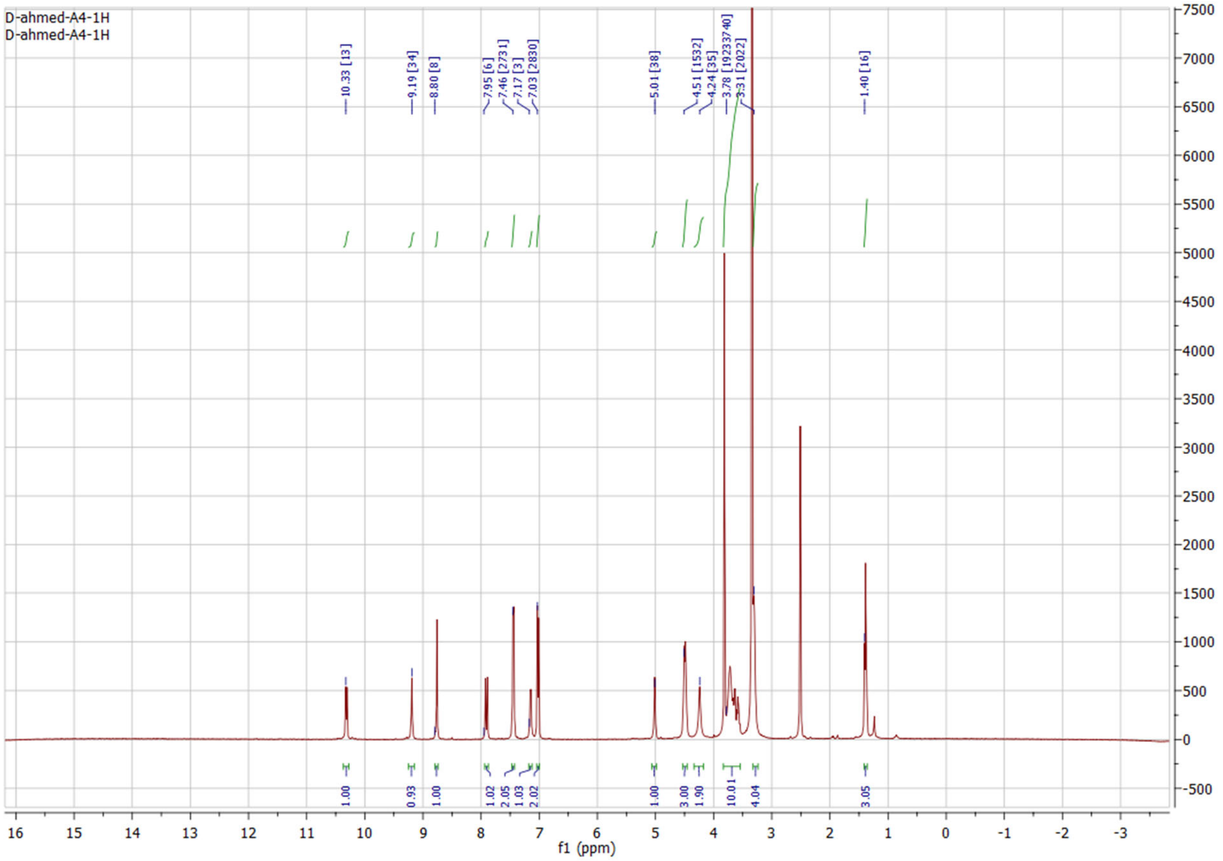

Figure S23: ^{1}H NMR of compound 5b

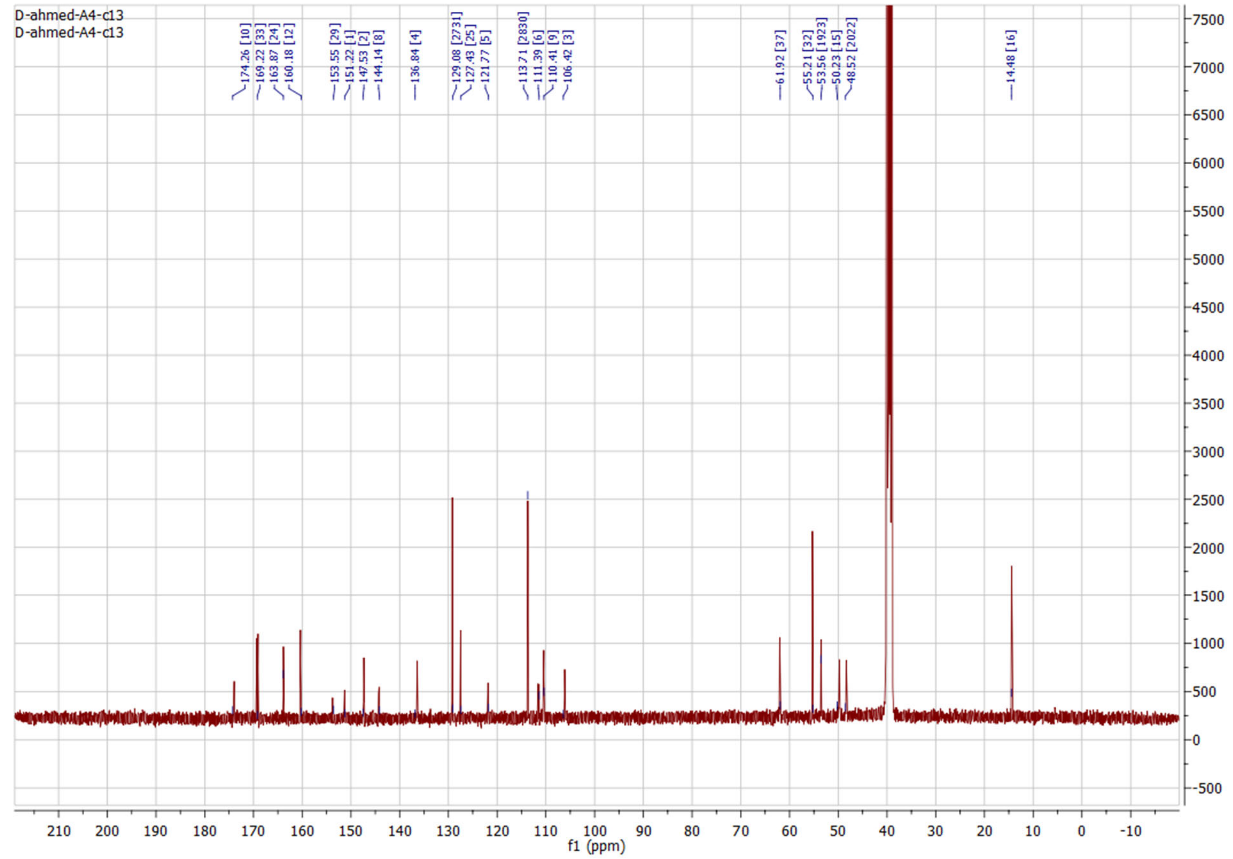

Figure S24: ^{13}C NMR of compound 5b

5c

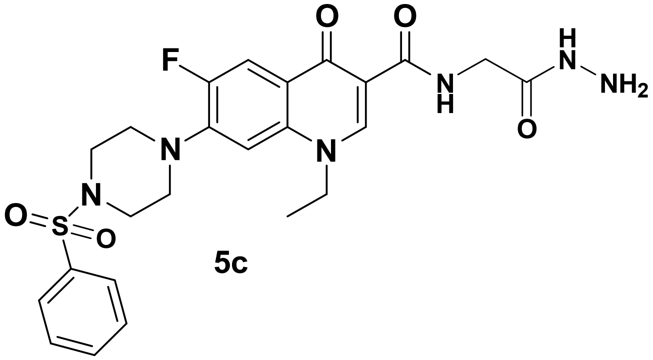

5c

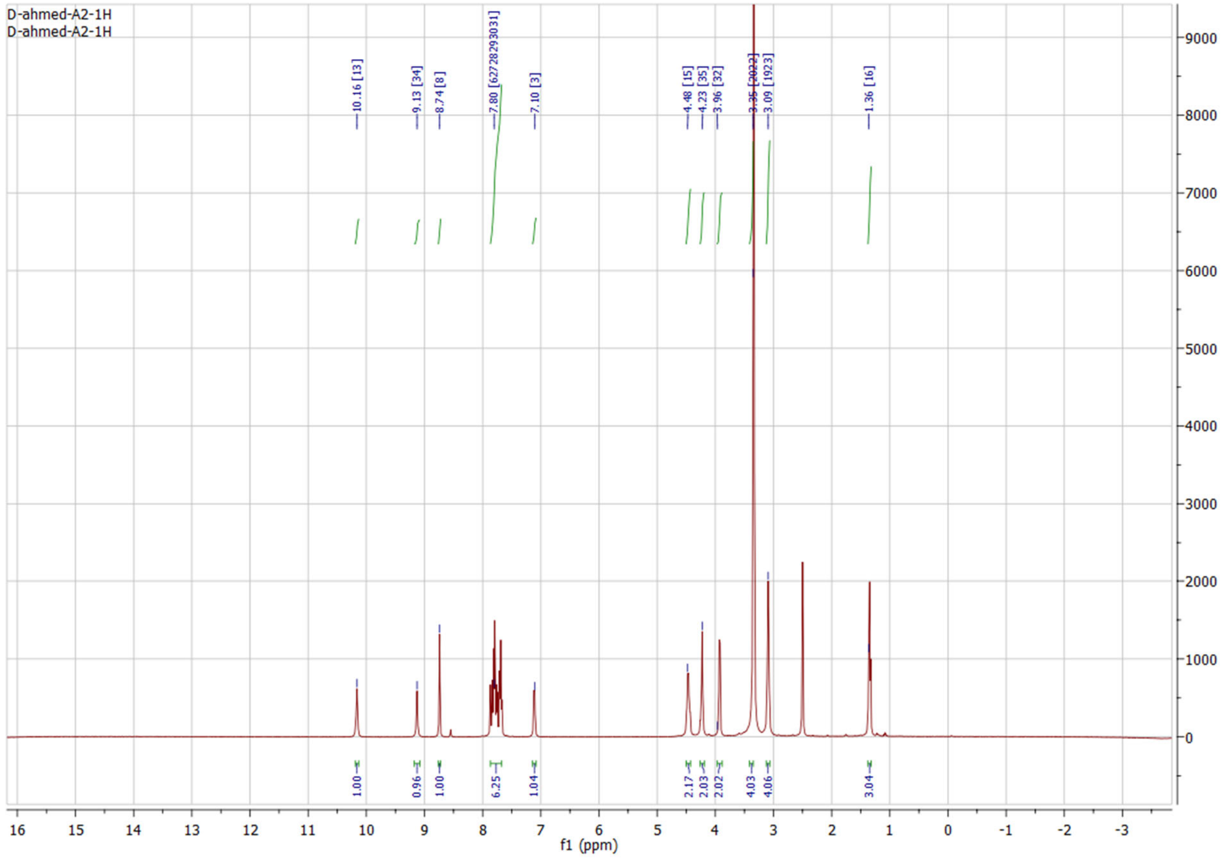

Figure S25: ^{1}H NMR of compound 5c

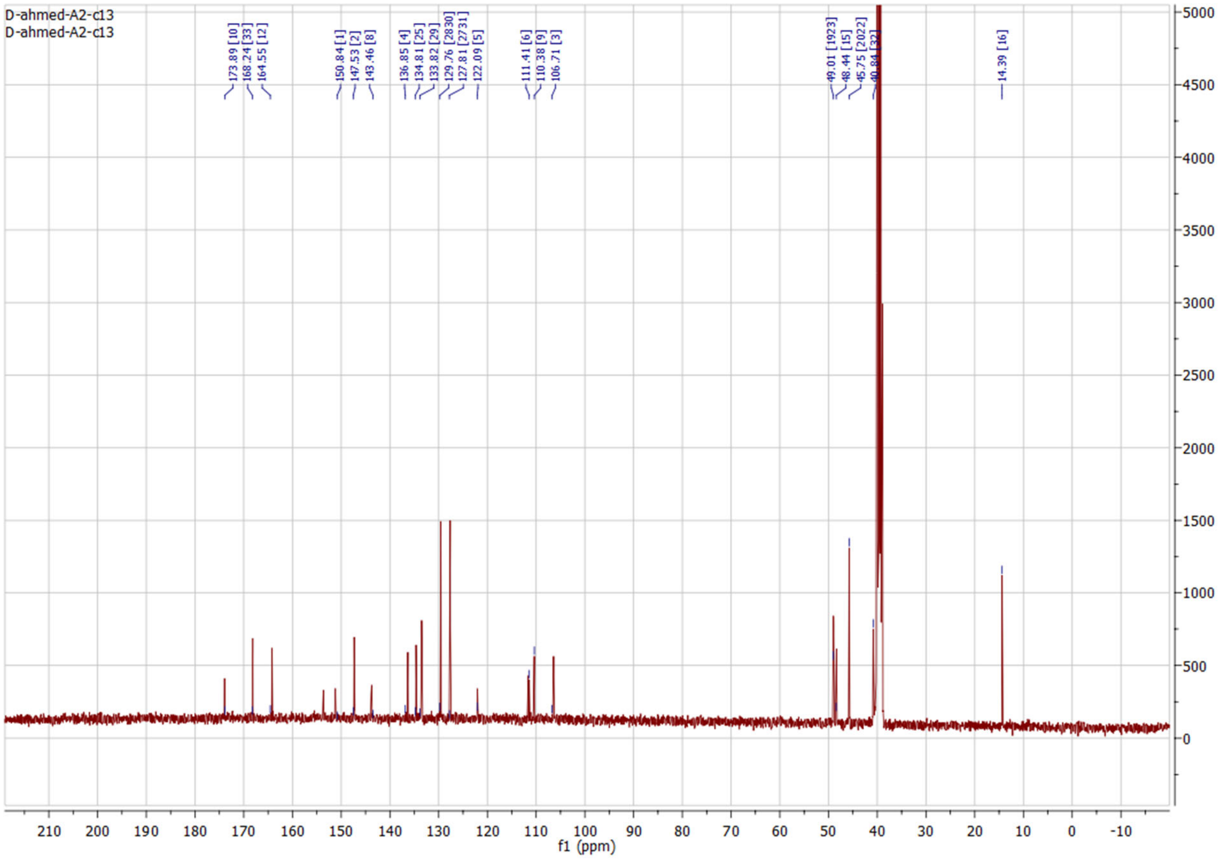

Figure S26: ^{13}C NMR of compound 5c

5d

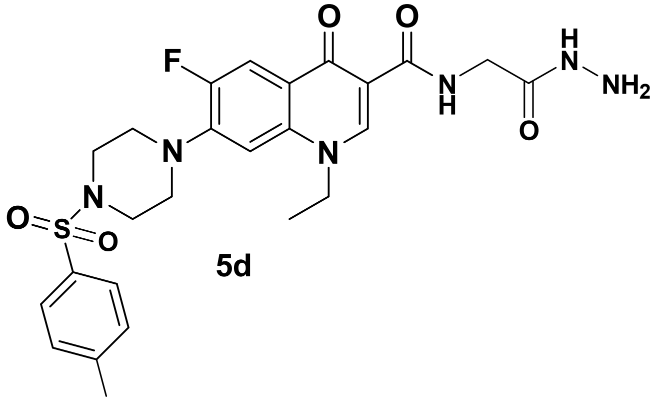

5d

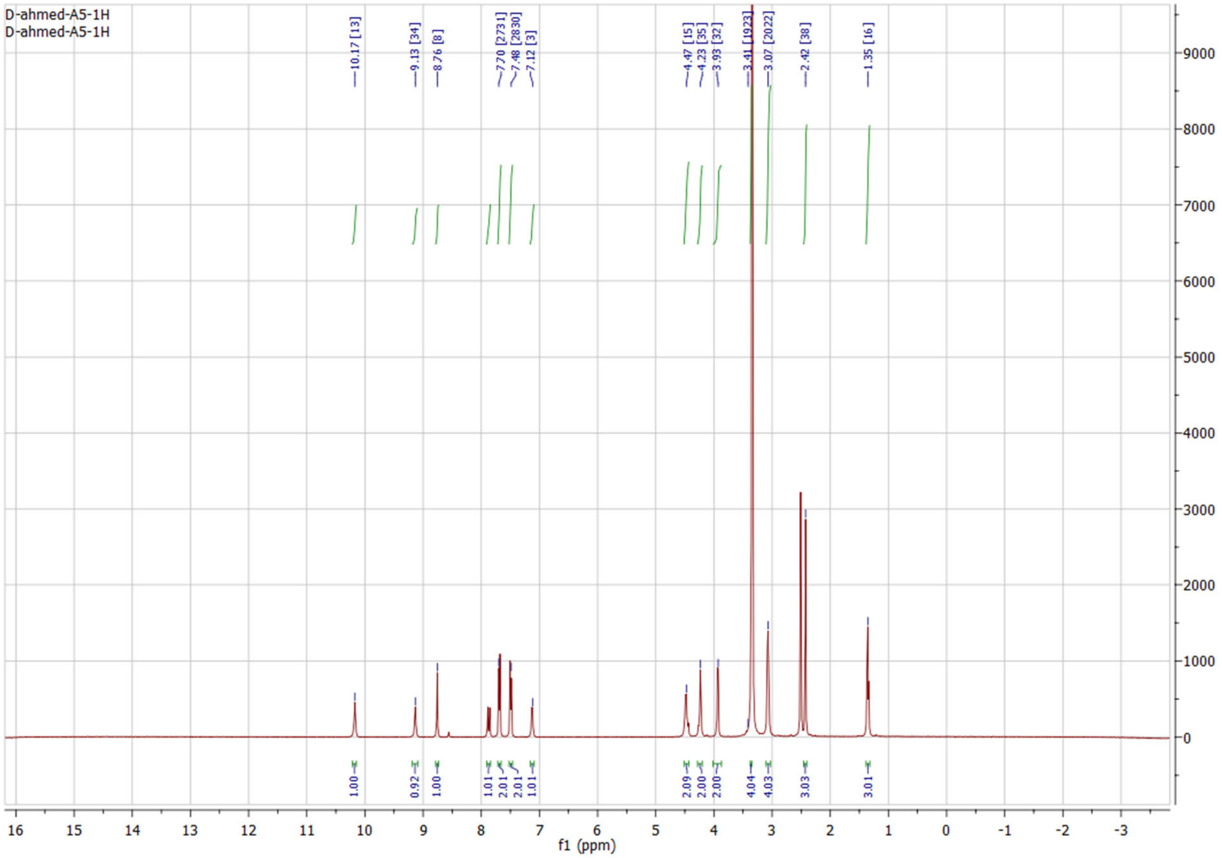

Figure S27: ^{1}H NMR of compound 5d

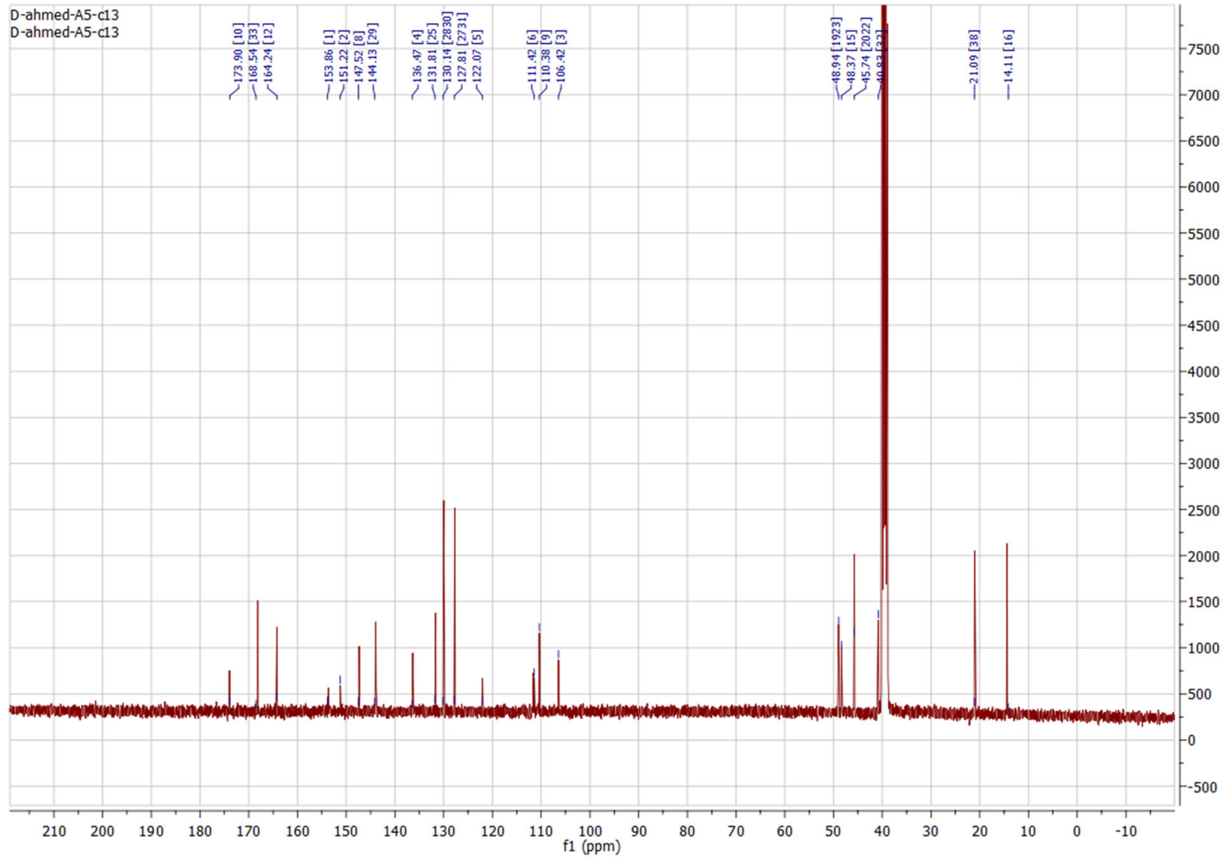

Figure S28: ^{13}C NMR of compound 5d

5e

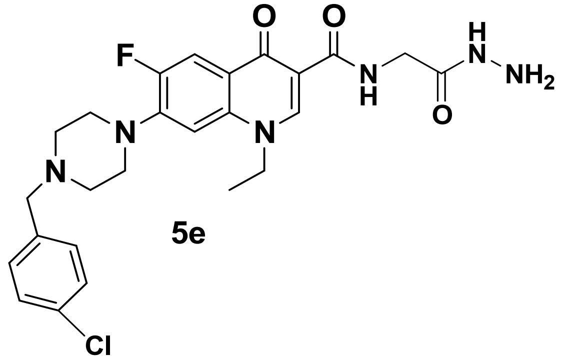

5e

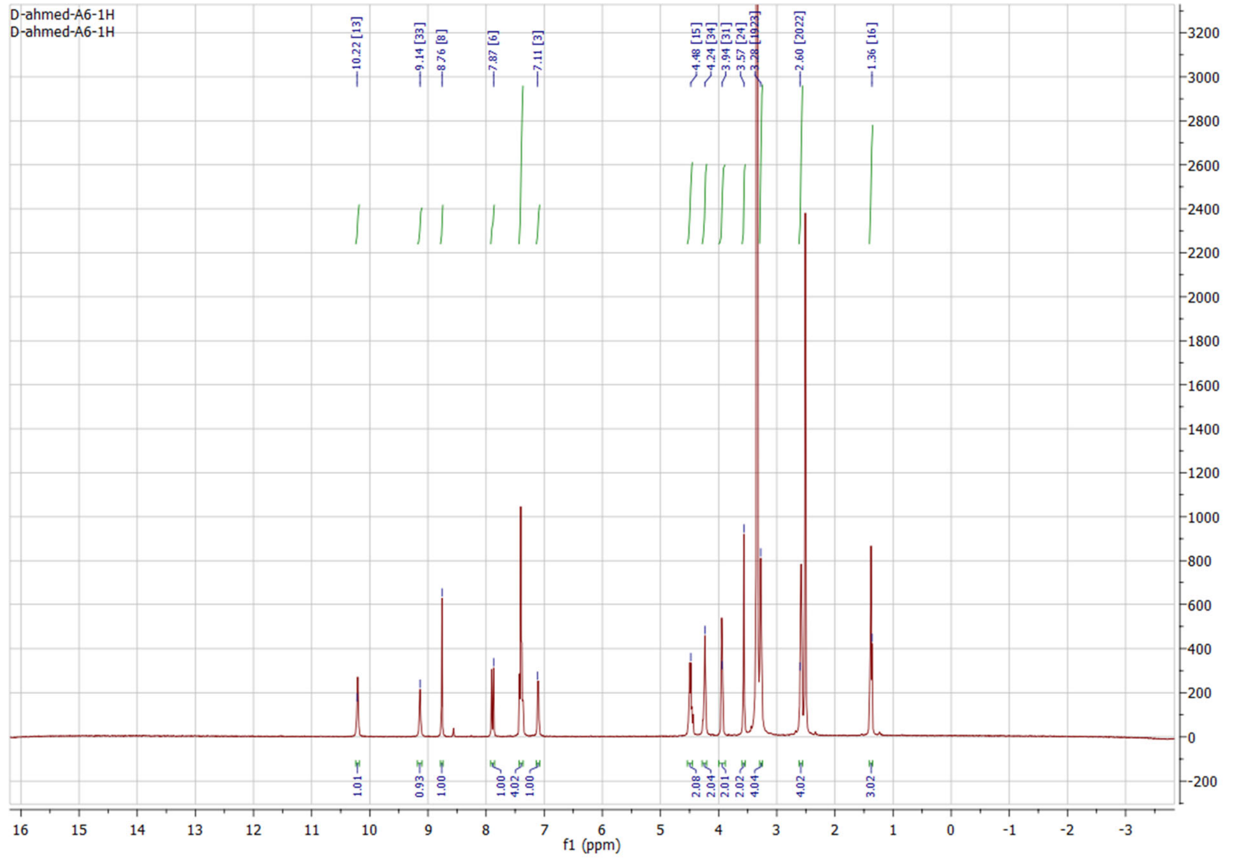

Figure S29: ^{1}H NMR of compound 5e

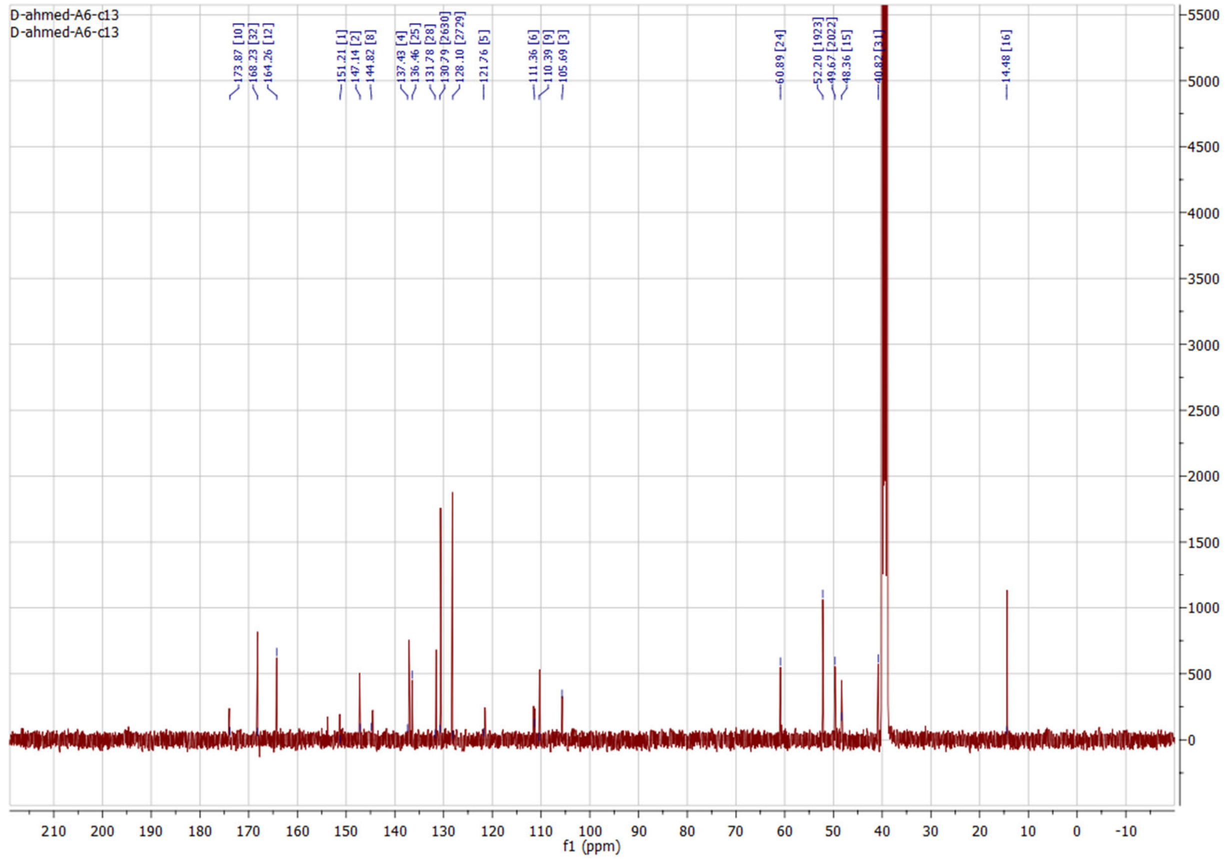

Figure S30: ^{13}C NMR of compound 5e

7a

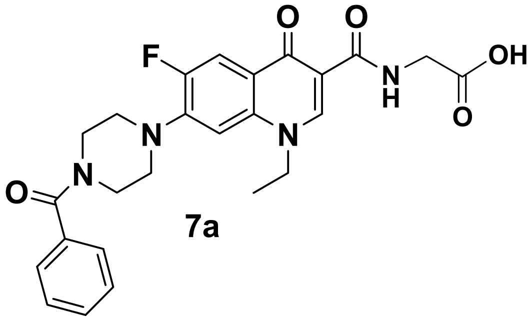

7a

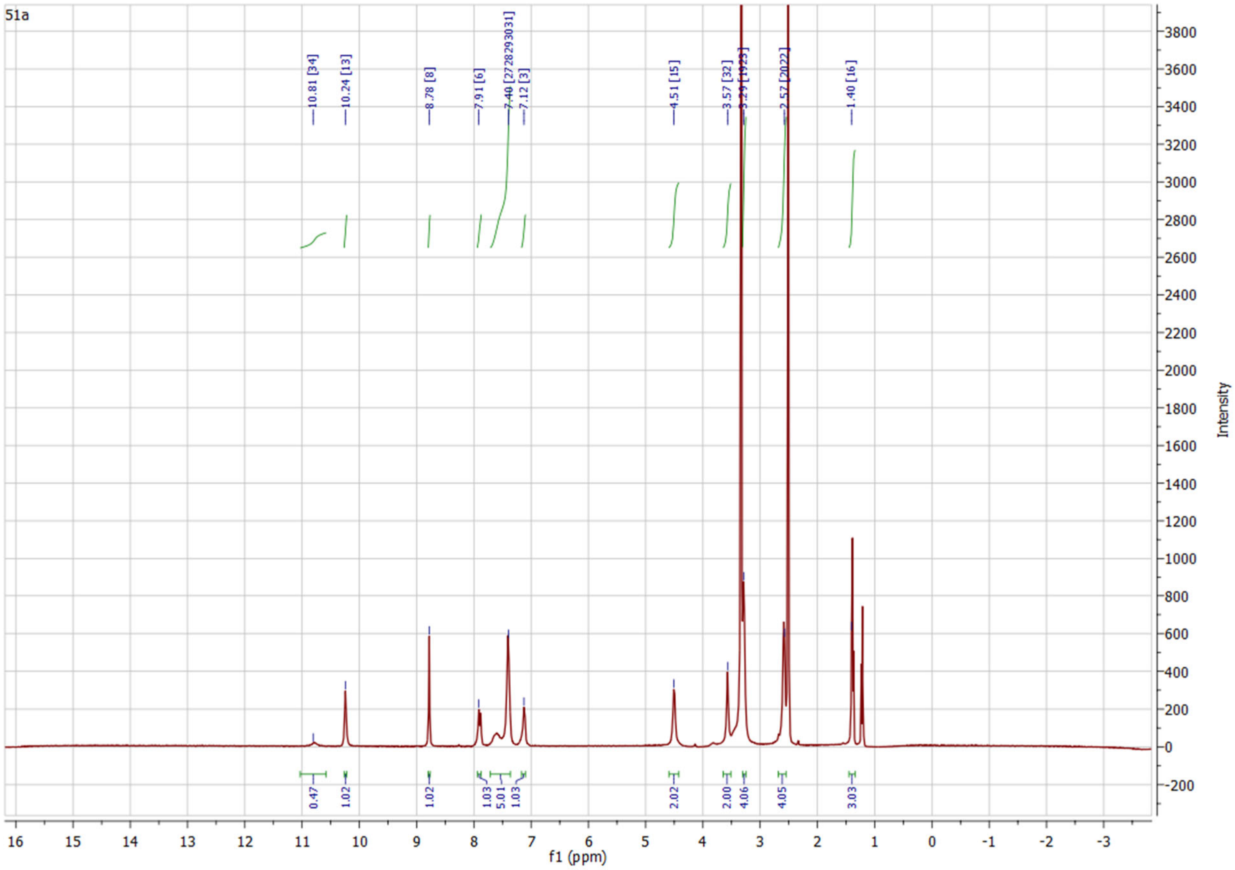

Figure S31: ^{1}H NMR of compound 7a

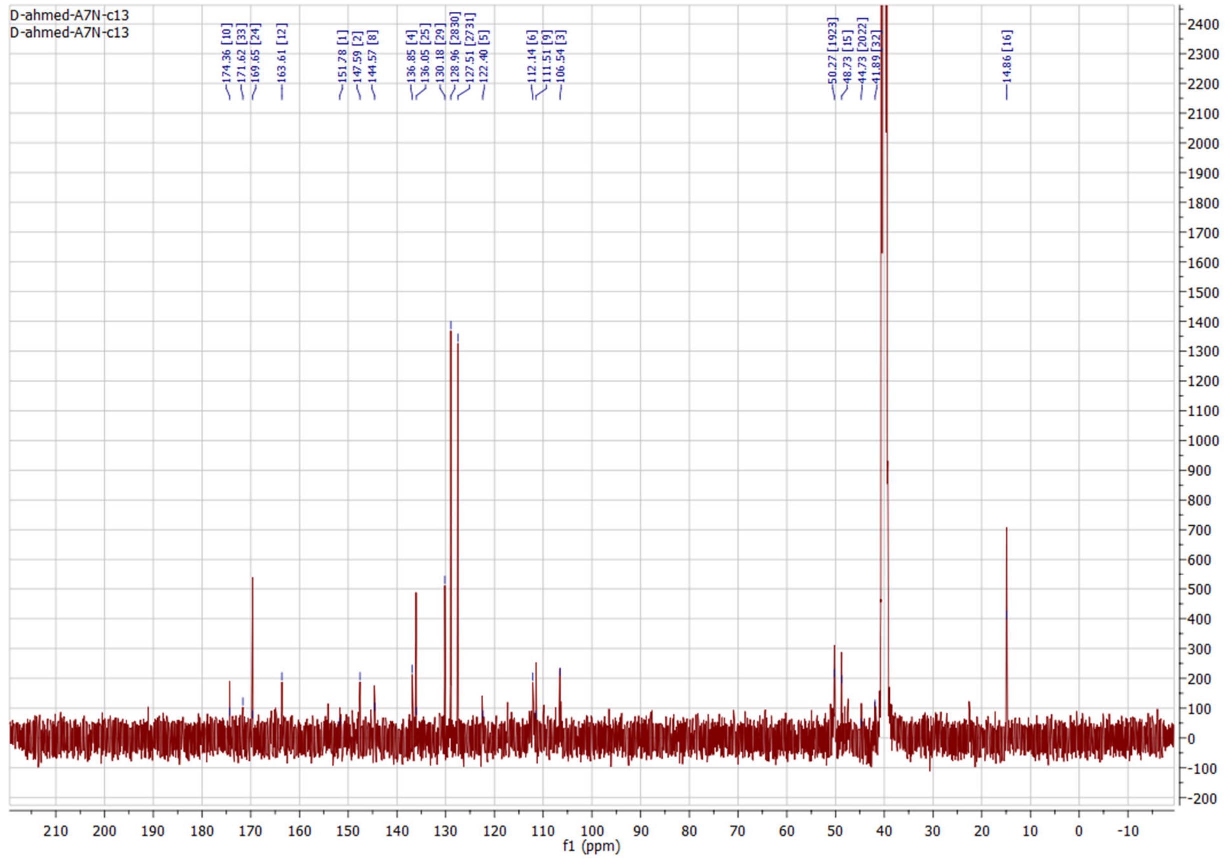

Figure S32: ^{13}C NMR of compound 7a

7b

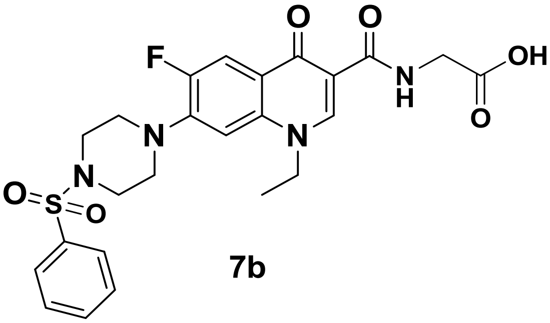

7b

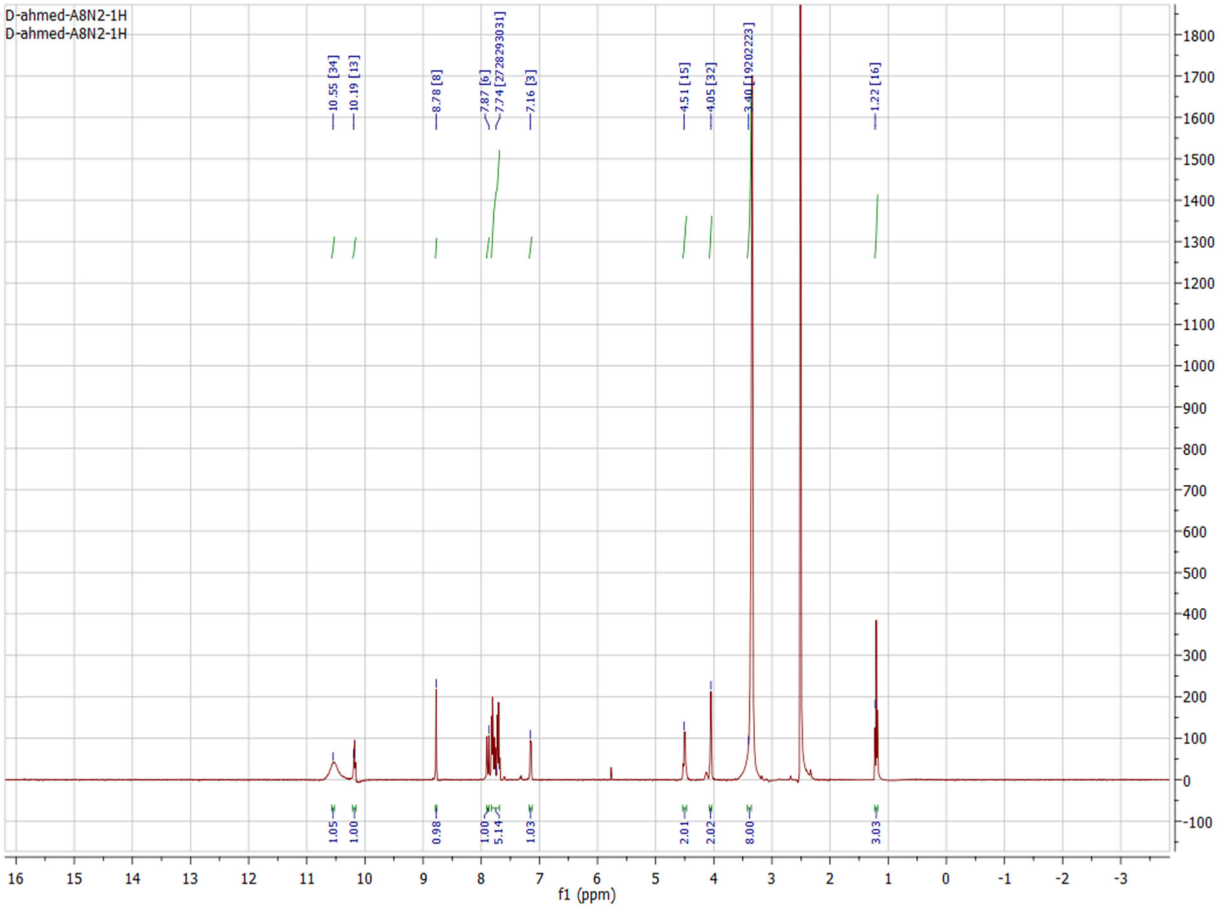

Figure S33: ^{1}H NMR of compound 7b

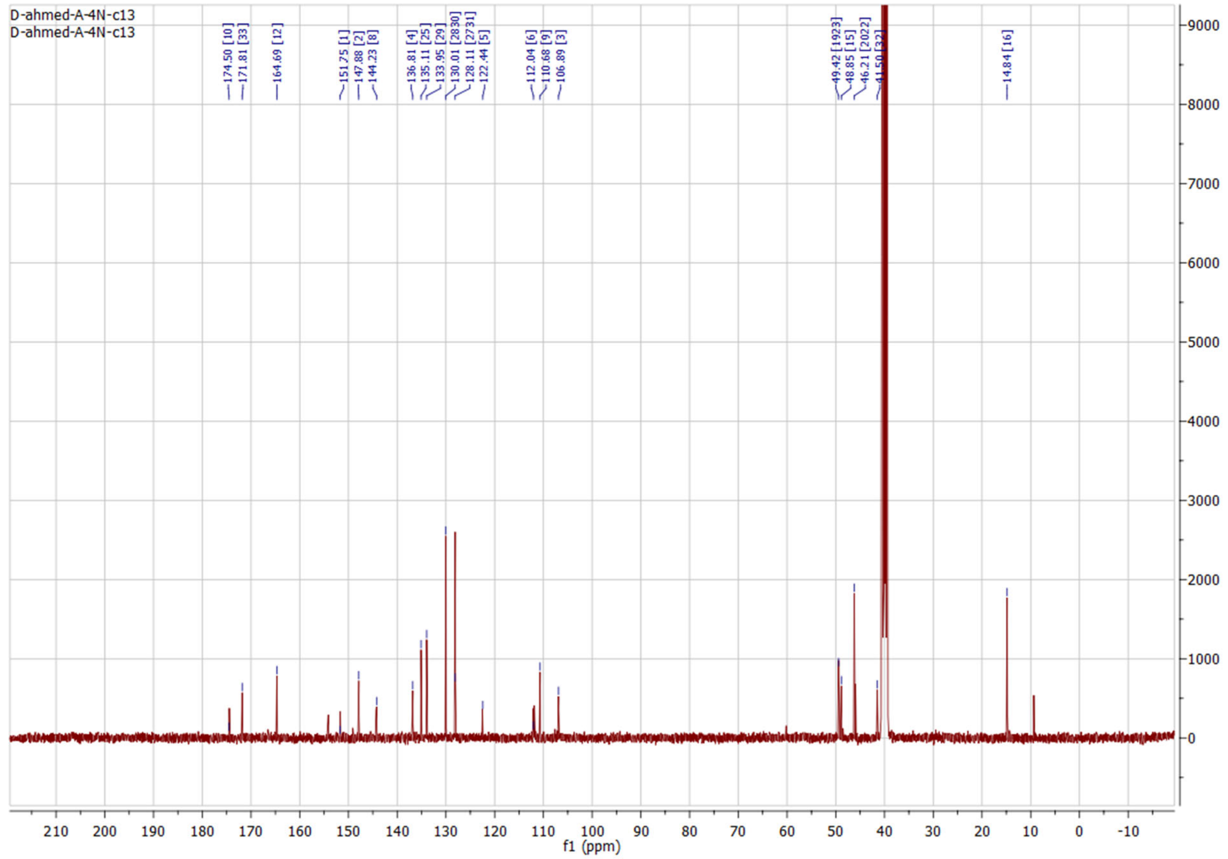

Figure S34: ^{13}C NMR of compound 7b

7c

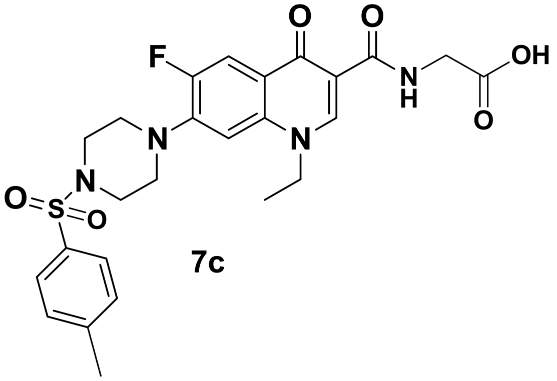

7c

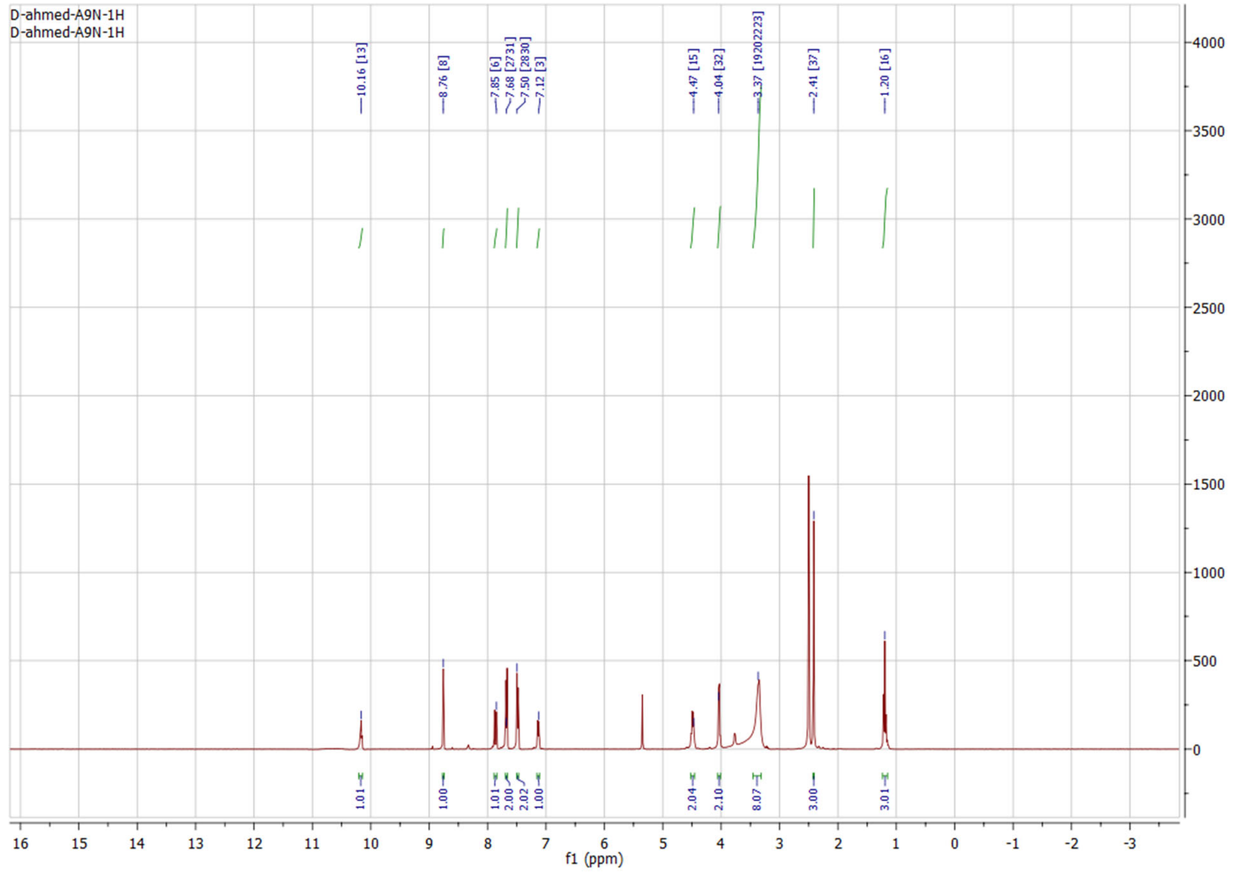

Figure S35: ^{1}H NMR of compound 7c

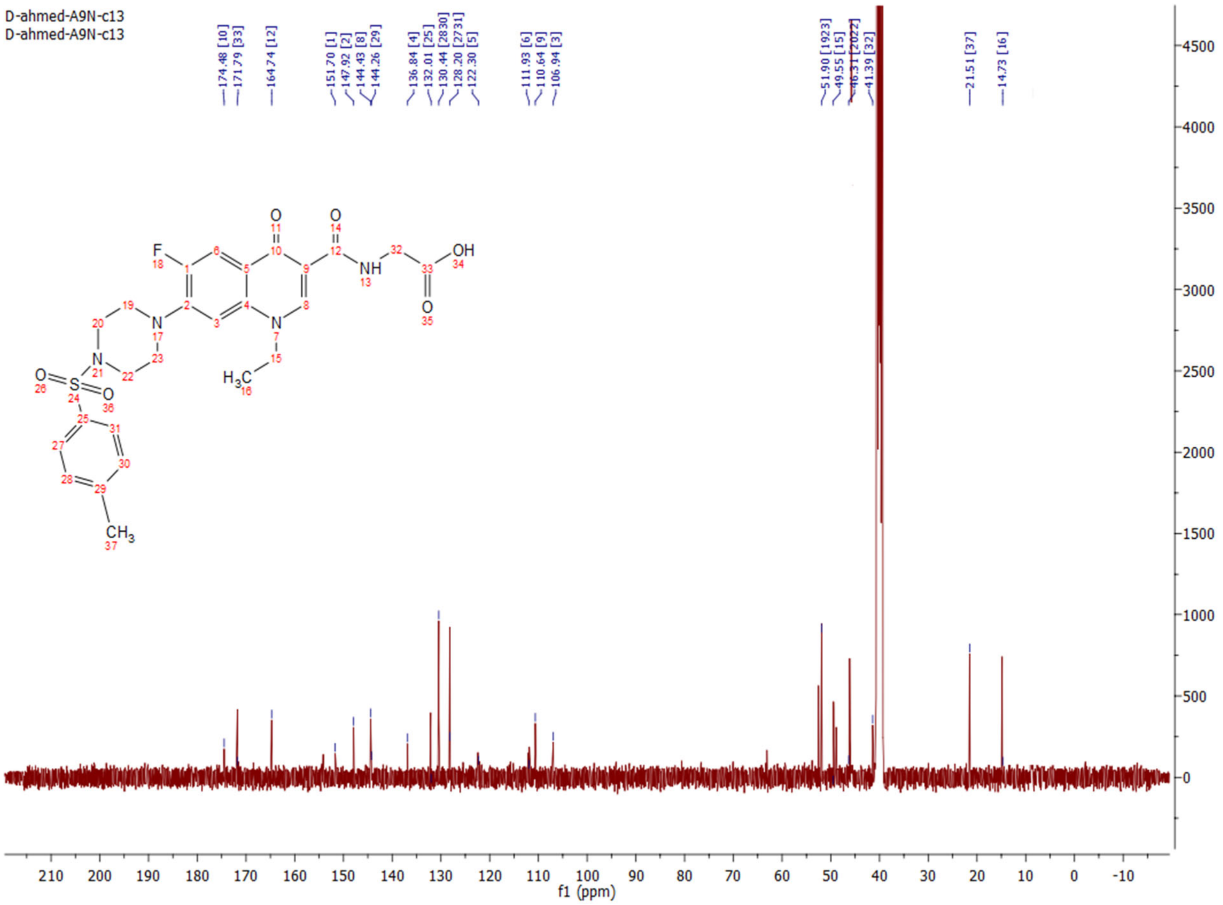

Figure S36: ^{13}C NMR of compound 7c

7d

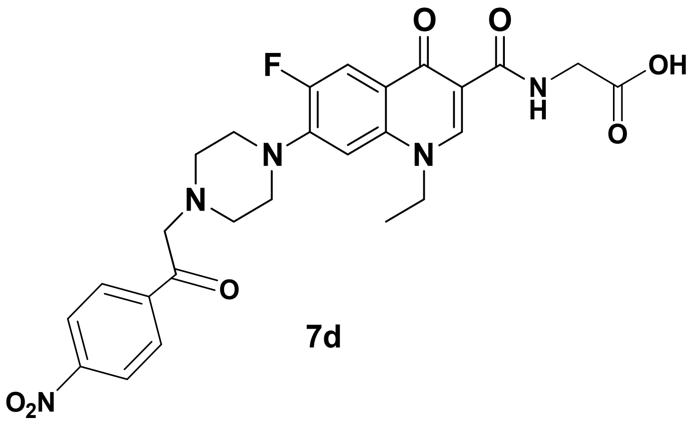

7d

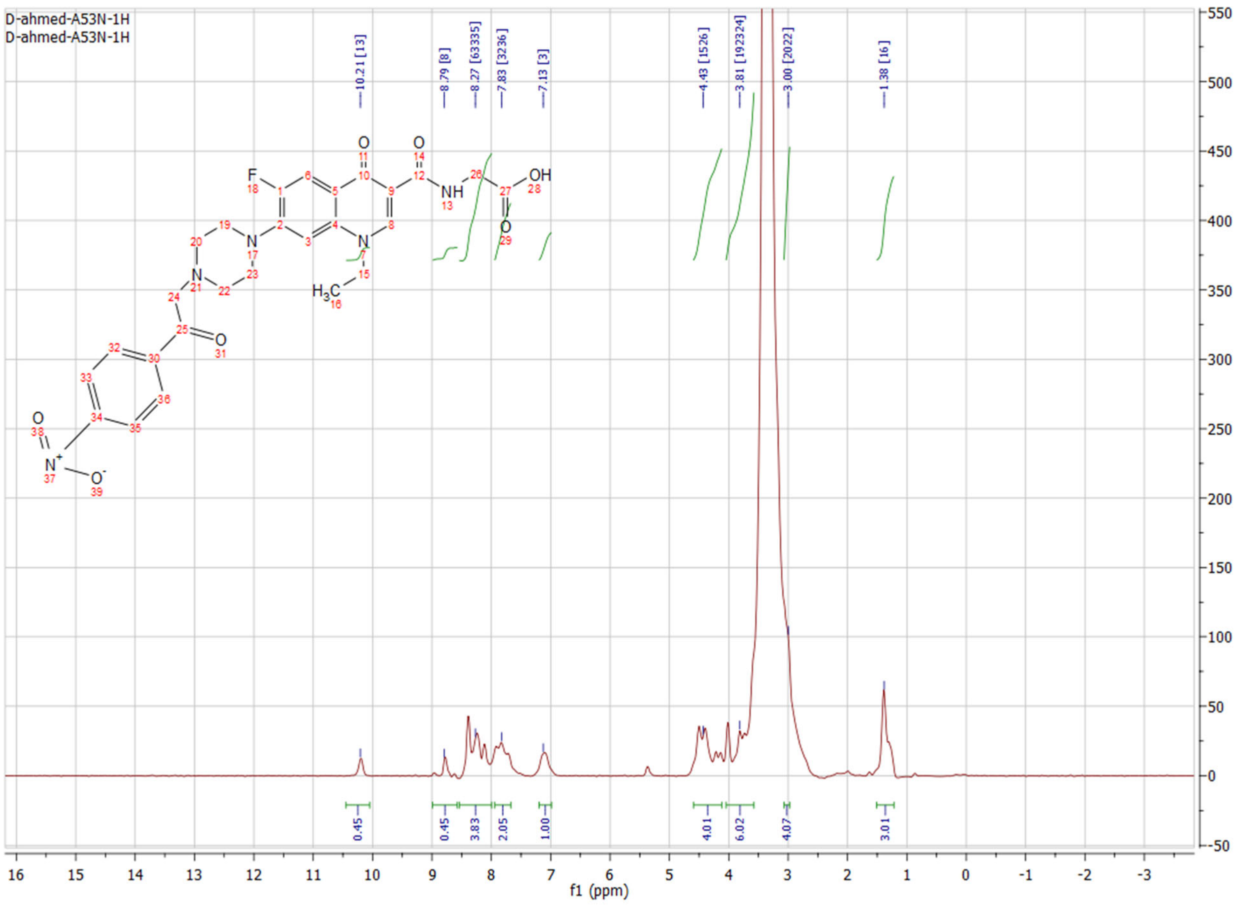

Figure S37: ^{1}H NMR of compound 7d

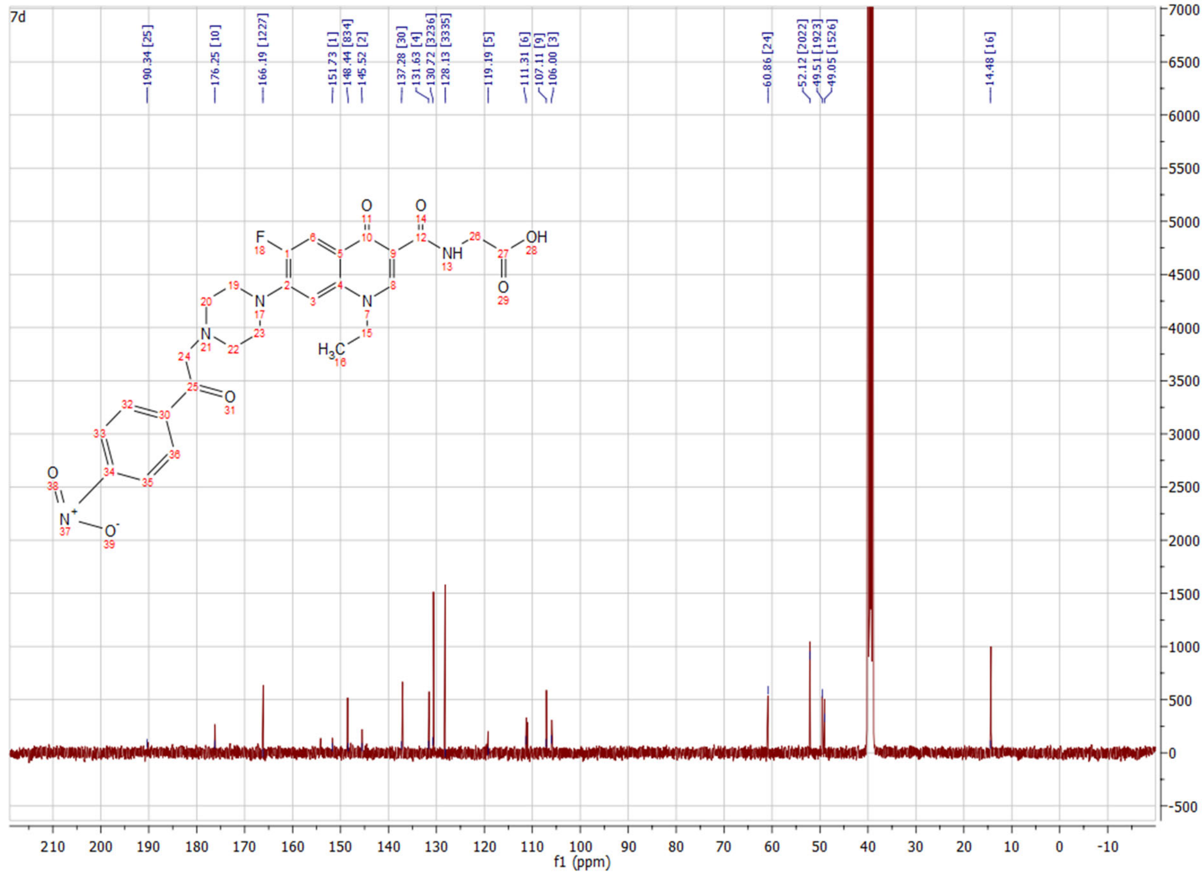

Figure S38: ^{13}C NMR of compound 7d

7e

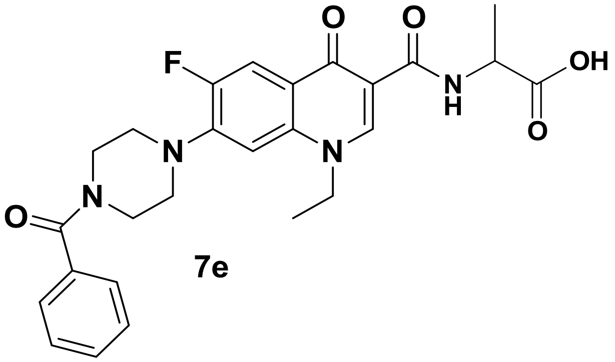

7e

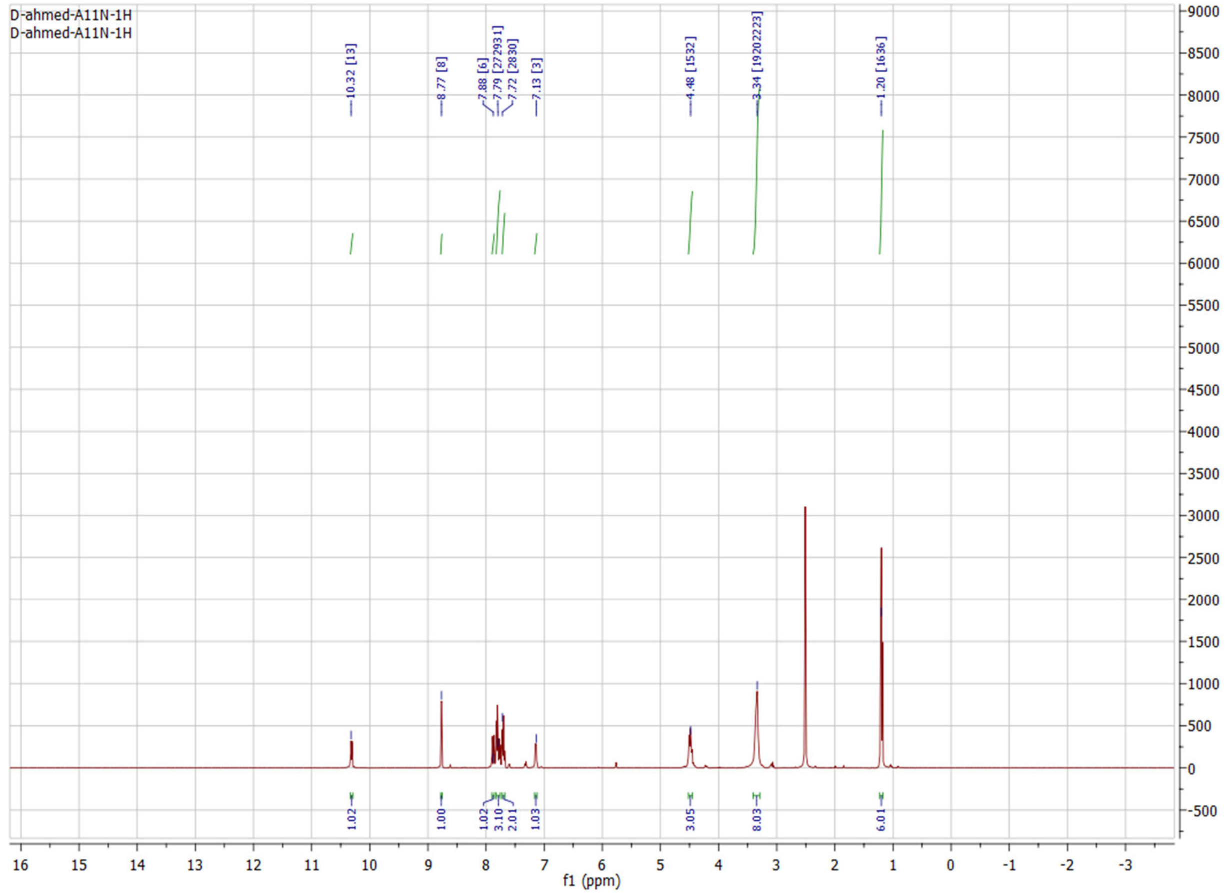

Figure S39: ^{1}H NMR of compound 7e

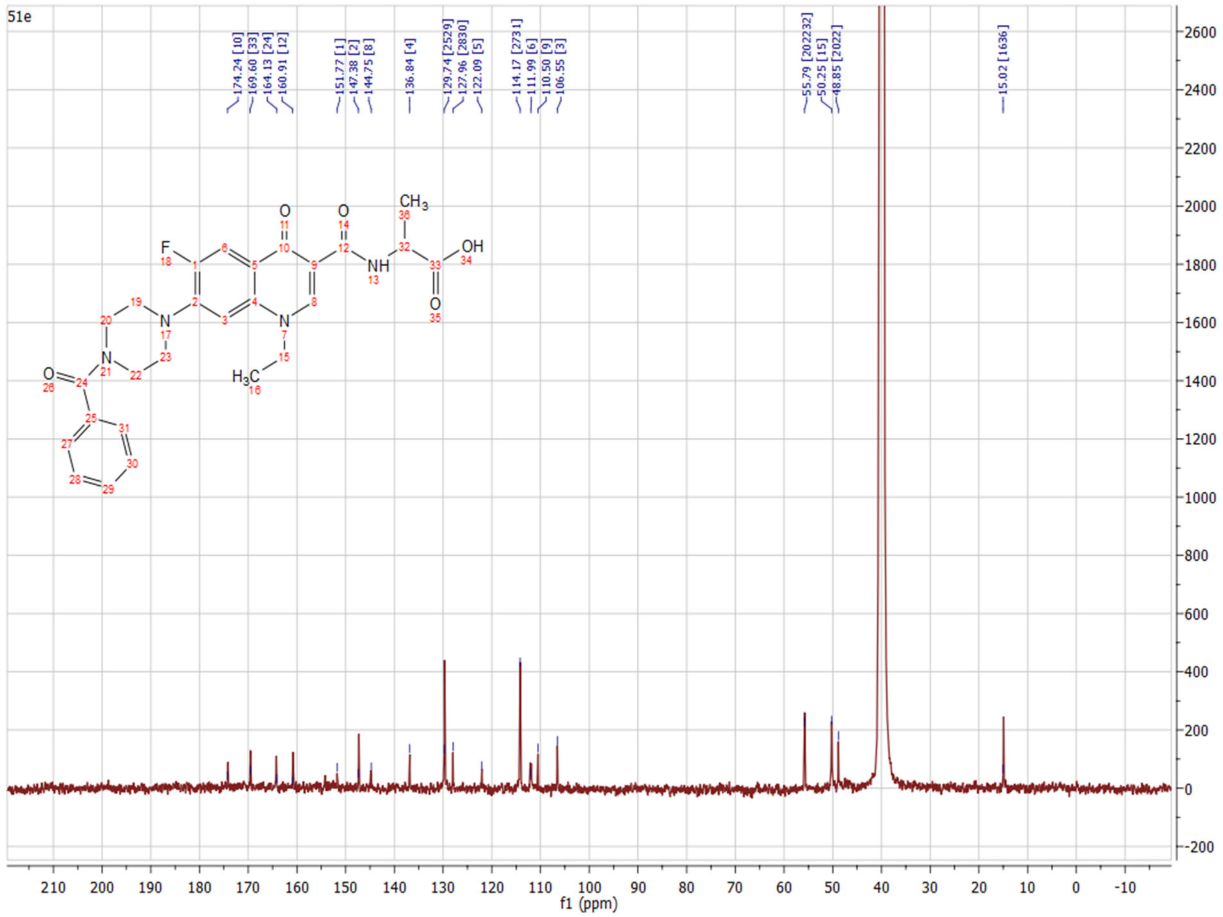

Figure S40: ^{13}C NMR of compound 7e

7f

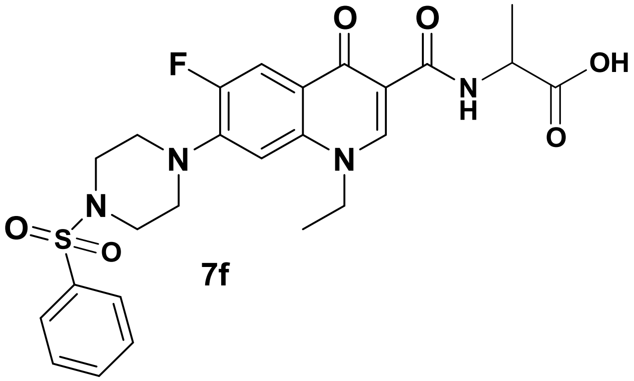
7f

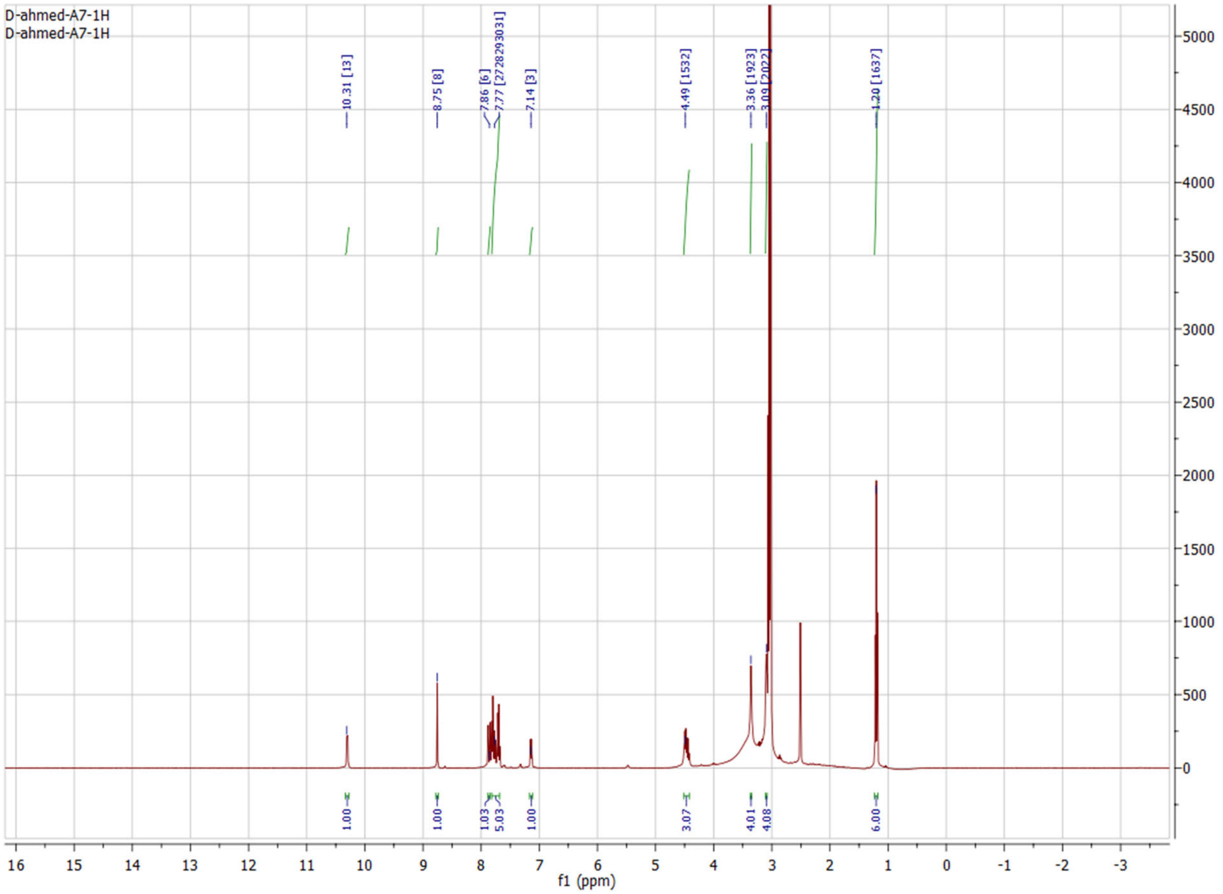

Figure S41: ^{1}H NMR of compound 7f

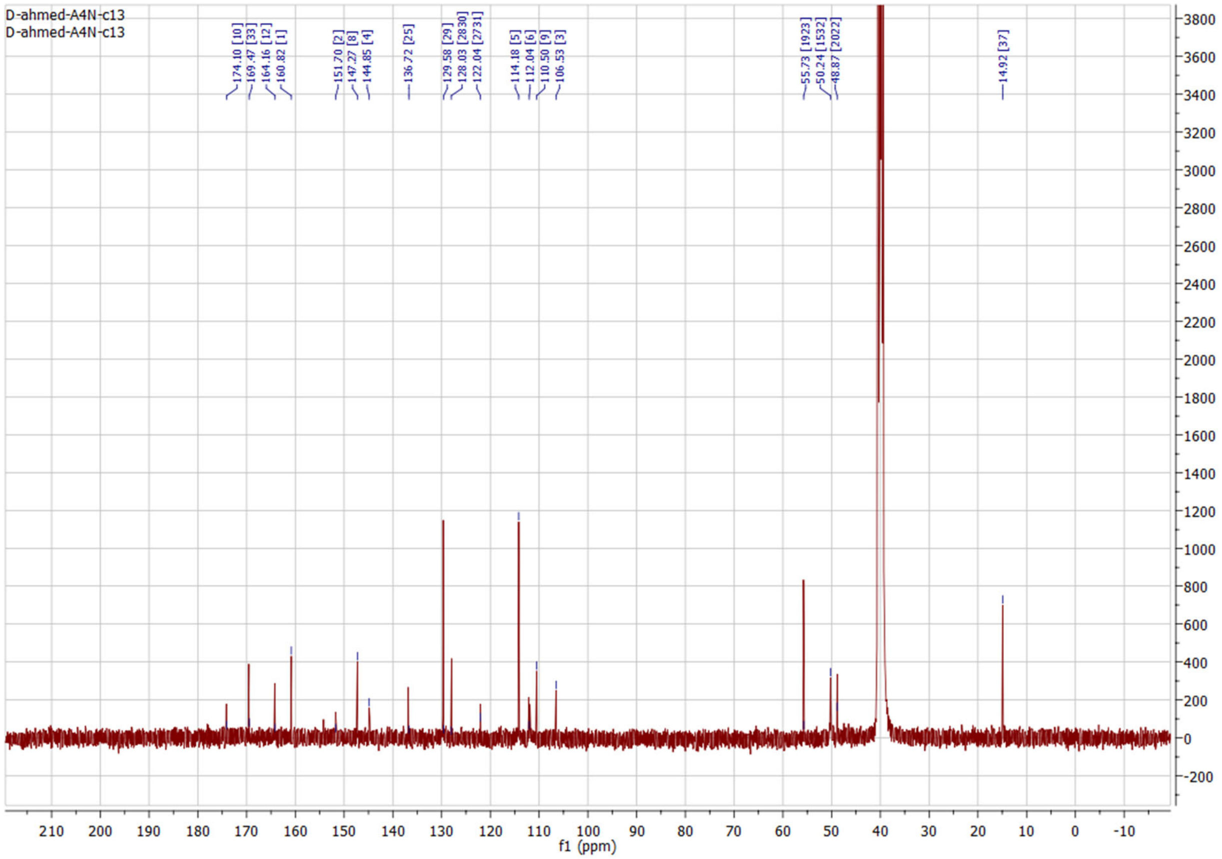

Figure S42: ^{13}C NMR of compound 7f

7g

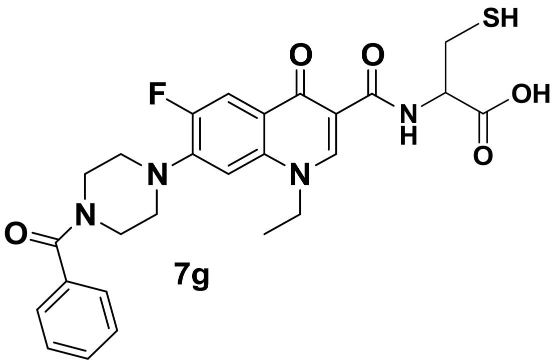

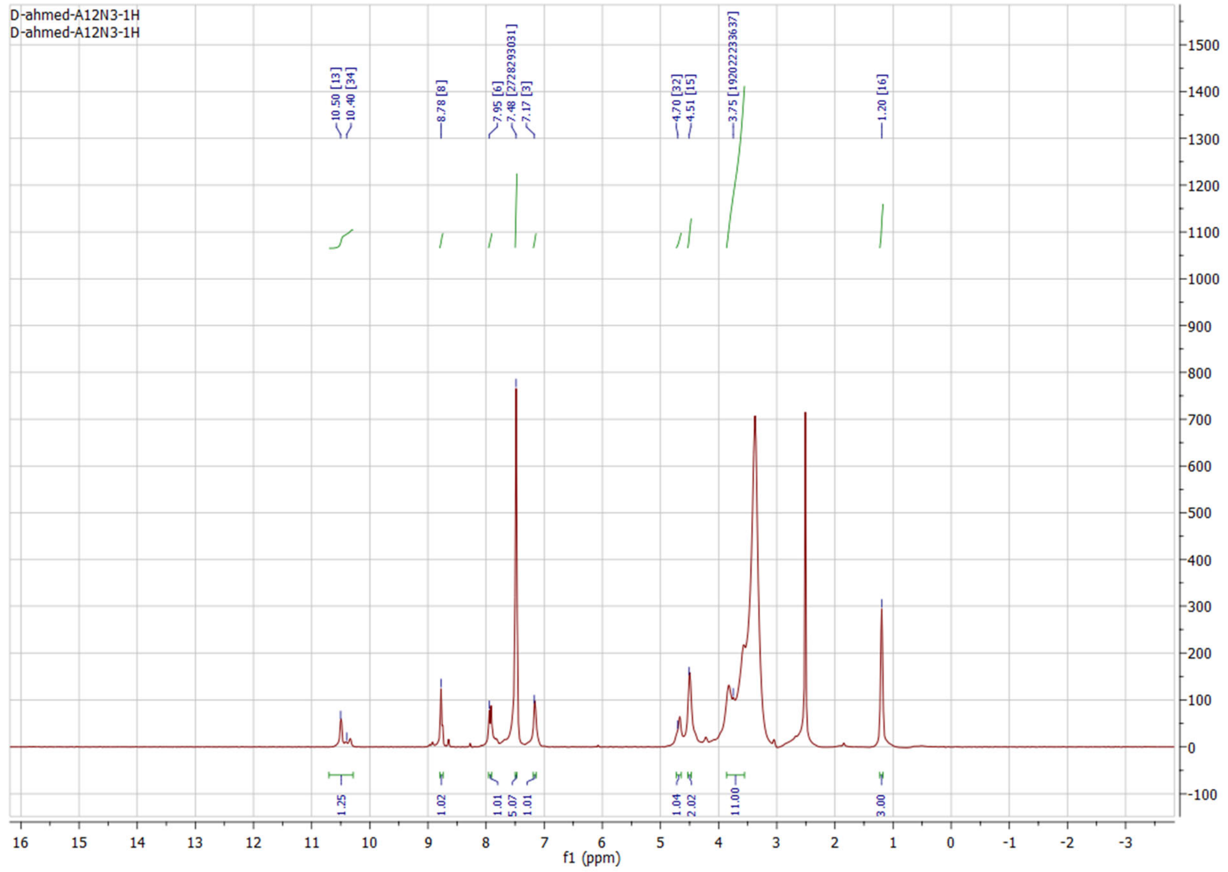

Figure S43: ^{1}H NMR of compound 7g

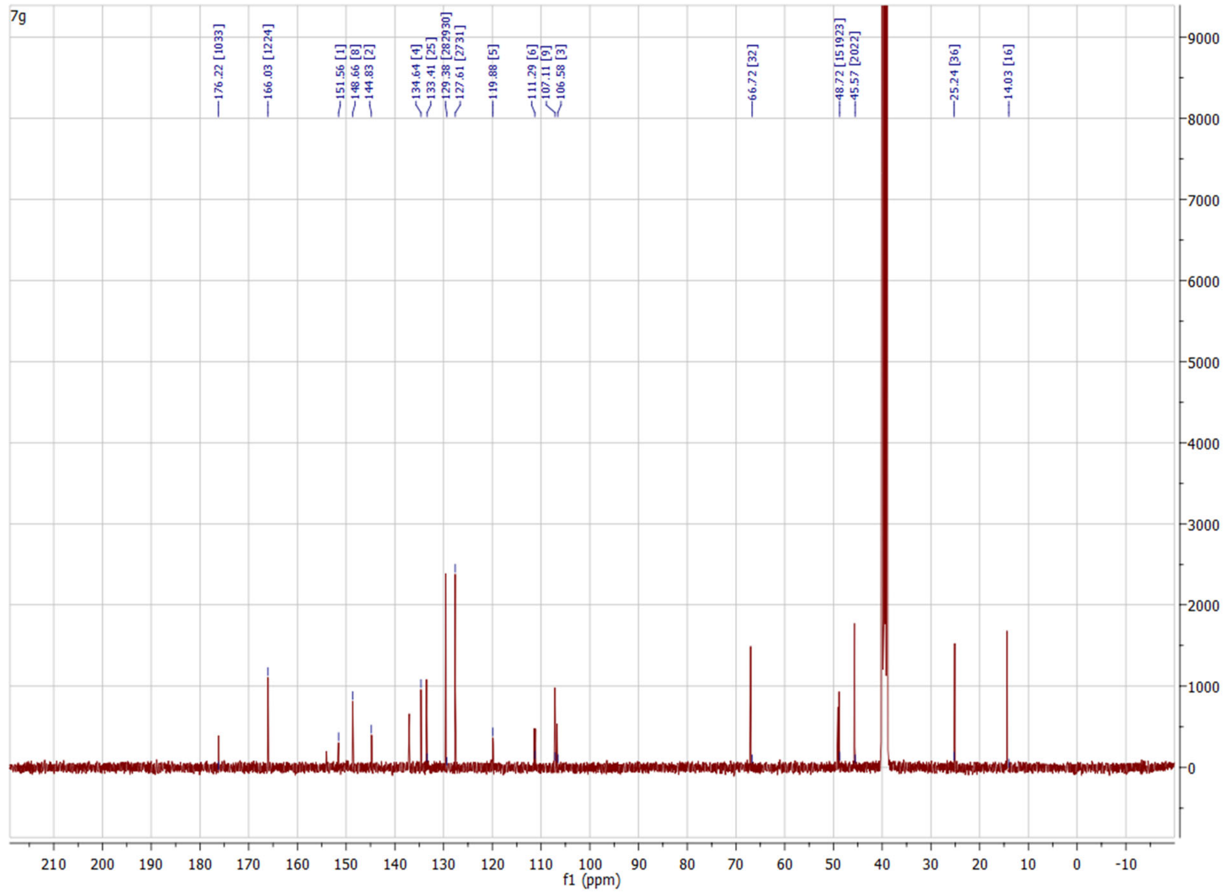

Figure S44: ^{13}C NMR of compound 7g

7h

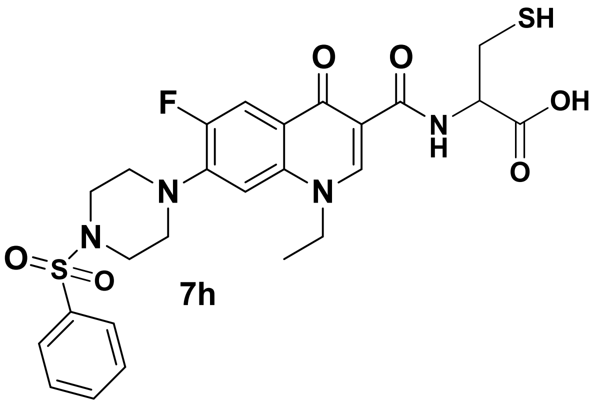

7h

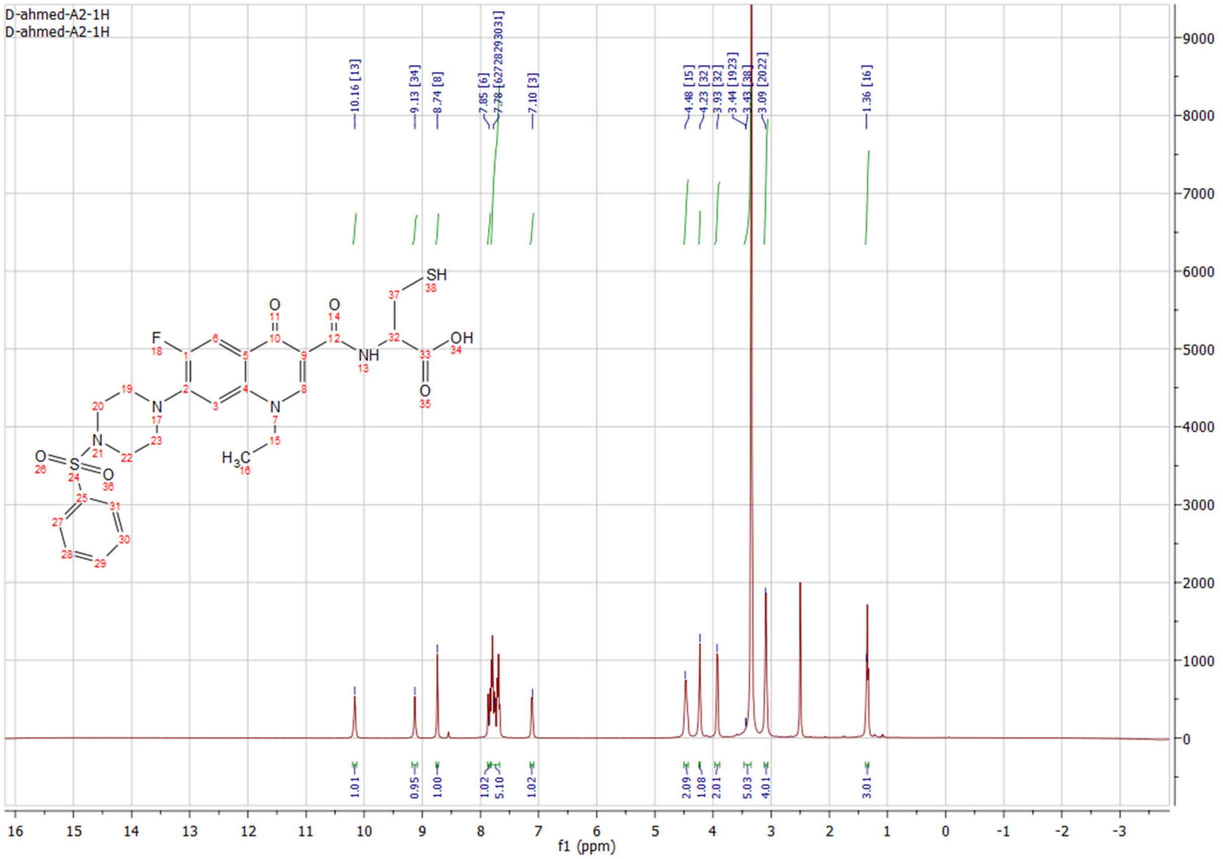

Figure S45: ^{1}H NMR of compound 7h

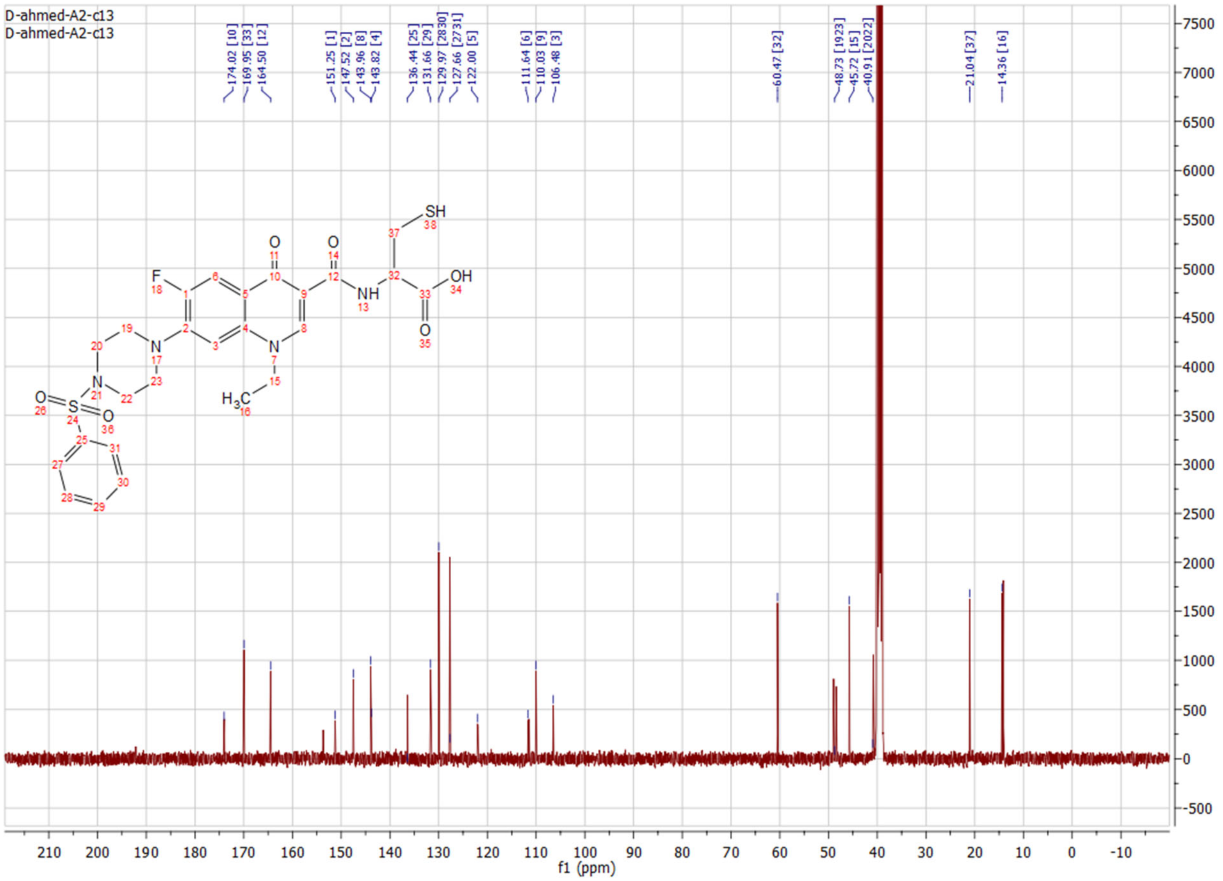

Figure S46: ^{13}C NMR of compound 7h

7i

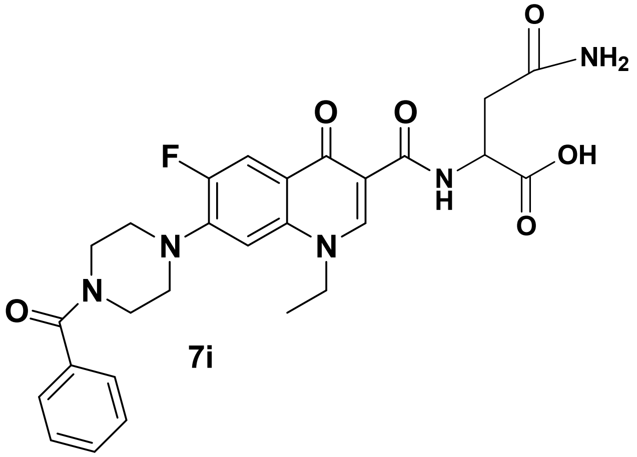

7i

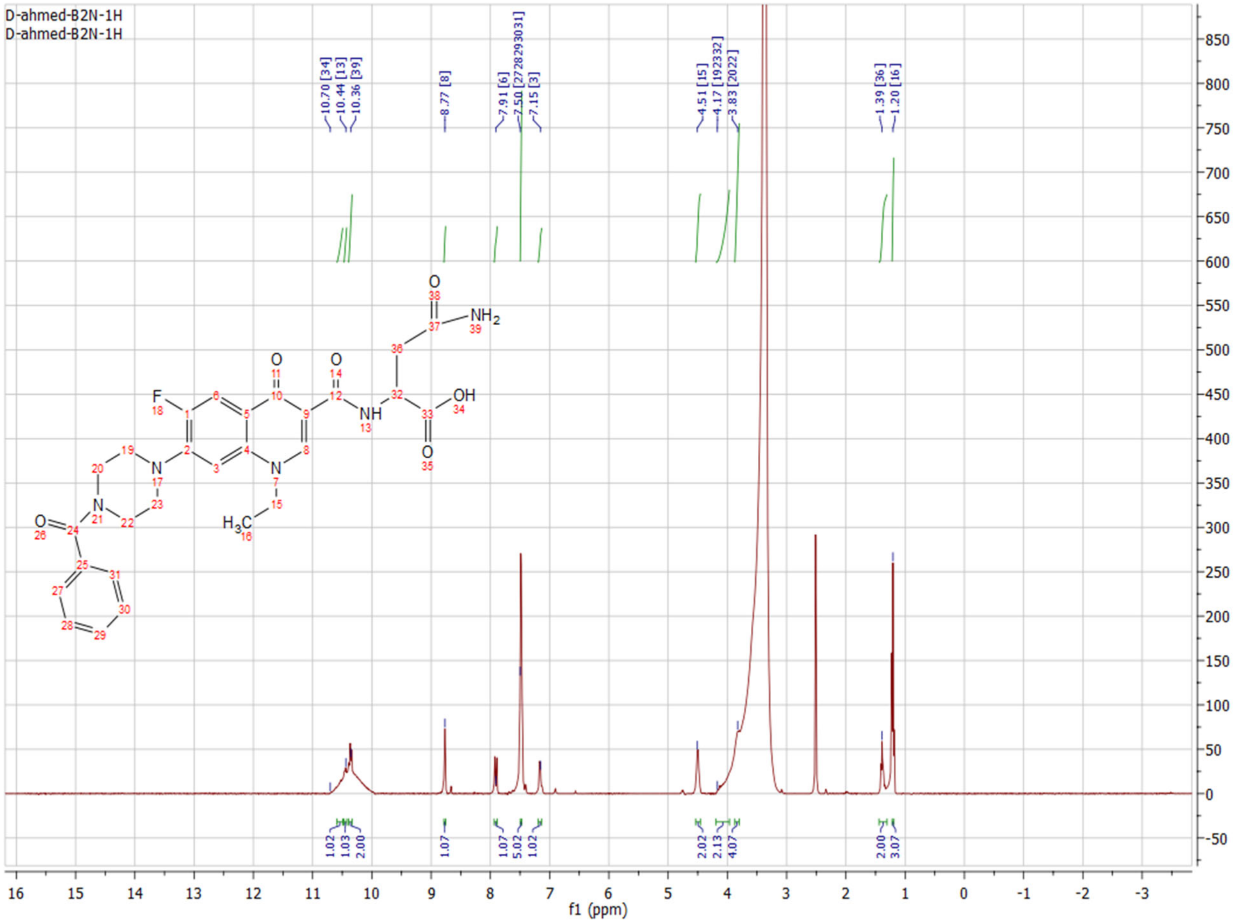

Figure S47: ^{1}H NMR of compound 7i

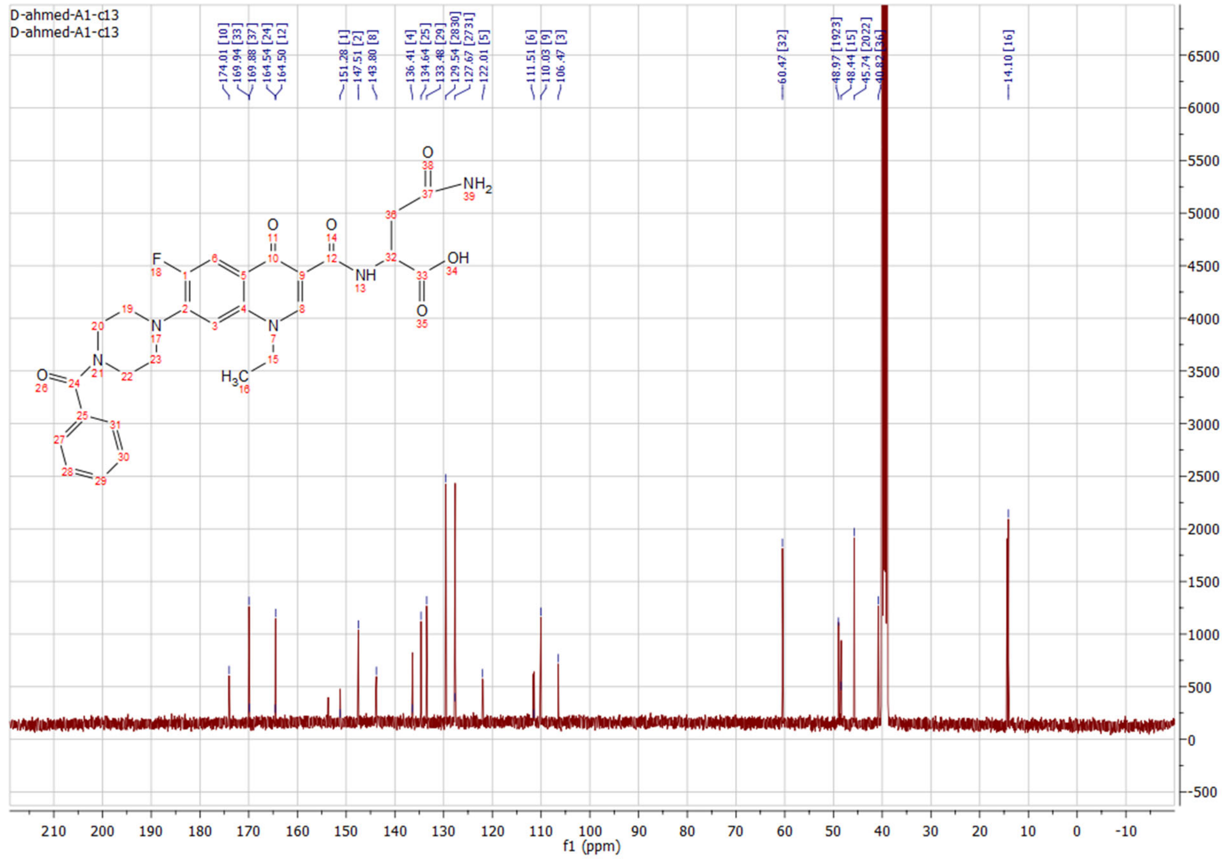

Figure S48: ^{13}C NMR of compound 7i

8a

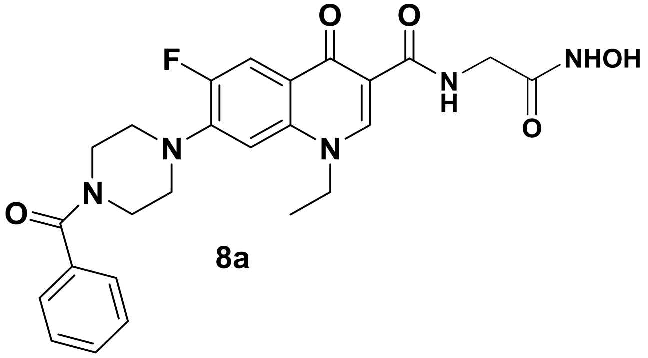

8a

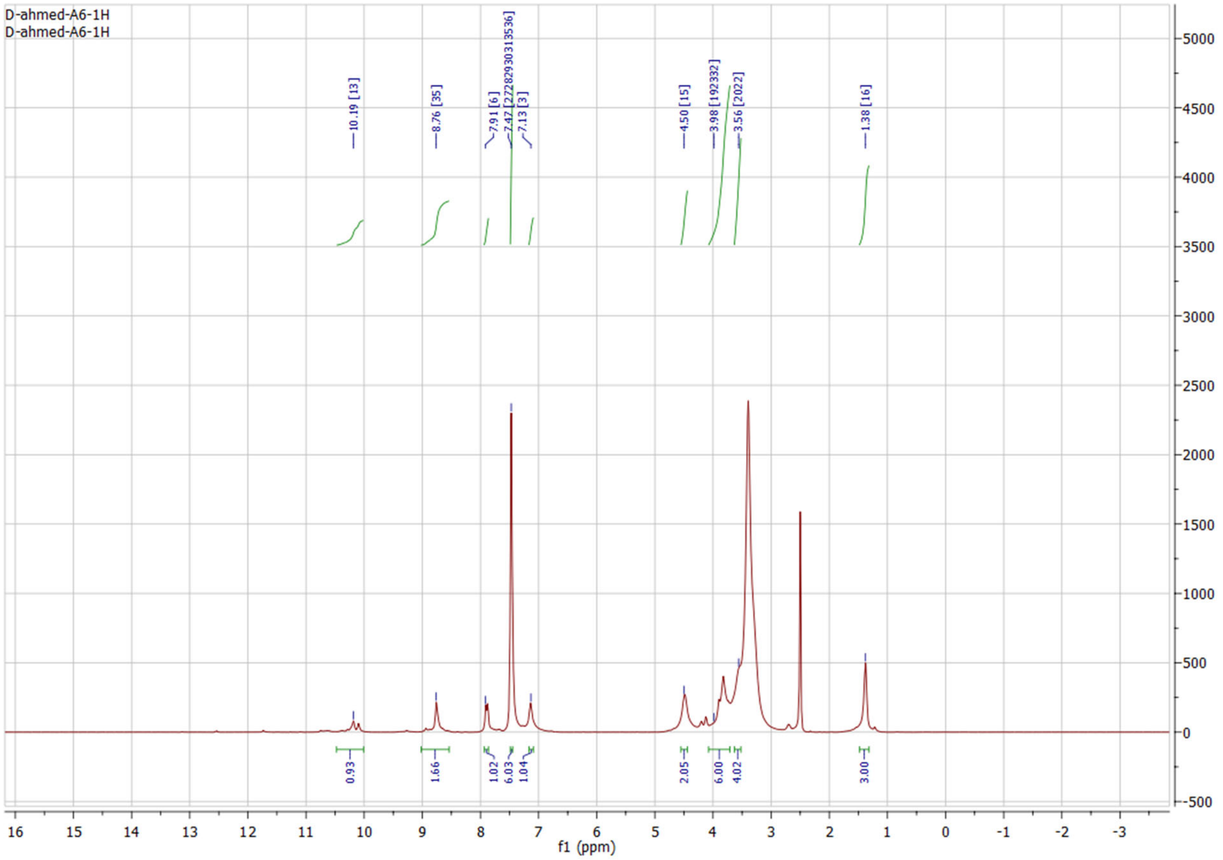

Figure S49: ^{1}H NMR of compound 8a

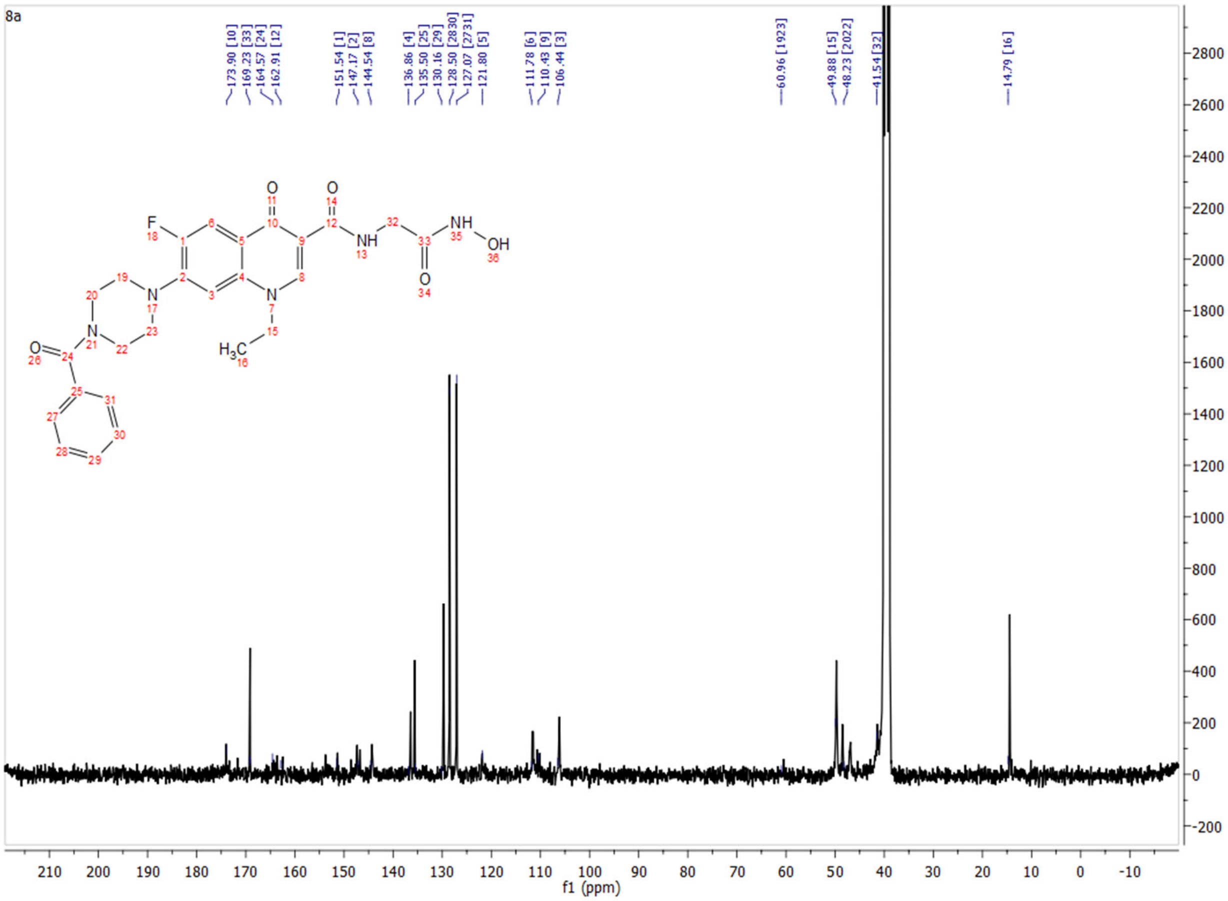

Figure S50: ^{13}C NMR of compound 8a

8b

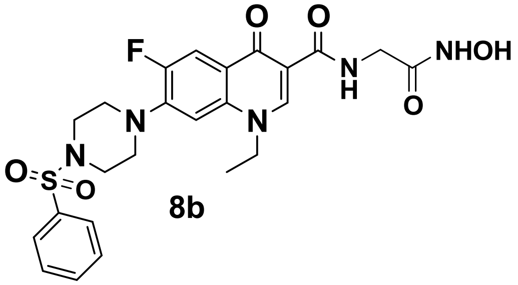

8b

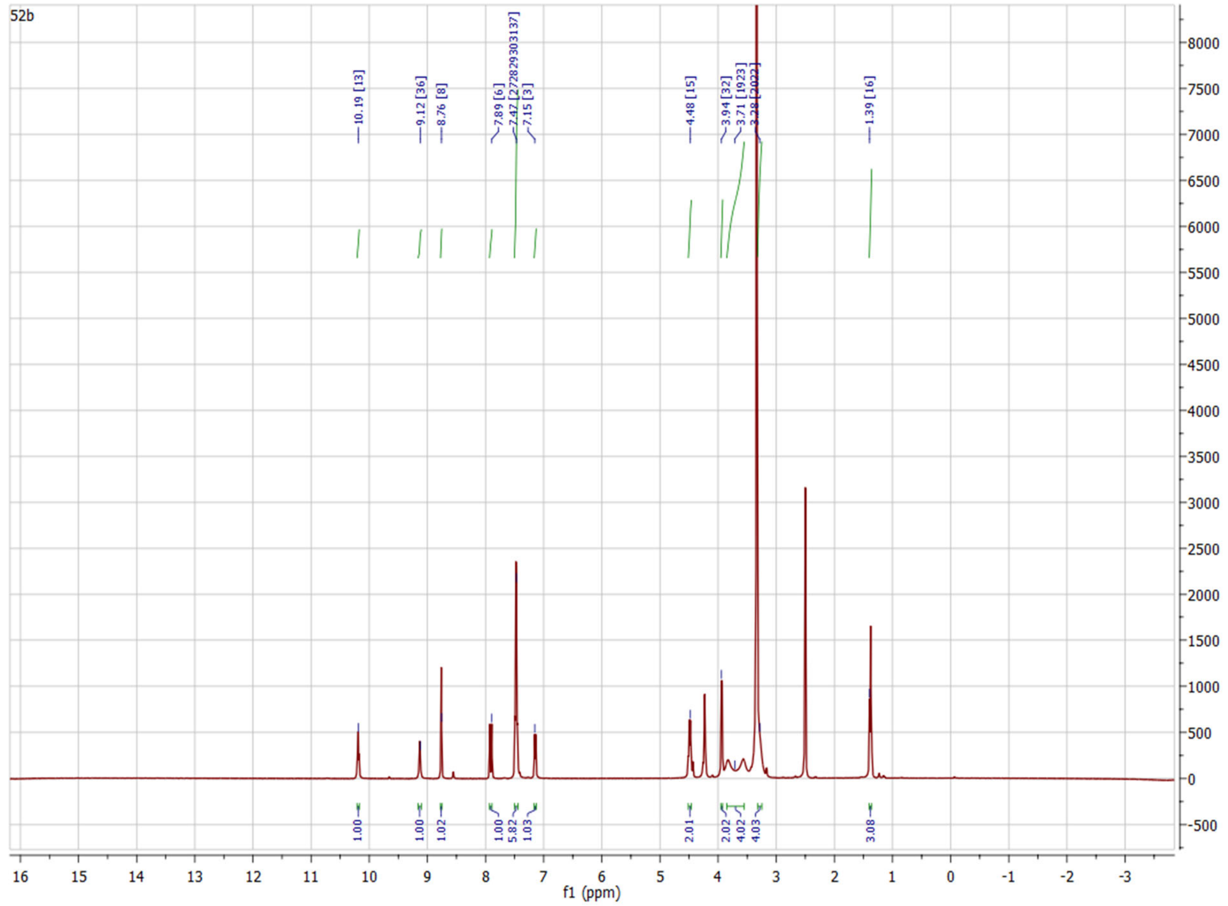

Figure S51: ^{1}H NMR of compound 8b

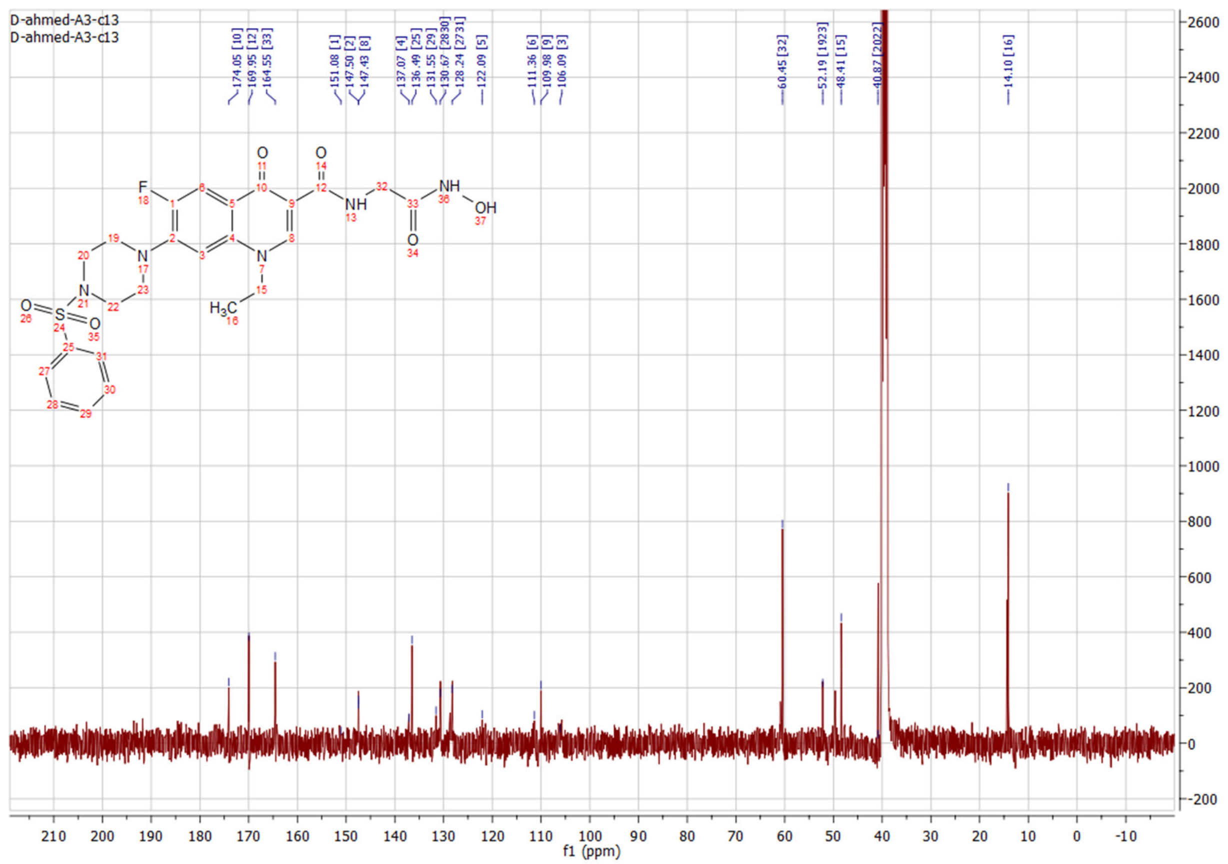

Figure S52: ^{13}C NMR of compound 8b

8c

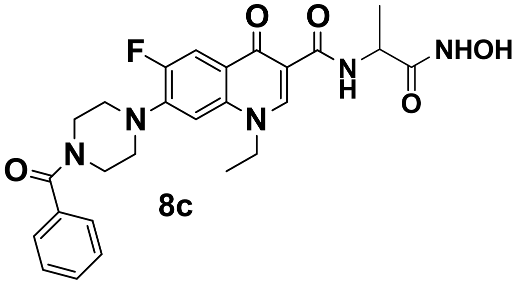

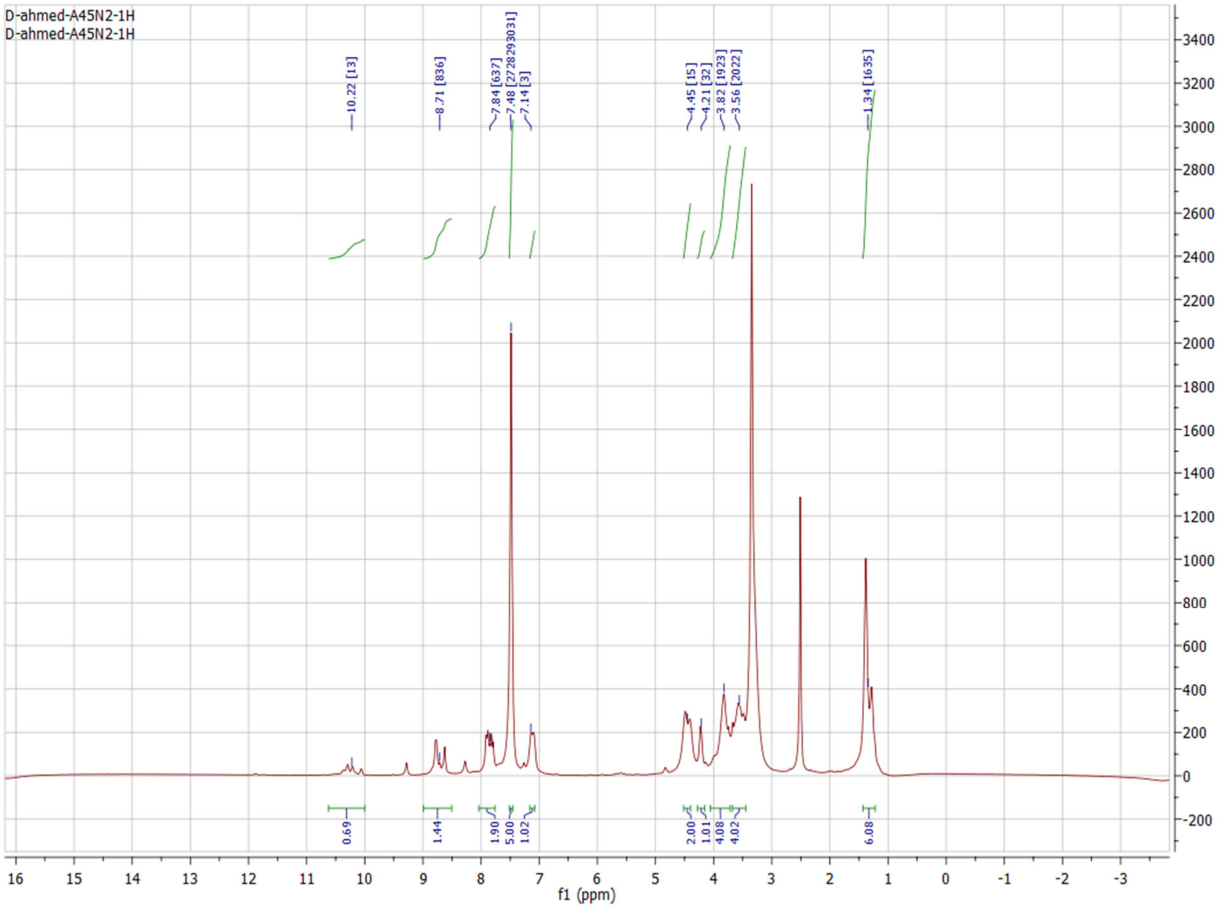

Figure S53: ^{1}H NMR of compound 8c

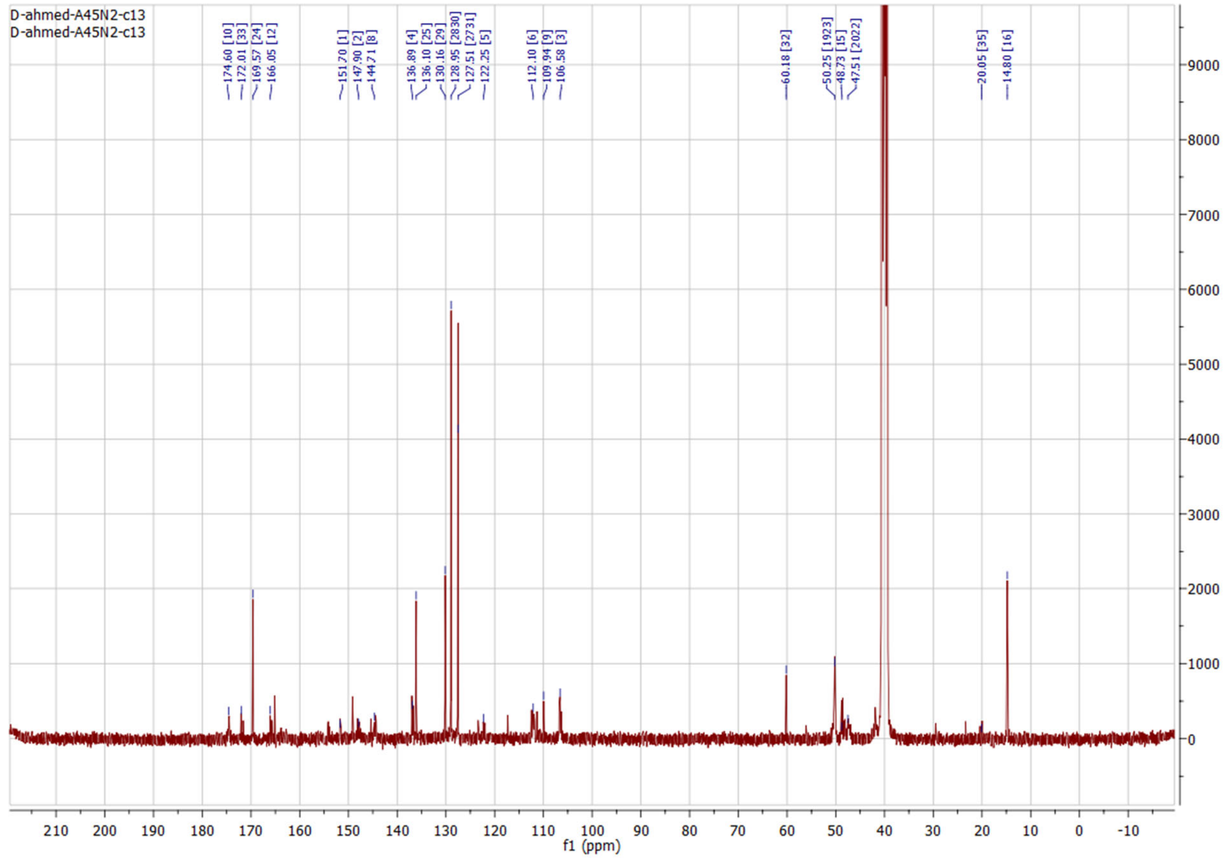

Figure S54: ^{13}C NMR of compound 8c

8d

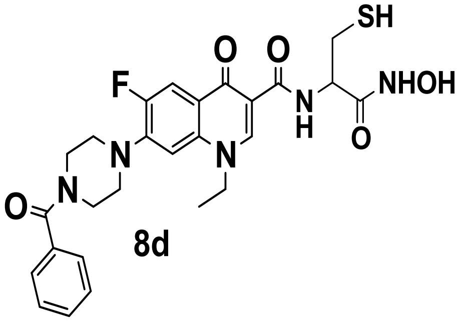

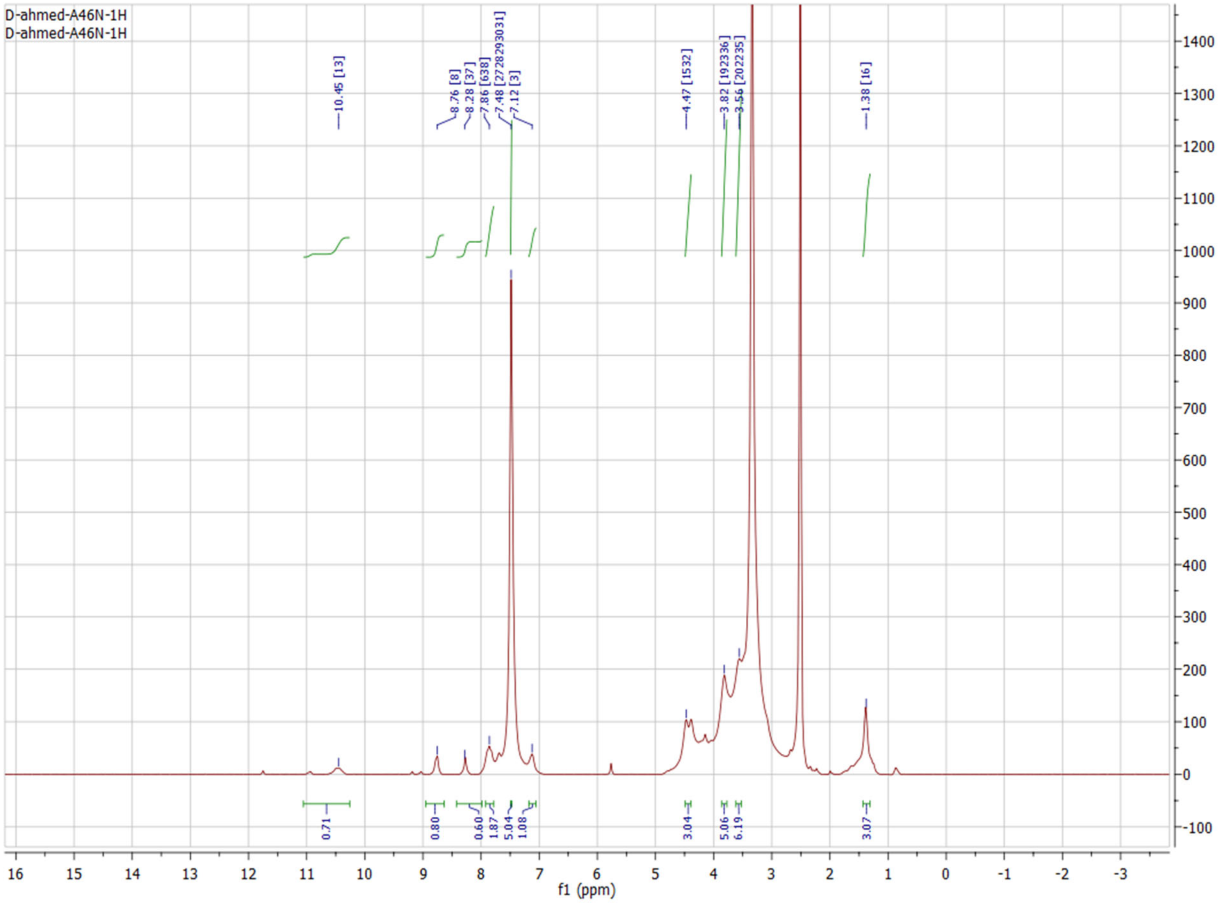

Figure S55: ^{1}H NMR of compound 8d

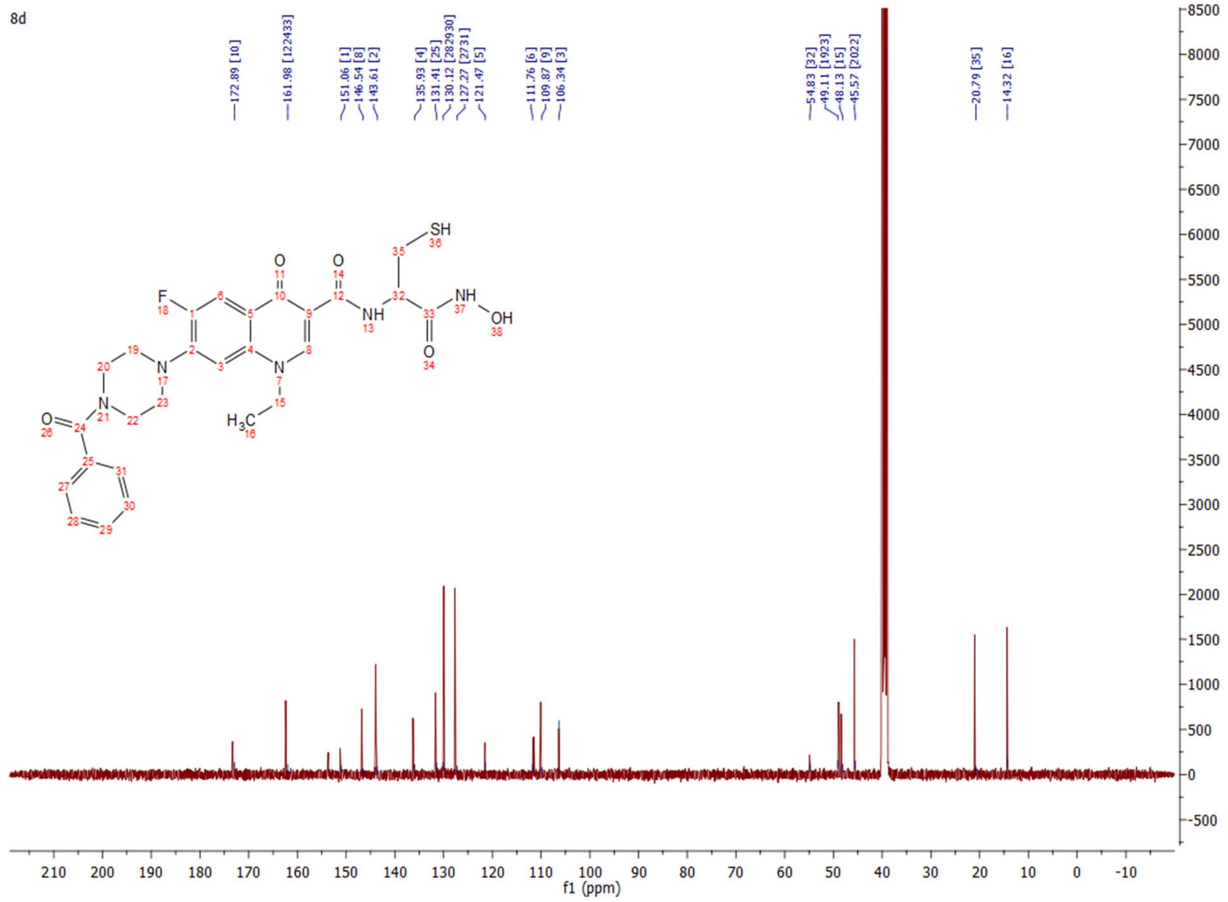

Figure S56: ^{13}C NMR of compound 8d

Table S1: Elemental analysis of newly synthesized compounds.

Sample code	C%	H%	N%	S%
**4a**	64.01	5.89	11.26	0
**4b**	60.85	5.79	10.37	0
**4c**	57.49	5.60	10.43	5.97
**4d**	58.23	5.71	10.27	5.83
**4e**	61.04	5.89	10.76	0
**4f**	55.48	5.09	10.13	5.57
**5a**	60.96	5.73	17.16	0
**5b**	58.71	5.80	15.39	0
**5c**	54.61	5.29	16.07	6.09
**5d**	55.39	5.44	15.61	5.97
**5e**	58.60	5.62	16.49	0
**7a**	62.31	5.48	11.90	0
**7b**	55.94	4.97	11.09	6.37
**7c**	56.76	5.40	10.85	6.11
**7d**	58.09	5.02	13.17	0
**7e**	63.41	5.64	11.57	0
**7f**	56.43	5.39	10.84	6.07
**7g**	59.47	5.38	10.89	6.21
**7h**	53.63	5.01	10.23	11.59
**7i**	60.47	5.43	13.29	0
**8a**	60.89	5.45	14.31	0
**8b**	54.51	5.12	13.40	6.11
**8c**	61.08	5.67	13.98	0
**8d**	57.89	5.42	13.21	5.86

2. Prediction of ADME/Tox

Text S1: Prediction of physicochemical properties

The physicochemical properties of a compound are determined by various structural features and can predict a compound's drug likeness. Key factors that influence the behavior of molecules in a living organism are hydrophobicity, molecular size, flexibility, and presence of various pharmacophoric features. Good bioavailability is achieved through balance between solubility and partitioning properties. Topological polar surface area (TPSA) and number of rotatable bonds (nrotb) are good descriptors of a drug's bioavailability. Two paradigms are commonly used to predict drug likeness, Lipinski's rule of five and Veber's rule. Lipinski's rule of five states that a compound with a molecular mass under 500 Dalton (MW), a coefficient of partition between octanol and water (LogP(o/w)) lower than 5, no more than five hydrogen bond donors (HBD), and no more than 10 hydrogen bond acceptors (HBA) could be a good drug candidate. Veber's rule says that a compound with 10 or fewer rotatable bonds (RTB) and a polar surface area (TPSA) no greater than 140 Å2 is likely to exhibit good oral bioavailability [1].

The physicochemical properties of the newly synthesized compounds were calculated using MOE 2020.1 (**Table S1**). TPSA was calculated as the surface areas that are occupied by oxygen and nitrogen atoms and by hydrogen atoms attached to them based on Ertl *et al*. [2]. It is considered a good descriptor for drug absorption, including intestinal absorption, bioavailability, and blood-brain barrier penetration. Molecules with TPSA values of 140 Å^2 or higher are expected to exhibit poor intestinal absorption [3]. Most of the synthesized compounds have TPSA values <140 Å^2 and are thus predicted to have good intestinal absorption. Molecules with more than 10 rotatable bonds may have problems with bioavailability [3]. Most of the compounds have 3-10 rotatable bonds predicting good bioavailability. LogP values are based on summation of fragment-based contributions and correction factors. It has been shown that for the compound to have a reasonable probability of being well-absorbed, LogP value must be in the range of −0.4-5 [3]. All the tested compounds had LogP values within the acceptable range for reasonable oral absorption. Except for compounds **4b**, **5b-d**, **7d**, **7i**, and **8b**, the analyzed compounds obeyed both Lipinski's and Veber's rules.

Table S2: Predicted physicochemical properties.

Code	Lip_acc	Lip_don	Lip_druglike	LogP(O/W)	LogS	TPSA	Weight	b_rotN
Nor	6	2	1	0.7250	-2.5094	72.879	319.33	3
4a	9	1	1	1.96	-5.48	99.26	508.549	10
4b	11	2	0	1.0019	-5.0096	128.72	554.575	11
4c	10	1	1	1.210	-5.3624	116.33	544.604	10
4d	10	1	1	1.5080	-5.8364	116.33	558.630	10
4e	8	1	1	3.1050	-6.0546	82.190	529.011	10
4f	11	2	0	0.2960	-4.8327	136.56	560.60	10
5a	10	4	1	0.2070	-5.0121	128.08	494.526	9
5b	12	5	0	-0.4099	-4.860	157.53	554.578	11
5c	11	4	0	-0.5429	-4.885	145.14	530.58	9
5d	11	4	0	-0.2450	-5.3595	145.14	544.607	9
5e	9	4	1	1.352	-5.577	111.01	514.989	9
7a	9	2	1	1.3550	-4.7495	110.26	480.496	8
7b	10	2	1	0.6050	-4.6229	127.33	516.549	8
7c	10	2	1	0.9029	-5.096	127.33	530.576	8
7d	12	2	0	1.398	-5.7024	156.08	539.519	10
7e	9	2	1	1.8170	-5.0767	110.26	494.522	8
7f	10	2	1	1.0670	-4.9501	127.33	530.576	8
7g	9	2	1	1.9270	-5.7379	149.05	526.588	9
7h	10	2	1	1.1770	-5.6114	166.13	562.643	9
7i	11	4	0	0.3350	-4.910	153.35	537.547	10
8a	10	3	1	0.67599	-4.8356	122.29	495.510	9
8b	11	3	0	-0.0740	-4.7090	139.36	531.565	9
8c	10	3	1	1.1380	-5.1628	122.29	509.537	9
8d	10	3	1	1.2480	-5.8241	161.08	541.604	10

Text S2: ADME/Tox prediction using pKCSM lab

in silico ADME/Tox profiles are helpful to predict the pharmacokinetic and toxicological properties of drug candidates. ADME/Tox predictions were first performed with the pKCSM web tool (http://structure.bioc.cam.ac.uk/ pkcsm) [4]. The following parameters were predicted:

Caco-2 permeability (Lag Papp in 10^{-6} cm/s)

Caco-2 (human colon carcinoma cell line) permeability measures the rate of flux of a compound across polarized Caco-2 monolayers, predicting intestinal drug absorption [5]. Log Papp values $\leq 10^{-6}$ cm/s indicate low intestinal absorption (0-20%), 10^{-6}-10^{-5} cm/s medium intestinal absorption (20-70%), and $>10^{-5}$ cm/s high intestinal absorption (70-100%) [6]. Most newly synthesized compounds are predicted to have medium intestinal absorption.

Steady-state volume of distribution (VDss)

Steady-state volume of distribution (Vss) reflects the blood and tissue volume, into which a drug is distributed and the relative binding of drug to protein in these spaces [7]. A drug with a high VDss has a propensity to leave the plasma and enter the extravascular compartments of the body, meaning that a higher dose of a drug is required to achieve a given plasma concentration. Conversely, a drug with a low Vd has a propensity to remain in the plasma meaning a lower dose of a drug is required to achieve a given plasma concentration [8]. Most compounds are predicted to have good volume of distribution and moderate plasma protein binding compared to norfloxacin.

Blood-brain barrier (BBB) permeability (Log BB)

The most common parameter used to quantify penetration of a compound across the BBB is the ratio of the concentration of compound measured in the brain to the concentration of compound measured in the blood at steadystate. This ratio is expressed as logBB (log[brain]/[blood]) and determines the total extent of brain exposure. Values of logBB can be used to determine if the compound is either BBB+ (crosses the BBB) or BBB− (does not cross the BBB)[9]. All newly synthesized compounds have lower logBB values than norfloxacin, predicting that all compounds have a low probability for crossing the BBB affecting the central nervous system, promising no or lower neuronal side effects than norfloxacin.

Metabolism

No metabolizing enzyme was predicted for norfloxacin, while all synthesized compounds are predicted to be metabolized by CYP3A4 and CYP2D6.

Total body clearance

Total body clearance describes the volume of plasma, from which a drug would be removed per unit time. It is a measure of the body's ability to remove a drug by either metabolism or excretion and determines the total systemic exposure to a drug (ratio of dose/clearance). Total body clearance is the sum of all processes, by which drugs are removed from the body or inactivated, primarily renal excretion and metabolism [10, 11]. All newly synthesized compounds are predicted to have total body clearance comparable to norfloxacin.

Toxicity

Toxicity of the newly synthesized compounds was evaluated by predicting oral rat acute toxicity (LD_{50}), oral rat chronic toxicity, hepatotoxicity, and AMES toxicity. All newly synthesized compounds showed higher IC_{50} values than norfloxacin (2.139 mol/kg) in oral rat acute toxicity predictions. The same trend was observed for oral rat chronic toxicity. Norfloxacin, like other fluoroquinolones, is associated with a low rate (1-3%) of serum enzyme elevations during therapy. While these abnormalities are generally mild, asymptomatic, and transient, it has also been linked to rare, but occasionally severe and even fatal cases, of acute liver injury [12, 13]. While the new compounds are predicted to be hepatotoxic as well, their LD_{50} values are higher than that of norfloxacin, suggesting less hepatotoxicity. The Ames test is used to assess carcinogenic effects of chemicals by determining the reversal rate of a histidine-auxotrophic *Salmonella typhimurium* strain [14]. Neither norfloxacin nor the new derivatives were predicted to be carcinogenic or mutagenic.

Table S3: ADME/Tox properties predicted by pKCSM.

Cmp	Caco2 permeability (log Papp in 10-6 cm/s)	Steady state volume of distribution VDss (log L/kg) (human)	Fraction unbound (human) (Fu)	BBB permeability (log BB)	Total Clearance (log ml/min/kg)	Oral Rat Acute Toxicity (LD50) (mol/kg)	Oral Rat Chronic Toxicity (LOAEL) (log mg/kg_bw/day)	Hepatotoxicity	AMES toxicity	Metabolizing enzyme
**Nor**	0.363	-0.201	0.478	-.0559	0.356	2.139	1.153	Yes	No	Not predicted
**4a**	0.631	-0.162	0.071	-0.711	0.662	2.594	0.495	Yes	No	CYP3A4
**4b**	-0.058	-0.19	0.129	-1.628	0.684	2.535	1.402	Yes	No	CYP3A4
**4c**	0.526	-0.444	0.087	-1.591	0.536	2.217	0.643	Yes	No	CYP3A4
**4d**	0.435	-0.4	0.109	-1.604	0.539	2.168	0.702	Yes	No	CYP3A4
**4e**	1.327	0.759	0.172	-0.758	0.768	2.681	0.408	Yes	No	CYP3A4
**5a**	-0.117	-0.59	0.141	-1.299	0.418	2.804	1.592	Yes	No	CYP3A4
**5b**	-0.205	-0.847	0.181	-1.956	0.692	3.015	1.227	Yes	No	CYP3A4
**5c**	0.027	-0.862	0.15	-1.547	0.589	2.924	1.469	Yes	No	CYP3A4
**5d**	0.102	-0.833	0.174	-1.578	0.592	2.918	1.351	Yes	No	CYP3A4
**5e**	0.386	0.285	0.237	-1.361	0.5	2.76	1.484	Yes	No	CYP3A4
**7a**	0.478	-1.242	0.063	-1.323	0.58	2.396	0.852	Yes	No	CYP2D6 CYP3A4
**7b**	-0.071	-1.507	0.067	-1.373	0.505	2.258	0.723	Yes	No	CYP3A4
**7c**	-0.099	-1.458	0.094	-1.368	0.508	2.263	0.62	Yes	No	CYP3A4
**7d**	-0.485	-0.711	0.14	-1.572	0.045	2.306	1.368	Yes	No	CYP3A4
**7e**	0.477	-1.174	0.058	-1.345	0.596	2.425	0.722	Yes	No	CYP2D6 CYP3A4
**7f**	0.373	-1.442	0.063	-1.351	0.52	2.262	0.592	Yes	No	CYP3A4
**7g**	0.448	-1.838	0.03	-1.707	0.131	2.435	1.055	Yes	No	CYP2D6 CYP3A4
**7h**	-0.096	-2.-89	0.029	-1.955	0.16	2.356	0.926	Yes	No	CYP2D6 CYP3A4
**7i**	-0.42	-1.659	0.061	-1.654	0.355	2.329	2.841	Yes	No	CYP2D6 CYP3A4
**8a**	-0.152	-0.577	0.133	-1.479	0.288	2.874	1.198	Yes	No	CYP3A4
**8b**	-0.078	-0.856	0.144	-1.727	0.458	2.977	1.069	Yes	No	CYP3A4
**8c**	-0.146	-0.54	0.119	-1.501	0.306	2.888	1.068	Yes	No	CYP3A4
**8d**	-0.181	-0.712	0.131	-2.009	0.038	2.915	1.466	Yes	No	CYP3A4

Text S3: ADME/Tox prediction using SwissADME

Further investigation of ADME properties was done for the most active compounds using the SwissADME web tool (http://www.swissadme.ch) [15]. The predicted values are shown in **Table S3**. Except for compounds **4f**, **5b**, **7g**, and **7i**, the studied compounds showed high gastrointestinal (GI) absorption predictions comparable to norfloxacin. TPSA values were similar to those predicted with MOE 2020.1. Most of the compounds showed aqueous solubility between -3.17 and -4.79, indicating good to moderate aqueous solubility. Lipophilicity was assessed using the logarithm of the n-octanol/water partition coefficient, which was predicted using the consensus LogPo/w, which is closely related to transport processes, including membrane permeability and penetration, which directly affect the ability of a drug to reach its target site[16]. Most of tested compounds had LogPo/w values between 0.91 and 2.59, indicating moderate permeability and tissue penetration (logP ($0 < logP < 3$) [17]. Except for **4f**, **5b**, **7g**, and **7i**, the investigated compounds showed good bioavailability scores ranging from 0.55 to 0.56.

Table S4: Physicochemical and pharmacokinetic properties predicted by SwissADME.

Cmpd	Consensus Log $P_{o/w}$	TPSA (Å²)	Log S (ESOL)	Water solubility class	GI absorption	BBB permeant	Bioavailability Score
**Nor**	0.98	74.57	-1.29	Very soluble	High	No	0.55
**4d**	2.59	126.40	-4.79	Moderately soluble	High	No	0.55
**4f**	1.61	146.63	-3.94	Soluble	Low	No	0.17
**5b**	0.91	159.23	-3.17	Soluble	Low	No	0.17
**7b**	1.63	137.40	-4.02	Moderately soluble	High	No	0.56
**7c**	2.01	137.40	-4.33	Moderately soluble	High	No	0.56
**7g**	2.20	150.75	-4.41	Moderately soluble	Low	No	0.11
**7i**	1.14	155.04	-3.48	Soluble	Low	No	0.11
**8a**	1.30	123.98	-3.55	Soluble	High	No	0.55
**8c**	1.78	123.98	-3.88	Soluble	High	No	0.55

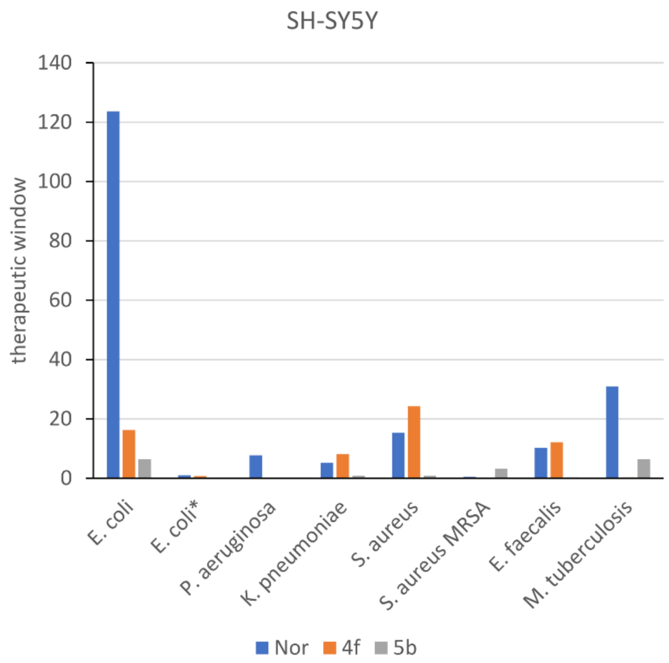

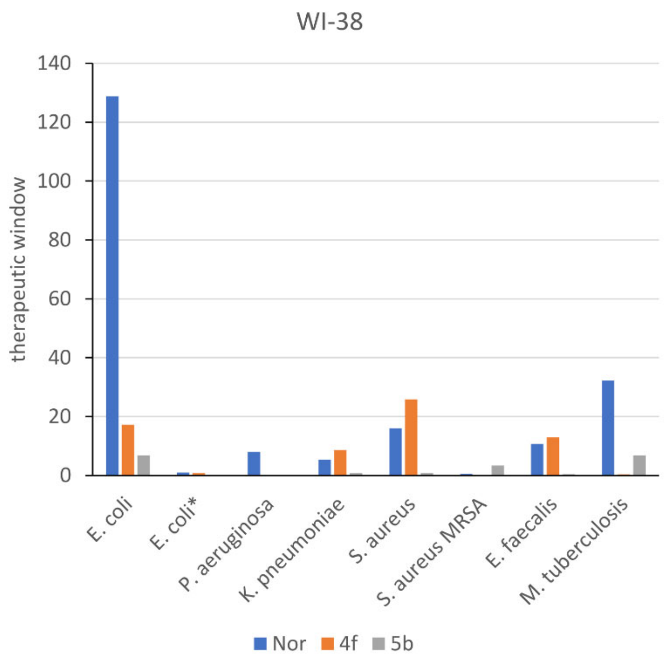

Figure S57: Therapeutic windows against different bacterial species based on toxicity against SH-SY5Y (top) and WI-38 (bottom) cell lines.

3. Molecular modeling

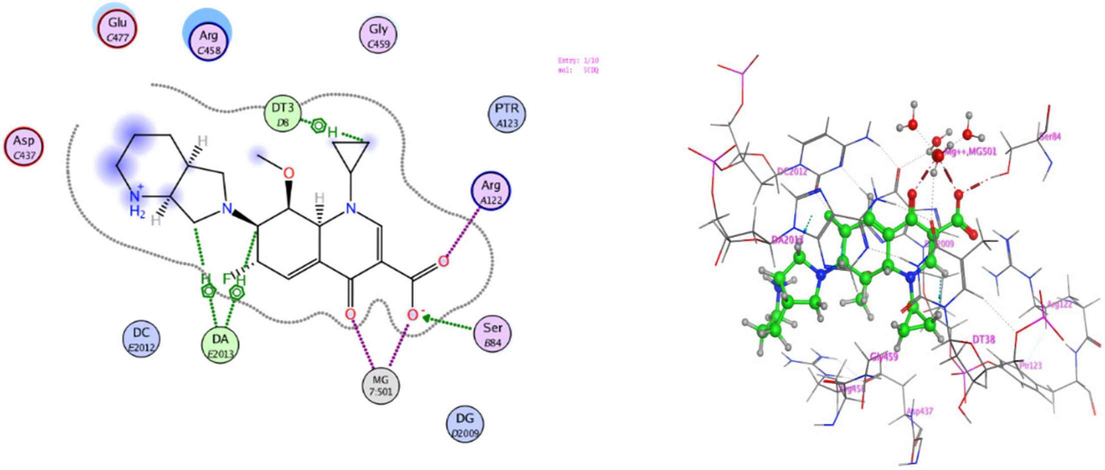

Figure S58: 2D and 3D interactions of moxifloxacin with *S. aureus* DNA gyrase.

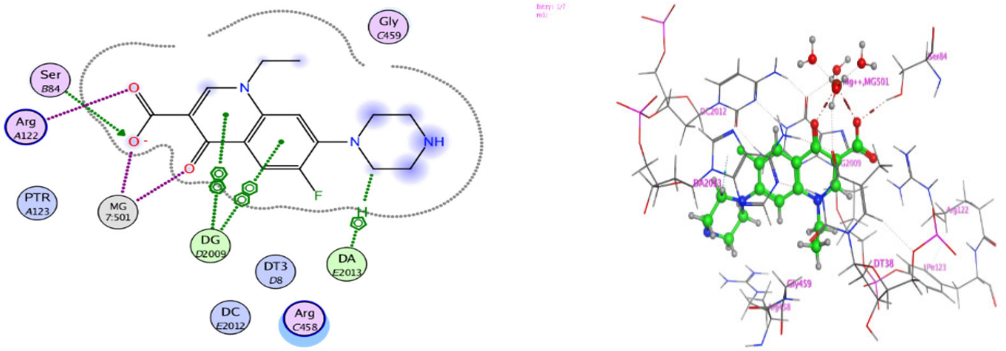

Figure S59: 2D and 3D interactions of norfloxacin with *S. aureus* DNA gyrase.

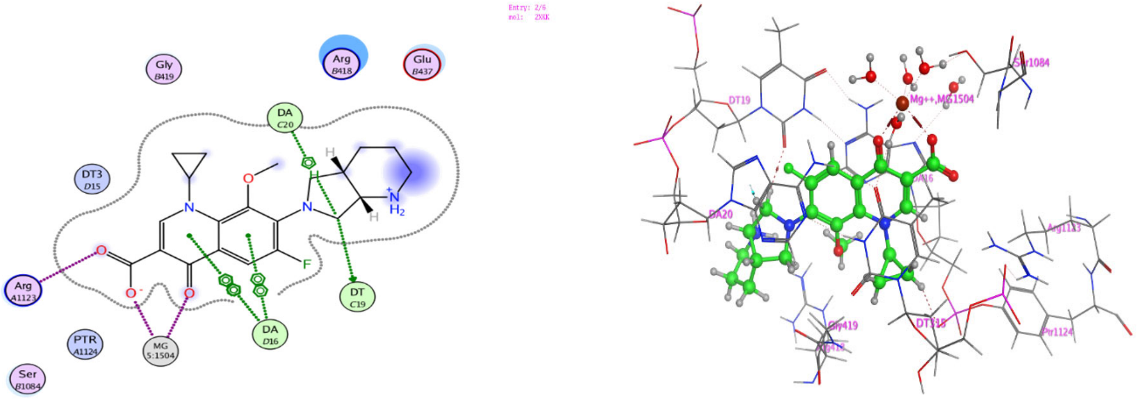

Figure S60: 2D and 3D interactions of moxifloxacin with *A. baumannii* topoisomerase IV.

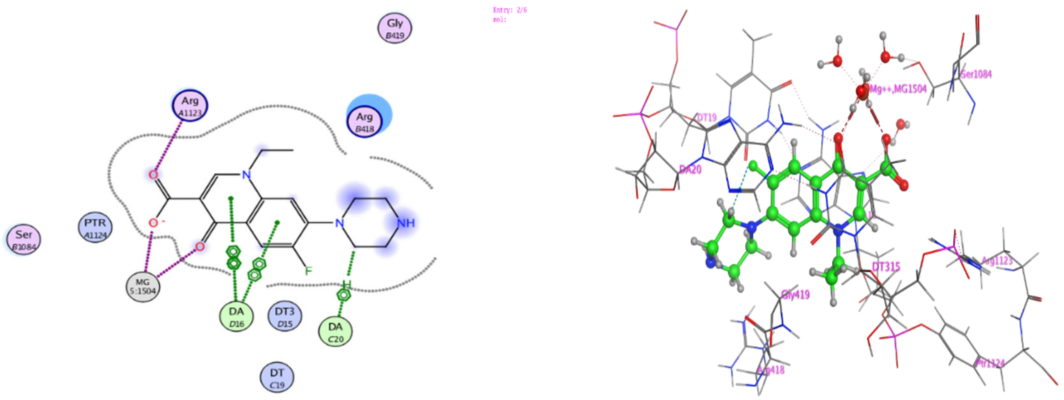

Figure S61: 2D and 3D interactions of norfloxacin with *A. baumannii* topoisomerase IV.

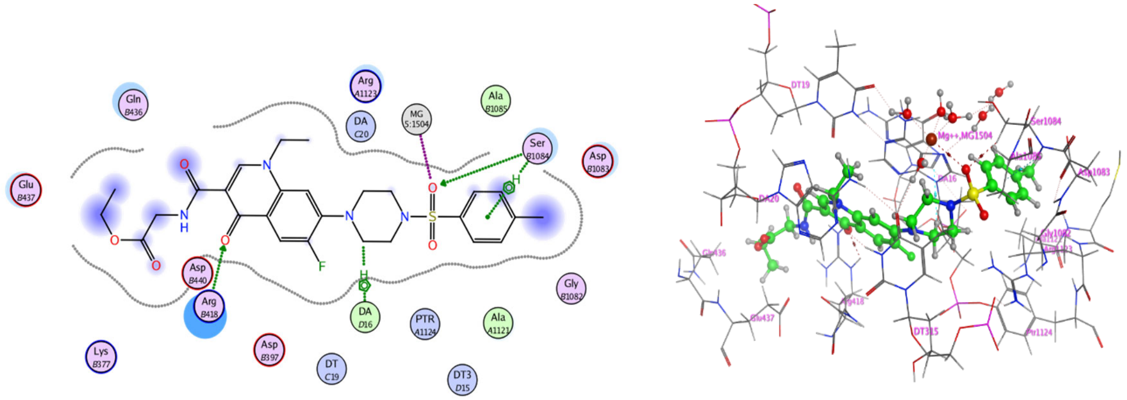

Figure S62: 2D and 3D interactions of compound **4d** with *A. baumannii* topoisomerase IV.

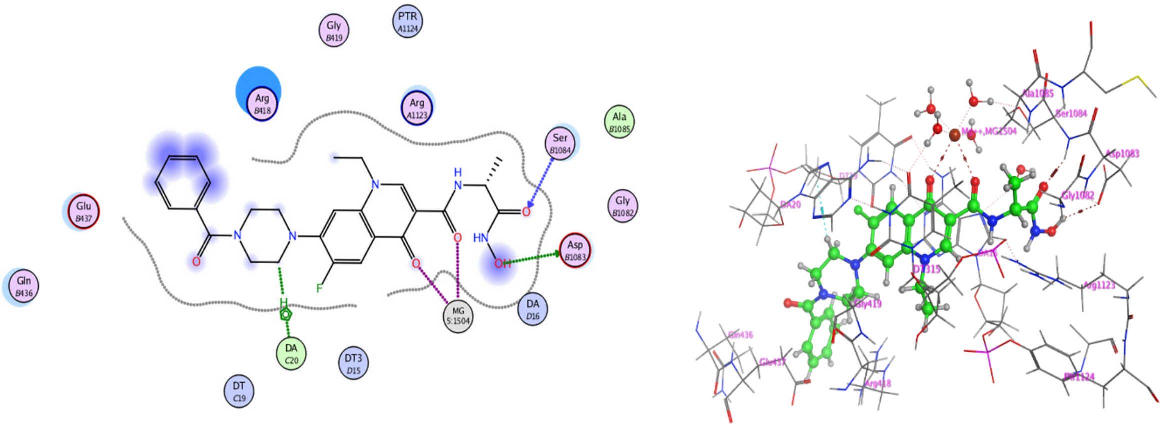

Figure S63: 2D and 3D interactions of compound **8c** with *A. baumannii* topoisomerase IV.

Text S4: Docking on *P. aeruginosa* LpxC

Docking studies of the designed compounds were performed based on the three-dimensional structure and conformations of the co-crystal structure of *P. aeruginosa* LpxC-50432 complex (PDB code: 6mod) [18]. The binding patterns and interactions of the designed molecules was then compared to that of co-crystallized ligand, N-[(1S)-2-(hydroxyamino)-1-(3-methoxy-1,1-dioxo-1lambda~6~-thietan-3-yl)-2-oxoethyl]-4-(6-hydroxyhexa-1,3-diyn-1-yl) benzamide (JWV) and norfloxacin at the active site. The docking protocol was validated by re-docking of the co-crystalized JWV ligand at the active site of LpxC (**Figure S65**, re-docking rmsd = 0.4827 Å, binding score = -10.74 kcal mol^{-1}). All essential interactions accomplished by the co-crystalized ligand with the key amino acids in the active site were reproducible in our docking setup. The validated docking setup was then used to investigate the ligand-receptor interactions for norfloxacin (**Figure S66**, score = -6.21 kcal mol^{-1}).

The residues involved in the binding of the co-crystallized ligand to LpxC (**Figure S65**) were Thr190, Phe191, Lys238, Leu18, His78, Met62, Asp241, and Mg^{2+} [19]. The main interactions were H-bonding between the hydroxyl of the hydroxamic group with His78 (2.59 Å), H-bond of the amino group of hydroxamic group with Met62 (2.27 Å), H-bonding between the carbonyl group and Thr190 (1.73 Å), a π-H bond between the phenyl ring with Leu18, H-bonding of the oxygen of the sulphonyl group with Lys238 (1.92 Å), and a coordination bond between the carbonyl group and Mg^{2+} (2.16 Å). In case of norfloxacin (**Figure S66**), the carbonyl of the carboxylic acid group formed a coordination bond with Mg^{2+} with length of 2.73 Å, a H-bond between the oxygen of carboxylic acid and Phe191 (2.51 Å), and a π-cation interaction between the phenyl ring of quinolone and Lys238.

Compounds **4d**, **4f**, **5b**, **7b**, **7d**, **7g**, **7i**, **8a**, and **8c** were selected for docking on LpxC as they were most active against *E. coli* (**Figure S67-77**). Compounds **4f**, **5b**, **7i**, and **8c** showed the lowest binding scores (-9.63, -9.42, -10.74, and -9.64 kcal mol^{-1}, respectively). A coordination bond with Mg^{2+} was formed by the hydroxyl group of the serine amino acid of compound **4f** (2.29 Å), with the NH_2 group of the hydrazide of compound **5b** (2.06 Å), the carboxylate carbonyl group of compound **7b** (1.91 Å), and the hydroxamic carbonyl group of compound **8a** (2.51 Å). A H-bond between Thr190 and compounds **4f**, **5b**, **7b**, **7g**, **8a**, and **8c** with an average length of 2.19 Å was formed with the ester carbonyl group, NH of hydrazide, carboxylate carbonyl group, or hydroxamic NH group, respectively. A H-bond with Lys238 with an average length of 2.19 Å was

formed with the amidic carbonyl groups of compounds **4f** and **7b** and with the quinolone carbonyl group and hydroxamic carbonyl group of compounds **5b** and **8c**, respectively. The SH group of compound **7g** formed a H-bond with Lys238 as well, but also with Asp241. Compounds **4f**, **5b**, **7i**, **8a**, and **8c** formed a H-bond with Met62 with an average length of 2.3 Å through the NH group (amidic NH of compound **4f** and **7i**, hydrazide NH of compound **5b**, and hydroxamic NH group of compounds **8a** and **8c**), while an interaction with Phe191 was mediated by water bridges in all investigated compounds.

In addition to these interactions, the newly designed compounds interacted with other amino acids mediated by hydrazide amino acid and hydroxamic amino acid residues and the added moieties at the *N*-4 of piperazine ring through H-bonds and π-cation bonds, such as a H-bond with His264 with the quinolone carbonyl group (compounds **4f** and **7b**), the hydrazide NH group (compound **5b**), and hydroxamic OH group (compound **8c**). Compounds **4f**, **5b**, and **7i** formed H-bonds with Lys142 mediated by the ester carbonyl (**4f**) and benzoyl carbonyl groups (**5b** and **7i**). Compound **5b** and **8a** formed H-bonds with Glu77 through their hydrazide NH_2 and hydroxamic OH group, respectively. The carbonyl group of the benzoyl moieties of compounds **5b** and **8a** formed H-bonds with Lys261. Compound **7b** formed a H-bond with this residue through its sulphonyl group. All these non-covalent interactions contribute to the low binding scores of the compounds.

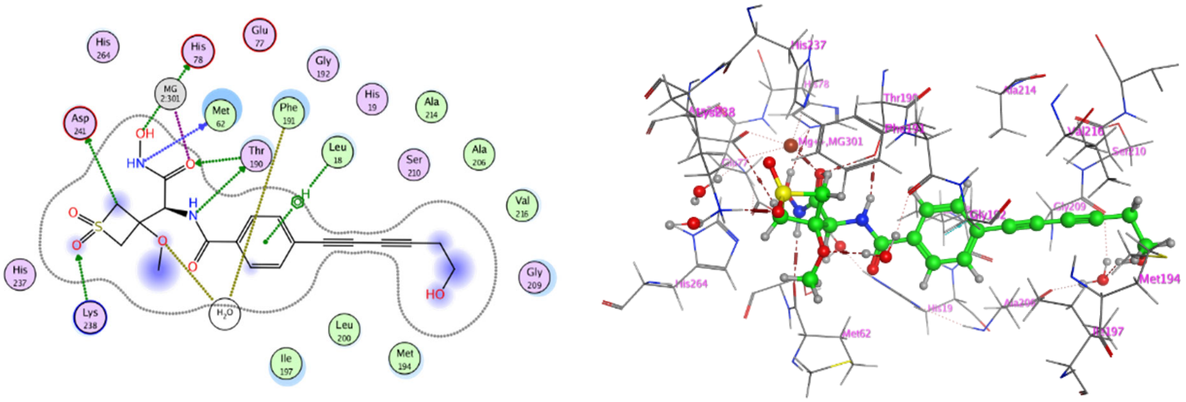

Figure S64: 2D and 3D interactions of JWV with *P. aeruginosa* LpxC.

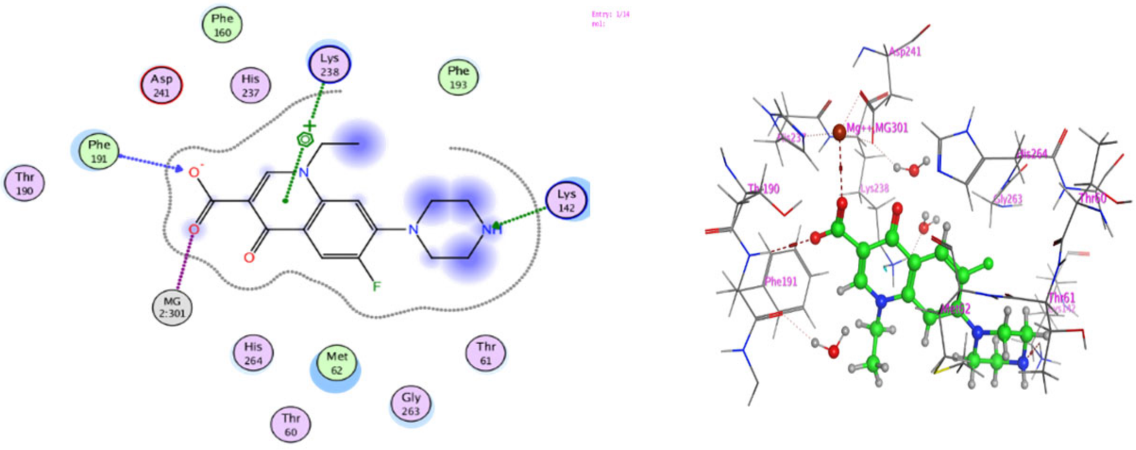

Figure S65: 2D and 3D interactions of norfloxacin with *P. aeruginosa* LpxC.

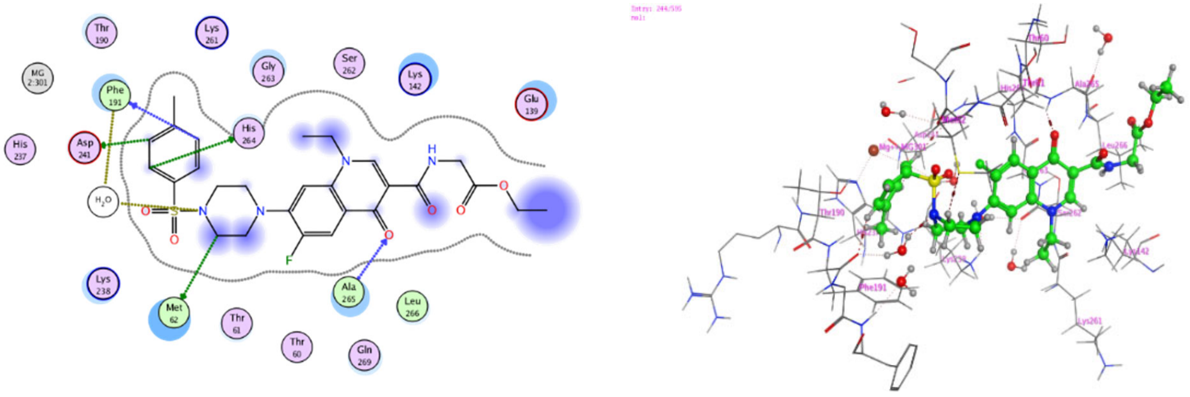

Figure S66: 2D and 3D interactions of compound **4d** with *P. aeruginosa* LpxC.

Compound 4f

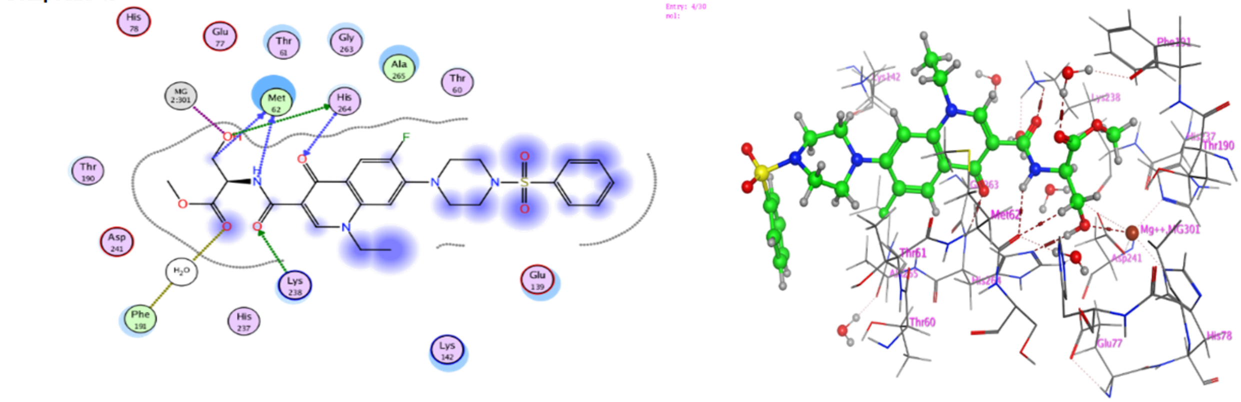

Figure S67: 2D and 3D interactions of compound **4f** with *P. aeruginosa* LpxC.

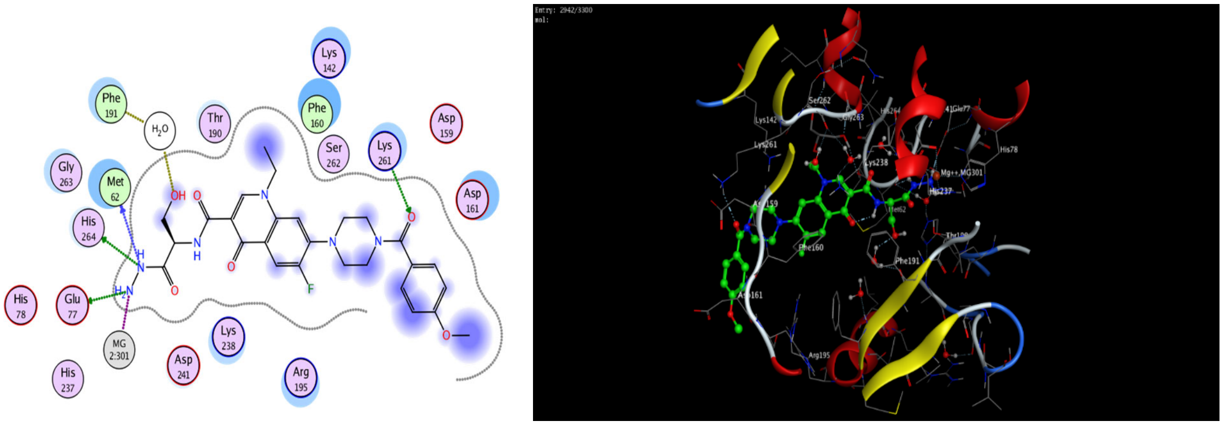

Figure S68: 2D and 3D interactions of compound **5b** with *P. aeruginosa* LpxC.

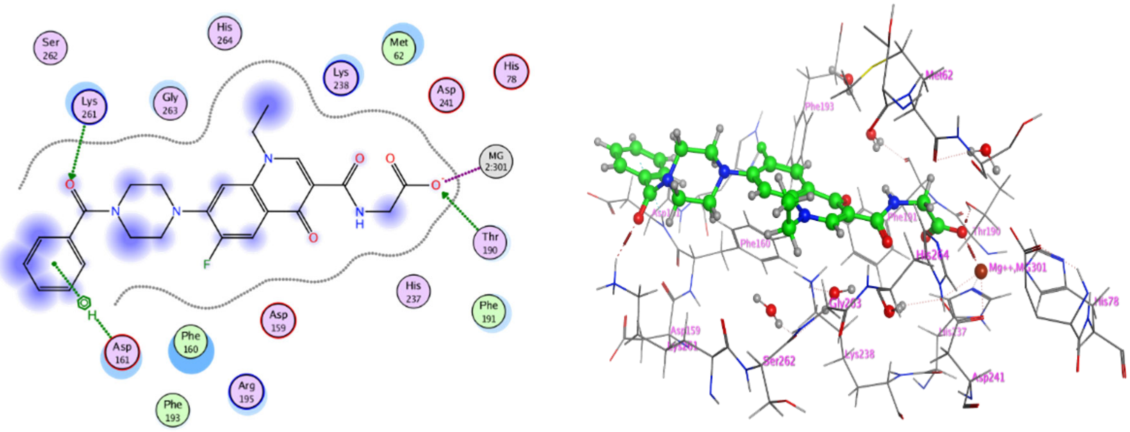

Figure S69: 2D and 3D interactions of compound **7a** with *P. aeruginosa* LpxC.

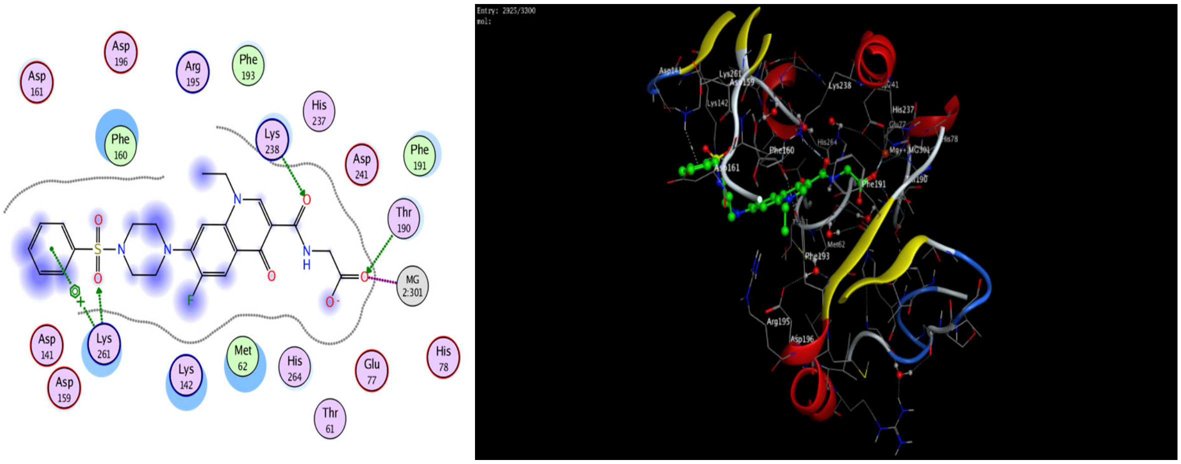

Figure S70: 2D and 3D interactions of compound **7b** with *P. aeruginosa* LpxC.

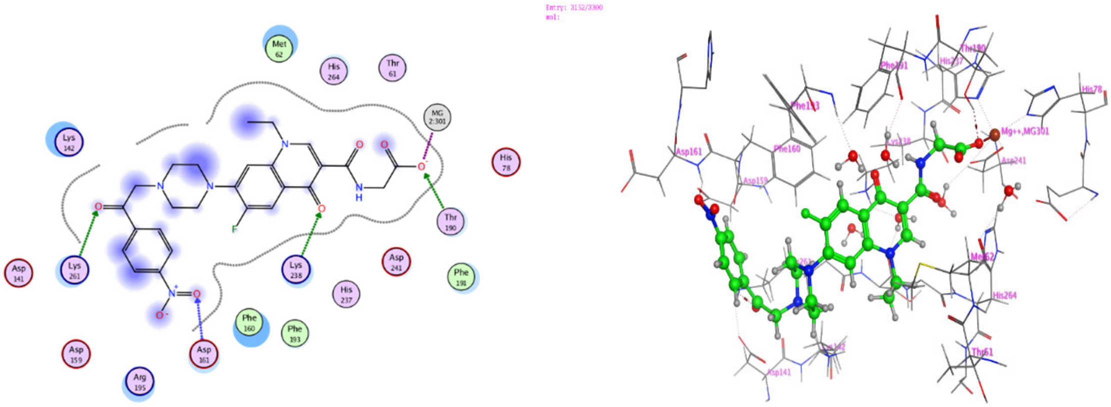

Figure S71: 2D and 3D interactions of compound **7d** with *P. aeruginosa* LpxC.

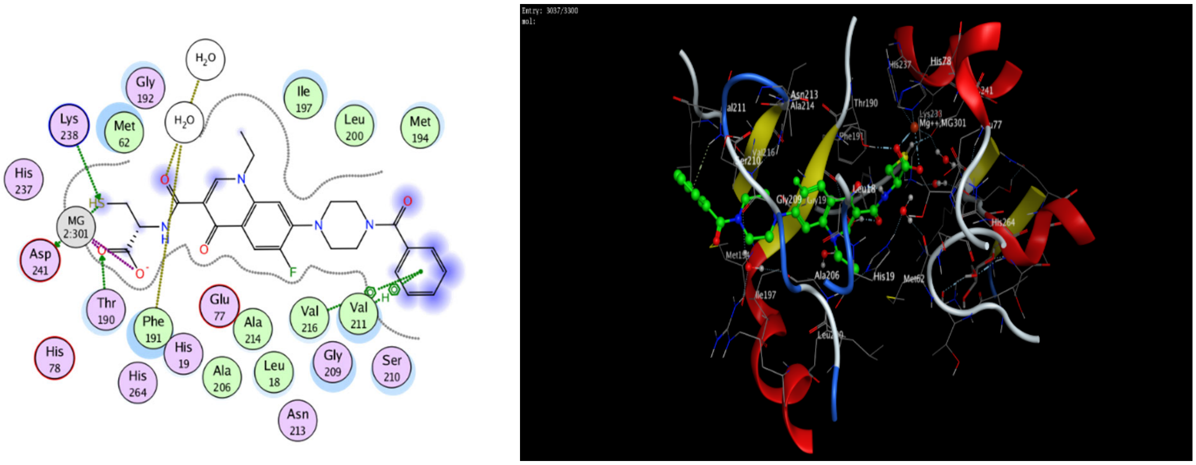

Figure S72: 2D and 3D interactions of compound **7g** with *P. aeruginosa* LpxC.

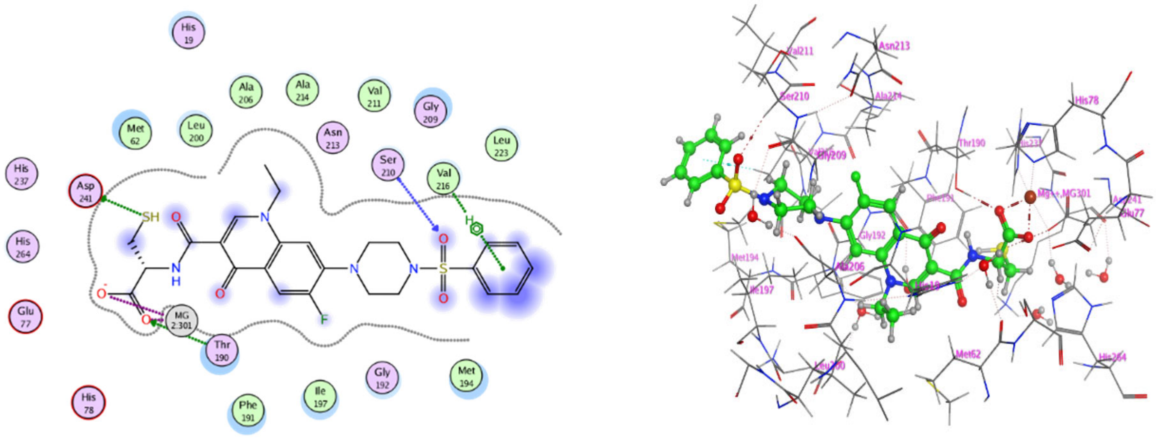

Figure S73: 2D and 3D interactions of compound **7h** with *P. aeruginosa* LpxC.

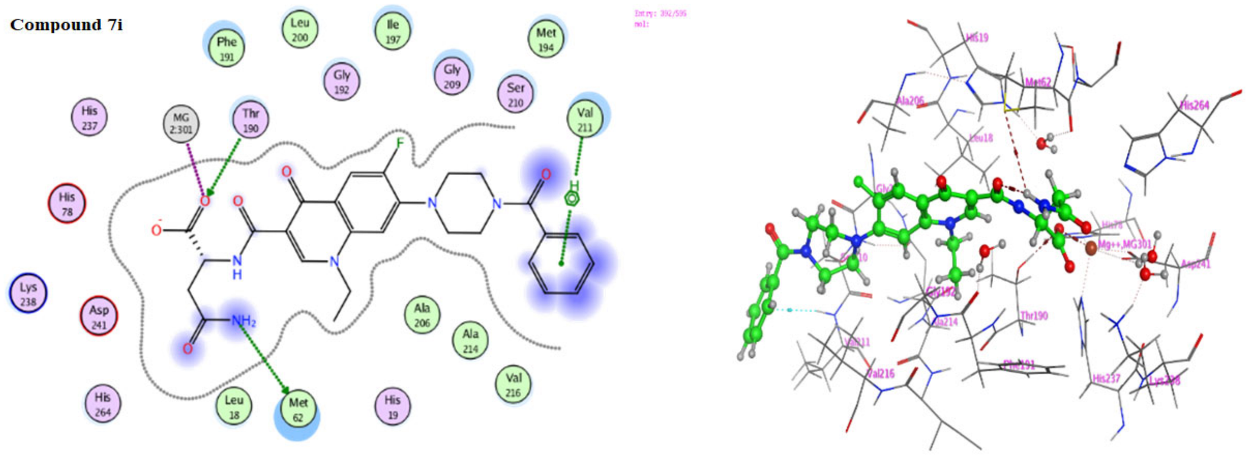

Figure S74: 2D and 3D interactions of compound **7i** with *P. aeruginosa* LpxC.

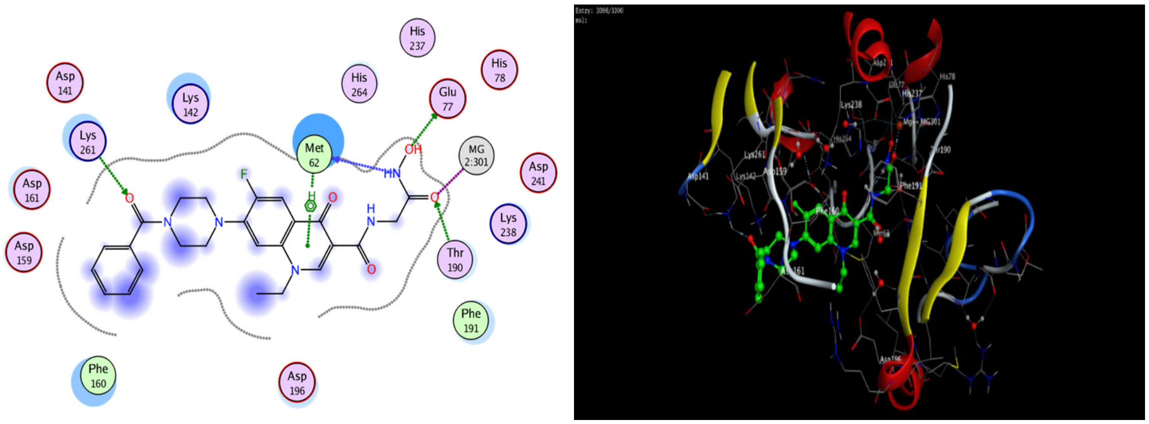

Figure S75: 2D and 3D interactions of compound **8a** with *P. aeruginosa* LpxC.

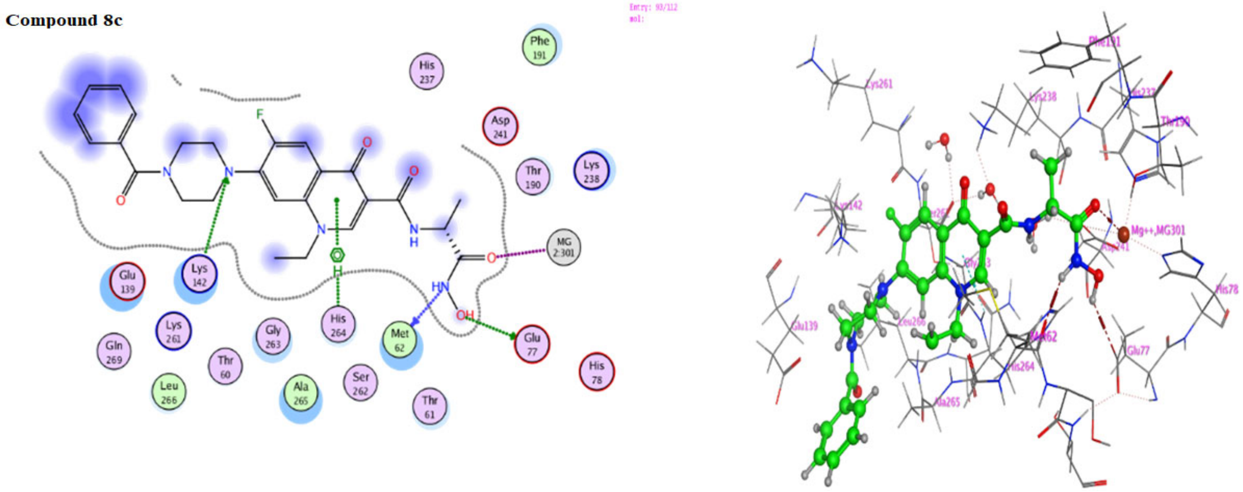

Figure S76: 2D and 3D interactions of compound **8c** with *P. aeruginosa* LpxC.

Text S5: Docking on *M. smegmatis* NagA

Docking studies were performed on the three-dimensional structure and conformations of the crystal structure of *M. smegmatis* *N*-acetyl-D-glucosamine-6-phosphate deacetylase (D267A mutant) in complex with *N*-acetyl-D-glucosamine-6-phosphate (PDB code 6fv4) [20]. The binding patterns and interactions of the designed molecules were then compared to that of *N*-acetyl-D-glucosamine-6-phosphate and norfloxacin at the active site. The docking protocol was validated by re-docking of the co-crystalized ligand (**Figure S78**, re-docking rmsd = 1.5535 Å, binding score = -12.63 kcal mol^{-1}). All key interactions of the co-crystalized ligand with amino acids at the active site were reproducible. The validated docking setup was then used to investigate the ligand-receptor interactions of norfloxacin (**Figure S79**, score = -8.40 kcal mol^{-1}). Docking of norfloxacin showed that the oxygen of the carboxylic acid group formed a coordination bond with Cd^{2+} (1.75 Å) and two H-bonds with Gly132 and Ala133 mediated by the ketonic carbonyl group (2.29 Å).

Compounds **5b**, **7b**, **7c**, **7d**, **7f**, **7h**, **7i**, and **8a** were selected for docking on NagA based on their high activity against *B. subtilis* (**Figure S80-87**). Compounds **7b**, **7c**, **7f**, and **8a** showed the lowest binding scores (-22.79, -22.58, -20.44, and -19.69 kcal mol^{-1}, respectively). The tested compounds interacted with the same amino acids as the co-crystallized ligand and norfloxacin, where common interactions in all docked compounds include coordination bonds with both Cd^{2+} and Zn^{2+} with an average length of 1.86 and 2.52 Å, respectively, which was mediated by carboxylate, hydroxamic acid, and hydrazide groups, and H-bonds with Ala133 and Gly132 formed with the amidic carbonyl groups (average length 2.03 and 2.21 Å, respectively). The quinolone rings of compounds **7c** and **7f** formed π-H bonds with Ala213 and Ile301, respectively. Compound **8a** formed a H-bond with His244 through its amidic NH group, while compound **7f** formed a H-bond with the same residue through its quinolone carbonyl group.

The newly designed compounds also formed additional interactions mediated by the added amino acid residues and moieties at the *N*-4 of piperazine ring through H-bonds and π-cation bonds, such as H-bonds between His134 and the carboxylate oxygen of compound **7i** and the carboxylic carbonyl groups of compounds **7b** and **7c,** respectively. The sulphonyl group of the phenylsulphonyl moiety of compound **7f** formed a H-bond with Arg294, and its quinolone ring formed a π-H bond with Thr300. The hydroxamic NH group of compound **8a** formed a H-bond with Ala302. The side chain amidic NH_2 group of compound **7i** formed a H-bond with the same residue. All these non-covalent interactions contribute to the low binding scores of the compounds.

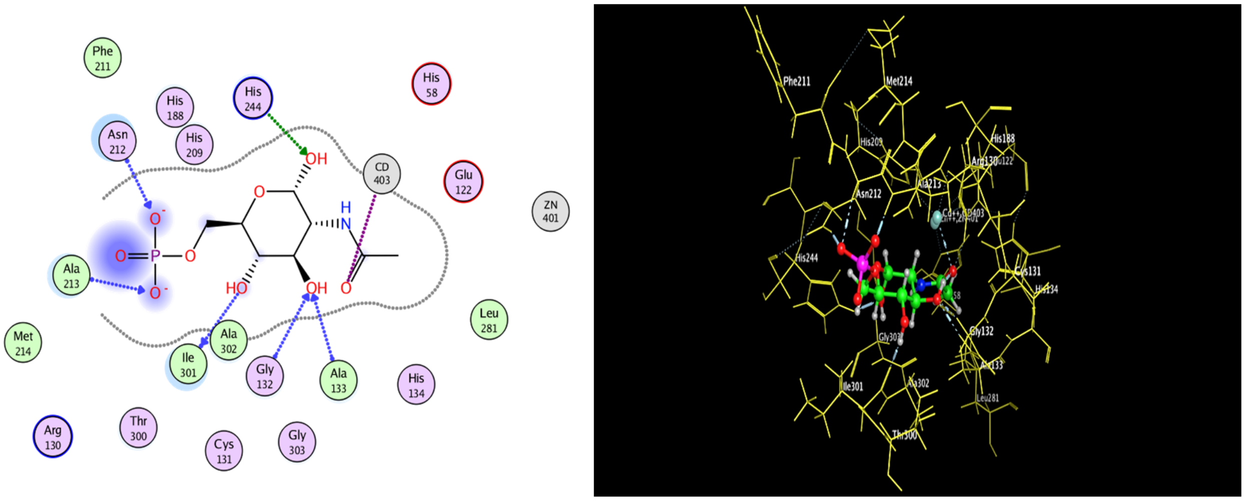

Figure S77: 2D and 3D interactions of *N*-acetyl-D-glucosamine-6-phosphate with *M. smegmatis* NagA.

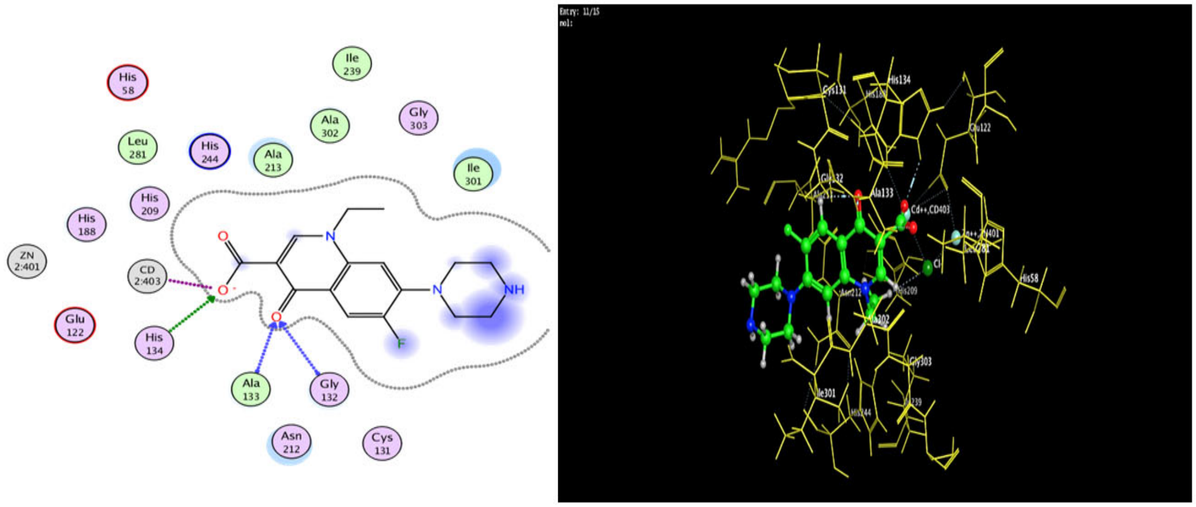

Figure S78: 2D and 3D interactions of norfloxacin with *M. smegmatis* NagA.

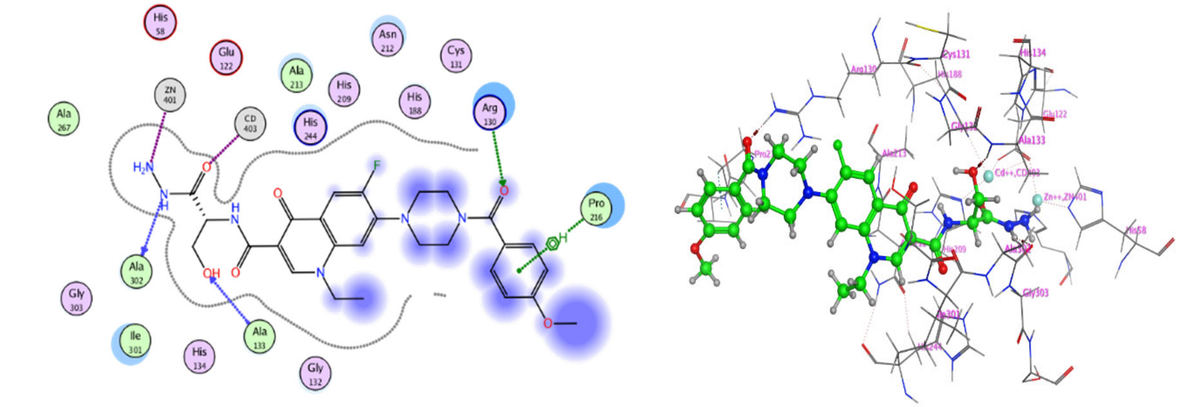

Figure S79: 2D and 3D interactions of compound **5b** with *M. smegmatis* NagA.

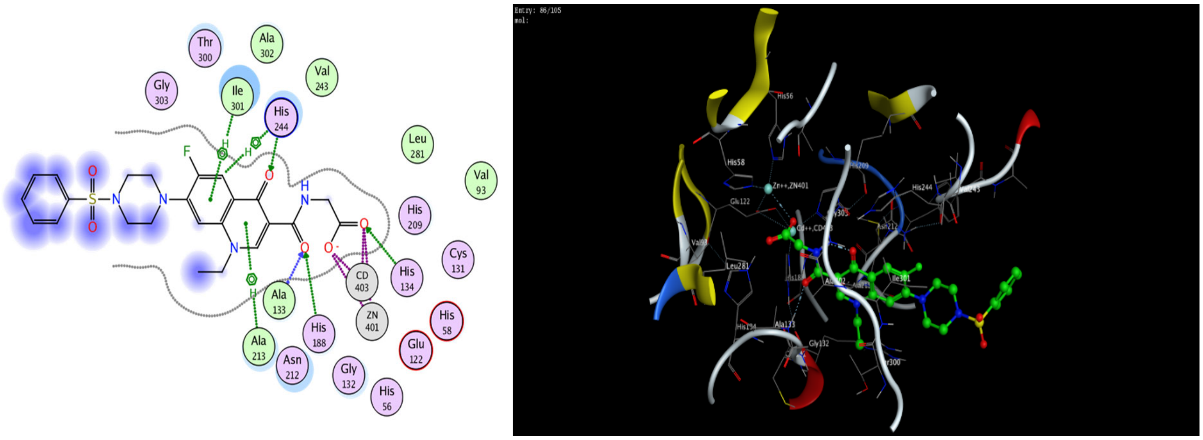

Figure S80: 2D and 3D interactions of compound **7b** with *M. smegmatis* NagA.

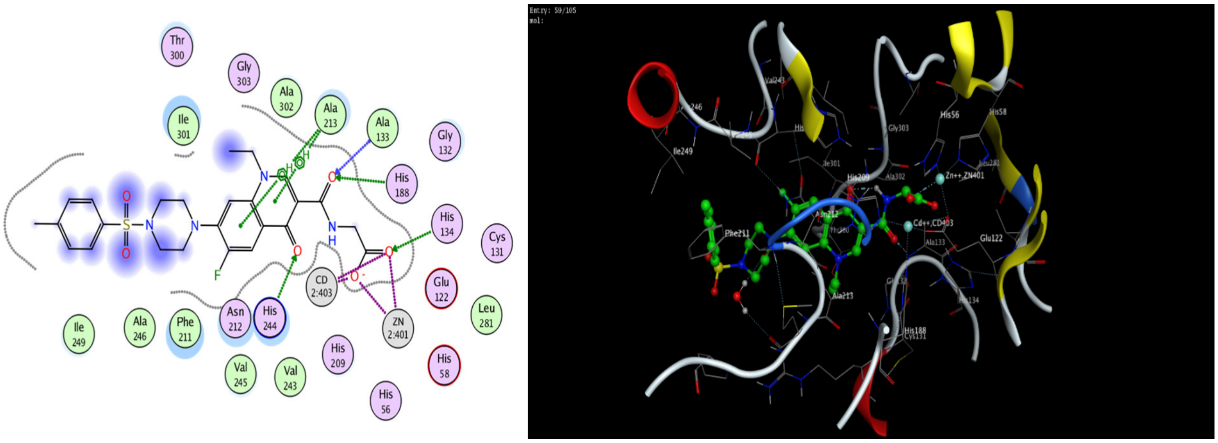

Figure S81: 2D and 3D interactions of compound **7c** with *M. smegmatis* NagA.

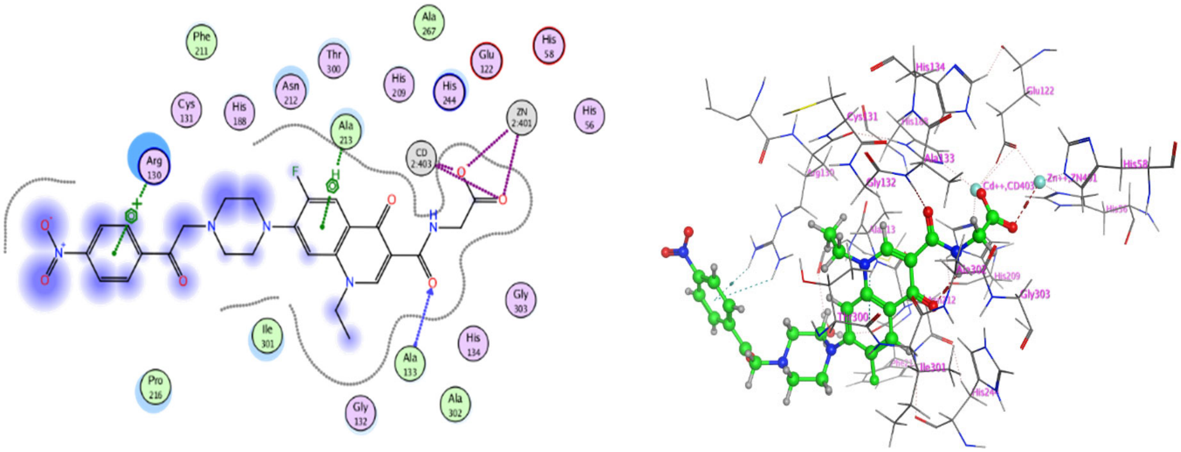

Figure S82: 2D and 3D interactions of compound **7d** with *M. smegmatis* NagA.

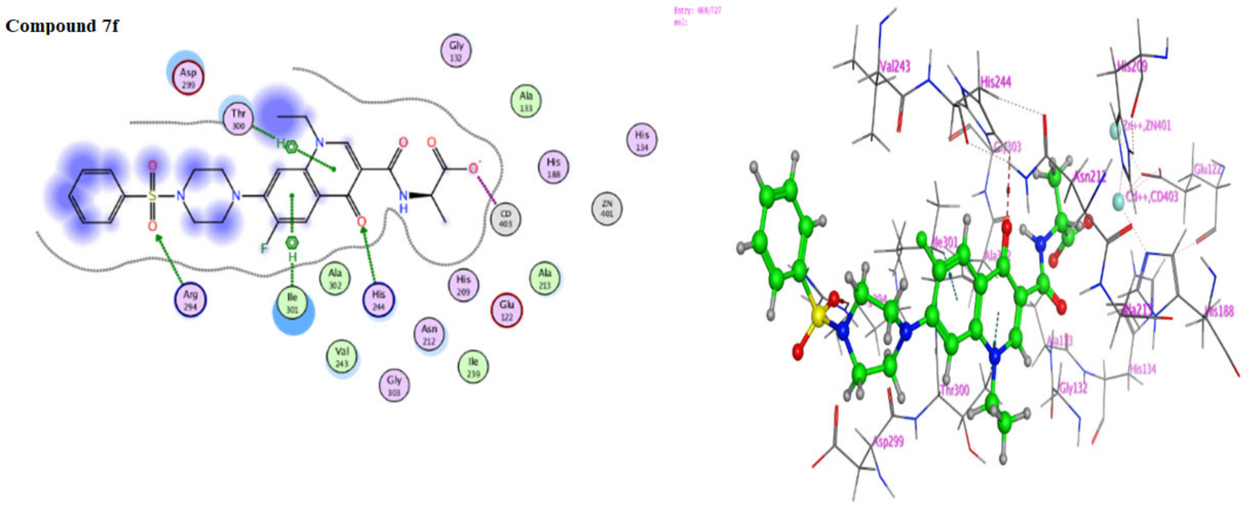

Figure S83: 2D and 3D interactions of compound **7f** with *M. smegmatis* NagA.

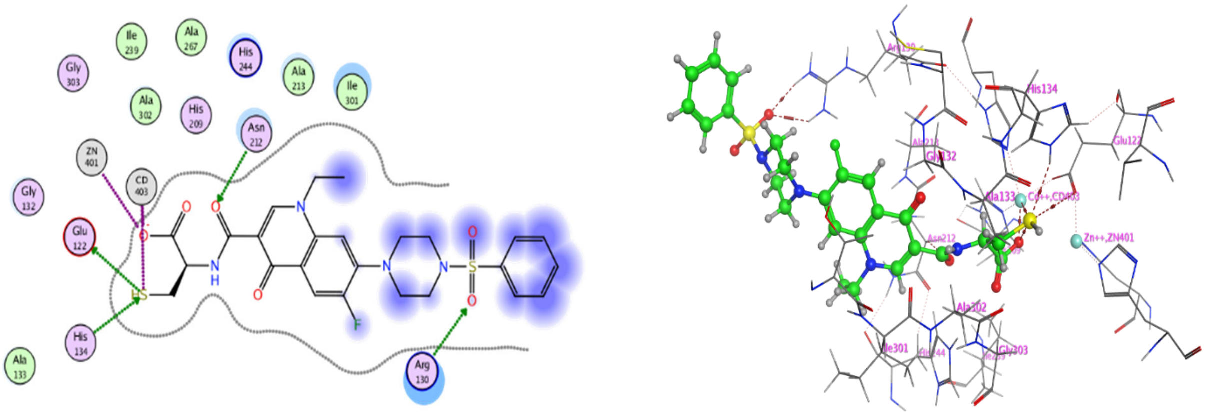

Figure S84: 2D and 3D interactions of compound **7h** with *M. smegmatis* NagA.

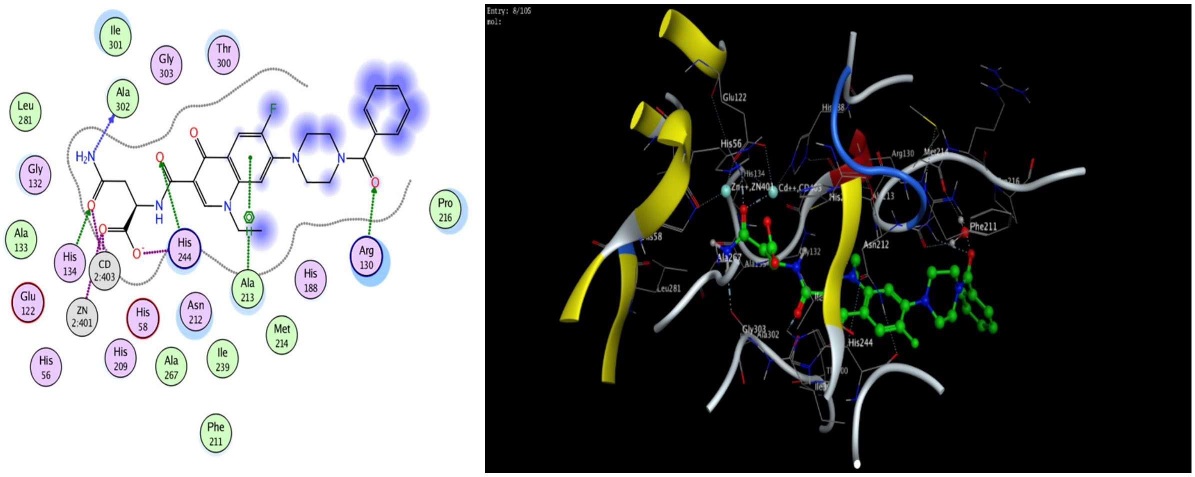

Figure S85: 2D and 3D interactions of compound **7i** with *M. smegmatis* NagA.

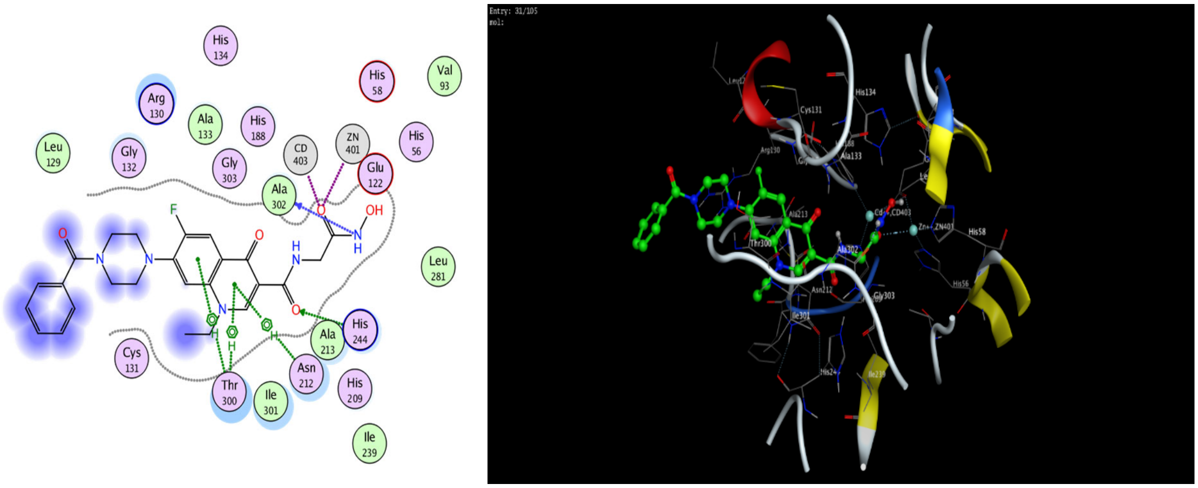

Figure S86: 2D and 3D interactions of compound **8a** with *M. smegmatis* NagA.

Text S6: Ligand-based pharmacophore modeling

Ligand-based pharmacophore modeling was performed with MOE 2020.01 using known LpxC inhibitors and their corresponding $IC_{50\%}$ values. The following criteria were considered during the selection of training set compounds: (1) all compounds have a reasonable range of experimental activities against LpxC, (2) all compounds were minimized to the most stable conformation on the MOE interface, and (3) the selected training set had variable chemical structures. A training set of 40 compounds was used to determine common pharmacophoric features using flexible alignment. The best model (score = -85.84 kcal mol^{-1}) comprised four pharmacophore features, which have mutual distance constraints between each other (**Table S4-5**, **Figure S88**), and was validated against the validation test set, identifying 13 hits (of 17 entries). These included hydrophobic moieties interacting with and occupying the hydrophobic tunnel at active site of LpxC and metal ligator groups (H-bond donor-acceptor represented by hydroxamic acid) chelating Mg^{2+}.

The set of target compounds was built and minimized to the least conformational energy and pharmacophoric search yielded 24 hits (of 24 entries), since all designed compounds possess hydrophobic side chains in addition to the quinoline core, and metal ligator groups represented by either carboxylic or hydroxamic groups, suggesting that they may be capable of interacting with LpxC. Compounds showing best hits are overlapping with all features of the generated pharmacophore query with rmsd values of 0.3331 (**4b**), 0.4511 (**4d**), 0.3456 (**5a**), 0.4094 (**5c**), 0.3521 (**7b**), 0.4564 (**7f**), 0.5608 (**7g**), 0.4539 (**8a**), and 0.5398 (**8b**) (**Figure S89**).

Ligand-based pharmacophore modeling suggested that zinc-binding groups such as hydroxamic acid, and lipophilic tails are crucial for most LpxC inhibitors. Norfloxacin showed an rmsd value of 0.9506, which was higher than most of the tested derivatives', suggesting that they have a higher probability to interact with LpxC. This is also supported by alignment of compound **5d** with the reported LpxC inhibitor **CHIR-90** (alignment score = -89.69 kcal mol^{-1} (**Figure S90**).

Table S5: Query features calculated from the aligned molecules.

Feature	Radius	Description
F1 Hyd | Aro	2.3 Å	Hydrophobic region | Aromatic ring center
F2 Aro | Hyd	2.9 Å	Aromatic ring center | Hydrophobic region
F3 ML | Acc | Don	1.5 Å	Metal ligator | H-bond acceptor | H-bond donor
F4 ML | Acc | Don	1.3 Å	Metal ligator | H-bond acceptor | H-bond donor

Table S6: Pharmacophore features with distance constraints (Å). Pharmacophore features have mutual distances between each other.

Feature	F1 Hyd | Aro	F2 Aro | Hyd	F3 ML | Acc | Don	F4 ML | Acc | Don
F1 Hyd | Aro	0 Å	4.38 Å	11.44 Å	10.70 Å
F2 Aro | Hyd	4.38 Å	0 Å	7.13 Å	6.72 Å
F3 ML | Acc | Don	11.44 Å	7.13 Å	0 Å	4.40 Å
F4 ML | Acc | Don	10.70 Å	6.72 Å	4.40 Å	0 Å

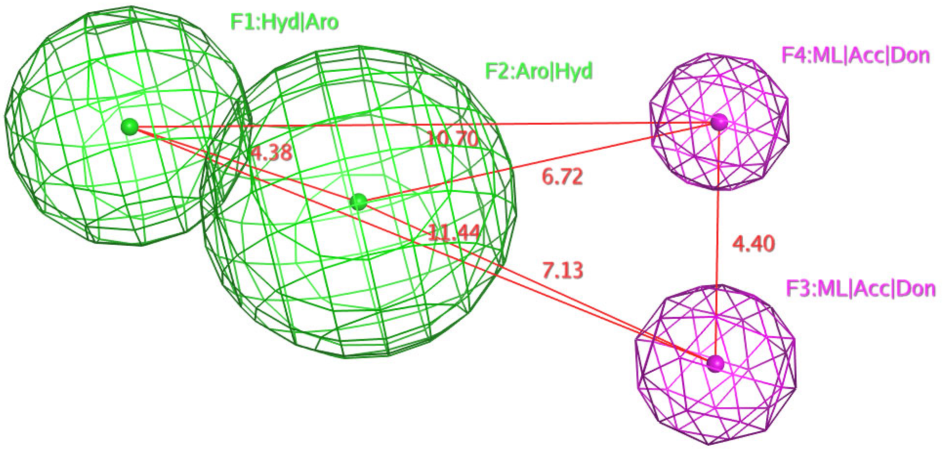

Figure S87: Query features calculated from the aligned molecules.

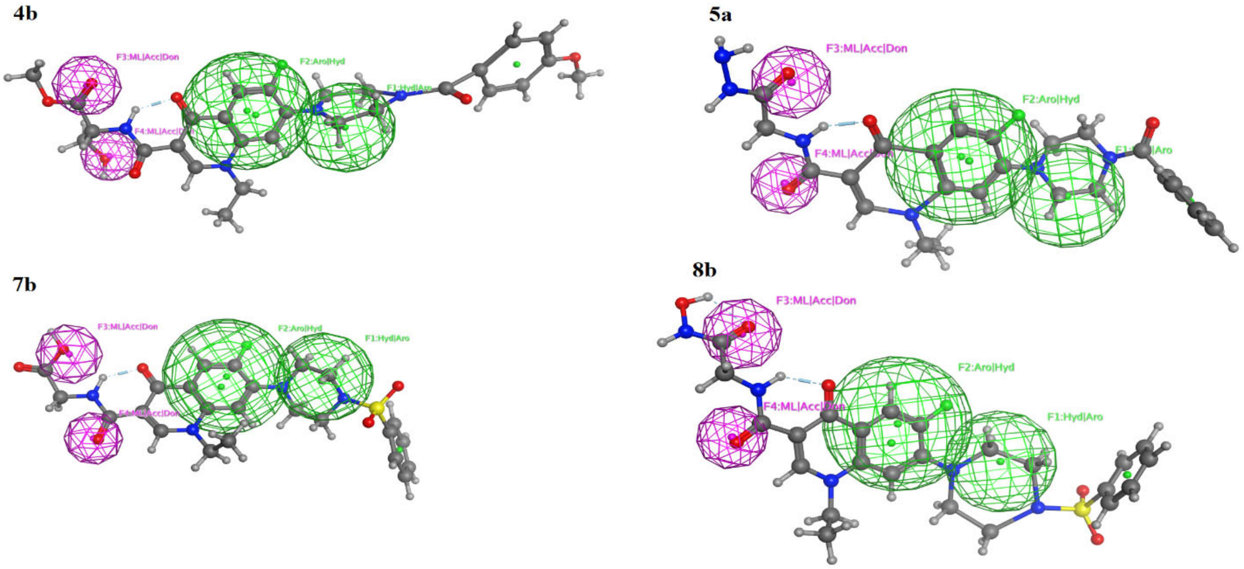

Figure S88: Overlapping of target compounds **4b**, **5a**, **7b**, and **8b** with the generated pharmacophore query.

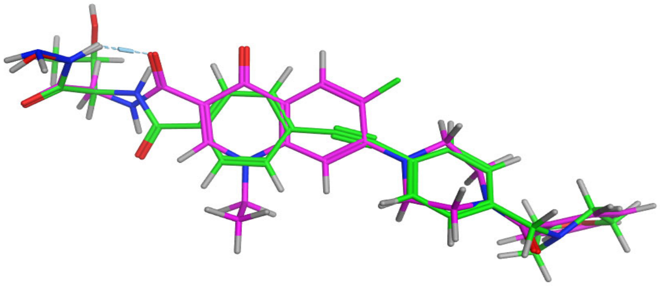

Figure S89: Alignment of **5d** compound (purple) and CHIR-90 (green).

4. Mechanism of action

Table S7: Absorbance of investigated compounds and their metal complexes.

Code	Absorbance			
	**Original**	**Zn^{2+} complex**	**Mg^{2+} complex**	**Cd^{2+} complex**
**Nor**	0.715	0.542	0.583	0.647
**5b**	0.873	0.62	0.692	0.751
**7b**	0.579	0.43	0.493	0.535
**7i**	0.853	0.641	0.712	0.78
**8a**	0.572	0.37	0.454	0.51
**8c**	0.689	0.443	0.51	0.582

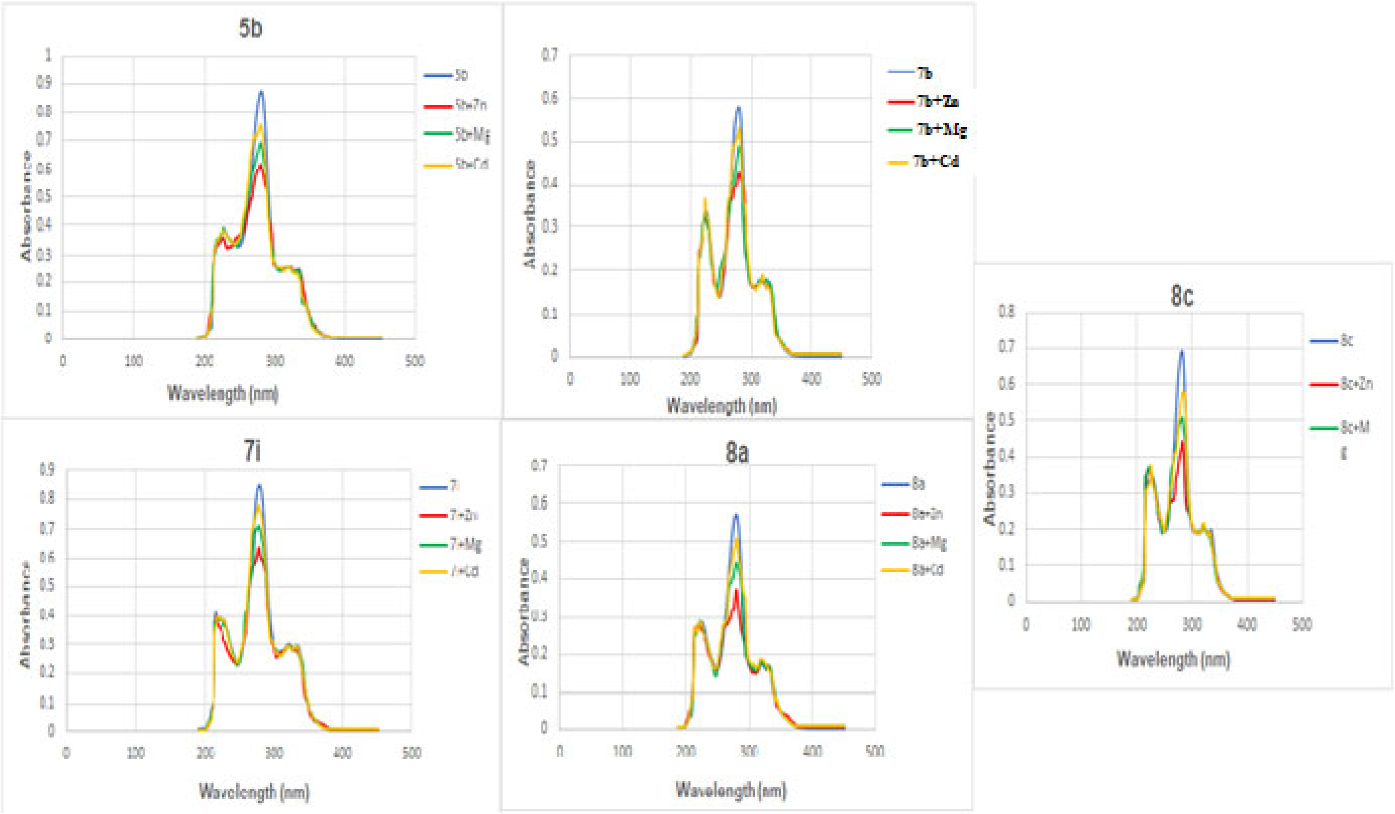

Figure S90: UV–vis absorption spectra of metal complex of compounds **5b, 7b, 7i, 8a** and **8c.**

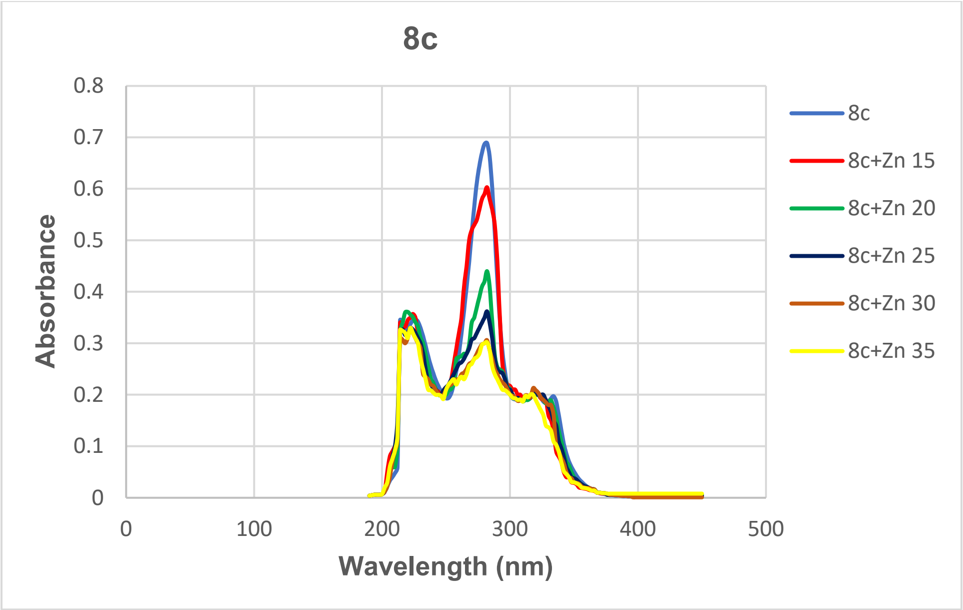

Figure S91: Molar ratio of ligand/metal in complex of compound **8c.**

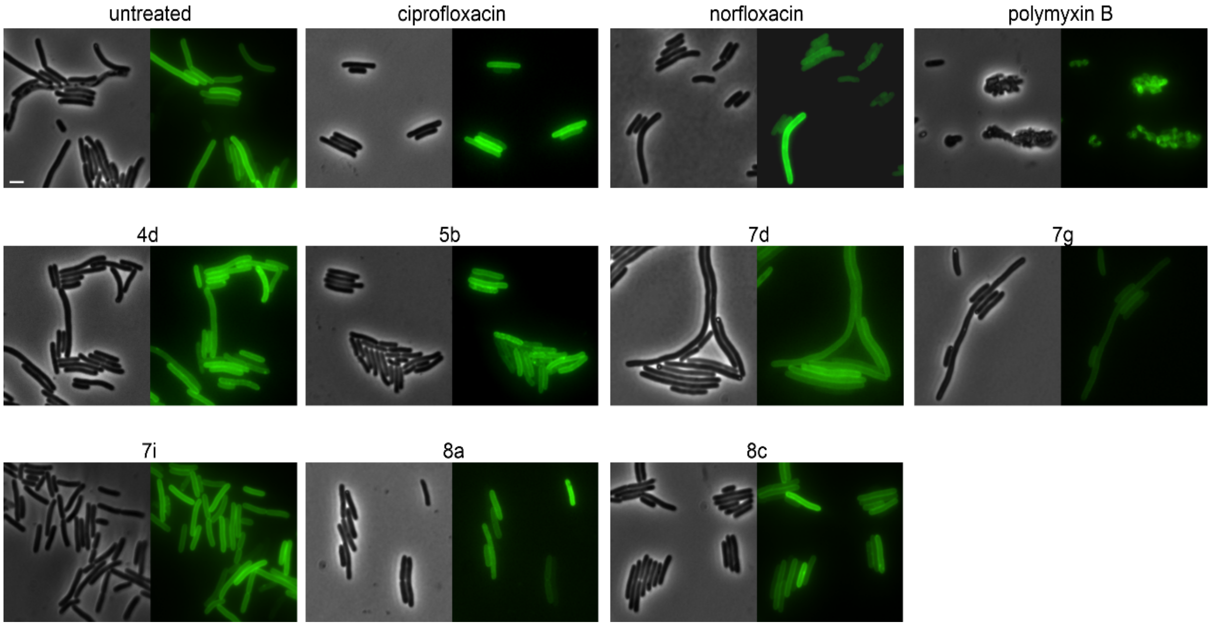

Figure S92: Fluorescence and phase contrast microscopy of *E. coli* BCB472. Cells were treated with 1xMIC of the respective compounds for 1 h prior to microscopy. Expression of NeonGreen-GlpT was induced with 10 µM IPTG for 1 h (concomitantly with antibiotic incubation). Scale bars 2 µm.

Table S8: Results summary of bacterial cytological profiling of *E. coli*. Phase contrast images indicate cell lysis. The fluorescent membrane dye FM4-64 and the GFP-tagged membrane protein GlpT report on membrane effects. The fluorescent DNA stain DAPI reports on DNA condensation. Cip = ciprofloxacin, Nor = norfloxacin, PolB = polymyxin B.

compound	concentration (μM)	phase contrast	FM4-64	GlpT	DAPI	membrane damage	gyrase inhibition
untreated	0	dark	smooth	smooth	regular	no	no
**Cip**	0.39	dark	smooth	smooth	condensed	no	yes
**Nor**	0.37	dark	smooth	smooth	condensed	no	yes
**Pol B**	0.83	light	patchy	dispersed	dispersed	yes	no
**4d**	4.47*	dark	smooth	smooth	regular	no	no
**5b**	7.21	dark	smooth	smooth	condensed	no	yes
**7d**	14.82	dark	smooth	smooth	condensed	no	yes
**7g**	15.19*	dark	smooth	smooth	regular	no	no
**7i**	7.44	dark	smooth	smooth	condensed	no	yes
**8a**	129.16*	dark	smooth	smooth	regular	no	no
**8c**	7.85	dark	smooth	smooth	condensed	no	yes

*Did not show DNA condensation at 1xMIC and was therefore additionally tested at 2x MIC.

Table S9: Results of checkerboard assays of norfloxacin derivatives combined with mupirocin. FICI values represent the average of at least two replicate experiments. Mup = muprocin, Cip, ciprofloxacin, Nor = norfloxacin, PolB = polymyxin

compound	MIC_C	$MIC_C^{checkerboard}$	FIC_C	$MIC_M^{checkerboard}$	FIC_M	FICI	outcome
Mup	64	-	-	-	-	-	-
Cip	0.125	0.031	0.25	2	0.031	0.281	synergistic
Nor	0.125	0.039	0.25	32	0.5	0.813	additive
ACHN-975	0.5	0.125	0.25	3	0.047	0.297	synergistic
PolBN	128	1	0.0078	1	0.016	0.023	synergistic
**4d**	5	0.02	0.004	0.02	0.004	1.004	additive
**5b**	4	0.016	0.004	0.016	0.004	1.004	additive
**7b**	32	0.125	0.004	0.125	0.004	1.004	additive
**7g**	64	0.25	0.004	0.25	0.004	1.004	additive
**7i**	4	0.016	0.004	0.016	0.004	1.004	additive
**8a**	0.25	0.25	0.004	0.25	0.004	1.004	additive
**8c**	4	0.016	0.004	0.016	0.004	1.004	additive

MIC_C: MIC of the test compound alone, $MIC_C^{checkerboard}$: MIC of the test compound in checkerboard assay, FIC_C: fractional inhibitory concentration of the test compound ($FIC_C=MIC_C^{checkerboard}/MIC_C$), MIC_M: MIC of mupirocin alone (64 µg/mL), $MIC_M^{checkerboard}$: MIC of mupirocin in checkerboard assay, FIC_M: fractional inhibitory concentration of the test compound ($FIC_M=MIC_M^{checkerboard}/MIC_M$), FICI: fractional inhibitory concentration index = FIC_C+FIC_M.

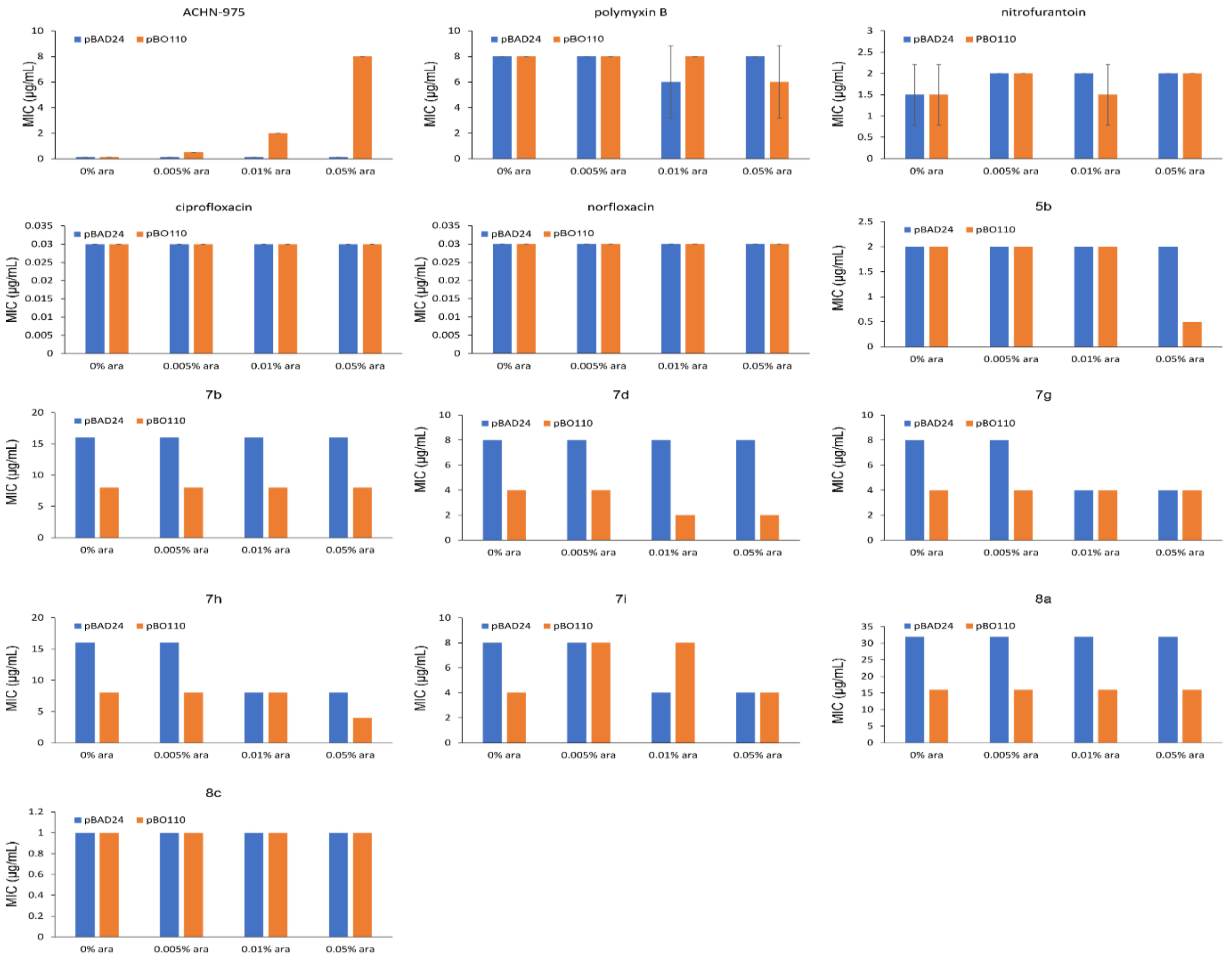

Figure S93: Effects on LpxC. *E. coli* BL21 DE03 carrying pBO110, expressing LpxC from the arabinose-inducible PBAD promoter, was grown in presence of increasing arabinose concentrations. Overexpression of LpxC leads to accumulation of lipid A in the cell membrane, which is toxic for *E. coli*. Inhibition of LpxC activity mitigates this effect. As controls for the presence of sugar, parallel cultures were grown in the presence of glucose. *E. coli* BL21 DE03 carrying pBAD24 was included as empty vector control.

Table S10: Minimal inhibitory concentrations against *B. subtilis* DSM402. Cip = ciprofloxacin, Nor = norfloxacin, Van = vancomycin, Fos = fosfomycin, Tun = tunicamycin.

compound	MIC (μM)	compound	MIC (μM))
**Cip**	3.01	**7c**	**1.88**
**Nor**	18.11	**7d**	51.89
**Van**	0.68	**7e**	4.04
**Fos**	72.43	**7f**	3.76
**Tun**	19.58	**7g**	**1.9**
**4d**	28.64	**7h**	**1.77**
**5b**	28.85	**7i**	5.58
**7a**	**1.56**	**8a**	8.07
**7b**	**1.45**		

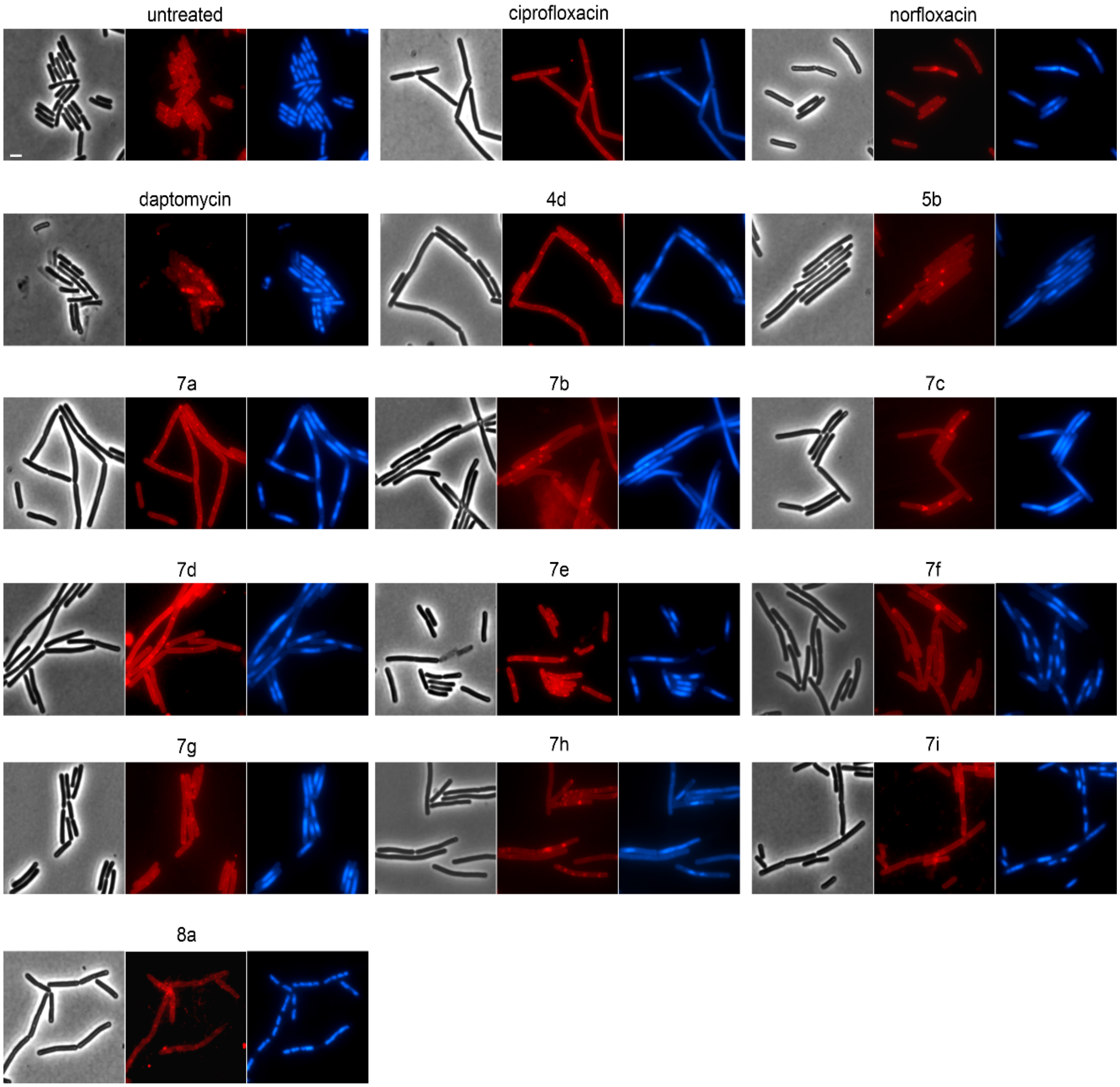

Figure S94: Bacterial cytological profiling of *B. subtilis*. Fluorescence and phase contrast microscopy of *B. subtilis* DSM402. Cells were treated with 1x MIC of the respective compounds for 1 h prior to staining with FM4-64 (membrane, red) and DAPI (nucleoid, blue). Scale bars 2 µm.

Table S11: Results summary of bacterial cytological profiling of *B. subtilis*. Phase contrast images indicate cell lysis. The fluorescent membrane dye FM4-64 reports on membrane effects. The fluorescent DNA stain DAPI reports on DNA condensation. Cip = ciprofloxacin, Nor = norfloxacin, Dap = daptomycin.

**compound**	**concentration (µM)**	**phase contrast**	**FM4-64**	**DAPI**	**membrane damage**	**gyrase inhibition**
untreated		dark	smooth	regular	no	no
Cip	3.01	dark	spotty	condensed	yes	yes
Nor	18.11	dark	patchy	condensed	yes	yes
Dap	0.62	light	patchy	regular	yes	no
**4d**	28.64*	dark	smooth	slightly condensed	no	possibly
**5b**	28.85	dark	patchy	condensed	yes	yes
**7a**	1.56	dark	smooth	condensed	no	yes
**7b**	1.45	dark	patchy	condensed	yes	yes
**7c**	1.88	dark	patchy	condensed	yes	yes
**7d**	51.89	dark	spotty	condensed	yes	yes
**7e**	4.04	dark	spotty	condensed	yes	yes
**7f**	3.76*	dark	smooth	condensed	no	yes
**7g**	1.9	dark	smooth	condensed	no	yes
**7h**	1.77*	dark	patchy	condensed	yes	yes
**7i**	5.58	dark	smooth	condensed	no	yes
**8a**	8.07	grey	spotty	regular	yes	no

*Did not show DNA condensation at 1xMIC and was therefore additionally tested at 2x MIC.

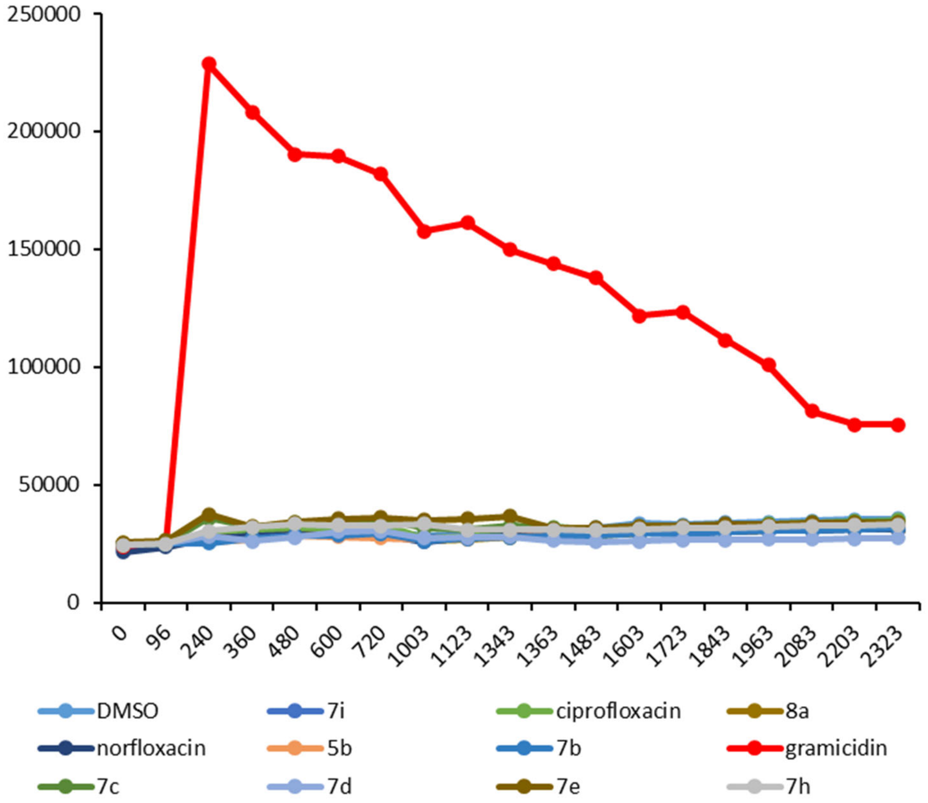

Figure S95: Effects of the membrane potential in *B. subtilis* DSM 402. Bacteria were grown until early log phase in Muller Hinton broth and stained with the membrane-potentiometric fluorescence probe DiSC(3)5. The dye binds to polarized membranes and self-quenches. Upon depolarization, the dye is released leading to de-quenching and an increased fluorescence signal. Gramicidin (1 µg/mL), which forms a transmembrane ion channel, was used as a positive control.

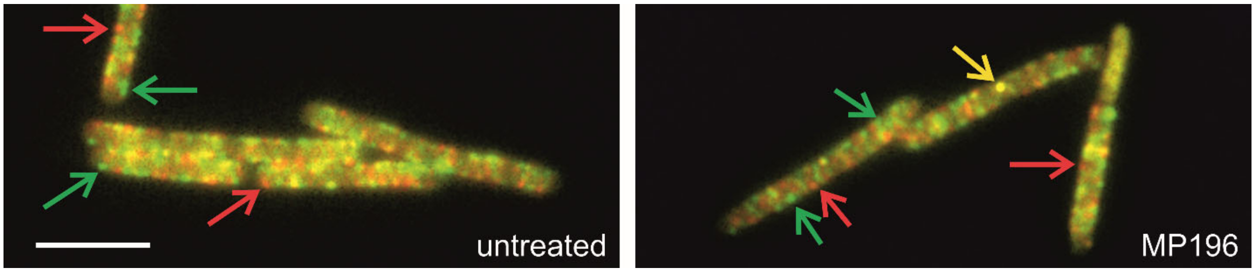

Figure S96: MreB mobility after treatment with MP196. MP196 is an antimicrobial peptide that indirectly interferes with cell wall synthesis by delocalizing the lipid II synthase MurG from the cell membrane [47]. Such indirect cell wall synthesis inhibition tests positive in the acetic acid/methanol fixation [47] but not in the MreB mobility assay. Red and green arrows indicate exemplary mobile MreB foci. Yellow arrow indicates a static cluster caused by membrane reorganization by MP196.

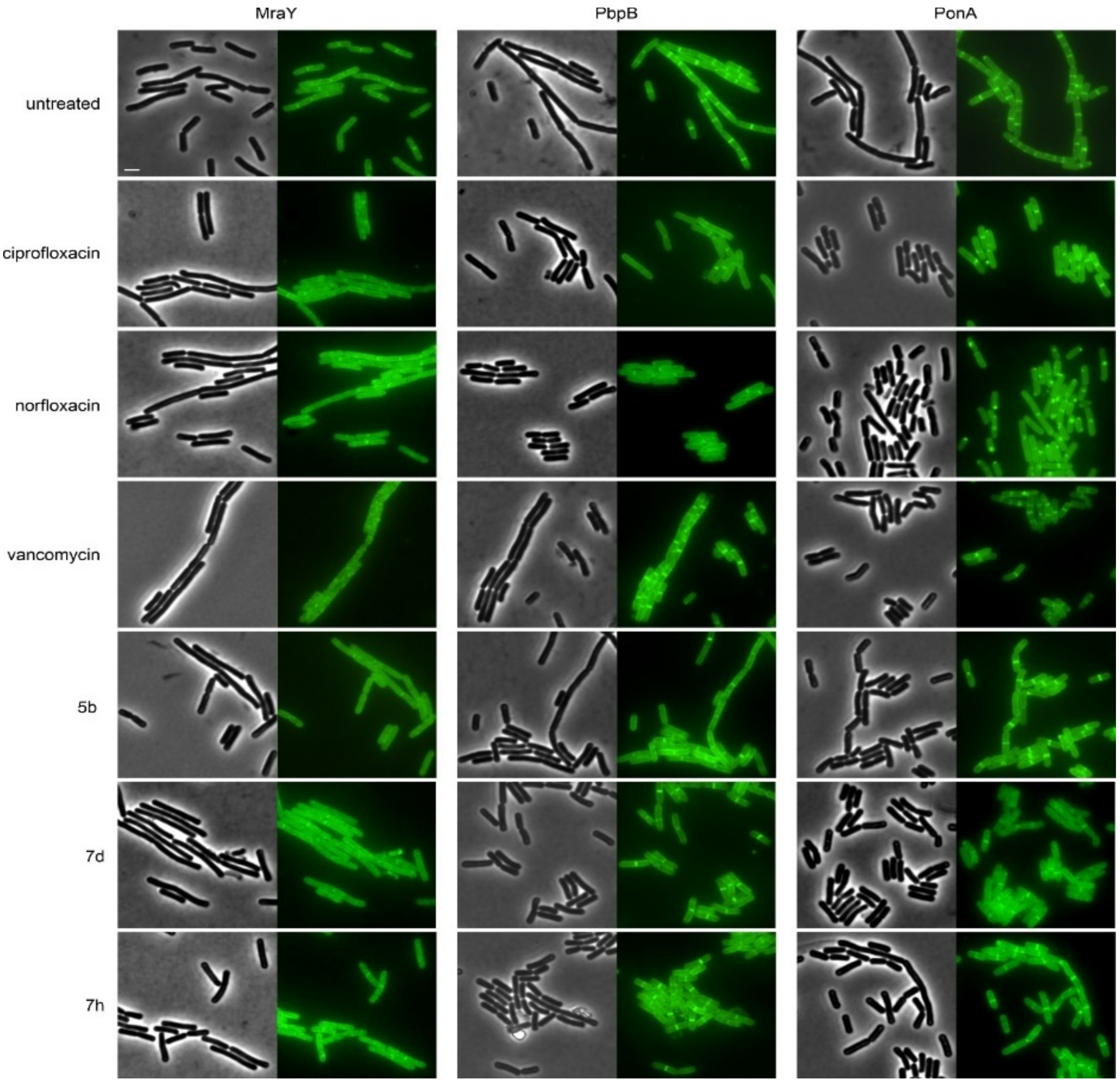

Figure S97: Fluorescence and phase contrast microscopy of *B. subtilis* TNVS284 (MraY-msfGFP) **(A)**, TNVS45 (PonA-mGFP) **(B)**, and EKB46 (msfGFP-PbpB) **(C)**. Expression of the respective GFP fusion proteins was induced with 0.1% xylose. Cells were treated with 1xMIC of the respective compounds for 30 min (vancomycin) or 1 h (all other compounds) prior to microscopy. Scale bar 2 µm.

5. Methods

Text S7: Chemistry

All reagents and solvents were of commercially available reagent grade quality and were used without further purification. IR spectra were recorded on a Nicolet® iS5 FT-IR Spectrometer. ^{1}HNMR and ^{13}CNMR spectra were recorded on a Bruker® Advance III (400 MHz). Chemical shifts are reported in δ parts per million (ppm) using TMS as an internal standard and coupling constants (J) are expressed in hertz (Hz). Abbreviations indicating multiplicity were used as follows: s = singlet, bs = broad singlet, d = doublet, t = triplet, q = quartet and m = multiplet. Low-resolution mass spectra (LRMS): were recorded on a JEOL® mass spectrometer. Elemental analysis was performed on a Perkin Elmer 2400 CHN elemental analyzer, and values were within ± 0.4% of the theoretical values. Melting points were measured on a Stuart® SMP10 melting point apparatus. Purity of the final compounds was ≥95%.

Synthesis of *N*-acyl, alkyl and phenacylnorfloxacin derivatives (2a-f).

N-acyl, sulphonyl, alkyl, or phenacyl norfloxacin derivatives were synthesized as reported previously [21, 22].

7-(4-benzoyl)piperazin-1-yl)-1-ethyl-6-fluoro-1,4-dihydro-4-oxoquinoline-3-carboxylic acid 2a.

Yield= 3.5 g (88%); white powder, mp:266-268 ºC (reported mp: 263-264 ºC)[23]. ^{1}H NMR 400 MHz ($CDCl_3$) δ (ppm): 15 (s, 1H), 8.65 (s, 1H), 8.03 (d, $J_{H\text{-}F}$ =12.9 Hz, 1H), 7.45 (m, 5H), 6.86 (d, $J_{\text{H-F}}$ = 6.6 Hz, 1H), 4.33 (q, J = 7.2 Hz, 2H), 3.88 (br. m, 4H), 3.34 (br. m, 4H), 1.58 (t, J = 5.1 Hz, 3H).

7-(4-(4-methoxybenzoyl)piperazin-1-yl)-1-ethyl-6-fluoro-1,4-dihydro-4-oxoquinoline-3-carboxylic acid 2b.

Yield= 3.45 g (81%); white powder, mp:247-249 ºC (No reported mp)[24]. ^{1}H NMR 400 MHz (DMSO-d_6) δ (ppm): 15.32 (s, 1H), 8.96 (s, 1H), 7.95 (d, $J_{H\text{-}F}$ =12.9 Hz,1H), 7.44 (d, J=8.4 Hz, 2H), 7.01 (d, J=8.4 Hz, 2H), 7.22 (d, $J_{\text{H–F}}$ = 6.6 Hz, 1H), 4.59 (q, J = 7.2 Hz, 2H), 3.8 (s, 3H), 3.72 (br. m, 4H), 3.36 (br. m, 4H) ,1.41 (t, J = 5.1 Hz, 3H).

7-(4-phenylsulphonylpiperazin-1-yl)-1-ethyl-6-fluoro-1,4-dihydro-4-oxoquinoline-3-carboxylic acid 2c.

Yield= 3.85 g (89%); white powder, mp:290-292 ºC (no reported mp)[25]. ^{1}H NMR 400 MHz (DMSO-d$_6$) δ (ppm): 15.2 (s, 1H), 8.94 (s, 1H), 7.89 (d, $J_{H\text{-}F}$ =12.9 Hz,1H), 7.82-7.66 (m, 5H), 7.19 (d, $J_{H\text{–}F}$ = 6.6 Hz, 1H), 4.57 (q, J = 7.2 Hz, 2H), 3.4 (br. m, 4H), 3.1 (br. m, 4H), 1.37 (t, J = 5.1 Hz, 3H). ^{13}C NMR 100 MHz (DMSO-d$_6$): 176.3, 166.2, 151.8, 148.8, 144.8, 137.1, 134.8, 133.5, 129.4, 127.8, 120.1, 111.4, 107.4, 106.7, 67.0, 49.2, 45.9, 14.8.

7-(4-(4-tolylsulphonyl)piperazin-1-yl)-1-ethyl-6-fluoro-1,4-dihydro-4-oxoquinoline-3-carboxylic acid 2d.

Yield= 4 g (90%); white powder, mp:277-279 ºC (no reported mp)[25]. ^{1}H NMR 400 MHz (DMSO-d6) δ (ppm): 15.24 (s, 1H), 8.93 (s, 1H), 7.88 (d, $J_{H\text{-}F}$ =12.9 Hz,1H), 7.67 (d, J= 8 Hz , 2H), 7.48 (d, J= 8 H , 2H), 7.18 (d, $J_{\mathrm{H–F}}$ = 6.6 Hz, 1H), 4.56 (q, J = 7.2 Hz, 2H), 3.39 (br. m, 4H), 3.07 (br. m, 4H) , 2.41 (s, 3H), 1.37 (t, J = 5.1 Hz, 3H).

1-ethyl-6-fluoro-1,4-dihydro-7-(4-(4-chlorobenzyl)piperazin-1-yl)-4-oxoquinoline-3-carboxylic acid 2e.

Yield= 2.65 g (61%); white powder, mp:248-250 ºC. ^{1}H NMR 400 MHz (DMSO-d$_6$) δ (ppm): 15.15 (br. s, 1H), 8.87 (s, 1H), 7.83 (d, $J_{H\text{-}F}$ = 12.9 Hz, 1H), 7.34-7.28 (m, 4H), 7.09 (d, $J_{H\text{-}F}$ = 6.6 Hz, 1H), 4.5 (q, J = 4.8, 9.5 Hz, 2H), 3.48 (s, 2H), 3.34 (br. m, 4H), 2.5 (br. m, 4H), 1.32 (t, J = 5.1 Hz, 3H). ^{13}C NMR 100 MHz (DMSO-d$_6$): 176.6, 166.2, 154.0, 151.5, 148.8, 145.5, 137.4, 131.5, 130.8, 128.1, 119.4, 111.4, 107.4, 106.0, 60.9, 52.1, 49.6, 49.1, 14.3. Anal. Calcd for $C_{23}H_{23}ClFN_3O_3$: C, 62.23; H, 5.22; N, 9.47. Found: C, 62.47; H, 5.38; N, 9.70.

1-ethyl-6-fluoro-1,4-dihydro-7-(4-(4-nitrophenacyl)piperazin-1-yl)-4-oxoquinoline-3-carboxylic acid 2f.

Yield= 3.64 g (80%); yellow powder, mp:242-244 ºC. ^{1}H NMR 400 MHz (DMSO-d$_6$) δ (ppm): 15.38 (br. s, 1H), 8.96 (s, 1H), 8.35 (d, J = 8.7 Hz, 2H), 8.25 (d, J = 8.7 Hz, 2H), 7.94 (d, $J_{H\text{-}F}$ = 12.9 Hz, 1H), 7.21 (d, $J_{H\text{-}F}$ = 6.6 Hz, 1H), 4.6 (q, J = 4.8, 9.5 Hz, 2H), 4.04 (s, 2H), 3.48 (br. m, 4H), 3.29 (br. m, 4H), 1.41 (t, J = 5.1 Hz, 3H). ^{13}C NMR 100 MHz (DMSO-d$_6$): 187.5, 177.7, 167.4, 153.7, 150.3, 149.0, 146.0, 143.6, 137.9, 130.2, 124.2, 116.2, 110.8, 107.5, 55.5, 52.7, 50.0, 49.7, 15.0. Anal. Calcd for $C_{24}H_{23}FN_4O_6$: C, 59.75; H, 4.81; N, 11.61. Found: C, 59.91; H, 5.05; N, 11.89.

Synthesis of *N*-acyl and alkylnorfloxacin amino acid ester derivatives (4a-f).

A suspension *N*-acyl or alkyl norfloxacin derivatives (0.5 g) in dichloromethane (20 ml), was treated with triethylamine (2 equivalents) and stirred in an ice bath for 10 minutes. Ethyl

chloroformate (1.5 equivalents) was added, and the reaction mixture stirred in an ice bath for an hour at 0-5 °C. The solvent and excess reagents were then evaporated under reduced pressure and the resulting solid mixed anhydride was dissolved in an appropriate amount of dichloromethane. Then, the respective amino acid ester hydrochloride (2 equivalents) was added, and the reaction mixture was stirred at room temperature for 24 hours. Progress of the reaction was followed up by TLC monitoring (Mobile phase: 9.7:0.3chloroform/methanol). After total consumption of the reactants, the organic layer was washed with a saturated solution of $NaHCO_3$ (2 x 25 ml) and then water (2 x 25 ml), and dried over Na_2SO4 anhydrous. Filtration and evaporation of the solvent under vacuum afforded the products. Purification of ester compounds was achieved through crystallization from ethanol.

Ethyl 2-(7-(4-benzoylpiperazin-1-yl)-1-ethyl-6-fluoro-4-oxo-1,4-dihydroquinoline-3-carboxamido)acetate 4a.

Yield= 0.36 g (60%); white powder, mp:221-223 °C. IR (KBr): 3451(NH str), 3050(aromatic C-H str), 2930(aliphatic C-H str), 1748(ester C=O str), 1655(carbamide C=O str), 1629(quinolone C=O str), 1202(C-O), 1009(C-N) cm^{-1}. ^{1}H NMR 400 MHz (DMSO-d_6) δ (ppm): 10.24 (t, *J*= 6 Hz, 1H), 8.78 (s, 1H), 7.92 (d, J_{H-F} = 12.9 Hz, 1H), 7.5-7.46 (m, 5H), 7.16 (d, $J_{H–F}$ = 6.6 Hz ,1H), 4.5 (q, *J* = 4.8, 9.5 Hz, 2H), 4.13 (m, 4H), 3.7 (br. m, 4H), 3.3 (br. m, 4H) ,1.39 (t, *J* = 5.1 Hz, 3H), 1.21 (t, *J* = 5.1 Hz, 3H). ^{13}C NMR 100 MHz (DMSO-d_6): 174.4, 170.4, 169.7, 165.0, 152.0, 148.0,145.0, 137.3, 136.3, 130.2, 129.0, 128.0, 122.2, 111.9, 110.5, 106.9, 61.0, 50.3, 49.0, 41.8, 41.3, 15.0, 14.6. Anal. Calcd for $C_{27}H_{29}FN_4O_5$: C, 63.77; H, 5.75; N, 11.02; Found: C, 64.01; H, 5.89; N,11.26. LRMS for $[C_{24}H_{23}FN_4O_4]^+$ $[M]^+$ calculated: 508.21 found: 508.16.

Methyl-7-(4-(4-methoxybenzoyl)piperazin-1-yl)-1-ethyl-6-fluoro-2-hydroxymethyl-4-oxo-1,4-dihydroquinoline-3-carboxamide)acetate 4b.

Yield= 0.41 g (67%); white powder, mp:132-134 °C. ^{1}H NMR 400 MHz (DMSO-d_6) δ (ppm): 10.5 (t, *J*= 6 Hz, 1H), 8.78 (s, 1H), 7.91 (d, J_{H-F} = 12.9 Hz, 1H), 7.44 (d, *J* = 8.4 Hz, 2H), 7.15 (d, $J_{H–F}$ = 6.6 Hz ,1H),7.02 (d, *J* = 8.4 Hz, 2H), 5.24 (t, *J* = 5.2 Hz, 1H), 4.62 (m, 1H), 4.49 (q, J = 4.8, 9.5 Hz, 2H), 3.88-3.66 (br. m, 12H), 3.3 (br. m, 4H), 1.39 (t, *J* = 5.1 Hz, 3H). ^{13}C NMR 100 MHz (DMSO-d_6): 174.3, 171.3, 169.2, 164.2, 160.6, 151.5, 147.5, 144.8, 136.2, 129.1, 127.8, 121.8, 113.7, 111.8, 110.1, 106.4, 61.6, 55.4, 55.2, 54.2, 51.5, 49.9, 48.5, 14.4. Anal. Calcd for $C_{28}H_{31}FN_4O_7$: C, 60.64; H, 5.63; N, 10.10; Found: C, 60.85; H, 5.79; N, 10.37.

Ethyl 2-(7-(4-phenylsulphonylpiperazin-1-yl)-1-ethyl-6-fluoro-4-oxo-1,4-dihydroquinoline-3-carboxamido)acetate 4c.

Yield= 0.35 g (59%); white powder, mp:185-187 °C. ^{1}H NMR 400 MHz (DMSO-d$_6$) δ (ppm): 10.2 (t, *J*= 6 Hz, 1H), 8.76 (s, 1H), 7.88-7.67 (m, 6H), 7.12 (d, $J_{H\text{–}F}$ = 6.6 Hz ,1H), 4.48 (q, *J* = 4.8, 9.5 Hz, 2H), 4.11 (q , *J*=7.2Hz ,4H), 3.37 (br. m, 4H), 3.09 (br. m, 4H), 1.35 (t, *J* = 5.1 Hz, 3H), 1.2 (t, *J* = 5.1 Hz, 3H). ^{13}C NMR 100 MHz (DMSO-d$_6$): 174.0, 169.9, 164.5, 151.5, 147.5, 143.8, 136.8, 135.1, 133.5, 129.8, 127.8, 121.8, 111.4, 110.4, 106.4, 60.6, 49.2, 48.5, 45.9, 41.2, 14.4, 14.1. Anal. Calcd for $C_{26}H_{29}FN_4O_6S$: C, 57.34; H, 5.37; N, 10.29; S, 5.89 Found: C, 57.49; H, 5.60; N, 10.43; S, 5.97.

Ethyl 2-(7-(4-tolylsulphonylpiperazin-1-yl)-1-ethyl-6-fluoro-4-oxo-1,4-dihydroquinoline-3-carboxamido)acetate 4d.

Yield= 0.38 g (64%); white powder, mp: 238-240 °C. ^{1}H NMR 400 MHz (DMSO-d$_6$) δ (ppm): 10.21 (t, *J*= 6 Hz, 1H), 8.77 (s, 1H), 7.87 (d, $J_{H\text{-}F}$ = 12.9 Hz, 1H), 7.69 (d, *J* = 8.4 Hz, 2H), 7.49 (d, *J* = 8.4 Hz, 2H), 7.13 (d, $J_{H\text{–}F}$ = 6.6 Hz ,1H), 4.49 (q, *J* = 4.8, 9.5 Hz, 2H), 4.12 (q, *J*=7.2Hz ,4H), 3.35 (br. m, 4H), 3.07 (br. m, 4H), 2.42 (s, 3H), 1.36 (t, *J* = 5.1 Hz, 3H), 1.21 (t, *J* = 5.1 Hz, 3H). ^{13}C NMR 100 MHz (DMSO-d$_6$): 174.3, 170.0, 164.6, 153.9, 151.2, 147.5, 143.8, 136.5, 131.8, 129.8, 127.8, 122.1, 111.4, 110.0, 106.5, 60.4, 49.0, 48.4, 45.7, 40.8, 21.0, 14.4, 14.1. Anal. Calcd for $C_{27}H_{31}FN_4O_6S$: C, 58.05; H, 5.59; N, 10.03; S, 5.74. Found: C, 58.23; H, 5.71; N, 10.27; S, 5.83.

Ethyl 2-(7-(4-(4-chlorobenzyl)piperazin-1-yl)-1-ethyl-6-fluoro-4-oxo-1,4-dihydroquinoline-3-carboxamido)acetate 4e.

Yield= 0.43 g (72%); white powder, mp:227-229 °C. ^{1}H NMR 400 MHz (DMSO-d$_6$) δ (ppm): 10.24 (t, *J*= 6 Hz, 1H), 8.78 (s, 1H), 7.9 (d, $J_{H\text{-}F}$ = 12.9 Hz, 1H), 7.68-7.38 (m, 4H), 7.12 (d, $J_{H\text{–}F}$ = 6.6 Hz ,1H), 4.5 (q, *J* = 4.8, 9.5 Hz, 2H), 4.13 (m, 4H), 3.57 (s, 2H), 3.29 (br. m, 4H), 2.59 (br. m, 4H) , 1.39 (t, *J* = 5.1 Hz, 3H), 1.21 (t, *J* = 5.1 Hz, 3H). ^{13}C NMR 100 MHz (DMSO-d$_6$): 174.3, 169.9, 164.6, 151.4, 147.5, 145.9, 137.1, 136.5, 131.6, 130.7, 128.2, 122.1, 111.4, 110.0, 106.0, 60.9, 60.5, 52.2, 49.6, 48.4, 40.8, 14.4, 14.1. Anal. Calcd for $C_{27}H_{30}ClFN_4O_4$: C, 61.30; H, 5.72; Cl, 6.70; N, 10.59; Found: C, 61.04; H, 5.89; N, 10.76.

Methyl-7-(4-phenylsulphonylpiperazin-1-yl)-1-ethyl-6-fluoro-2-hydroxymethyl-4-oxo-1,4-dihydroquinoline-3-carboxamide)acetate 4f.

Yield= 0.30 g (49%);white powder , mp:163-165 °C. ^{1}H NMR 400 MHz (DMSO-d_6) δ (ppm): 10.48 (t, *J*= 6 Hz, 1H), 8.76 (s, 1H), 7.90-7,69 (m, 6H), 7.13 (d, $J_{H\text{–}F}$ = 6.6 Hz ,1H), 5.23 (t, *J* = 5.2 Hz, 1H), 4.61 (m, 1H), 4.48 (q, *J* = 4.8, 9.5 Hz, 2H), 3.89-3.60 (br. m, 6H), 3.36 (br. m, 4H), 3.10 (s, 3H), 1.36 (t, *J* = 5.1 Hz, 3H). ^{13}C NMR 100 MHz (DMSO-d_6): 174.5, 171.6, 164.7, 151.7, 148.1, 144.3, 136.8, 135.2, 134.0, 130.0, 128.1, 122.5, 112.0, 110.6, 106.9, 62.0, 54.5, 52.5, 49.4, 48.9, 46.2, 14.8. Anal. Calcd for $C_{26}H_{29}FN_4O_7S$: C, 55.70; H, 5.21; N, 9.99; S, 5.72; Found: C, 55.48; H, 5.09; N, 10.13; S, 5.57.

Synthesis of N-acyl or alkylnorfloxacin amino acid hydrazide derivatives (5a-e).

To a stirred solution of *N*-acyl norfloxacin amino acid esters **4a-e** (0.25 g) in methanol (10 ml), 3 equivalents of hydrazine hydrate (99%) were added. The reaction mixture was heated under reflux overnight (TLC monitoring using 9.7:0.3 chloroform/methanol as mobile phase). The volume of the solution was reduced to 1/3 by evaporation under vacuum and then cooled in a refrigerator overnight. The formed precipitate was collected by filtration and washed with cold methanol and water and dried. Purification of the obtained hydrazides was achieved by recrystallization using ethanol and then trituration using diethyl ether.

1-ethyl-6-fluoro-*N*-(1-hydrazinyl-1-oxoethyl)-7-(4-benzoylpiperazin-1-yl)-4-oxo-1,4-dihydroquinoline-3-carboxamide 5a.

Yield= 0.13 g (53%); white powder, mp: 171-173 °C. IR (KBr): 3316(NH str), 3051(aromatic C-H str), 2922(aliphatic C-H str), 1693(hydrazide C=O str), 1669(carbamidic C=O str), 1610(quinolone C=O str), 1254(C-O), 1011(C-N) cm^{-1}. ^{1}H NMR 400 MHz (DMSO-d_6) δ (ppm): 10.19 (t, J= 6 Hz, 1H),9.13 (s, 1H), 8.76 (s, 1H), 7.9 (d, $J_{H\text{-}F}$ = 12.9 Hz, 1H), 7.50-7.45 (m, 5H), 7.14 (d, $J_{H\text{–}F}$ = 6.6 Hz ,1H), 4.48 (q, *J* = 4.8, 9.5 Hz, 2H), 4.23 (s, 2H), 3.93 (d , *J*=7.2Hz ,2H), 3.7 (br. m, 4H), 2.5 (br. m, 4H) ,1.38 (t, *J* = 5.1 Hz, 3H). ^{13}C NMR 100 MHz (DMSO-d_6): 174.3, 169.2, 168.2, 164.2, 151.5, 147.5, 144.5, 136.4, 135.8, 129.8, 127.8, 122.1, 111.8, 110.4, 106.4, 49.8, 48.4, 40.8, 14.8. Anal. Calcd for $C_{25}H_{27}FN_6O_4$: C, 60.72; H, 5.50; N, 16.99; Found: C, 60.96; H, 5.73; N, 17.16. LRMS for $[C_{24}H_{23}FN_4O_4]^+$ $[M]^+$ calculated: 494.21 found: 494.14.

1-ethyl-6-fluoro-*N*-(1-hydrazinyl-3-hydroxy-1-oxopropan-2-yl)-7-(4-(4-methoxybenzoyl)piperazin-1-yl)-4-oxo-1,4-dihydroquinoline-3-carboxamide 5b.

Yield= 0.14 g (56%); greenish white powder, mp: 95-97 °C. ^{1}H NMR 400 MHz (DMSO-d_6) δ (ppm): 10.31 (d, *J*= 8 Hz, 1H), 9.19 (s,1H), 8.76 (s, 1H),7.9 (d, $J_{H\text{-}F}$ = 12.9 Hz, 1H), 7.44 (d, *J* = 8.4 Hz, 2H), 7.15 (d, $J_{H\text{–}F}$ = 6.6 Hz ,1H), 7.02 (d, J = 8.4 Hz, 2H), 5.01 (t, *J* = 5.2 Hz, 1H), 4.49 (m

,3 H, 2H), 4.24 (s, 2H), 3.83-3.56 (br. m, 9H), 3.3 (br. m, 4H), 1.38 (t, J = 5.1 Hz, 3H). ^{13}C NMR 100 MHz (DMSO-d$_6$): 174.3, 169.2, 163.9, 160.2, 153.6, 151.2, 147.5, 144.1, 136.8, 129.1, 127.4, 121.8, 113.7, 111.4, 110.4, 106.4, 61.9, 55.2, 53.6, 50.2, 48.5, 14.5. Anal. Calcd for $C_{27}H_{31}FN_6O_6$: C, 58.48; H, 5.63; N, 15.15; Found: C, 58.71; H, 5.80; N, 15.39.

1-ethyl-6-fluoro-*N*-(1-hydrazinyl-1-oxoethyl)-7-(4-phenylsulphonylpiperazin-1-yl)-4-oxo-1,4-dihydroquinoline-3-carboxamide 5c.

Yield= 0.16 g (66%); white powder, mp:172-174 °C. ^{1}H NMR 400 MHz (DMSO-d$_6$) δ (ppm): 10.16 (t, J= 6 Hz ,1H), 9.13 (s, 1H), 8.74 (s, 1H), 7.88-7.67 (m , 6H) 7.11 (d, $J_{H–F}$ = 6.6 Hz ,1H), 4.45 (q, J = 4.8, 9.5 Hz, 2H), 4.22 (s, 2H), 3.92 (d , J=7.2Hz, 2H), 3.37 (br. m, 4H), 3.09 (br. m, 4H), 1.35 (t, J = 5.1 Hz, 3H). ^{13}C NMR 100 MHz (DMSO-d$_6$): 173.9, 168.2, 164.6, 150.8, 147.5, 143.5, 136.9, 134.8, 133.8, 129.8, 127.8, 122.1, 111.4, 110.4, 106.7, 49.0, 48.4, 45.8, 40.8, 14.4. Anal. Calcd for $C_{24}H_{27}FN_6O_5S$: C, 54.33; H, 5.13; N, 15.84; S, 6.04; Found: C, 54.61; H, 5.29; N, 16.07; S, 6.09.

1-ethyl-6-fluoro-*N*-(1-hydrazinyl-1-oxoethyl)-7-(4-tolylsulphonylpiperazin-1-yl)-4-oxo-1,4-dihydroquinoline-3-carboxamide 5d.

Yield= 0.15 g (62%); white powder, mp:190-192 °C. ^{1}HNMR 400 MHz (DMSO-d$_6$) δ (ppm): 10.17 (t, J= 6 Hz ,1H), 9.13 (s,1H), 8.75 (s, 1H), 7.87 (d, J_{H-F} = 12.9 Hz, 1H), 7.68 (d, J = 8.4 Hz, 2H), 7.49 (d, J = 8.4 Hz, 2H), 7.12 (d, $J_{H–F}$ = 6.6 Hz ,1H), 4.48 (q, J = 4.8, 9.5 Hz, 2H), 4.23 (s, 2H), 3.93 (d , J=7.2Hz, 2H), 3.36 (br. m, 4H), 3.07 (br. m, 4H), 2.42 (s, 3H), ppm (t, J = 5.1 Hz, 3H). ^{13}CNMR 100 MHz (DMSO-d$_6$): 173.9, 168.5, 164.2, 153.9, 151.2, 147.5, 144.1, 136.5, 131.8, 130.1, 127.8, 122.1, 111.4, 110.4, 106.4, 48.9, 48.4, 45.7, 40.8, 21.1, 14.1. Anal. Calcd for $C_{25}H_{29}FN_6O_5S$: C, 55.14; H, 5.37; N, 15.43; S, 5.89; Found: C, 55.39; H, 5.44; N, 15.61; S, 5.97.

1-ethyl-6-fluoro-*N*-(1-hydrazinyl-1-oxoethyl)-7-(4-(4-chlorobenzyl)piperazin-1-yl)-4-oxo-1,4-dihydroquinoline-3-carboxamide 5e.

Yield= 0.17 g (70%); white powder, mp:210-212 °C. ^{1}H NMR 400 MHz (DMSO-d$_6$) δ (ppm): 10.2 (t, J= 6 Hz, 1H), 9.13 (s,1H), 8.75 (s, 1H), 7.88 (d, J_{H-F} = 12.9 Hz, 1H), 7.43-7.36 (m, 4H), 7.1 (d, $J_{H–F}$ = 6.6 Hz ,1H), 4.49 (q, J = 4.8, 9.5 Hz, 2H), 4.23 (s, 2H), 3.94 (d, J=7.2Hz, 2H), 3.56 (s, 2H), 3.27 ppm (br. m, 4H), 2.58 (br. m, 4H), 1.38 (t, J = 5.1 Hz, 3H). ^{13}C NMR 100 MHz (DMSO-d$_6$): 173.9, 168.2, 164.3, 151.2, 147.1, 144.8, 137.4, 136.5, 131.8, 130.8, 128.1, 121.8, 111.4, 110.4, 105.7, 60.9, 52.2, 49.7, 48.4, 40.8, 14.5. Anal. Calcd for $C_{25}H_{28}ClFN_6O_3$: C, 58.31; H, 5.48; Cl, 6.88; N, 16.32; Found: C, 58.60; H, 5.62; N, 16.49.

Synthesis of *N*-acylnorfloxacin amino acid derivatives (7a-i).

To a cooled stirred suspension of *N*-acyl norfloxacin derivative (0.5 g) in dichloromethane, triethylamine (2 equivalents) and ethyl chloroformate (1.5 equivalents) were added dropwise. The reaction mixture was stirred in an ice bath for one hour at 0-5 °C. After that, the solvent was evaporated under reduced pressure to get solid mixed anhydride and remove excess ethyl chloroformate. The obtained mixed anhydride was dissolved in a suitable amount of dichloromethane, then amino acid hydrochloride with 2 equivalents was added, and stirring was continued at room temperature for 24 hours. Progress of the reaction was observed by TLC monitoring (mobile phase 9.7:0.3 chloroform/methanol). After total consumption of the reactants, the organic layer was washed with saturated brine solution (2 x 25 ml) and distilled water (2 x 25 ml). Then, drying of the organic layer was done using sodium sulfate anhydrous, and the organic layer was evaporated under reduced pressure to obtain the solid residue. Purification of acid compounds was achieved through column chromatography using 9.7:0.3 dichloromethane/methanol as mobile phase and then by trituration with diethyl ether.

2-(1-Ethyl-6-fluoro-4-oxo-7-(4-(benzoylpiperazin-1-yl)-1,4-dihydroquinoline-3-carboxamido)acetic acid 7a.

Yield= 0.32 g (56%); beige powder, mp:243-245 °C. ^{1}H NMR 400 MHz (DMSO-d$_6$) δ (ppm): 10.81 (br. s,1H), 10.24 (t, *J*= 6 Hz, 1H), 8.78 (s,1H), 7.90 (d, $J_{H\text{-}F}$ = 12.9 Hz, 1H), 7.71-7.37 (m, 5H), 7.12 (d, $J_{H\text{–}F}$ = 6.6 Hz, 1H), 4.51 (q, *J* = 4.8, 9.5 Hz, 2H), 3.57 (s, 2H), 3.29 (m ,4H), 2.58 (m, 4H), 1.39 (t, *J* = 5.1 Hz, 3H). ^{13}C NMR 100 MHz (DMSO-d$_6$): 174.4, 171.6, 168.9, 163.6, 151.8, 148.0, 144.6,136.9, 136.1, 130.2, 129.0, 127.5, 122.4, 112.1, 111.5, 106.5, 50.3, 48.7, 44.7, 41.9, 14.9. Anal. Calcd for $C_{25}H_{25}FN_4O_5$: C, 62.49; H, 5.24; N, 11.66; Found: C, 62.31; H, 5.48; N, 11.90.

2-(1-Ethyl-6-fluoro-4-oxo-7-(4-(phenylsulphonylpiperazin-1-yl)-1,4-dihydroquinoline-3-carboxamido)acetic acid 7b.

Yield= 0.27 g (48%); yellow powder, mp:114-116 °C. ^{1}H NMR 400 MHz (DMSO-d$_6$) δ (ppm): 10.55 (br. s, 1H), 10.18 (t, *J*= 6 Hz, 1H), 8.77 (s, 1H), 7.88 (d, $J_{H\text{-}F}$ = 12.9 Hz, 1H), 7.82-7.68 (m, 5H), 7.14 (d, $J_{H\text{–}F}$ = 6.6 Hz, 1H), 4.49 (q, *J* = 4.8, 9.5 Hz, 2H), 4.05 (d, *J* = 5.6 Hz, 2H), 3.42-3.35 (m, 8H), 1.36 (t, *J* = 5.1 Hz, 3H). ^{13}C NMR 100 MHz (DMSO-d$_6$): 174.5, 171.8, 164.7, 151.8, 147.9, 144.2, 136.8, 135.1, 134.0, 130.0, 128.1, 122.4, 112.0, 110.7, 106.9, 49.4, 48.9, 46.2, 41.5,

14.8. Anal. Calcd for $C_{24}H_{25}FN_4O_6S$: C, 55.81; H, 4.88; N, 10.85; S, 6.21 Found: C, 55.94; H, 4.97; N, 11.09; S, 6.37.

2-(1-Ethyl-6-fluoro-4-oxo-7-(4-(tolylsulphonylpiperazin-1-yl)-1,4-dihydroquinoline-3-carboxamido)acetic acid 7c.

Yield= 0.25 g (45%); buff powder, mp:118-120 °C. IR (KBr): 3428 (NH str), 3400-2500 (OH str), 3055 (aromatic C-H str), 2980 (aliphatic C-H str), 1724 (carboxylic C=O str), 1656 (carbamidic C=O str) 1628 (quinolone C=O str), 1260 (C-O), 1164 (C-N) cm^{-1}. ^1H NMR 400 MHz (DMSO-d_6) δ (ppm): 10.16 (t, J= 6 Hz, 1H), 8.75 (s,1H), 7.86 (d, $J_{H\text{-}F}$ = 12.9 Hz, 1H), 7.67 (d, J = 8.3 Hz, 2H), 7.48 (d, J = 8.3 Hz, 2H), 7.13 (d, $J_{H\text{–}F}$ = 6.6 Hz ,1H), 4.48 (q, J = 4.8, 9.5 Hz, 2H), 4.03 (d , 2H), 3.45-3.30 (m, 8H), 2.41 (s, 3H), 1.21 (t, J = 5.1 Hz, 3H). ^{13}C NMR 100 MHz (DMSO-d_6): 174.5, 171.8, 164.7, 151.7, 147.9, 144.4, 144.3, 136.8, 132.0, 130.4, 128.2, 122.3, 111.9, 110.6, 106.9, 51.9, 49.6, 46.3, 41.4, 21.5, 14.7. Anal. Calcd for $C_{25}H_{27}FN_4O_6S$: C, 56.59; H, 5.13; N, 10.56; S, 6.04 Found: C, 56.76; H, 5.40; N, 10.85; S, 6.11.

2-(1-Ethyl-6-fluoro-4-oxo-7-(4-(4-nitrophenacyl)piperazin-1-yl)-1,4-dihydroquinoline-3-carboxamido)acetic acid 7d.

Yield= 0.38 g (63%); orange powder, mp: 170-172 °C. ^1H NMR 400 MHz (DMSO-d_6) δ (ppm): 10.19 (t, J= 6 Hz, 1H), 8.78 (s, 1H), 8.40-7.12 (m, 3H), 7.82 (d, J = 8 Hz, 2H), 7.10 (d, $J_{H\text{–}F}$ = 6.6 Hz, 1H), 4.60-4.12 (m, 4H), 4.05-3.58 (m, 6H), 3.06-2.98 (m, 4H), 1.39 (t, J = 5.1 Hz, 3H). ^{13}C NMR 100 MHz (DMSO-d_6): 190.3, 176.3, 166.2, 151.7, 148.4, 145.5, 137.3, 131.6, 130.7, 128.1, 119.2, 111.3, 107.1, 106.0, 60.9, 52.1, 49.5, 49.1, 14.5. Anal. Calcd for $C_{26}H_{26}FN_5O_7$: C, 57.88; H, 4.86; N, 12.98; Found: C, 58.09; H, 5.02; N, 13.17.

2-(1-Ethyl-6-fluoro-2-methyl-4-oxo-7-(4-(benzoylpiperazin-1-yl)-1,4-dihydroquinoline-3-carboxamido)acetic acid 7e.

Yield= 0.34 g (58%); yellow powder, mp: 139-141 °C. ^1H NMR 400 MHz (DMSO-d_6) δ (ppm): 10.31 (d, J= 6 Hz, 1H), 8.77 (s, 1H), 7.88 (d, $J_{H\text{-}F}$ = 12.9 Hz, 1H), 7.82-7.75 (m, 3H), 7.70 (m, 2H), 7.14 (d, $J_{H\text{–}F}$ = 6.6 Hz ,1H), 4.52-4.45 (m, 3H), 3.38-3.30 (m, 8H), 1.23-1.17 (m, 6H). ^{13}C NMR 100 MHz (DMSO-d_6): 174.4, 169.6, 164.1, 160.9, 151.8, 147.4, 144.8, 136.8, 129.7, 128.0, 122.1, 114.2, 112.0, 110.5, 106.6, 55.8, 50.3, 48.9, 15.0. Anal. Calcd for $C_{26}H_{27}FN_4O_5$: C, 63.15; H, 5.50; N, 11.33. Found: C, 63.41; H, 5.64; N, 11.57.

2-(1-Ethyl-6-fluoro-2-methyl-4-oxo-7-(4-(phenylsulphonylpiperazin-1-yl)-1,4-dihydroquinoline-3-carboxamido)acetic acid 7f.

Yield= 0.32 g (55%); yellow powder, mp:111-113 °C. IR (KBr): 3421 (NH str), 3300-2500 (OH str), 3026 (aromatic C-H str), 2977 (aliphatic C-H str), 1714 (carboxylic C=O str), 1655 (carbamidic C=O str) 1637 (quinolone C=O str), 1172 (C-O), 1036 (C-N) cm^{-1}. ^{1}H NMR 400 MHz (DMSO-d_6) δ (ppm): 10.30 (d, *J*= 6 Hz, 1H), 8.75 (s, 1H), 7.86 (d, J_{H-F} = 12.9 Hz, 1H), 7.82-7.67 (m, 5H), 7.14 (d, J_{H-F} = 6.6 Hz ,1H), 4.51-4.42 (m, 3H), 3.35 (m, 4H), 3.09 (m, 4H), 1.23-1.18 (m, 6H). ^{13}C NMR 100 MHz (DMSO-d_6): 174.1, 169.5, 164.2, 160.8, 151.7, 147.3, 144.9, 136.7, 129.6, 128.0, 122.0, 114.2, 112.0, 110.5, 106.5, 55.7, 50.2, 48.9, 14.9. Anal. Calcd for $C_{25}H_{27}FN_4O_6S$: C, 56.59; H, 5.13; N, 10.56; S, 6.04 Found: C, 56.43; H, 5.39; N, 10.84; S, 6.07.

2-(1-Ethyl-6-fluoro-2-mercaptomethyl-4-oxo-7-(4-(benzoylpiperazin-1-yl)-1,4-dihydroquinoline-3-carboxamido)acetic acid 7g.

Yield= 0.36 g (58%); yellow powder, mp:101-103 °C. IR (KBr): 3435 (NH str), 3300-2500 (OH str), 3041 (aromatic C-H str), 2977 (aliphatic C-H str), 1720 (carboxylic C=O str), 1650 (carbamidic C=O str) 1628 (quinolone C=O str), 1254 (C-O), 1171 (C-N) cm^{-1}. ^{1}HNMR 400 MHz (DMSO-d_6) δ (ppm): 10.49 (br. s, 1H), 10.40 (br. s, 1H), 8.77 (s,1H), 7.93 (d, J_{H-F} = 12.9 Hz, 1H), 7.48 (m, 5H), 7.16 (d, J_{H-F} = 6.6 Hz ,1H), 4.66 (br. s, 1H), 4.50 (q, *J* = 4.8, 9.5 Hz, 2H), 3.89-3.54 (m, 11H), 1.20 (t, *J* = 5.1 Hz, 3H). ^{13}C NMR 100 MHz (DMSO-d_6): 176.22, 166.03, 151.56, 148.66, 144.83, 134.64, 133.41, 129.38, 127.61, 119.88, 111.29, 107.11, 106.58, 66.72, 48.72, 45.57, 25.24, 14. Anal. Calcd for $C_{26}H_{27}FN_4O_5S$: C, 59.30; H, 5.17; N, 10.64; S, 6.09 Found: C, 59.47; H, 5.38; N, 10.89; S, 6.21.

2-(1-Ethyl-6-fluoro-2-mercaptomethyl-4-oxo-7-(4-(phenylsulphonylpiperazin-1-yl)-1,4-dihydroquinoline-3-carboxamido)acetic acid 7h.

Yield= 0.35 g (57%); yellow powder, mp:127-129 °C. ^{1}H NMR 400 MHz (DMSO-d_6) δ (ppm): 10.16 (br. s, 1H), 9.13 (s, 1H), 8.73 (s, 1H), 7.85 (d, J_{H-F} = 12.9 Hz, 1H), 7.81-7.67 (m, 5H), 7.11 (d, J_{H-F} = 6.6 Hz, 1H), 4.46 (q, *J* = 4.8, 9.5 Hz, 2H), 4.27-4.20 (br. m, 2H), 3.92 (d, *J* = 5.6 Hz, 2H), 3.36 (m, 4H), 3.09 (m, 4H), 1.34 (t, *J* = 5.1 Hz, 3H). ^{13}C NMR 100 MHz (DMSO-d_6): 174.0, 169.1, 164.5, 151.3, 147.5, 144.0, 143.8, 136.4, 131.7, 130.0, 127.7, 122.0, 111.6, 110.0, 106.5, 60.5, 48.7, 45.7, 40.9, 21.0, 14.4. Anal. Calcd for $C_{25}H_{27}FN_4O_6S_2$: C, 53.37; H, 4.84; N, 9.96; S, 11.40 Found: C, 53.63; H, 5.01; N, 10.23; S, 11.59.

2-(1-Ethyl-6-fluoro-2-carbamoylmethyl-4-oxo-7-(4-(benzoylpiperazin-1-yl)-1,4-dihydroquinoline-3-carboxamido)acetic acid 7i.

Yield= 0.29 g (46%); White powder, mp:110-112 °C. ^{1}H NMR 400 MHz (DMSO-d_6) δ (ppm): 10.58-10.34 (m, 4H), 8.76 (s, 1H), 7.91 (d, $J_{H\text{-}F}$ = 12.9 Hz, 1H), 7.51-7.45 (m, 5H) 7.16 (d, $J_{H–F}$ = 6.6 Hz ,1H), 4.50 (q, *J* = 4.8, 9.5 Hz, 2H), 4.33-4.02 (m, 5H), 3.87-3.79 (m, 4H), 1.39 (m, 2H), 1.20 (t, *J* = 5.1 Hz, 3H). ^{13}C NMR 100 MHz (DMSO-d_6): 174.0, 169.9, 169.9, 164.5, 164.5, 151.3, 147.5, 143.8, 136.4, 134.6, 133.5, 129.5, 127.7, 122.0, 111.5, 110.0, 106.5, 60.5, 49.0, 48.4, 45.7, 40.8, 14.1. Anal. Calcd for $C_{27}H_{28}FN_5O_6$: C, 60.33; H, 5.25; N, 13.03; Found: C, 60.47; H, 5.43; N, 13.29.

Synthesis of *N*-acylnorfloxacin amino acid hydroxamic derivatives (8a-d).

A mixture of 0.2 g *N*-acyl norfloxacin amino acid derivative **7a-b**, **7e**, or **7g** and triethylamine with two equivalents in dichloromethane (15 mL) was stirred in an ice bath for ten minutes. Following this, 1.5 equivalents of ethyl chloroformate were added to the reaction mixture dropwise, and the reaction mixture was further stirred in an ice bath for one hour at 0-5 °C. Then, two equivalents of hydroxylamine hydrochloride were added, and stirring was continued at room temperature for 24 hours. Progress of the reaction was observed by TLC (mobile phase 9.7:0.3 chloroform/methanol). After total consumption of the reactants, the organic layer was washed with saturated brine solution (2 x 25 mL) and distilled water (2 x 25 mL), and then dried using sodium sulfate anhydrous. The dried organic layer was evaporated under vacuum, and the obtained hydroxamic compounds were purified by column chromatography using 9.5:0.5 dichloromethane/methanol as mobile phase.

7-(4-benzoylpiperazin-1-yl)-1-ethyl-6-fluoro-*N*-(2-(hydroxyamino)-2-oxoethyl)-4-oxo-1,4-dihydroquinoline-3-carboxamide 8a.

Yield= 0.08 g (39%); brown powder, mp:103-105 °C. IR (KBr): 3419 (NH str), 3233 (OH str), 3056 (aromatic C-H str), 2916 (aliphatic C-H str), 1672 (Hydroxamic C=O str), 1650 (carbamidic C=O str) 1628 (quinolone C=O str), 1254 (C-O), 1010 (C-N) cm^{-1}. ^{1}H NMR 400 MHz (DMSO-d_6) δ (ppm): 10.19 (t, *J*= 6 Hz, 1H), 8.75 (br. s, 2H), 7.89 (d, *J(H-F)* = 12.9 Hz, 1H), 7.50-7.45 (m, 5H), 7.13 (d, *J(H–F)* = 6.6 Hz, 1H), 4.48 (q, *J* = 4.8, 9.5 Hz, 2H), 4.07-3.71 (m, 6H), 3.63-3.52 (m, 4H), 1.38 (t, *J* = 5.1 Hz, 3H). ^{13}C NMR 100 MHz (DMSO-d_6): 173.9, 169.2, 164.6, 162.9, 151.5, 147.2, 144.5, 136.9, 135.5, 130.2, 128.5, 127.1, 121.8, 111.8, 110.4, 106.4, 61.0, 49.9, 48.2, 41.5, 14.8. Anal. Calcd for $C_{25}H_{26}FN_5O_5$: C, 60.60; H, 5.29; N, 14.13; Found: C, 60.89; H, 5.45; N, 14.31. LRMS for $[C_{24}H_{23}FN_4O_4]^+$ $[M]^+$ calculated: 495.19 found: 495.13.

7-(4-phenylsulphonylpiperazin-1-yl)-1-ethyl-6-fluoro-*N*-(2-(hydroxyamino)-2-oxoethyl)-4-oxo-1,4-dihydroquinoline-3-carboxamide 8b.

Yield= 0.06 g (29%); yellow powder, mp:139-141 °C. ^{1}H NMR 400 MHz (DMSO-d$_6$) δ (ppm): 10.19 (t, *J*= 6 Hz, 1H), 9.13 (br. S, 1H), 8.76 (s, 1H), 7.91 (d, J_{H-F} = 12.9 Hz, 1H), 7.50-7.44 (m, 6H), 7.14 (d, $J_{H–F}$ = 6.6 Hz ,1H), 4.49 (q, *J* = 4.8, 9.5 Hz, 2H), 3.93 (d, *J* = 5.6 Hz, 2H), 3.84-3.55 (m, 4H), 3.32-3.25 (m, 4H), 1.38 (t, *J* = 5.1 Hz, 3H). ^{13}C NMR 100 MHz (DMSO-d$_6$): 174.1, 170.0, 164.6, 151.1, 147.9, 147.4, 137.1, 136.5, 131.6, 130.7, 128.2, 122.1, 111.4, 110.0, 106.1, 60.5, 52.2, 48.1, 40.9, 14.1. Anal. Calcd for $C_{24}H_{26}FN_5O_6S$: C, 54.23; H, 4.93; N, 13.18; S, 6.03 Found: C, 54.51; H, 5.12; N, 13.40: S, 6.11.

7-(4-benzoylpiperazin-1-yl)-1-ethyl-6-fluoro-*N*-(2-(hydroxyamino)-2-oxoethyl)-2-methyl-4-oxo-1,4-dihydroquinoline-3-carboxamide 8c.

Yield= 0.14 g (68%); yellow powder, mp:103-105 °C. ^{1}H NMR 400 MHz (DMSO-d$_6$) δ (ppm): 10.25 (d, *J*= 6 Hz, 1H), 8.92-8.53 (m, 2H), 8.02-7.77 (m, 2H), 7.51-7.45 (m, 5H), 7.12 (d, $J_{H–F}$ = 6.6 Hz ,1H), 4.45 (q, *J* = 4.8, 9.5 Hz, 2H), 4.22 (q, *J* = 7.2 Hz, 1H), 4.05-3.72 (m, 4H), 3.67-3.45 (m, 4H), 1.41-1.22 (m, 6H). ^{13}C NMR 100 MHz (DMSO-d$_6$): 174.6, 172.0, 169.6, 166.1, 151.7, 147.9, 144.7, 136.9, 136.1, 130.2, 129.0, 127.5, 122.3, 112.1, 109.9, 106.6, 60.2, 50.3, 48.7, 47.5, 20.1, 14.8. Anal. Calcd for $C_{26}H_{28}FN_5O_5$: C, 61.29; H, 5.54; N, 13.74; Found: C, 61.08; H, 5.67; N, 13.98.

7-(4-benzoylpiperazin-1-yl)-1-ethyl-6-fluoro-*N*-(2-(hydroxyamino)-2-oxoethyl)-2-2-mercaptomethyl-methyl-4-oxo-1,4-dihydroquinoline-3-carboxamide 8d.

Yield= 0.11 g (54%); yellow powder, mp:247-249 °C. ^{1}H NMR 400 MHz (DMSO-d$_6$) δ (ppm): 10.45 (br. s, 1H), 8.76 (s, 1H), 8.27 (br. s, 1H), 7.86 (m, 2H), 7.48 (m, 5H), 7.12 (d, $J_{H–F}$ = 6.6 Hz ,1H), 4.44 (m, 3H), 3.86-3.77 (m, 5H), 3.62-3.51 (m, 6H), 1.38 (t, *J* = 5.1 Hz, 3H). ^{13}C NMR 100 MHz (DMSO-d$_6$): 172.9, 162.0, 151.1, 146.5, 143.6, 135.9, 131.4, 130.1, 127.3, 121.5, 111.8, 109.9, 106.3, 54.8, 49.1, 48.1, 45.6, 20.8, 14.3. Anal Calcd for $C_{26}H_{28}FN_5O_5S$: C, 57.66; H, 5.21; N, 12.93; S, 5.92 Found: C, 57.89; H, 5.42; N, 13.21; S, 5.88.

Text S8: Molecular modeling

Optimization of target compounds

The target ligands for modelling compounds were built using the builder interface of the MOE software package 2020.01 and subjected to conformational search. Conformers were subjected to energy minimization until a RMSD gradient of 0.01 Kcal/mol and RMS distance of 0.1 Å with MMFF94X force-field and the partial charges were automatically calculated. The obtained database was then saved as MDB file to be used in the physicochemical properties and docking calculations.

Calculation of physicochemical properties

Calculation of the physicochemical properties of the compounds, including AM1_dipole (AM1), water accessible surface area (ASA), Lipinski acceptor count (lip_acc), Lipinski donor count (lip_don), Lipinski druglike test (lip_druglike), log octanol/water partition coefficient (logP(o/w)), log solubility in water (logS), topological polar surface area (TPSA), van der waals surface area (VSA), molecular weight (weight), and number of rotatable bonds (nrotb), were calculated on MOE 2020.1 using the calculate descriptors command.

***in silico* prediction of ADME/Tox**

Predictions were performed using the pkCSM pharmacokinetics and SwissADME webtools (http://biosig.unimelb.edu.au/pkcsm/prediction and http://www.swissadme.ch) [4, 15]. Molecular structures of the compounds were built in ChemDraw Ultra 8.0, then copied as SMILES (simplified molecular-input line-entry specification) nomenclature, and pasted into the respective web tools.

Molecular docking

The crystal structure of moxifloxacin with *S. aureus* DNA gyrase and DNA (PDB code 5cdq) [26], the crystal structure of moxifloxacin, DNA, and *A. baumannii* topoisomerase IV (PDB code 2xkk) [27], structure of co-crystal of *P. aeruginosa* LpxC-50432 complex (PDB code: 6mod) [18], and the crystal structure of N-acetyl-D-glucosamine-6-phosphate deacetylase D267A mutant from *M. smegmatis* in complex with N-acetyl-D-glucosamine-6-phosphate (PDB code 6fv4) [20] were obtained from the protein data bank (PDB). Docking was run on the binding site of the co-crystallized ligand. Since the crystal structure contains a ligand molecule, the program

automatically identifies the binding site, and the tested ligands were docked onto it. Docking of the conformations database of the target ligands was done using MOE-DOCK software wizard. The following parameters were adjusted: 1. receptor and solvent as receptor, 2. co-crystalized ligand atoms as active site, database containing test ligands as ligand, London dG as initial scoring function, GBVI/WSA dG as final scoring function, and MMFF94x force field was used for calculating the energy parameters of the ligand – cleavage complex model. To compare between the conformers London dG was used as scoring function. The 2D and 3D ligand interactions for each compound were saved as picture files and color coding was chosen according to **Figure S99**.

Ligand-based pharmacophore modeling

The ligand-based pharmacophore query was determined from a collection of 40 active ligands in MOE 2020.01 using the following steps: 1. flexible alignment, 2. pharmacophore consensus, 3. feature selection and pharmacophore saving, 4. model validation, 5. pharmacophore search. The training set (40 compounds, **Table S11**), the validation test set (17 compounds, **Table S12**) and the target compound set (24 compounds) were built using the MOE builder interface and subjected to conformational search. Conformers were subjected to energy minimization as in the mentioned docking experiments. The obtained databases were then saved as MDB file to be used in the flexible alignment, validation, and pharmacophore search. Flexible alignment was adjusted to iteration limit = 200, failure limit = 30, and energy cutoff = 20.

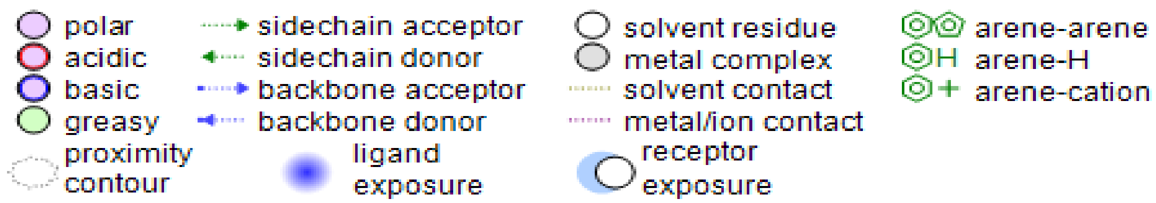

Figure S98: Color scheme for the 2D representations of the interactions between the docked ligands and the active site of the enzyme.

Table S12: Training set compounds.

Code	$IC_{50\%}$ (nM)	Code	$IC_{50\%}$ (nM)
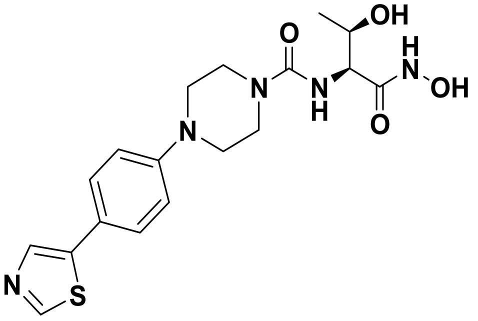 **1**	12	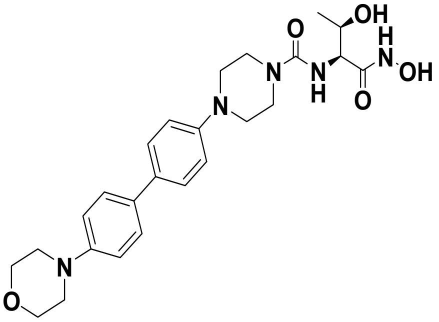 **2**	16.3
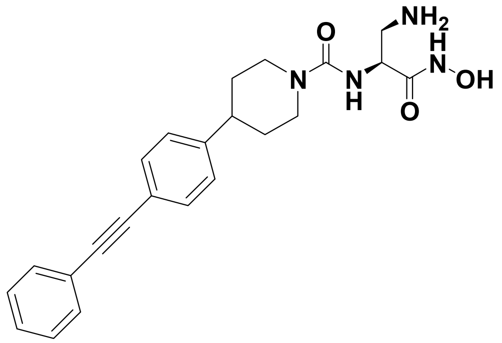 **3**	163.1	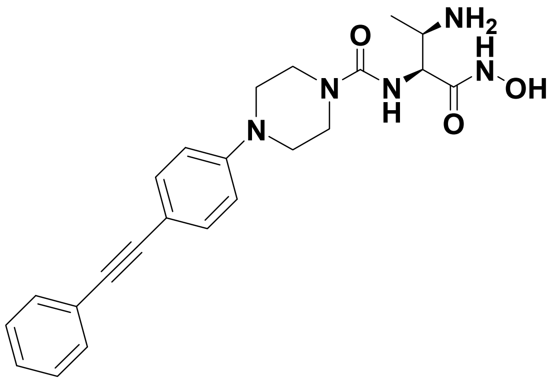 **4**	0.6
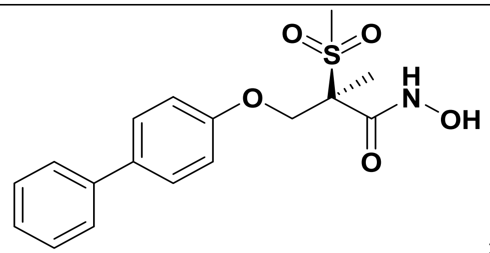 **5**	31.4	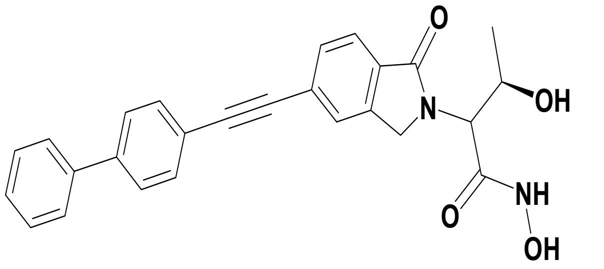 **6**	13
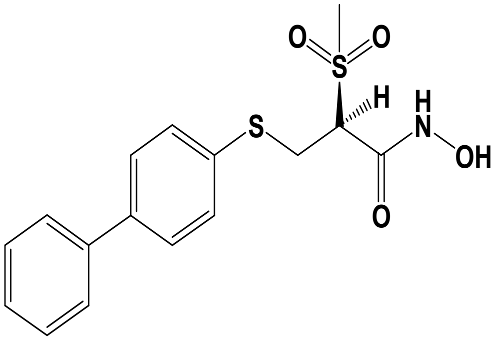 **7**	9.04	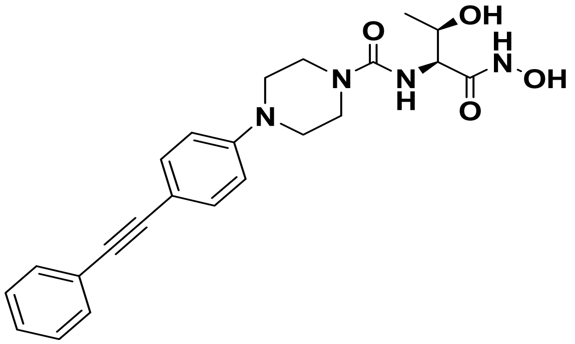 **8**	2.4

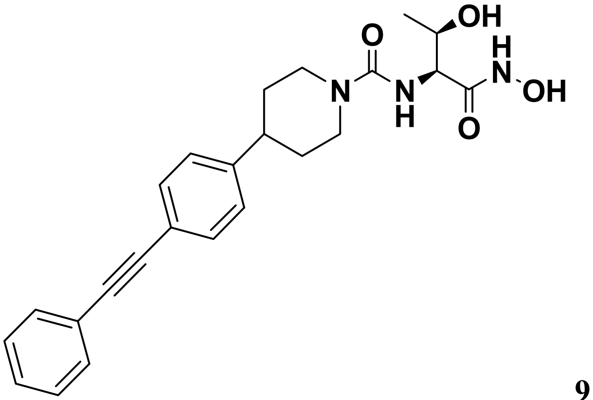 **9**	13.8	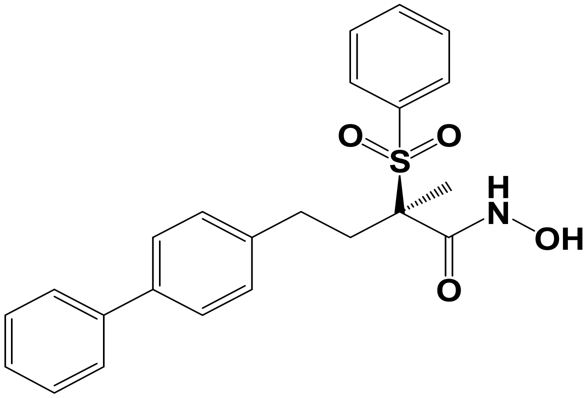 **10**	67.9
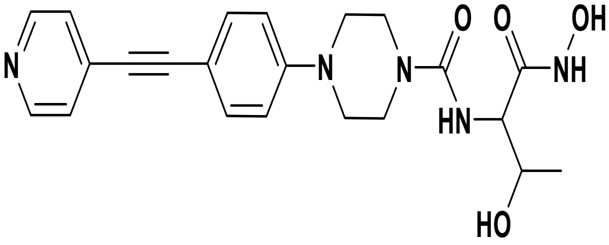 **11**	4	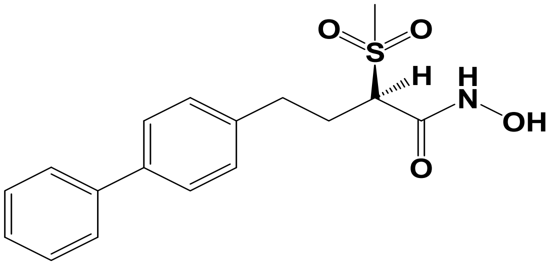 **12**	13.8
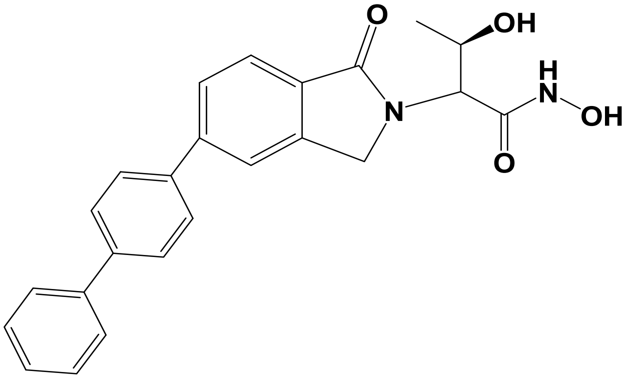 **13**	50	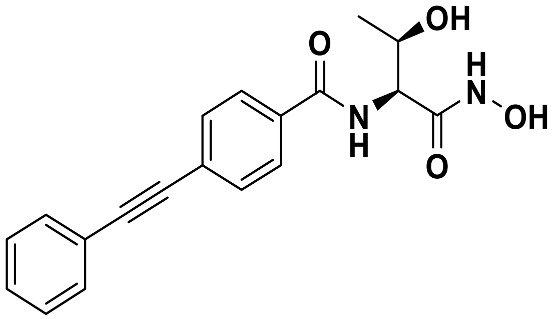 **CHIR-12** **14**	2
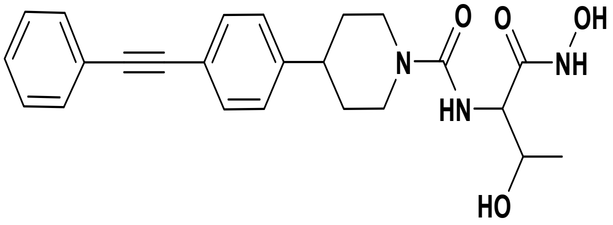 **15**	2.1	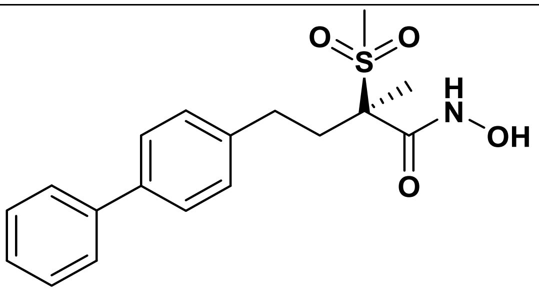 **16**	1.37

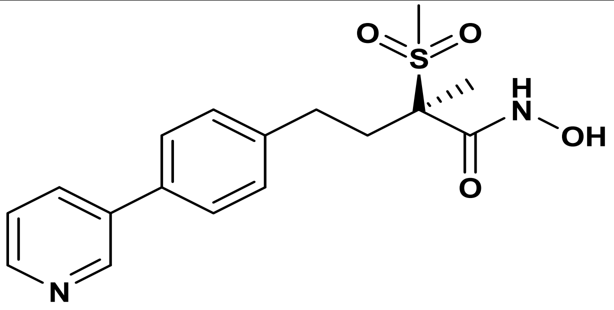<br>**17**	29.7	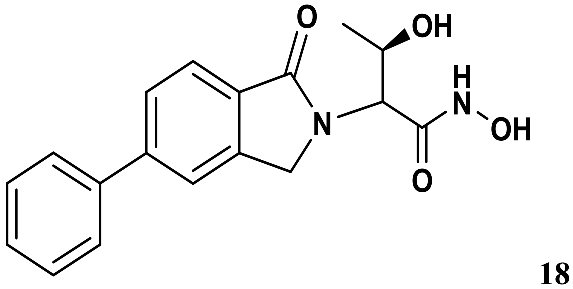<br>**18**	41
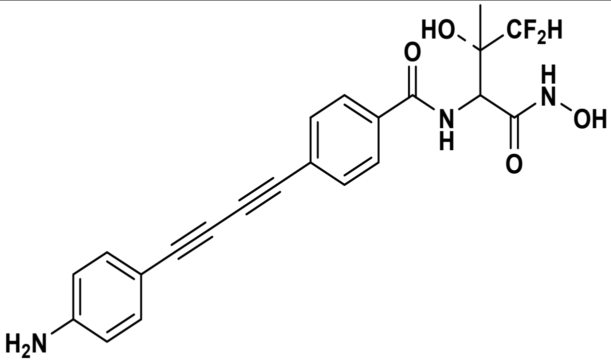<br>**LPC-058**<br>**19**	0.0035	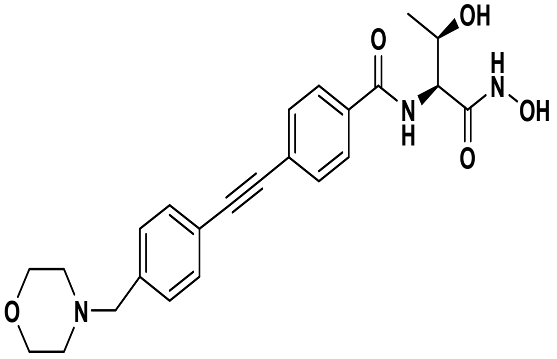<br>**CHIR-90**<br>**20**	2
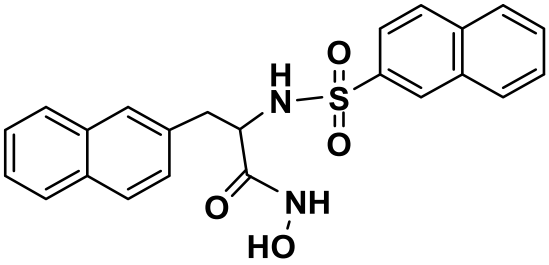<br>**BB-78485**<br>**21**	20	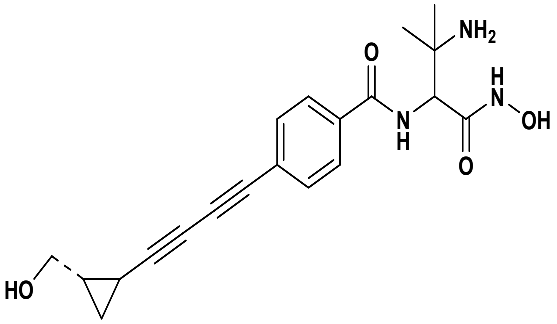<br>**ACHN-975**<br>**22**	0.02
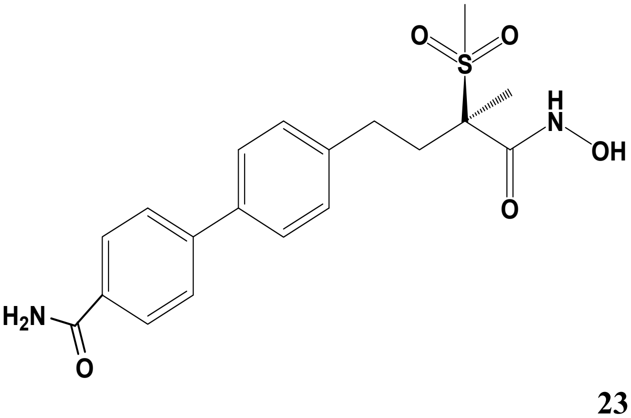<br>**23**	247	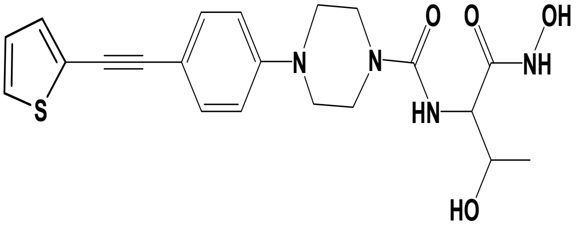<br>**24**	21

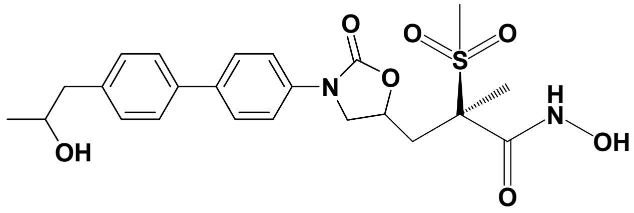 25	0.12	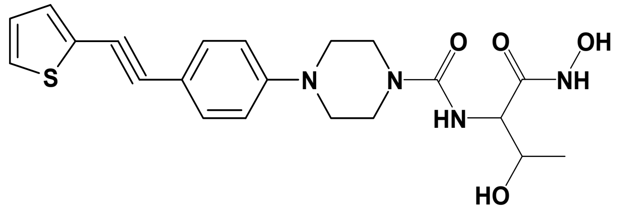 26	136
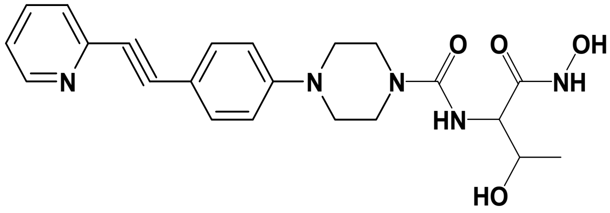 27	10	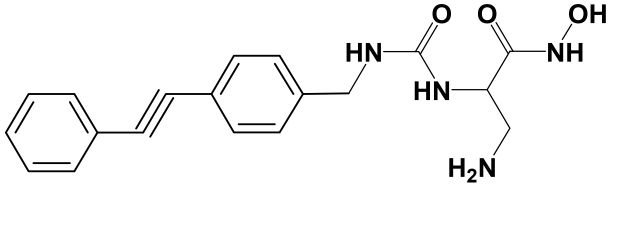 28	163.1
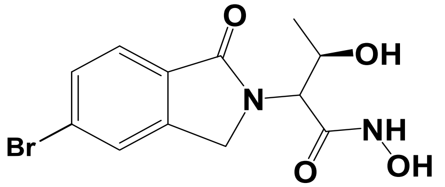 29	61	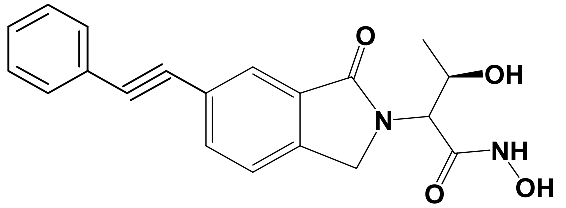 30	34
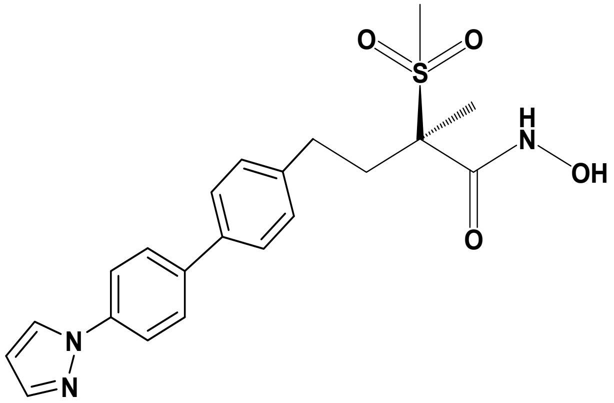 31	22.1	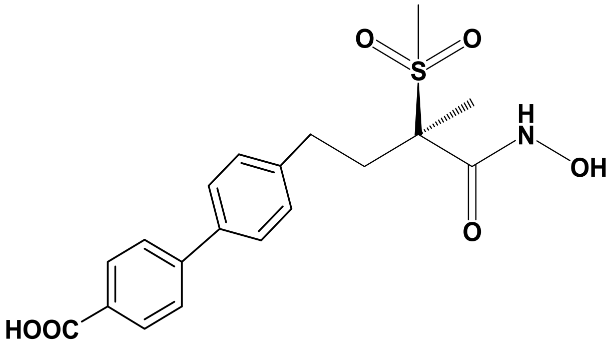 32	73.6
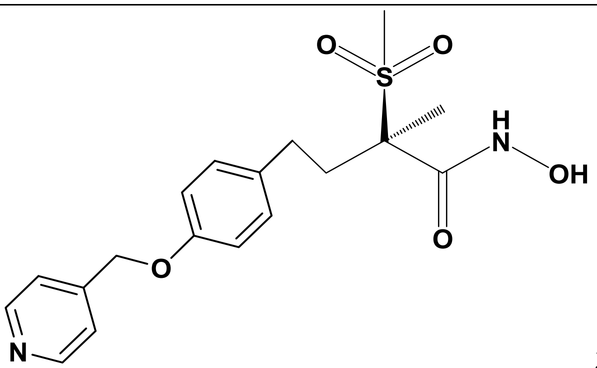 33	21.6	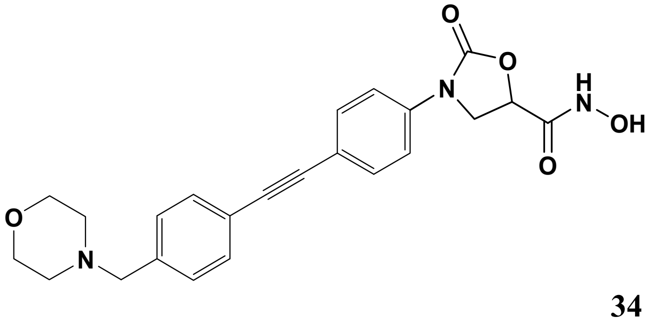 34	130

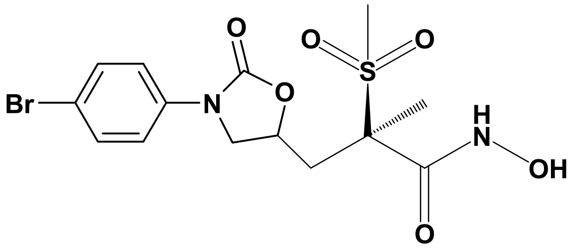 **35**	0.21	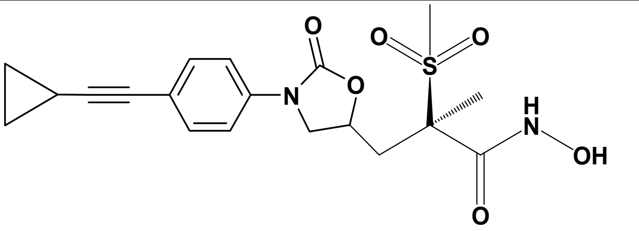 **36**	0.65
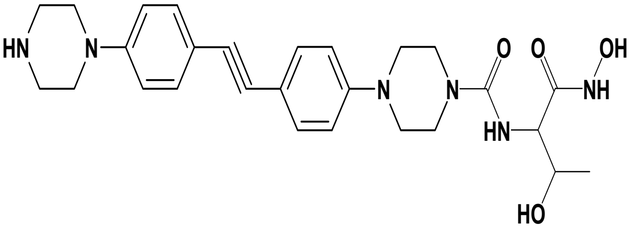 **37**	23.3	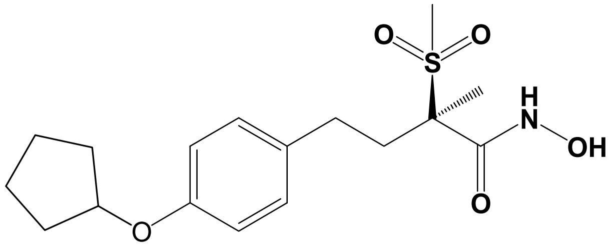 **38**	10.1
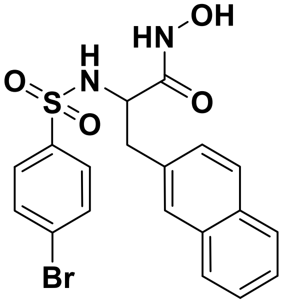 **BB-78484** **39**	400	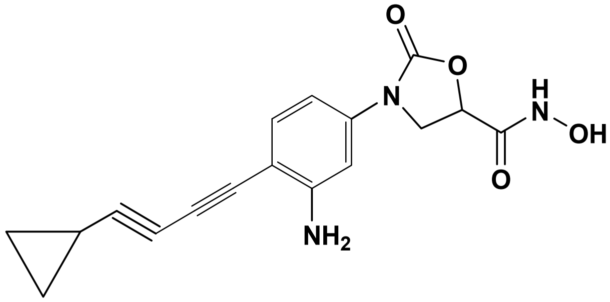 **40**	27

Table S13: Validation test set compounds.

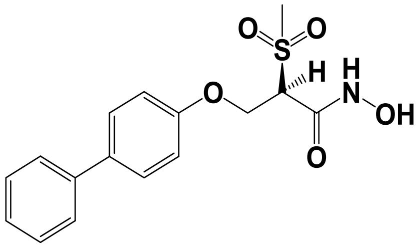 **41**	31.4	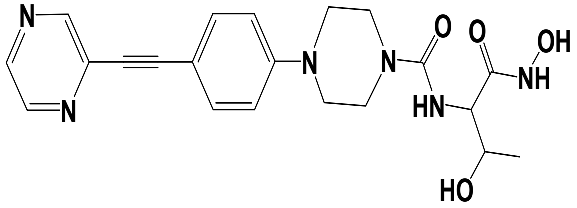 **42**	136
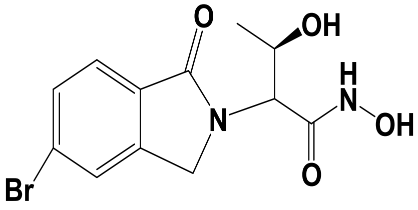 **43**	61	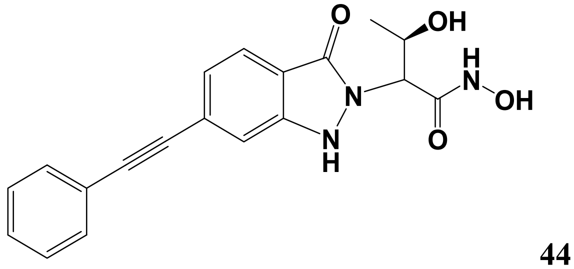 **44**	200
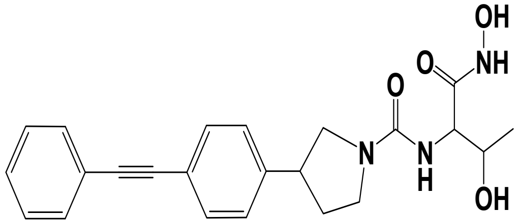 **45**	146.4	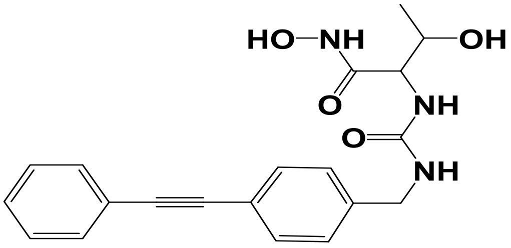 **46**	201.4
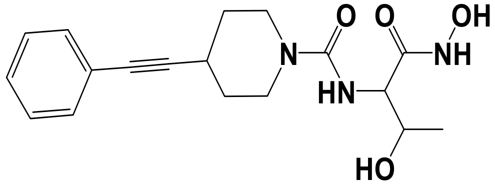 **47**	207	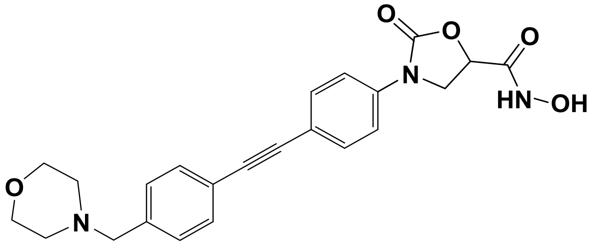 **48**	260
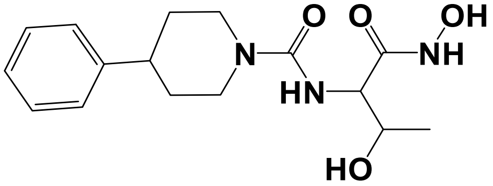 **49**	3944.5	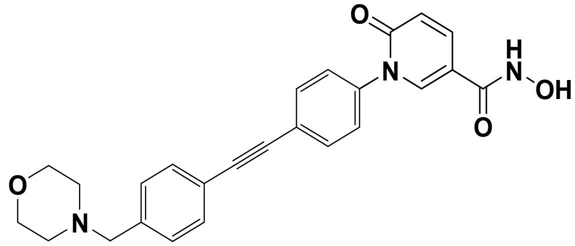 **50**	1000

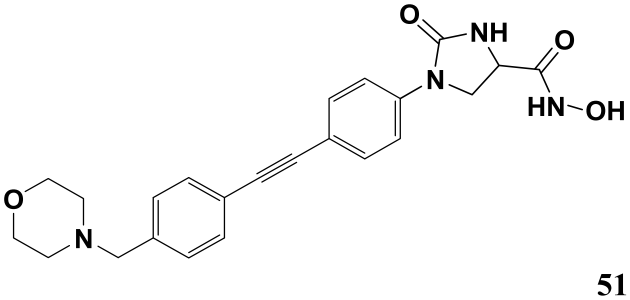 **51**	700	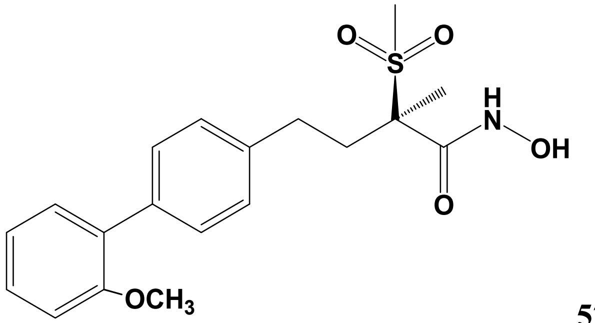 **52**	7.04
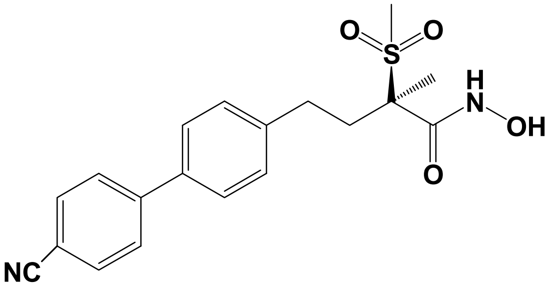 **53**	0.64	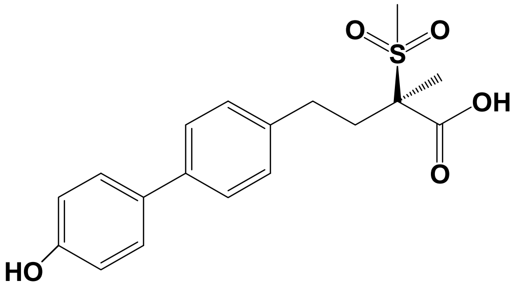 **54**	92.5
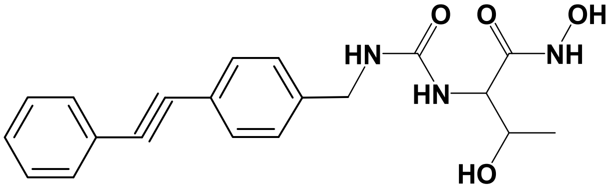 **55**	204.1	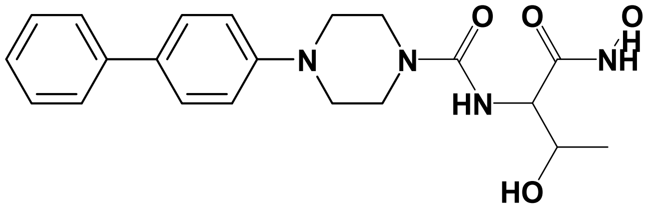 **56**	5.6
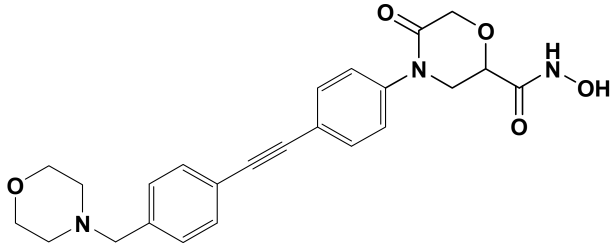 **57**	510		

Text S9: Biology

Cytotoxicity

Cell viability was evaluated by the reduction of 3-(4,5-dimethyl-2-thiazolyl)-2,5-diphenyl-2Htetrazolium bromide (MTT) to its insoluble formazan, reporting on mitochondrial metabolic function. SH-SY5Y (human neuroblastoma cell line) and diploid human normal (WI-38) cells were seeded in a 96-well plate at 2 × 104 cells per well. Cells were treated for 24 h with staurosporine, norfloxacin, or compounds **4f** and **5b** (2.5-80 μM)., followed by replacement of the culture medium d with MTT solution (0.5 mg mL^{-1}) in Hank's Balanced Salt Solution (HBSS) for 2 h at 37 °C in a 5% CO_2 atmosphere. After washing with HBSS, formazan crystals were dissolved in isopropanol. The amount of formazan was measured using a multilabel plate reader (VICTOR™ X3, PerkinElmer, Waltham, MA, USA) and an Anthos Zenyth 200rt microplate reader (Biochrom, UK) (λ = 570 nm, reference filter 690 nm). The cytotoxicity of the test compound was obtained using the following formula: [(A − B)/A × 100], where A represents the absorbance of untreated cells and B the absorbance of cells treated with different concentrations of the test compounds. Cytotoxic concentration for 50% of cells (IC_{50}) was determined by linear regression.

in vitro enzyme inhibition

Inhibition of *E. coli* DNA gyrase and topoisomerase IV was assessed with corresponding assay kits (Inspiralis®) according to the manufacturer's instructions [28]. Compounds most active against *E. coli* were selected for testing gyrase inhibition, while compounds most active against *S. aureus* were selected for testing topoisomerase IV inhibition. Compounds were diluted in DMSO and IC_{50} values were determined using norfloxacin as a reference drug.

UV-Vis spectroscopy

Compounds were prepared as 30 μM solutions in methanol. Metals were prepared in varying concentrations from 15 to 35 μM in HEPES buffer (20 mM, pH 7.4) [29]. The absorption spectra of the compounds alone or in the presence of $MgCl_2$, $ZnCl_2$, and $CdCl_2$, respectively, were recorded at room temperature in a 1 cm quartz cell using UV-Visible Spectrophotometer (PG Instruments Limited, T80, United Kingdom). Additionally, the ratio of ligand/metal ion in the complex was determined by a molar ratio method [30], wherein fixed concentrations of the

compounds (30 μM) were mixed with ascending concentrations of each metal (15–35 μM), and UV–vis absorption spectra were recorded.

Bacterial strains and growth conditions

Bacterial strain used in this study are listed in **Table S13**. *E. coli, S. aureus*, and *B. subtilis* were grown in Mueller Hinton broth, *P. aeruginosa* in cation-adjusted Muller Hinton II, and *M. tuberculosis* in Middlebrook 7H9 medium. *M. tuberculosis* was grown at 30 °C, all other strains at 37 °C. Expression of NeonGreen-GlpT in *E. coli* BCB472 was induced by addition of 20 μM isopropyl β- d-1-thiogalactopyranoside (IPTG) for 60 min. *B. subtilis* GFP-expressing strains were constantly grown in the presence of inducer (0.3% xylose for MW10, 0.05% xylose for TNVS284, and 0.1% xylose for TNVS284, EKB46, and TNVS45).

Minimal inhibitory concentrations

Minimal inhibitory concentrations against *E. coli, P. aeruginosa, S. aureus,* and *B. subtilis* were performed in a microdilution protocol according to CLSI guidelines as described previously [31, 32] . Antimicrobial activity against *M. tuberculosis* was tested using a modified protocol according to [33].

Microscopy

All microscopy performed on a Nikon Eclipse Ti2 equipped with a CFI Plan Apochromat DM Lambda 100X Oil objective (N.A. 1.45, W.D. 0.13mm), a Photometrics, PRIME BSI camera, a Lumencor Sola SE II FISH 365 light source, and an Okolab temperature incubation chamber. Images were obtained using the NIS elements AR software version 5.21.03 and analyzed with ImageJ and MicrobeJ [34, 35].

Bacterial cytological profiling (BCP)

BCP was performed using *E. coli* W3110 and *B. subtilis* DSM402. Strains were grown until an OD_{600} of 0.3 prior to treatment with 1xMIC of the respective compounds for 60 min. If no clear phenotype was observed, compounds were additionally tested at 2x and 4xMIC to confirm negative results. After antibiotic treatment, samples were stained with 1 μM FM6-64 and 1 μM DAPI for 5 min. Samples were then spotted on agarose-covered microscopy slides as described previously [36]. *E. coli* BCB472 was grown until and OD_{600} of 0.3 prior to addition of 20 μM IPTG together with the respective compounds for 60 min prior to sample spotting and microscopy.

Membrane potential measurements

Membrane potential was assessed with DiSC(3)5 as described previously [37]. In short, *B. subtilis* DSM402 was grown in presence of 50 µg/mL bovine albumin serum (BSA) and after reaching an OD_{600} of 0.3, 1 µM DiSC(3)5 was added to the cells. Antibiotics were added after the fluorescence baseline was stable and fluorescence was monitored over 30 min in a BMG Clariostar Plus plate reader at an excitation wavelength of 610-30 nm and an emission wavelength of 675-50 nm.

Checkerboard assays

Outer membrane integrity was tested by assessing synergy with mupirocin, which is able to inhibit *E. coli* isoleucine tRNA synthase but cannot pass its outer membrane [37]. To this end, checkerboard assays were performed [38]. The fractional inhibitory concentration index was calculated according to the formula FICI = $(MIC_A^{combi}/MIC_A^{alone}) + (MIC_B^{combi}/MIC_B^{alone})$. FICI values of ≤0.5 were defined as synergy, >0.5 to ≤4.0 as additive (no interaction), and >4.0 was defined as antagonism.

LpxC overexpression assay

To assess LpxC as possible target, MICs were determined against a strain overexpressing the *lpxC* gene from the arabinose-inducible P_{BAD} promoter. To this end, *E. coli* BL21 DE03 carrying either pBO110 (P_{BAD}-*lpxC*) or pBAD24 (empty vector control) [39] were grown in presence of 100 µg/mL ampicillin to ensure plasmid maintenance. MICs were determined in Muller Hinton broth containing 0, 0.005, 0.01, or 0.05% arabinose. If LpxC is a target of the compound, the MIC should increase with rising arabinose concentrations due to the presence of more target molecules. As positive control, the specific LpxC inhibitor ACHN-975 was used [40]. As additional control for the specificity of the assay, the outer membrane-permeabilizing lipopeptide polymyxin B and the reactive species-forming pro-drug nitrofurantoin were included.

Acetic acid/methanol fixation

Peptidoglycan integrity was tested with an established acetic acid/methanol fixation protocol using *B. subtilis* DSM402 [41, 42]. In short, *B. subtilis* was grown until an OD_{600} of 0.3, treated with antibiotics for 10 (vancomycin, 1 µg/mL) or 60 min (all other compounds, 1xMIC), and subsequently fixed in a 1:3 mixture of acetic acid and methanol. Samples were observed by phase contrast microscopy as described above.

Protein localization

GFP-expressing *B. subtilis* strains MW10 (GFP-MreB), TNVS175 (MurG-msfGFP), TNVS284 (MraY-msfGFP), EKB46 (msfGFP-PbpB), and TNVS45 (mGFP-PonA) were grown until early log phase in Muller Hinton broth supplemented with appropriate concentrations of xylose (see above). Cells were treated with 1x MIC of the respective compounds for 30 min (vancomycin) or 1 h (all other compounds) prior to microscopy. TNVS45, which showed a spotty localization with some compounds was additionally stained with FM4-64 to visualize co-localization with membrane patches. Samples were spotted on agarose-covered microscopy slides and observed by fluorescence microscopy. In the case of MreB, two separate images if the same field of view were recorded in a 30 sec interval and overlaid in ImageJ to visualize MreB mobility. A perfect overlap (yellow) indicates stalled MreB movement while distinct red and green spots are indicative of MreB mobility.

Table S14: Strains used in this study. i. a. = if applicable, *mgfp* = monomeric green-fluorescent protein, *msfgfp* = monomeric superfolder green-fluorescent protein, #Ciprofloxacin=R, *Nitrofurantoin=R, Cefadroxil=R, Penicillin G/V=R, Isoxa-pc=R, Cefuroxim=R, Cefotaxim=R, Ceftazidim=R, Imipenem=R, Tobramycin=R, Trim-Sulfa=R, Norfloxacin=R, Ciprofloxacin=R, Clindamycin=R, Fusidic acid=S, Vancomycin=S, Netilmic=R

Species and strain	Relevant genotype	Reference
E. coli W3110	*F-, IN(rrnD-rrnE)1*	https://doi.org/10.13145/bacdive4747.20201210.5
*E. coli**		clinical resistant isolate
E. coli BCB472	*psav057-NeonGreen-2GS-GlpT*	[43]
E. coli BL21DE03 pBAD24	P_{BAD}, *araC, rrnBT,* Amp^r	[39]
E. coli BL21DE03 pBO110	P_{BAD}-*lpxC*	[39]
K. pneumoniae ATCC10031		doi:10.13145/bacdive4968.20220920.7
P. aeruginosa PAO1		doi.org/10.13145/bacdive12801.20201210.5
S. aureus CCUG1800T		doi.org/10.13145/bacdive14487.20201210.5
S. aureus ATCC43300#		doi:10.13145/bacdive14464.20220920.7
M. tuberculosis MC26020	Δ*lysA* Δ*panCD*	[44]
B. subtilis DSM402	*trpC2*	doi.org/10.13145/bacdive1156.20201210.5
B. subtilis EKB46	*trpC2 amyE::spc Pxyl-msfgfp*	[45]
B. subtilis MW10	*trpC2 amyE::spc Pxyl-gfp-mreB*	[45]
B. subtilis TNVS45	*trpC2 amyE::spc Pxyl-mgfp-ponA*	[45]
B. subtilis TNVS175	*trpC2 amyE::spc Pxyl-murG-msfgfp*	[45]
B. subtilis TNVS284	*trpC2 amyE::spc Pxyl-mraY-msfgfp*	[45]

#Ciprofloxacin = R

* Nitrofurantoin=R, Cefadroxil=R, Penicillin G/V=R, Isoxa-pc=R, Cefuroxim=R, Cefotaxim=R, Ceftazidim=R, Imipenem=R, Tobramycin=R, Trim-Sulfa=R, Ciprofloxacin=R, Clindamycin=R, Fusidic acid=S, Vancomycin=S, Netilmic=R

5. References

Text S10: References

[1] I.M. Vlad, D.C. Nuta, C. Chirita, M.T. Caproiu, C. Draghici, F. Dumitrascu, C. Bleotu, S. Avram, A.M. Udrea, A.V. Missir, L.G. Marutescu, C. Limban, In Silico and In Vitro Experimental Studies of New Dibenz[b,e]oxepin-11(6H)one O-(arylcarbamoyl)-oximes Designed as Potential Antimicrobial Agents. *Molecules* (Basel, Switzerland) 25(2) (2020).
[2] P. Ertl, B. Rohde, P. Selzer, Fast calculation of molecular polar surface area as a sum of fragment-based contributions and its application to the prediction of drug transport properties. *J. Med. Chem.* 43(20) (2000) 3714-3717.
[3] D.F. Veber, S.R. Johnson, H.-Y. Cheng, B.R. Smith, K.W. Ward, K.D. Kopple, Molecular Properties That Influence the Oral Bioavailability of Drug Candidates. *J. Med. Chem.* 45(12) (2002) 2615-2623.
[4] http://biosig.unimelb.edu.au/pkcsm/prediction, (Accessed at January 8,. 2022.).
[5] S. O'Hagan, D.B. Kell, The apparent permeabilities of Caco-2 cells to marketed drugs: magnitude, and independence from both biophysical properties and endogenite similarities. *PeerJ.* 3 (2015) e1405.
[6] https://readycell.com/caco-2-permeability-protocol/, (Accessed at January 5,. 2022.).
[7] J. Yates, P. Arundel, On the Volume of Distribution at Steady State and Its Relationship With Two-Compartmental Models. *J. Pharm. Sci.* 97 (2008) 111-122.
[8] A. Mansoor, N. Mahabadi, Volume of Distribution, StatPearls, StatPearls Publishing Copyright © 2021, StatPearls Publishing LLC., Treasure Island (FL), 2021.

[9] T.S. Carpenter, D.A. Kirshner, E.Y. Lau, S.E. Wong, J.P. Nilmeier, F.C. Lightstone, A method to predict blood-brain barrier permeability of drug-like compounds using molecular dynamics simulations. *Biophys. J.* 107(3) (2014) 630-641.
[10] S.K. Bardal, J.E. Waechter, D.S. Martin, Chapter 2 - Pharmacokinetics, in: S.K. Bardal, J.E. Waechter, D.S. Martin (Eds.). *Appl. Pharmacol.* W.B. Saunders, Philadelphia, 2011, pp. 17-34.
[11] J.M. Collins, Chapter 46 - Pharmacokinetics, Pharmacodynamics, and Pharmacogenetics1, in: J. Mendelsohn, P.M. Howley, M.A. Israel, J.W. Gray, C.B. Thompson (Eds.). *The Molecular Basis of Cancer* (Third Edition), W.B. Saunders, Philadelphia, 2008, pp. 547-552.
[12] L.C.a.R.I.o.D.-I.L.I.I.B.M.N.I.o.D.a.D.a.K.D.-N.U. 2020 Mar 10]. Available from: https://www.ncbi.nlm.nih.gov/books/NBK547850/, (Accessed at January 6,. 2022.).
[13] E. Adikwu, Fluoroquinolones Reported Hepatotoxicity. *J. Pharm. Pharmacol.* 03 (2012) 328-336.
[14] A.K. Jain, D. Singh, K. Dubey, R. Maurya, S. Mittal, A.K. Pandey, Chapter 3 - Models and Methods for In Vitro Toxicity, in: A. Dhawan, S. Kwon (Eds.). *In Vitro Toxicol.* Academic Press2018, pp. 45-65.
[15] http://www.swissadme.ch/, (Accessed at January 12,. 2022.).
[16] J.B.T.D.J. Taylor, Comprehensive medicinal chemistry II, (2007).
[17] A. Zerroug, S. Belaidi, I. BenBrahim, L. Sinha, S. Chtita, Virtual screening in drug-likeness and structure/activity relationship of pyridazine derivatives as Anti-Alzheimer drugs. *J. King Saud Univ. Sci.* 31(4) (2019) 595-601.

[18] https://www.rcsb.org/structure/6MOD, (Accessed at January 10,. 2022.).
[19] F. Cohen, J.B. Aggen, L.D. Andrews, Z. Assar, J. Boggs, T. Choi, P. Dozzo, A.N. Easterday, C.M. Haglund, D.J. Hildebrandt, M.C. Holt, K. Joly, A. Jubb, Z. Kamal, T.R. Kane, A.W. Konradi, K.M. Krause, M.S. Linsell, T.D. Machajewski, O. Miroshnikova, H.E. Moser, V. Nieto, T. Phan, C. Plato, A.W. Serio, J. Seroogy, A. Shakhmin, A.J. Stein, A.D. Sun, S. Sviridov, Z. Wang, K. Wlasichuk, W. Yang, X. Zhou, H. Zhu, R.T. Cirz, Optimization of LpxC Inhibitors for Antibacterial Activity and Cardiovascular Safety. *ChemMedChem*. 14(16) (2019) 1560-1572.
[20] https://www.rcsb.org/structure/6fv4 (Accessed at November 5,. 2021).
[21] K. Khan, K. Khan, R. Siddiqui, N. Ambreen, N. Sultana, S. Tauseef, A. Ahmad, S. Perveen, P. Dr, H. Khan, Synthesis, antibacterial and antifungal evaluation of norfloxacin derivatives. *J. Pharm. Res.* 55 (2012) 92.
[22] S.A. Gamal El-din Abuo-rahma , Mai Shoman , Ebtihal Samır , Rehab Abdel-baky, New N -4 piperazinyl derivatives of norfloxacin: design, synthesis, and correlation of calculated physicochemical parameters with antibacterial activity. *Turk. J. Chem.* 42 (4) (2018) 1072 - 1085.
[23] Z. Yu, G. Shi, Q. Sun, H. Jin, Y. Teng, K. Tao, G. Zhou, W. Liu, F. Wen, T. Hou, Design, synthesis and in vitro antibacterial/antifungal evaluation of novel 1-ethyl-6-fluoro-1,4-dihydro-4-oxo-7(1-piperazinyl)quinoline-3-carboxylic acid derivatives. *Eur. J. Med. Chem.* 44(11) (2009) 4726-33.
[24] https://pubchem.ncbi.nlm.nih.gov/compound/15993159, (Accessed on March 23,.2021).
[25] M.J. Nieto, F.d.L. Alovero, R.H. Manzo, M.R. Mazzieri, A new class of fluoroquinolones: benzenesulfonamidefluoroquinolones (BSFQs), antibacterial activity and SAR studies. *Eur. J. Med. Chem.* 34(3) (1999) 209-214.
[26] https://www.rcsb.org/structure/5CDQ, ((Accessed at March 27,. 2022)).
[27] https://www.rcsb.org/structure/2XKK, ((Accessed at March 23,.2022)).
[28] M.R. Burrell, N.P. Burton, A. Maxwell, A high-throughput assay for DNA topoisomerases and other enzymes, based on DNA triplex formation. *Methods Mol. Bio.* (Clifton, N.J.) 613 (2010) 257-66.
[29] Y.R.P.H.S.S.M. Farhat, pH 7.4." Protocol Place. Dec 2013. <http://protocol-place.com>, (Accessed on 5/20/2022.).
[30] M.L. Bolognesi, A. Cavalli, L. Valgimigli, M. Bartolini, M. Rosini, V. Andrisano, M. Recanatini, C. Melchiorre, Multi-target-directed drug design strategy: from a dual binding site acetylcholinesterase inhibitor to a trifunctional compound against Alzheimer's disease. *J. Med. Chem.* 50(26) (2007) 6446-9.
[31] C. Performance Standards for Antimicrobial Susceptibility Testing; 23rd Informational Supplement M100-S23. 2013, Wayne, PA, USA.
[32] C.A. Albada HB, Wenzel M, Penkova M, Bandow JE, Sahl HG, Metzler-Nolte N, Modulating the activity of short arginine-tryptophan containing antibacterial peptides with N-terminal metallocenoyl groups. *Beilstein J. Org. Chem.* 8 (2012) 1753–1764.
[33] W.J. Schön T, Machado D, Borroni E, Wijkander M, Lina G, Mouton J, Matuschek E, Kahlmeter G, Giske C, Santin M, Cirillo DM, Viveiros M, Cambau E, Antimicrobial susceptibility testing of Mycobacterium tuberculosis complex isolates; the EUCAST broth microdilution reference method for MIC determination. *Clin. Microbiol. Infect.* : the official publication of the European Society of Clinical Microbiology and Infectious Diseases 26 (2020) 1488–1492.
[34] A.-C.I. Schindelin J, Frise E, Kaynig V, Longair M, Pietzsch T, Preibisch S, Rueden C, Saalfeld S, Schmid B, Tinevez JY, White DJ, Hartenstein V, Eliceiri K, Tomancak P, Cardona A, Fiji: An open-source platform for biological-image analysis. *Nat. Methods.* 9 (2012) 676–682.

[35] A. Ducret, E.M. Quardokus, Y.V. Brun, MicrobeJ, a tool for high throughput bacterial cell detection and quantitative analysis. *Nat. Microbiol*. 1(7) (2016) 16077.
[36] G.D. Winkel JD, Seistrup KH, Hamoen LW, Strahl H, Analysis of antimicrobial-triggered membrane depolarisation using voltage sensitive dyes. *Front. Cell Dev. Biol*. 4:29 (2016).

[37] S. P, Expanding the spectrum of activity of mupirocin to include gram-negative bacteria using cationic steroid antibiotics. *J. Am. Acad. Dermatol.* 52 (2005) P7.
[38] H.K. Schmidt S, Melzig MF, Bereswill S, Heimesaat MM, Glycyrrhizic Acid Decreases Gentamicin-Resistance in Vancomycin-Resistant Enterococci, Planta Med 82 (2016) 1540–1545.
[39] F. Führer, S. Langklotz, F. Narberhaus, The C-terminal end of LpxC is required for degradation by the FtsH protease. *Mol. Microbiol.* 59(3) (2006) 1025-36.
[40] K.M. Krause, C.M. Haglund, C. Hebner, A.W. Serio, G. Lee, V. Nieto, F. Cohen, T.R. Kane, T.D. Machajewski, D. Hildebrandt, C. Pillar, M. Thwaites, D. Hall, L. Miesel, M. Hackel, A. Burek, L.D. Andrews, E. Armstrong, L. Swem, A. Jubb, R.T. Cirz, Potent LpxC Inhibitors with In Vitro Activity against Multidrug-Resistant Pseudomonas aeruginosa. *Antimicrob. Agents Chemother*. 63(11) (2019).
[41] K.T. Schneider T, Wimmer R, Wiedemann I, Sass V, Pag U, Jansen A, Nielsen AK, Mygind PH, Raventós DS, Neve S, Ravn B, Bonvin AMJJ, De Maria L, Andersen AS, Gammelgaard LK, Sahl H-GH-G, Kristensen H-HH-H, Raventos DS, Neve S, Ravn B, Bonvin AMJJ, De Maria L, Andersen AS, Gammelgaard LK, Sahl H-GH-G, Kristensen H-HH-H, Plectasin, a fungal defensin, targets the bacterial cell wall precursor Lipid II. *Science* 328 (2010) 1168–1172.
[42] K.B. Wenzel M, Münch D, Raatschen N, Albada HB, Hamoen L, Metzler-Nolte N, Sahl HG, Bandow JE, Proteomic response of Bacillus subtilis to lantibiotics reflects differences in interaction with the cytoplasmic membrane. *Antimicrob. Agents Chemother.* 56 (2012) 5749–5757.
[43] M.N. Liu X, Bouhss A, den Blaauwen T, FtsW activity and lipid II synthesis are required for recruitment of MurJ to midcell during cell division in Escherichia coli. *Mol. Microbiol.* 109 (2018) 855–884.
[44] B.K. Larsen MH, Chen B, Hsu T, Sambandamurthy VK, Lackner AA, Aye PP, Didier P, Huang D, Shao L, Wei H, Letvin NL, Frothingham R, Haynes BF, Chen ZW, Jacobs Jr WR, Efficacy and safety of live attenuated persistent and rapidly cleared Mycobacterium tuberculosis vaccine candidates in non-human primates. *Vaccine* 27 (2009) 4709–4717.
[45] A. Müller, M. Wenzel, H. Strahl, F. Grein, T.N.V. Saaki, B. Kohl, T. Siersma, J.E. Bandow, H.G. Sahl, T. Schneider, L.W. Hamoen, Daptomycin inhibits cell envelope synthesis by interfering with fluid membrane microdomains. *Proc. Natl. Acad. Sci. U. S. A.* 113(45) (2016) E7077-e7086.
